# Supplementary material for: Does curve pattern impact on the effects of physiotherapeutic scoliosis specific exercises on Cobb angles of participants with adolescent idiopathic scoliosis: A prospective clinical trial with two years follow-up
Source: PLoS One. 2021 Jan 25;16(1):e0245829. doi: 10.1371/journal.pone.0245829 (PMC7833215; doi:10.1371/journal.pone.0245829)
Supplement: S1 File — (PDF) [file pone.0245829.s001.pdf]

THE UNIVERSITY OF HONG KONG – SHENZHEN HOSPITAL

STUDY PROTOCOL

1. Project Title:

1.1 Original Tile used for IRB Approval and Clinical Registration

Schroth 脊柱侧弯 3D 矫正运动对于脊柱侧弯的治疗作用*

Effects of Physiotherapeutic Scoliosis Specific Exercise – Shcroth approach for subjects with Adolescent Idiopathic Scoliosis*

1.2 Tile in manuscript prepared for publication

Dose Curve Pattern Impact on the Effects of Physiotherapeutic Scoliosis Specific Exercise on the Cobb Angle in Adolescent Idiopathic Scoliosis Subjects: A prospective clinical trial with a two-year follow-up #

2. Project Objectives: (Purpose of proposed investigation)

This project aims to investigate the long-term therapeutic effect of Physiotherapeutic Scoliosis Specific Exercise （PSSE） on thoracic and lumbar major curves and to determine whether a more flexible lumbar curve can respond better to exercise.

3. Scope and Background of Research:

Adolescent idiopathic scoliosis (AIS) is a three-dimensional spinal deformity with an unknown etiology, characterized by lateral deviation in the frontal plane, axial rotation in the horizontal plane and an abnormal sagittal curvature[1]. Surgery is usually recommended if the curve reaches 50 degrees, as this is associated with a continued progression risk into adulthood[2]. Surgical fusion undoubtedly leads to stiff spines and should be avoided if possible. Thus, the goal of conservative management is to prevent spinal deformity deterioration into the operative threshold.

The option for nonsurgical management varies widely and depends on the strength of the prognostic evaluation of curve progression[3]. The curve type is one of the established risk factors of scoliosis progression[4]. In particular, a larger Cobb angle with the presence of a thoracic curve shows higher progression odds than a single lumbar or thoracolumbar curve[4]. Therefore, understanding the impact of curvature on intervention is valuable and helpful for clinicians to choose proper treatments for patients. Correction of deformities is highly influenced by increased mobility of spinal segments[5-9]. In addition, scoliosis-specific exercise is a nonoperative method that is well received by patients and parents[10]. There are several systematic reviews and randomized controlled trials reporting the positive effects of Physiotherapeutic Scoliosis Specific Exercise (PSSE) on slowing the curve progression as well as improving the cosmetic appearance and quality of life (QOL) outcomes[11-13]; however, in these studies, the relationship of curve location with correction effects was not clearly discussed, and there was only a short-term follow-up. Specifically, Schroth is the most commonly used PSSE approach, which adopts specific respiratory technique-asymmetrical breathing in the diagonal direction for vertebral and rib cage derotation. It utilizes muscle activation and emphasizes core muscle stabilization of the corrected posture throughout the day to change habitual postures and improve spinal alignment[14]. However, the influence of curve magnitude on exercise outcomes is unknown. The relationship between spinal flexibility and PSSE is also not well established. It is unclear which profile of spinal deformity leads to the best outcomes with PSSE. Thus, this study aimed to investigate the long-term therapeutic effect of PSSE on thoracic and

remains the same study with *

lumbar major curves and to determine whether a more flexible lumbar curve would respond better to exercise. The results of this study will provide physiotherapists with additional information to evaluate individualized, curve magnitude-based exercises for patients and will form the basis for further controlled trials to evaluate the relationship between spinal flexibility and PSSE.

4. Research Methodology:

Study Design

This is a prospective, clinical controlled trial based in the department of physiotherapy. Subjects are initially recruited from the Out-patient clinic, department of orthopedics and referred to physiotherapy department for PSSE program. Subjects should agree and sign with consent form before commencing the study. The inclusion criteria are as follows: 1) age: 10 to 16 years, 2) bone immaturity in terms of Sanders stage: less than 8[16], 3) Cobb angles: from 10 degrees to 50 degrees, 4) be able to complete all physiotherapy sessions. Exclusion criteria are 1) diagnoses other than AIS, 2) disabilities or systemic illnesses preventing exercise performance, 3) hypermobility (Beighton score[17] greater than 4) and 4) previous treatment for AIS.

Study Intervention

The Rigo Scoliosis Classification system is applied to categorize subjects and define the appropriate exercise program[18]. Subjects with 3C (thoracic major with or without a minor lumbar) or N3N4 curves (double curves but well balanced) are considered thoracic major (group A), and those with single lumbar/single thoracolumbar or 4C curves (lumbar major with a minor thoracic) are considered lumbar major (group B).

1.1 PSSE Protocol

The PSSE intervention consists of 50 breaths per exercise. Short semi-hanging, corrective exercises in the standing, sitting and lateral positions will be prescribed for subjects in group A. Short semi-hanging, muscle cylinder, corrective exercises in the lateral, sitting and standing positions will be prescribed to subjects in group B. The difference between the two groups is that a muscle cylinder will be only applied to the lumbar major patients (group B). Subjects are asked to record and report any discomfort, such as muscle fatigue or muscle sprain, to the on-duty physiotherapist during the study period.

1.2 Exercise Intensity and Compliance

The frequency and time of supervised treatment during the 6 months are ≥1 time per month and one hour, each time. The subjects are instructed to perform >30 minutes of home exercise/session with ≥5 sessions a week. After the first 6 months of PSSE, all subjects can reduce the frequency of supervised PSSE to once per three months to review the exercises performed until two years after completion of the PSSE program. During this time, the frequency of home exercise should be maintained. Attendance of supervised PSSE in the outpatient setting will be collected from the hospital prospective database system. Each session of treatment is one hour. One simple questionnaire will be completed by all subjects themselves to check their home exercise compliance. Exercise compliance is calculated and documented in hours per week for each year.

remains the same study with *

Outcome Measurement

Subjects will receive whole-spine standing radiographs to assess their major curve Cobb angles. The assessor of the images will be blinded to the patient information and treatment details. The Cobb angle is collected at the initial assessment and every 6 months after the PSSE program until the two-year follow-up. All subjects remain out of their braces overnight prior to obtaining radiographs.

Statistics

1.1 Sample Size Estimation

The sample size estimation was conducted before commencing the study using the software Gpower3.1. The effect size was adopted from the study conducted by Sanjia etl. in 2016 [19] which showed significant Cobb reduction after six months of PSSE (PSSE group: 1.4+/-0.2 degree; Standard care group: 1.2+/-0.2 degrees, per protocol analysis of changes from the biggest curves), with 80% power using a two-tailed 0.05 hypothesis test, 17 subjects were required in each group, considering of dropouts during long term follow-up, 20 subjects in each group was planned.

1.2 Outcome data analysis

Student's t test is used to compare continuous variables, including age and initial Cobb angles. The chi-square test is used to compare categorical variables including gender, Sanders stage, curvature types (single or double curves) and brace treatment for all subjects. The absolute D-value of the change in the Cobb angle during the study period between the two groups will be compared with repeated measures one-way analysis of variance (ANOVA), and intragroup analysis by multiple paired t tests is performed to detect any curve regression outcome differences in patients with similar curve patterns. The data was analyzed using SPSS version 20.0 (IBM, Chicago, IL). The level of significance is set to 0.05 with a two-tailed test.

4.2 Time Schedule

Project Progress	Time Period
**Proposal writing and finalization**	Nov-Dec 2016
**Ethics application**	Dec 2016 – Jan 2017
**Main Study**	
**Subjects Enrolment**	Jan 2017-Jun 2017
**Data Collection**	Dec 2017-Dec 2019
**Report Writing**	Nov-Dec 2019

5. Project Significance and Value

The results of this study will provide physiotherapists with additional information to evaluate individualized, curve magnitude-based exercises for patients and will form the basis for further controlled trials to evaluate the relationship between spinal flexibility and PSSE.

6. Details of Any External Collaboration

No external collaboration was required.

remains the same study with *

References

1. Cheng JC, Castelein RM, Chu WC, Danielsson AJ, Dobbs MB, Grivas TB, et al. Adolescent idiopathic scoliosis. Nature reviews Disease primers. 2015;1:15030. Epub 2015/01/01. doi: 10.1038/nrdp.2015.30. PubMed PMID: 27188385.
2. Weinstein SL. The Natural History of Adolescent Idiopathic Scoliosis. Journal of pediatric orthopedics. 2019;39(Issue 6, Supplement 1 Suppl 1):S44-s6. Epub 2019/06/07. doi: 10.1097/bpo.0000000000001350. PubMed PMID: 31169647.
3. Sy N, Bettany-Saltikov J, Moramarco M. Evidence for Conservative Treatment of Adolescent Idiopathic Scoliosis - Update 2015 (Mini-Review). Current pediatric reviews. 2016;12(1):6-11. Epub 2015/11/18. PubMed PMID: 26573167.
4. Dolan LA, Weinstein SL, Abel MF, Bosch PP, Dobbs MB, Farber TO, et al. Bracing in Adolescent Idiopathic Scoliosis Trial (BrAIST): Development and Validation of a Prognostic Model in Untreated Adolescent Idiopathic Scoliosis Using the Simplified Skeletal Maturity System. Spine Deform. 2019;7(6):890-8.e4. Epub 2019/11/17. doi: 10.1016/j.jspd.2019.01.011. PubMed PMID: 31731999.
5. Almansour H, Pepke W, Bruckner T, Diebo BG, Akbar M. Three-Dimensional Analysis of Initial Brace Correction in the Setting of Adolescent Idiopathic Scoliosis. Journal of clinical medicine. 2019;8(11). Epub 2019/10/31. doi: 10.3390/jcm8111804. PubMed PMID: 31661811.
6. He C, To MK, Cheung JP, Cheung KM, Chan CK, Jiang WW, et al. An effective assessment method of spinal flexibility to predict the initial in-orthosis correction on the patients with adolescent idiopathic scoliosis (AIS). PloS one. 2017;12(12):e0190141. Epub 2017/12/22. doi: 10.1371/journal.pone.0190141. PubMed PMID: 29267389; PubMed Central PMCID: PMCPMC5739463.
7. He C, Wong MS. Spinal Flexibility Assessment on the Patients With Adolescent Idiopathic Scoliosis: A Literature Review. Spine. 2018;43(4):E250-e8. Epub 2017/06/13. doi: 10.1097/brs.0000000000002276. PubMed PMID: 28604491.
8. Ohrt-Nissen S, Hallager DW, Gehrchen M, Dahl B. Flexibility Predicts Curve Progression in Providence Nighttime Bracing of Patients With Adolescent Idiopathic Scoliosis. Spine. 2016;41(22):1724-30. Epub 2016/04/15. doi: 10.1097/brs.0000000000001634. PubMed PMID: 27076435.
9. Yao G, Cheung JPY, Shigematsu H, Ohrt-Nissen S, Cheung KMC, Luk KDK, et al. Characterization and Predictive Value of Segmental Curve Flexibility in Adolescent Idiopathic Scoliosis Patients. Spine. 2017;42(21):1622-8. Epub 2016/12/21. doi: 10.1097/brs.0000000000002046. PubMed PMID: 27997505.
10. Negrini S, Carabalona R. Social acceptability of treatments for adolescent idiopathic scoliosis: a cross-sectional study. Scoliosis. 2006;1:14. Epub 2006/08/26. doi: 10.1186/1748-7161-1-14. PubMed PMID: 16930488; PubMed Central PMCID: PMCPMC1560163.
11. Burger M, Coetzee W, du Plessis LZ, Geldenhuys L, Joubert F, Myburgh E, et al. The effectiveness of Schroth exercises in adolescents with idiopathic scoliosis: A systematic review and meta-analysis. The South African journal of physiotherapy. 2019;75(1):904. Epub 2019/06/18. doi: 10.4102/sajp.v75i1.904. PubMed PMID: 31206094; PubMed Central PMCID: PMCPMC6556933.
12. Ceballos Laita L, Tejedor Cubillo C, Mingo Gomez T, Jimenez Del Barrio S. Effects of corrective, therapeutic exercise techniques on adolescent idiopathic scoliosis. A systematic review. Archivos argentinos de pediatria. 2018;116(4):e582-e9. Epub 2018/07/18. doi: 10.5546/aap.2018.eng.e582. PubMed PMID: 30016036.
13. Thompson JY, Williamson EM, Williams MA, Heine PJ, Lamb SE. Effectiveness of scoliosis-specific exercises for adolescent idiopathic scoliosis compared with other non-surgical interventions: a systematic review and meta-analysis. Physiotherapy. 2019;105(2):214-34. Epub 2019/03/03. doi: 10.1016/j.physio.2018.10.004. PubMed PMID: 30824243.
14. Lehnert-Schroth C. [Schroth's three dimensional treatment of scoliosis]. ZFA Zeitschrift fur Allgemeinmedizin. 1979;55(34):1969-76. Epub 1979/12/10. PubMed PMID: 547573.
15. Negrini S, Donzelli S, Aulisa AG, Czaprowski D, Schreiber S, de Mauroy JC, et al. 2016 SOSORT

remains the same study with *

guidelines: orthopaedic and rehabilitation treatment of idiopathic scoliosis during growth. Scoliosis and spinal disorders. 2018;13:3. Epub 2018/02/13. doi: 10.1186/s13013-017-0145-8. PubMed PMID: 29435499; PubMed Central PMCID: PMCPMC5795289.

16. Sitoula P, Verma K, Holmes L, Jr., Gabos PG, Sanders JO, Yorgova P, et al. Prediction of Curve Progression in Idiopathic Scoliosis: Validation of the Sanders Skeletal Maturity Staging System. Spine. 2015;40(13):1006-13. Epub 2015/09/12. doi: 10.1097/brs.0000000000000952. PubMed PMID: 26356067.
17. Smits-Engelsman B, Klerks M, Kirby A. Beighton score: a valid measure for generalized hypermobility in children. The Journal of pediatrics. 2011;158(1):119-23, 23.e1-4. Epub 2010/09/21. doi: 10.1016/j.jpeds.2010.07.021. PubMed PMID: 20850761.
18. Rigo MD, Villagrasa M, Gallo D. A specific scoliosis classification correlating with brace treatment: description and reliability. Scoliosis. 2010;5(1):1. Epub 2010/03/09. doi: 10.1186/1748-7161-5-1. PubMed PMID: 20205842; PubMed Central PMCID: PMCPMC2825498.
19. Schreiber S, Parent EC, Khodayari Moez E, Hedden DM, Hill DL, Moreau M, Lou E, Watkins EM, Southon SC (2016) Schroth Physiotherapeutic Scoliosis-Specific Exercises Added to the Standard of Care Lead to Better Cobb Angle Outcomes in Adolescents with Idiopathic Scoliosis - an Assessor and Statistician Blinded Randomized Controlled Trial. PloS one 11:e0168746. doi: 10.1371/journal.pone.0168746

7. Declaration of the first author

I sincerely declare that this protocol identically explained study methodology with the original protocol in Chinese (Supplementary document 1). The ethic approval was obtained before study.

Signature ____________ Date 21st Jan 2020

Name FAN Yunli

8a. Research Ethics/ Safety Approval

I confirm that approval:

	* has been Obtained	* is not required	* will be obtained before the start of the project
Human Research Ethics	✓		
Animal Research Ethics		✓	
Biological Safety		✓	
Ionizing Radiation Safety		✓	
Non-ionizing Radiation Safety		✓	
Chemical Safety		✓	

remains the same study with *

8b. Research Facilities and Space

☑ I confirm, to the best of my knowledge, that adequate facilities and space are available to conduct and complete the study in an efficient and safe manner.

☐ I would like to request the following additional research facilities and/or space to conduct and complete the study in an efficient and safe manner:

Research Facilities	PSSE-Schroth method facility set up for subjects
Space (Other than the regular space provided by the Department of Physiotherapy)	No

Signature [signature] (Department manager) Experimental Site: The Department of Physiotherapy

Name Eric, HK Yeung Date 22nd Jan 2020

remains the same study with *
