# Supplementary material for: Does curve pattern impact on the effects of physiotherapeutic scoliosis specific exercises on Cobb angles of participants with adolescent idiopathic scoliosis: A prospective clinical trial with two years follow-up
Source: PLoS One. 2021 Jan 25;16(1):e0245829. doi: 10.1371/journal.pone.0245829 (PMC7833215; doi:10.1371/journal.pone.0245829)
Supplement: S2 File — (PDF) [file pone.0245829.s002.pdf]

# Home Exercise (HE) Adherence Checklist

The Department of Physiotherapy, The University of Hong Kong - Shenzhen Hospital, China

**Name:** **Age:** **Sex:** **Medical No.:** **Brace:** ☐ **yes** ☐**no**

PHYSIOTHERAPIST ONLY

**Scoliosis classification:** ☐ Major thoracic ☐ Major lumbar ☐ Single Thoracic ☐ Single lumbar
☐ Thoracolumbar ☐ Double thoracic ☐ Double major

**Precautions (if any):**

**Home exercise (HE) protocol:**

☐ semi-hanging ☐ corrective exercise in sitting ☐ corrective exercise in standing
☐ corrective exercise in supine ☐ corrective exercise in prone ☐ muscle cylinder
☐ corrective exercise in side-lying ☐ others:

**Home exercise (HE) dosage:**

☐ _______long of either four of ticked exercises for one day, ≥5 days per week (recommended)
☐ others:

***Scheduled next visit (dd/mm/year): / /**

**Physiotherapist:**

**Date:**

PATIENT/PARENTS ONLY: **Please return this HE adherence checklist to the therapist at your next visit.**

| | When (dd/mm/year)<br>*The date that you did the HE. | How long<br>*The minutes that you spend on the HE. | | | Brace compliance (hours/day)<br>*For braced children only | |
|---|---|---|---|---|---|---|
| **1** | __ __/__ __/20__ __ | ☐ 1hr | ☐30min | ☐other:_____min | ☐ 21 | ☐other:_____ |
| **2** | __ __/__ __/20__ __ | ☐ 1hr | ☐30min | ☐other:_____min | ☐ 21 | ☐other:_____ |
| **3** | __ __/__ __/20__ __ | ☐ 1hr | ☐30min | ☐other:_____min | ☐ 21 | ☐other:_____ |
| **4** | __ __/__ __/20__ __ | ☐ 1hr | ☐30min | ☐other:_____min | ☐ 21 | ☐other:_____ |
| **5** | __ __/__ __/20__ __ | ☐ 1hr | ☐30min | ☐other:_____min | ☐ 21 | ☐other:_____ |
| **6** | __ __/__ __/20__ __ | ☐ 1hr | ☐30min | ☐other:_____min | ☐ 21 | ☐other:_____ |
| **7** | __ __/__ __/20__ __ | ☐ 1hr | ☐30min | ☐other:_____min | ☐ 21 | ☐other:_____ |
| **8** | __ __/__ __/20__ __ | ☐ 1hr | ☐30min | ☐other:_____min | ☐ 21 | ☐other:_____ |
| **9** | __ __/__ __/20__ __ | ☐ 1hr | ☐30min | ☐other:_____min | ☐ 21 | ☐other:_____ |
| **10** | __ __/__ __/20__ __ | ☐ 1hr | ☐30min | ☐other:_____min | ☐ 21 | ☐other:_____ |
| **11** | __ __/__ __/20__ __ | ☐ 1hr | ☐30min | ☐other:_____min | ☐ 21 | ☐other:_____ |
| **12** | __ __/__ __/20__ __ | ☐ 1hr | ☐30min | ☐other:_____min | ☐ 21 | ☐other:_____ |
| **13** | __ __/__ __/20__ __ | ☐ 1hr | ☐30min | ☐other:_____min | ☐ 21 | ☐other:_____ |
| **14** | __ __/__ __/20__ __ | ☐ 1hr | ☐30min | ☐other:_____min | ☐ 21 | ☐other:_____ |
| **15** | __ __/__ __/20__ __ | ☐ 1hr | ☐30min | ☐other:_____min | ☐ 21 | ☐other:_____ |
| **16** | __ __/__ __/20__ __ | ☐ 1hr | ☐30min | ☐other:_____min | ☐ 21 | ☐other:_____ |
| **17** | __ __/__ __/20__ __ | ☐ 1hr | ☐30min | ☐other:_____min | ☐ 21 | ☐other:_____ |
| **18** | __ __/__ __/20__ __ | ☐ 1hr | ☐30min | ☐other:_____min | ☐ 21 | ☐other:_____ |
| **19** | __ __/__ __/20__ __ | ☐ 1hr | ☐30min | ☐other:_____min | ☐ 21 | ☐other:_____ |
| **20** | __ __/__ __/20__ __ | ☐ 1hr | ☐30min | ☐other:_____min | ☐ 21 | ☐other:_____ |
| **21** | __ __/__ __/20__ __ | ☐ 1hr | ☐30min | ☐other:_____min | ☐ 21 | ☐other:_____ |
| **22** | __ __/__ __/20__ __ | ☐ 1hr | ☐30min | ☐other:_____min | ☐ 21 | ☐other:_____ |
| **23** | __ __/__ __/20__ __ | ☐ 1hr | ☐30min | ☐other:_____min | ☐ 21 | ☐other:_____ |
| **24** | __ __/__ __/20__ __ | ☐ 1hr | ☐30min | ☐other:_____min | ☐ 21 | ☐other:_____ |
| **25** | __ __/__ __/20__ __ | ☐ 1hr | ☐30min | ☐other:_____min | ☐ 21 | ☐other:_____ |
| **26** | __ __/__ __/20__ __ | ☐ 1hr | ☐30min | ☐other:_____min | ☐ 21 | ☐other:_____ |
