# Supplementary material for: Does curve pattern impact on the effects of physiotherapeutic scoliosis specific exercises on Cobb angles of participants with adolescent idiopathic scoliosis: A prospective clinical trial with two years follow-up
Source: PLoS One. 2021 Jan 25;16(1):e0245829. doi: 10.1371/journal.pone.0245829 (PMC7833215; doi:10.1371/journal.pone.0245829)
Supplement: S4 File — (RTF) [file pone.0245829.s004.rtf]

GET
  FILE='D:\curvepattern191205.sav'.
DATASET NAME DataSet1 WINDOW=FRONT.
GLM InitialMajorCobb OneYrCobb One_HalfYrCobb TwoYrCobb BY GroupNo WITH BracingHours
  /WSFACTOR=factor1 4 Polynomial
  /METHOD=SSTYPE(3)
  /EMMEANS=TABLES(OVERALL) WITH(BracingHours=MEAN)
  /EMMEANS=TABLES(GroupNo) WITH(BracingHours=MEAN)COMPARE ADJ(BONFERRONI)
  /EMMEANS=TABLES(factor1) WITH(BracingHours=MEAN)COMPARE ADJ(BONFERRONI)
  /EMMEANS=TABLES(GroupNo*factor1) WITH(BracingHours=MEAN)
  /PRINT=DESCRIPTIVE ETASQ OPOWER HOMOGENEITY
  /CRITERIA=ALPHA(.05)
  /WSDESIGN=factor1
  /DESIGN=BracingHours GroupNo.


General Linear Model


Notes	
Output Created	02-DEC-2020 21:26:55	
Comments		
Input	Data	D:\curvepattern191205.sav	
	Active Dataset	DataSet1	
	Filter	<none>	
	Weight	<none>	
	Split File	<none>	
	N of Rows in Working Data File	40	
Missing Value Handling	Definition of Missing	User-defined missing values are treated as missing.	
	Cases Used	Statistics are based on all cases with valid data for all variables in the model.	

Notes	
Syntax	GLM InitialMajorCobb OneYrCobb One_HalfYrCobb TwoYrCobb BY GroupNo WITH BracingHours
  /WSFACTOR=factor1 4 Polynomial
  /METHOD=SSTYPE(3)
  /EMMEANS=TABLES(OVERALL) WITH(BracingHours=MEAN)
  /EMMEANS=TABLES(GroupNo) WITH(BracingHours=MEAN)COMPARE ADJ(BONFERRONI)
  /EMMEANS=TABLES(factor1) WITH(BracingHours=MEAN)COMPARE ADJ(BONFERRONI)
  /EMMEANS=TABLES(GroupNo*factor1) WITH(BracingHours=MEAN)
  /PRINT=DESCRIPTIVE ETASQ OPOWER HOMOGENEITY
  /CRITERIA=ALPHA(.05)
  /WSDESIGN=factor1
  /DESIGN=BracingHours GroupNo.	
Resources	Processor Time	00:00:00.08	
	Elapsed Time	00:00:00.11	


[DataSet1] D:\curvepattern191205.sav


Within-Subjects Factors	
Measure: MEASURE_1	
factor1	Dependent Variable	
1	InitialMajorCobb	
2	OneYrCobb	
3	One_HalfYrCobb	
4	TwoYrCobb	


Between-Subjects Factors	
	Value Label	N	
Group No.	1	Group A	22	
	2	Group B	18	


Descriptive Statistics	
	Group No.	Mean	Std. Deviation	N	
Initial Cobb angle of Major curve	Group A	28.500	8.4219	22	
	Group B	26.778	10.1086	18	
	Total	27.725	9.1371	40	
Cobb of 1yr after PSSE	Group A	27.000	7.2045	22	
	Group B	24.722	9.4047	18	
	Total	25.975	8.2353	40	
Cobb of 1.5 after PSSE	Group A	27.091	7.6589	22	
	Group B	23.278	9.4172	18	
	Total	25.375	8.5984	40	
Cobb of 2yrs after PSSE	Group A	27.455	6.9606	22	
	Group B	22.944	9.1553	18	
	Total	25.425	8.2334	40	


Box's Test of Equality of Covariance Matricesa	
Box's M	15.193	
F	1.342	
df1	10	
df2	6264.265	
Sig.	.201	

Tests the null hypothesis that the observed covariance matrices of the dependent variables are equal across groups.a	
a. Design: Intercept + BracingHours + GroupNo 
 Within Subjects Design: factor1	


Multivariate Testsa	
Effect	Value	F	Hypothesis df	Error df	
factor1	Pillai's Trace	.173	2.436b	3.000	35.000	
	Wilks' Lambda	.827	2.436b	3.000	35.000	
	Hotelling's Trace	.209	2.436b	3.000	35.000	
	Roy's Largest Root	.209	2.436b	3.000	35.000	
factor1 * BracingHours	Pillai's Trace	.113	1.491b	3.000	35.000	
	Wilks' Lambda	.887	1.491b	3.000	35.000	
	Hotelling's Trace	.128	1.491b	3.000	35.000	
	Roy's Largest Root	.128	1.491b	3.000	35.000	
factor1 * GroupNo	Pillai's Trace	.163	2.279b	3.000	35.000	
	Wilks' Lambda	.837	2.279b	3.000	35.000	
	Hotelling's Trace	.195	2.279b	3.000	35.000	
	Roy's Largest Root	.195	2.279b	3.000	35.000	

Multivariate Testsa	
Effect	Sig.	Partial Eta Squared	Noncent. Parameter	Observed Power	
factor1	Pillai's Trace	.081	.173b	7.308	.557	
	Wilks' Lambda	.081	.173b	7.308	.557	
	Hotelling's Trace	.081	.173b	7.308	.557	
	Roy's Largest Root	.081	.173b	7.308	.557	
factor1 * BracingHours	Pillai's Trace	.234	.113b	4.474	.359	
	Wilks' Lambda	.234	.113b	4.474	.359	
	Hotelling's Trace	.234	.113b	4.474	.359	
	Roy's Largest Root	.234	.113b	4.474	.359	
factor1 * GroupNo	Pillai's Trace	.097	.163b	6.836	.527	
	Wilks' Lambda	.097	.163b	6.836	.527	
	Hotelling's Trace	.097	.163b	6.836	.527	
	Roy's Largest Root	.097	.163b	6.836	.527	

a. Design: Intercept + BracingHours + GroupNo 
 Within Subjects Design: factor1	
b. Exact statistic	
c. Computed using alpha = .05	


Mauchly's Test of Sphericitya	
Measure: MEASURE_1	
Within Subjects Effect	Mauchly's W	Approx. Chi-Square	df	Sig.	Epsilonb	
					Greenhouse-Geisser	
factor1	.409	31.979	5	.000	.719	

Mauchly's Test of Sphericitya	
Measure: MEASURE_1	
Within Subjects Effect	Epsilon	
	Huynh-Feldt	Lower-bound	
factor1	.806	.333	

Tests the null hypothesis that the error covariance matrix of the orthonormalized transformed dependent variables is proportional to an identity matrix.a	
a. Design: Intercept + BracingHours + GroupNo 
 Within Subjects Design: factor1	
b. May be used to adjust the degrees of freedom for the averaged tests of significance. Corrected tests are displayed in the Tests of Within-Subjects Effects table.	


Tests of Within-Subjects Effects	
Measure: MEASURE_1	
Source	Type III Sum of Squares	df	Mean Square	F	
factor1	Sphericity Assumed	93.030	3	31.010	4.112	
	Greenhouse-Geisser	93.030	2.157	43.139	4.112	
	Huynh-Feldt	93.030	2.418	38.470	4.112	
	Lower-bound	93.030	1.000	93.030	4.112	
factor1 * BracingHours	Sphericity Assumed	30.896	3	10.299	1.366	
	Greenhouse-Geisser	30.896	2.157	14.327	1.366	
	Huynh-Feldt	30.896	2.418	12.776	1.366	
	Lower-bound	30.896	1.000	30.896	1.366	
factor1 * GroupNo	Sphericity Assumed	52.827	3	17.609	2.335	
	Greenhouse-Geisser	52.827	2.157	24.496	2.335	
	Huynh-Feldt	52.827	2.418	21.845	2.335	
	Lower-bound	52.827	1.000	52.827	2.335	
Error(factor1)	Sphericity Assumed	837.013	111	7.541		
	Greenhouse-Geisser	837.013	79.791	10.490		
	Huynh-Feldt	837.013	89.476	9.355		
	Lower-bound	837.013	37.000	22.622		

Tests of Within-Subjects Effects	
Measure: MEASURE_1	
Source	Sig.	Partial Eta Squared	Noncent. Parameter	
factor1	Sphericity Assumed	.008	.100	12.337	
	Greenhouse-Geisser	.018	.100	8.868	
	Huynh-Feldt	.014	.100	9.945	
	Lower-bound	.050	.100	4.112	
factor1 * BracingHours	Sphericity Assumed	.257	.036	4.097	
	Greenhouse-Geisser	.261	.036	2.945	
	Huynh-Feldt	.260	.036	3.303	
	Lower-bound	.250	.036	1.366	
factor1 * GroupNo	Sphericity Assumed	.078	.059	7.006	
	Greenhouse-Geisser	.099	.059	5.036	
	Huynh-Feldt	.092	.059	5.647	
	Lower-bound	.135	.059	2.335	
Error(factor1)	Sphericity Assumed				
	Greenhouse-Geisser				
	Huynh-Feldt				
	Lower-bound				

Tests of Within-Subjects Effects	
Measure: MEASURE_1	
Source	Observed Power	
factor1	Sphericity Assumed	.837	
	Greenhouse-Geisser	.736	
	Huynh-Feldt	.772	
	Lower-bound	.506	
factor1 * BracingHours	Sphericity Assumed	.355	
	Greenhouse-Geisser	.297	
	Huynh-Feldt	.315	
	Lower-bound	.207	
factor1 * GroupNo	Sphericity Assumed	.573	
	Greenhouse-Geisser	.479	
	Huynh-Feldt	.510	
	Lower-bound	.319	
Error(factor1)	Sphericity Assumed		
	Greenhouse-Geisser		
	Huynh-Feldt		
	Lower-bound		

a. Computed using alpha = .05	


Tests of Within-Subjects Contrasts	
Measure: MEASURE_1	
Source	factor1	Type III Sum of Squares	df	Mean Square	F	Sig.	
factor1	Linear	78.177	1	78.177	7.717	.009	
	Quadratic	13.459	1	13.459	1.719	.198	
	Cubic	1.394	1	1.394	.299	.588	
factor1 * BracingHours	Linear	7.283	1	7.283	.719	.402	
	Quadratic	9.350	1	9.350	1.194	.282	
	Cubic	14.263	1	14.263	3.059	.089	
factor1 * GroupNo	Linear	52.071	1	52.071	5.140	.029	
	Quadratic	.012	1	.012	.002	.969	
	Cubic	.744	1	.744	.159	.692	
Error(factor1)	Linear	374.812	37	10.130			
	Quadratic	289.700	37	7.830			
	Cubic	172.500	37	4.662			

Tests of Within-Subjects Contrasts	
Measure: MEASURE_1	
Source	factor1	Partial Eta Squared	Noncent. Parameter	Observed Power	
factor1	Linear	.173	7.717	.772	
	Quadratic	.044	1.719	.248	
	Cubic	.008	.299	.083	
factor1 * BracingHours	Linear	.019	.719	.131	
	Quadratic	.031	1.194	.187	
	Cubic	.076	3.059	.399	
factor1 * GroupNo	Linear	.122	5.140	.598	
	Quadratic	.000	.002	.050	
	Cubic	.004	.159	.068	
Error(factor1)	Linear				
	Quadratic				
	Cubic				

a. Computed using alpha = .05	


Levene's Test of Equality of Error Variancesa	
	F	df1	df2	Sig.	
Initial Cobb angle of Major curve	.392	1	38	.535	
Cobb of 1yr after PSSE	2.605	1	38	.115	
Cobb of 1.5 after PSSE	1.794	1	38	.188	
Cobb of 2yrs after PSSE	2.922	1	38	.096	

Tests the null hypothesis that the error variance of the dependent variable is equal across groups.a	
a. Design: Intercept + BracingHours + GroupNo 
 Within Subjects Design: factor1	


Tests of Between-Subjects Effects	
Measure: MEASURE_1 
 Transformed Variable: Average	
Source	Type III Sum of Squares	df	Mean Square	F	Sig.	Partial Eta Squared	
Intercept	67718.237	1	67718.237	491.726	.000	.930	
BracingHours	5038.670	1	5038.670	36.588	.000	.497	
GroupNo	134.065	1	134.065	.973	.330	.026	
Error	5095.472	37	137.715				

Tests of Between-Subjects Effects	
Measure: MEASURE_1 
 Transformed Variable: Average	
Source	Noncent. Parameter	Observed Power	
Intercept	491.726	1.000	
BracingHours	36.588	1.000	
GroupNo	.973	.161	
Error			

a. Computed using alpha = .05	


Estimated Marginal Means


1. Grand Mean	
Measure: MEASURE_1	
Mean	Std. Error	95% Confidence Interval	
		Lower Bound	Upper Bound	
26.032a	.932	24.143	27.922	

a. Covariates appearing in the model are evaluated at the following values: BracingHours = 3.625.	


2. Group No.


Estimates	
Measure: MEASURE_1	
Group No.	Mean	Std. Error	95% Confidence Interval	
			Lower Bound	Upper Bound	
Group A	26.958a	1.254	24.416	29.499	
Group B	25.107a	1.388	22.296	27.918	

a. Covariates appearing in the model are evaluated at the following values: BracingHours = 3.625.	


Pairwise Comparisons	
Measure: MEASURE_1	
(I) Group No.	(J) Group No.	Mean Difference (I-J)	Std. Error	Sig.a	95% Confidence Interval for Differencea	
					Lower Bound	Upper Bound	
Group A	Group B	1.851	1.876	.330	-1.950	5.652	
Group B	Group A	-1.851	1.876	.330	-5.652	1.950	

Based on estimated marginal means	
a. Adjustment for multiple comparisons: Bonferroni.	


Univariate Tests	
Measure: MEASURE_1	
	Sum of Squares	df	Mean Square	F	Sig.	Partial Eta Squared	
Contrast	33.516	1	33.516	.973	.330	.026	
Error	1273.868	37	34.429				

Univariate Tests	
Measure: MEASURE_1	
	Noncent. Parameter	Observed Power	
Contrast	.973	.161	
Error			

The F tests the effect of Group No.. This test is based on the linearly independent pairwise comparisons among the estimated marginal means.	
a. Computed using alpha = .05	


3. factor1


Estimates	
Measure: MEASURE_1	
factor1	Mean	Std. Error	95% Confidence Interval	
			Lower Bound	Upper Bound	
1	27.708a	1.062	25.556	29.860	
2	25.917a	1.034	23.821	28.012	
3	25.247a	.995	23.230	27.263	
4	25.259a	.928	23.379	27.139	

a. Covariates appearing in the model are evaluated at the following values: BracingHours = 3.625.	


Pairwise Comparisons	
Measure: MEASURE_1	
(I) factor1	(J) factor1	Mean Difference (I-J)	Std. Error	Sig.b	95% Confidence Interval for Differenceb	
					Lower Bound	Upper Bound	
1	2	1.791	.731	.114	-.246	3.828	
	3	2.461*	.758	.015	.348	4.574	
	4	2.449*	.668	.005	.587	4.310	
2	1	-1.791	.731	.114	-3.828	.246	
	3	.670	.549	1.000	-.860	2.200	
	4	.658	.573	1.000	-.940	2.255	
3	1	-2.461*	.758	.015	-4.574	-.348	
	2	-.670	.549	1.000	-2.200	.860	
	4	-.013	.319	1.000	-.901	.876	
4	1	-2.449*	.668	.005	-4.310	-.587	
	2	-.658	.573	1.000	-2.255	.940	
	3	.013	.319	1.000	-.876	.901	

Based on estimated marginal means	
*. The mean difference is significant at the .05 level.	
b. Adjustment for multiple comparisons: Bonferroni.	


Multivariate Tests	
	Value	F	Hypothesis df	Error df	Sig.	Partial Eta Squared	
Pillai's trace	.267	4.254a	3.000	35.000	.012	.267	
Wilks' lambda	.733	4.254a	3.000	35.000	.012	.267	
Hotelling's trace	.365	4.254a	3.000	35.000	.012	.267	
Roy's largest root	.365	4.254a	3.000	35.000	.012	.267	

Multivariate Tests	
	Noncent. Parameter	Observed Power	
Pillai's trace	12.762	.819a	
Wilks' lambda	12.762	.819a	
Hotelling's trace	12.762	.819a	
Roy's largest root	12.762	.819a	

Each F tests the multivariate effect of factor1. These tests are based on the linearly independent pairwise comparisons among the estimated marginal means.	
a. Exact statistic	
b. Computed using alpha = .05	


4. Group No. * factor1	
Measure: MEASURE_1	
Group No.	factor1	Mean	Std. Error	95% Confidence Interval	
				Lower Bound	Upper Bound	
Group A	1	27.881a	1.429	24.987	30.776	
	2	26.500a	1.391	23.681	29.320	
	3	26.531a	1.339	23.819	29.244	
	4	26.919a	1.248	24.390	29.448	
Group B	1	27.534a	1.580	24.332	30.736	
	2	25.333a	1.539	22.214	28.451	
	3	23.962a	1.481	20.961	26.963	
	4	23.599a	1.381	20.802	26.397	

a. Covariates appearing in the model are evaluated at the following values: BracingHours = 3.625.	


ERROR : (16302) No error.


GLM ThoracicInitialCobb Thoracic1yrCobb Thoracic1_5Cobb Thoracic2Cobb BY GroupNo
  /WSFACTOR=Time4 4 Polynomial
  /METHOD=SSTYPE(3)
  /POSTHOC=GroupNo(BONFERRONI)
  /EMMEANS=TABLES(GroupNo) COMPARE ADJ(BONFERRONI)
  /EMMEANS=TABLES(Time4) COMPARE ADJ(BONFERRONI)
  /EMMEANS=TABLES(GroupNo*Time4)
  /EMMEANS=TABLES(OVERALL)
  /PRINT=DESCRIPTIVE ETASQ OPOWER HOMOGENEITY
  /CRITERIA=ALPHA(.05)
  /WSDESIGN=Time4
  /DESIGN=GroupNo.


General Linear Model


Notes	
Output Created	28-NOV-2020 09:37:45	
Comments		
Input	Data	D:\curvepattern191205.sav	
	Active Dataset	DataSet1	
	Filter	<none>	
	Weight	<none>	
	Split File	<none>	
	N of Rows in Working Data File	40	
Missing Value Handling	Definition of Missing	User-defined missing values are treated as missing.	
	Cases Used	Statistics are based on all cases with valid data for all variables in the model.	
Syntax	GLM ThoracicInitialCobb Thoracic1yrCobb Thoracic1_5Cobb Thoracic2Cobb BY GroupNo
  /WSFACTOR=Time4 4 Polynomial
  /METHOD=SSTYPE(3)
  /POSTHOC=GroupNo(BONFERRONI)
  /EMMEANS=TABLES(GroupNo) COMPARE ADJ(BONFERRONI)
  /EMMEANS=TABLES(Time4) COMPARE ADJ(BONFERRONI)
  /EMMEANS=TABLES(GroupNo*Time4)
  /EMMEANS=TABLES(OVERALL)
  /PRINT=DESCRIPTIVE ETASQ OPOWER HOMOGENEITY
  /CRITERIA=ALPHA(.05)
  /WSDESIGN=Time4
  /DESIGN=GroupNo.	

Notes	
Resources	Processor Time	00:00:00.11	
	Elapsed Time	00:00:00.09	


[DataSet1] D:\curvepattern191205.sav


Warnings	
Box's Test of Equality of Covariance Matrices is not computed because there are less than two nonempty cells.	
Levene's Test of Equality of Error Variances is not computed because there are less than two nonempty groups.	
Post hoc tests are not performed for Group No. because there are fewer than three groups.	


Within-Subjects Factors	
Measure: MEASURE_1	
Time4	Dependent Variable	
1	ThoracicInitialCobb	
2	Thoracic1yrCobb	
3	Thoracic1_5Cobb	
4	Thoracic2Cobb	


Between-Subjects Factors	
	Value Label	N	
Group No.	1	Group A	22	


Descriptive Statistics	
	Group No.	Mean	Std. Deviation	N	
Group A Cobb: initial	Group A	28.500	8.4219	22	
	Total	28.500	8.4219	22	
Group A Cobb: one year after	Group A	27.000	7.2045	22	
	Total	27.000	7.2045	22	
Group A Cobb: one and a half years after	Group A	27.091	7.6589	22	
	Total	27.091	7.6589	22	
Group A Cobb: two years after	Group A	27.455	6.9606	22	
	Total	27.455	6.9606	22	


Multivariate Testsa	
Effect	Value	F	Hypothesis df	Error df	Sig.	
Time4	Pillai's Trace	.104	.731b	3.000	19.000	.546	
	Wilks' Lambda	.896	.731b	3.000	19.000	.546	
	Hotelling's Trace	.115	.731b	3.000	19.000	.546	
	Roy's Largest Root	.115	.731b	3.000	19.000	.546	
Time4 * GroupNo	Pillai's Trace	.000	.b	.000	.000	.	
	Wilks' Lambda	1.000	.b	.000	20.000	.	
	Hotelling's Trace	.000	.b	.000	2.000	.	
	Roy's Largest Root	.000	.000b	3.000	18.000	1.000	

Multivariate Testsa	
Effect	Partial Eta Squared	Noncent. Parameter	Observed Power	
Time4	Pillai's Trace	.104	2.194b	.176	
	Wilks' Lambda	.104	2.194b	.176	
	Hotelling's Trace	.104	2.194b	.176	
	Roy's Largest Root	.104	2.194b	.176	
Time4 * GroupNo	Pillai's Trace	.	.b	.	
	Wilks' Lambda	.	.b	.	
	Hotelling's Trace	.	.b	.	
	Roy's Largest Root	.000	.000b	.050	

a. Design: Intercept + GroupNo 
 Within Subjects Design: Time4	
b. Exact statistic	
c. Computed using alpha = .05	


Mauchly's Test of Sphericitya	
Measure: MEASURE_1	
Within Subjects Effect	Mauchly's W	Approx. Chi-Square	df	Sig.	Epsilonb	
					Greenhouse-Geisser	
Time4	.240	28.167	5	.000	.586	

Mauchly's Test of Sphericitya	
Measure: MEASURE_1	
Within Subjects Effect	Epsilon	
	Huynh-Feldt	Lower-bound	
Time4	.635	.333	

Tests the null hypothesis that the error covariance matrix of the orthonormalized transformed dependent variables is proportional to an identity matrix.a	
a. Design: Intercept + GroupNo 
 Within Subjects Design: Time4	
b. May be used to adjust the degrees of freedom for the averaged tests of significance. Corrected tests are displayed in the Tests of Within-Subjects Effects table.	


Tests of Within-Subjects Effects	
Measure: MEASURE_1	
Source	Type III Sum of Squares	df	Mean Square	F	
Time4	Sphericity Assumed	31.216	3	10.405	1.581	
	Greenhouse-Geisser	31.216	1.757	17.764	1.581	
	Huynh-Feldt	31.216	1.905	16.385	1.581	
	Lower-bound	31.216	1.000	31.216	1.581	
Time4 * GroupNo	Sphericity Assumed	.000	0	.	.	
	Greenhouse-Geisser	.000	.000	.	.	
	Huynh-Feldt	.000	.000	.	.	
	Lower-bound	.000	.000	.	.	
Error(Time4)	Sphericity Assumed	414.534	63	6.580		
	Greenhouse-Geisser	414.534	36.903	11.233		
	Huynh-Feldt	414.534	40.008	10.361		
	Lower-bound	414.534	21.000	19.740		

Tests of Within-Subjects Effects	
Measure: MEASURE_1	
Source	Sig.	Partial Eta Squared	Noncent. Parameter	Observed Power	
Time4	Sphericity Assumed	.203	.070	4.744	.397	
	Greenhouse-Geisser	.221	.070	2.779	.295	
	Huynh-Feldt	.219	.070	3.013	.308	
	Lower-bound	.222	.070	1.581	.225	
Time4 * GroupNo	Sphericity Assumed	.	.000	.000	.	
	Greenhouse-Geisser	.	.000	.000	.	
	Huynh-Feldt	.	.000	.000	.	
	Lower-bound	.	.000	.000	.	
Error(Time4)	Sphericity Assumed					
	Greenhouse-Geisser					
	Huynh-Feldt					
	Lower-bound					

a. Computed using alpha = .05	


Tests of Within-Subjects Contrasts	
Measure: MEASURE_1	
Source	Time4	Type III Sum of Squares	df	Mean Square	F	Sig.	
Time4	Linear	10.202	1	10.202	1.142	.297	
	Quadratic	19.102	1	19.102	2.386	.137	
	Cubic	1.911	1	1.911	.683	.418	
Time4 * GroupNo	Linear	.000	0	.	.	.	
	Quadratic	.000	0	.	.	.	
	Cubic	.000	0	.	.	.	
Error(Time4)	Linear	187.648	21	8.936			
	Quadratic	168.148	21	8.007			
	Cubic	58.739	21	2.797			

Tests of Within-Subjects Contrasts	
Measure: MEASURE_1	
Source	Time4	Partial Eta Squared	Noncent. Parameter	Observed Power	
Time4	Linear	.052	1.142	.175	
	Quadratic	.102	2.386	.314	
	Cubic	.032	.683	.124	
Time4 * GroupNo	Linear	.000	.000	.	
	Quadratic	.000	.000	.	
	Cubic	.000	.000	.	
Error(Time4)	Linear				
	Quadratic				
	Cubic				

a. Computed using alpha = .05	


Tests of Between-Subjects Effects	
Measure: MEASURE_1 
 Transformed Variable: Average	
Source	Type III Sum of Squares	df	Mean Square	F	Sig.	Partial Eta Squared	
Intercept	66605.011	1	66605.011	316.862	.000	.938	
GroupNo	.000	0	.	.	.	.000	
Error	4414.239	21	210.202				

Tests of Between-Subjects Effects	
Measure: MEASURE_1 
 Transformed Variable: Average	
Source	Noncent. Parameter	Observed Power	
Intercept	316.862	1.000	
GroupNo	.000	.	
Error			

a. Computed using alpha = .05	


Estimated Marginal Means


1. Group No.


Estimates	
Measure: MEASURE_1	
Group No.	Mean	Std. Error	95% Confidence Interval	
			Lower Bound	Upper Bound	
Group A	27.511	1.546	24.297	30.725	


Pairwise Comparisonsa	
	

a. This pairwise comparison table cannot be constructed because Group No., the factor being compared, has one level.	


Univariate Tests	
Measure: MEASURE_1	
	Sum of Squares	df	Mean Square	F	Sig.	Partial Eta Squared	
Contrast	.000	0	.	.	.	.000	
Error	1103.560	21	52.550				

Univariate Tests	
Measure: MEASURE_1	
	Noncent. Parameter	Observed Power	
Contrast	.000	.	
Error			

The F tests the effect of Group No.. This test is based on the linearly independent pairwise comparisons among the estimated marginal means.	
a. Computed using alpha = .05	


2. Time4


Estimates	
Measure: MEASURE_1	
Time4	Mean	Std. Error	95% Confidence Interval	
			Lower Bound	Upper Bound	
1	28.500	1.796	24.766	32.234	
2	27.000	1.536	23.806	30.194	
3	27.091	1.633	23.695	30.487	
4	27.455	1.484	24.368	30.541	


Pairwise Comparisons	
Measure: MEASURE_1	
(I) Time4	(J) Time4	Mean Difference (I-J)	Std. Error	Sig.a	95% Confidence Interval for Differencea	
					Lower Bound	Upper Bound	
1	2	1.500	1.008	.910	-1.436	4.436	
	3	1.409	.993	1.000	-1.482	4.300	
	4	1.045	.891	1.000	-1.550	3.641	
2	1	-1.500	1.008	.910	-4.436	1.436	
	3	-.091	.522	1.000	-1.611	1.429	
	4	-.455	.627	1.000	-2.280	1.370	
3	1	-1.409	.993	1.000	-4.300	1.482	
	2	.091	.522	1.000	-1.429	1.611	
	4	-.364	.358	1.000	-1.405	.678	
4	1	-1.045	.891	1.000	-3.641	1.550	
	2	.455	.627	1.000	-1.370	2.280	
	3	.364	.358	1.000	-.678	1.405	

Based on estimated marginal means	
a. Adjustment for multiple comparisons: Bonferroni.	


Multivariate Tests	
	Value	F	Hypothesis df	Error df	Sig.	Partial Eta Squared	
Pillai's trace	.104	.731a	3.000	19.000	.546	.104	
Wilks' lambda	.896	.731a	3.000	19.000	.546	.104	
Hotelling's trace	.115	.731a	3.000	19.000	.546	.104	
Roy's largest root	.115	.731a	3.000	19.000	.546	.104	

Multivariate Tests	
	Noncent. Parameter	Observed Power	
Pillai's trace	2.194	.176a	
Wilks' lambda	2.194	.176a	
Hotelling's trace	2.194	.176a	
Roy's largest root	2.194	.176a	

Each F tests the multivariate effect of Time4. These tests are based on the linearly independent pairwise comparisons among the estimated marginal means.	
a. Exact statistic	
b. Computed using alpha = .05	


3. Group No. * Time4	
Measure: MEASURE_1	
Group No.	Time4	Mean	Std. Error	95% Confidence Interval	
				Lower Bound	Upper Bound	
Group A	1	28.500	1.796	24.766	32.234	
	2	27.000	1.536	23.806	30.194	
	3	27.091	1.633	23.695	30.487	
	4	27.455	1.484	24.368	30.541	


4. Grand Mean	
Measure: MEASURE_1	
Mean	Std. Error	95% Confidence Interval	
		Lower Bound	Upper Bound	
27.511	1.546	24.297	30.725	


ERROR : (16302) No error.


GLM LumbarInitialCobb Lumbar1yrCobb Lumbar1_5Cobb Lumbar2yrCobb BY GroupNo
  /WSFACTOR=Time4 4 Polynomial
  /METHOD=SSTYPE(3)
  /POSTHOC=GroupNo(BONFERRONI)
  /EMMEANS=TABLES(GroupNo) COMPARE ADJ(BONFERRONI)
  /EMMEANS=TABLES(Time4) COMPARE ADJ(BONFERRONI)
  /EMMEANS=TABLES(GroupNo*Time4)
  /EMMEANS=TABLES(OVERALL)
  /PRINT=DESCRIPTIVE ETASQ OPOWER HOMOGENEITY
  /CRITERIA=ALPHA(.05)
  /WSDESIGN=Time4
  /DESIGN=GroupNo.


General Linear Model


Notes	
Output Created	28-NOV-2020 09:38:53	
Comments		
Input	Data	D:\curvepattern191205.sav	
	Active Dataset	DataSet1	
	Filter	<none>	
	Weight	<none>	
	Split File	<none>	
	N of Rows in Working Data File	40	
Missing Value Handling	Definition of Missing	User-defined missing values are treated as missing.	
	Cases Used	Statistics are based on all cases with valid data for all variables in the model.	
Syntax	GLM LumbarInitialCobb Lumbar1yrCobb Lumbar1_5Cobb Lumbar2yrCobb BY GroupNo
  /WSFACTOR=Time4 4 Polynomial
  /METHOD=SSTYPE(3)
  /POSTHOC=GroupNo(BONFERRONI)
  /EMMEANS=TABLES(GroupNo) COMPARE ADJ(BONFERRONI)
  /EMMEANS=TABLES(Time4) COMPARE ADJ(BONFERRONI)
  /EMMEANS=TABLES(GroupNo*Time4)
  /EMMEANS=TABLES(OVERALL)
  /PRINT=DESCRIPTIVE ETASQ OPOWER HOMOGENEITY
  /CRITERIA=ALPHA(.05)
  /WSDESIGN=Time4
  /DESIGN=GroupNo.	

Notes	
Resources	Processor Time	00:00:00.13	
	Elapsed Time	00:00:00.11	


[DataSet1] D:\curvepattern191205.sav


Warnings	
Box's Test of Equality of Covariance Matrices is not computed because there are less than two nonempty cells.	
Levene's Test of Equality of Error Variances is not computed because there are less than two nonempty groups.	
Post hoc tests are not performed for Group No. because there are fewer than three groups.	


Within-Subjects Factors	
Measure: MEASURE_1	
Time4	Dependent Variable	
1	LumbarInitialCobb	
2	Lumbar1yrCobb	
3	Lumbar1_5Cobb	
4	Lumbar2yrCobb	


Between-Subjects Factors	
	Value Label	N	
Group No.	2	Group B	18	


Descriptive Statistics	
	Group No.	Mean	Std. Deviation	N	
Group B Cobb: initial	Group B	26.778	10.1086	18	
	Total	26.778	10.1086	18	
Group B Cobb: one year after	Group B	24.722	9.4047	18	
	Total	24.722	9.4047	18	
Group B Cobb: one and a half years after	Group B	23.278	9.4172	18	
	Total	23.278	9.4172	18	
Group B Cobb: two years after	Group B	22.944	9.1553	18	
	Total	22.944	9.1553	18	


Multivariate Testsa	
Effect	Value	F	Hypothesis df	Error df	Sig.	
Time4	Pillai's Trace	.462	4.301b	3.000	15.000	.022	
	Wilks' Lambda	.538	4.301b	3.000	15.000	.022	
	Hotelling's Trace	.860	4.301b	3.000	15.000	.022	
	Roy's Largest Root	.860	4.301b	3.000	15.000	.022	
Time4 * GroupNo	Pillai's Trace	.000	.b	.000	.000	.	
	Wilks' Lambda	1.000	.b	.000	16.000	.	
	Hotelling's Trace	.000	.b	.000	2.000	.	
	Roy's Largest Root	.000	.000b	3.000	14.000	1.000	

Multivariate Testsa	
Effect	Partial Eta Squared	Noncent. Parameter	Observed Power	
Time4	Pillai's Trace	.462	12.902b	.758	
	Wilks' Lambda	.462	12.902b	.758	
	Hotelling's Trace	.462	12.902b	.758	
	Roy's Largest Root	.462	12.902b	.758	
Time4 * GroupNo	Pillai's Trace	.	.b	.	
	Wilks' Lambda	.	.b	.	
	Hotelling's Trace	.	.b	.	
	Roy's Largest Root	.000	.000b	.050	

a. Design: Intercept + GroupNo 
 Within Subjects Design: Time4	
b. Exact statistic	
c. Computed using alpha = .05	


Mauchly's Test of Sphericitya	
Measure: MEASURE_1	
Within Subjects Effect	Mauchly's W	Approx. Chi-Square	df	Sig.	Epsilonb	
					Greenhouse-Geisser	
Time4	.513	10.503	5	.063	.784	

Mauchly's Test of Sphericitya	
Measure: MEASURE_1	
Within Subjects Effect	Epsilon	
	Huynh-Feldt	Lower-bound	
Time4	.918	.333	

Tests the null hypothesis that the error covariance matrix of the orthonormalized transformed dependent variables is proportional to an identity matrix.a	
a. Design: Intercept + GroupNo 
 Within Subjects Design: Time4	
b. May be used to adjust the degrees of freedom for the averaged tests of significance. Corrected tests are displayed in the Tests of Within-Subjects Effects table.	


Tests of Within-Subjects Effects	
Measure: MEASURE_1	
Source	Type III Sum of Squares	df	Mean Square	F	
Time4	Sphericity Assumed	164.375	3	54.792	6.163	
	Greenhouse-Geisser	164.375	2.352	69.882	6.163	
	Huynh-Feldt	164.375	2.754	59.687	6.163	
	Lower-bound	164.375	1.000	164.375	6.163	
Time4 * GroupNo	Sphericity Assumed	.000	0	.	.	
	Greenhouse-Geisser	.000	.000	.	.	
	Huynh-Feldt	.000	.000	.	.	
	Lower-bound	.000	.000	.	.	
Error(Time4)	Sphericity Assumed	453.375	51	8.890		
	Greenhouse-Geisser	453.375	39.987	11.338		
	Huynh-Feldt	453.375	46.817	9.684		
	Lower-bound	453.375	17.000	26.669		

Tests of Within-Subjects Effects	
Measure: MEASURE_1	
Source	Sig.	Partial Eta Squared	Noncent. Parameter	Observed Power	
Time4	Sphericity Assumed	.001	.266	18.490	.949	
	Greenhouse-Geisser	.003	.266	14.498	.902	
	Huynh-Feldt	.002	.266	16.974	.935	
	Lower-bound	.024	.266	6.163	.648	
Time4 * GroupNo	Sphericity Assumed	.	.000	.000	.	
	Greenhouse-Geisser	.	.000	.000	.	
	Huynh-Feldt	.	.000	.000	.	
	Lower-bound	.	.000	.000	.	
Error(Time4)	Sphericity Assumed					
	Greenhouse-Geisser					
	Huynh-Feldt					
	Lower-bound					

a. Computed using alpha = .05	


Tests of Within-Subjects Contrasts	
Measure: MEASURE_1	
Source	Time4	Type III Sum of Squares	df	Mean Square	F	Sig.	
Time4	Linear	150.803	1	150.803	13.184	.002	
	Quadratic	13.347	1	13.347	1.733	.205	
	Cubic	.225	1	.225	.030	.865	
Time4 * GroupNo	Linear	.000	0	.	.	.	
	Quadratic	.000	0	.	.	.	
	Cubic	.000	0	.	.	.	
Error(Time4)	Linear	194.447	17	11.438			
	Quadratic	130.903	17	7.700			
	Cubic	128.025	17	7.531			

Tests of Within-Subjects Contrasts	
Measure: MEASURE_1	
Source	Time4	Partial Eta Squared	Noncent. Parameter	Observed Power	
Time4	Linear	.437	13.184	.928	
	Quadratic	.093	1.733	.237	
	Cubic	.002	.030	.053	
Time4 * GroupNo	Linear	.000	.000	.	
	Quadratic	.000	.000	.	
	Cubic	.000	.000	.	
Error(Time4)	Linear				
	Quadratic				
	Cubic				

a. Computed using alpha = .05	


Tests of Between-Subjects Effects	
Measure: MEASURE_1 
 Transformed Variable: Average	
Source	Type III Sum of Squares	df	Mean Square	F	Sig.	Partial Eta Squared	
Intercept	42973.347	1	42973.347	127.720	.000	.883	
GroupNo	.000	0	.	.	.	.000	
Error	5719.903	17	336.465				

Tests of Between-Subjects Effects	
Measure: MEASURE_1 
 Transformed Variable: Average	
Source	Noncent. Parameter	Observed Power	
Intercept	127.720	1.000	
GroupNo	.000	.	
Error			

a. Computed using alpha = .05	


Estimated Marginal Means


1. Group No.


Estimates	
Measure: MEASURE_1	
Group No.	Mean	Std. Error	95% Confidence Interval	
			Lower Bound	Upper Bound	
Group B	24.431	2.162	19.870	28.991	


Pairwise Comparisonsa	
	

a. This pairwise comparison table cannot be constructed because Group No., the factor being compared, has one level.	


Univariate Tests	
Measure: MEASURE_1	
	Sum of Squares	df	Mean Square	F	Sig.	Partial Eta Squared	
Contrast	.000	0	.	.	.	.000	
Error	1429.976	17	84.116				

Univariate Tests	
Measure: MEASURE_1	
	Noncent. Parameter	Observed Power	
Contrast	.000	.	
Error			

The F tests the effect of Group No.. This test is based on the linearly independent pairwise comparisons among the estimated marginal means.	
a. Computed using alpha = .05	


2. Time4


Estimates	
Measure: MEASURE_1	
Time4	Mean	Std. Error	95% Confidence Interval	
			Lower Bound	Upper Bound	
1	26.778	2.383	21.751	31.805	
2	24.722	2.217	20.045	29.399	
3	23.278	2.220	18.595	27.961	
4	22.944	2.158	18.392	27.497	


Pairwise Comparisons	
Measure: MEASURE_1	
(I) Time4	(J) Time4	Mean Difference (I-J)	Std. Error	Sig.b	95% Confidence Interval for Differenceb	
					Lower Bound	Upper Bound	
1	2	2.056	1.101	.476	-1.230	5.341	
	3	3.500*	1.144	.043	.086	6.914	
	4	3.833*	1.014	.009	.808	6.859	
2	1	-2.056	1.101	.476	-5.341	1.230	
	3	1.444	1.039	1.000	-1.656	4.545	
	4	1.778	.999	.557	-1.202	4.757	
3	1	-3.500*	1.144	.043	-6.914	-.086	
	2	-1.444	1.039	1.000	-4.545	1.656	
	4	.333	.548	1.000	-1.303	1.970	
4	1	-3.833*	1.014	.009	-6.859	-.808	
	2	-1.778	.999	.557	-4.757	1.202	
	3	-.333	.548	1.000	-1.970	1.303	

Based on estimated marginal means	
*. The mean difference is significant at the .05 level.	
b. Adjustment for multiple comparisons: Bonferroni.	


Multivariate Tests	
	Value	F	Hypothesis df	Error df	Sig.	Partial Eta Squared	
Pillai's trace	.462	4.301a	3.000	15.000	.022	.462	
Wilks' lambda	.538	4.301a	3.000	15.000	.022	.462	
Hotelling's trace	.860	4.301a	3.000	15.000	.022	.462	
Roy's largest root	.860	4.301a	3.000	15.000	.022	.462	

Multivariate Tests	
	Noncent. Parameter	Observed Power	
Pillai's trace	12.902	.758a	
Wilks' lambda	12.902	.758a	
Hotelling's trace	12.902	.758a	
Roy's largest root	12.902	.758a	

Each F tests the multivariate effect of Time4. These tests are based on the linearly independent pairwise comparisons among the estimated marginal means.	
a. Exact statistic	
b. Computed using alpha = .05	


3. Group No. * Time4	
Measure: MEASURE_1	
Group No.	Time4	Mean	Std. Error	95% Confidence Interval	
				Lower Bound	Upper Bound	
Group B	1	26.778	2.383	21.751	31.805	
	2	24.722	2.217	20.045	29.399	
	3	23.278	2.220	18.595	27.961	
	4	22.944	2.158	18.392	27.497	


4. Grand Mean	
Measure: MEASURE_1	
Mean	Std. Error	95% Confidence Interval	
		Lower Bound	Upper Bound	
24.431	2.162	19.870	28.991	

LOGISTIC REGRESSION VARIABLES TwoYrProgressionCode
  /METHOD=ENTER GroupNo Brace
  /CONTRAST (GroupNo)=Indicator
  /CONTRAST (Brace)=Indicator
  /PRINT=GOODFIT SUMMARY CI(95)
  /CRITERIA=PIN(0.05) POUT(0.10) ITERATE(20) CUT(0.5).


Logistic Regression


Notes	
Output Created	28-NOV-2020 09:53:00	
Comments		
Input	Data	D:\curvepattern191205.sav	
	Active Dataset	DataSet1	
	Filter	<none>	
	Weight	<none>	
	Split File	<none>	
	N of Rows in Working Data File	40	
Missing Value Handling	Definition of Missing	User-defined missing values are treated as missing	
Syntax	LOGISTIC REGRESSION VARIABLES TwoYrProgressionCode
  /METHOD=ENTER GroupNo Brace
  /CONTRAST (GroupNo)=Indicator
  /CONTRAST (Brace)=Indicator
  /PRINT=GOODFIT SUMMARY CI(95)
  /CRITERIA=PIN(0.05) POUT(0.10) ITERATE(20) CUT(0.5).	
Resources	Processor Time	00:00:00.05	
	Elapsed Time	00:00:00.05	


[DataSet1] D:\curvepattern191205.sav


Case Processing Summary	
Unweighted Casesa	N	Percent	
Selected Cases	Included in Analysis	40	100.0	
	Missing Cases	0	.0	
	Total	40	100.0	
Unselected Cases	0	.0	
Total	40	100.0	

a. If weight is in effect, see classification table for the total number of cases.	


Dependent Variable Encoding	
Original Value	Internal Value	
stable	0	
Regress	1	


Categorical Variables Codings	
	Frequency	Parameter coding	
		(1)	
Braced or not	No brace	30	1.000	
	Braced	10	.000	
Group No.	Group A	22	1.000	
	Group B	18	.000	


Block 0: Beginning Block


Classification Tablea,b	
	Observed	Predicted	
		Progression after two years of PSSE	Percentage Correct	
		stable	Regress		
Step 0	Progression after two years of PSSE	stable	32	0	100.0	
		Regress	8	0	.0	
	Overall Percentage			80.0	

a. Constant is included in the model.	
b. The cut value is .500	


Variables in the Equation	
	B	S.E.	Wald	df	Sig.	Exp(B)	
Step 0	Constant	-1.386	.395	12.300	1	.000	.250	


Variables not in the Equation	
	Score	df	Sig.	
Step 0	Variables	GroupNo(1)	3.636	1	.057	
		Brace(1)	.000	1	1.000	
	Overall Statistics	3.750	2	.153	


Block 1: Method = Enter


Omnibus Tests of Model Coefficients	
	Chi-square	df	Sig.	
Step 1	Step	3.849	2	.146	
	Block	3.849	2	.146	
	Model	3.849	2	.146	


Model Summary	
Step	-2 Log likelihood	Cox & Snell R Square	Nagelkerke R Square	
1	36.183a	.092	.145	

a. Estimation terminated at iteration number 5 because parameter estimates changed by less than .001.	


Hosmer and Lemeshow Test	
Step	Chi-square	df	Sig.	
1	.180	2	.914	


Contingency Table for Hosmer and Lemeshow Test	
	Progression after two years of PSSE = stable	Progression after two years of PSSE = Regress	Total	
	Observed	Expected	Observed	Expected		
Step 1	1	14	13.788	1	1.212	15	
	2	6	6.212	1	.788	7	
	3	10	10.212	5	4.788	15	
	4	2	1.788	1	1.212	3	


Classification Tablea	
	Observed	Predicted	
		Progression after two years of PSSE	Percentage Correct	
		stable	Regress		
Step 1	Progression after two years of PSSE	stable	32	0	100.0	
		Regress	8	0	.0	
	Overall Percentage			80.0	

a. The cut value is .500	


Variables in the Equation	
	B	S.E.	Wald	df	Sig.	Exp(B)	95% C.I.for EXP(B)	
							Lower	
Step 1a	GroupNo(1)	-1.675	.918	3.325	1	.068	.187	.031	
	Brace(1)	-.368	.990	.138	1	.710	.692	.100	
	Constant	-.389	.953	.167	1	.683	.677		

Variables in the Equation	
	95% C.I.for EXP(B)	
	Upper	
Step 1a	GroupNo(1)	1.134	
	Brace(1)	4.816	
	Constant		

a. Variable(s) entered on step 1: GroupNo, Brace.	

CROSSTABS
  /TABLES=TwoYrProgressionCode BY GroupNo
  /FORMAT=AVALUE TABLES
  /STATISTICS=CHISQ
  /CELLS=COUNT COLUMN
  /COUNT ROUND CELL.


Crosstabs


Notes	
Output Created	28-NOV-2020 09:58:07	
Comments		
Input	Data	D:\curvepattern191205.sav	
	Active Dataset	DataSet1	
	Filter	<none>	
	Weight	<none>	
	Split File	<none>	
	N of Rows in Working Data File	40	
Missing Value Handling	Definition of Missing	User-defined missing values are treated as missing.	
	Cases Used	Statistics for each table are based on all the cases with valid data in the specified range(s) for all variables in each table.	
Syntax	CROSSTABS
  /TABLES=TwoYrProgressionCode BY GroupNo
  /FORMAT=AVALUE TABLES
  /STATISTICS=CHISQ
  /CELLS=COUNT COLUMN
  /COUNT ROUND CELL.	
Resources	Processor Time	00:00:00.02	
	Elapsed Time	00:00:00.02	
	Dimensions Requested	2	
	Cells Available	174762	


[DataSet1] D:\curvepattern191205.sav


Case Processing Summary	
	Cases	
	Valid	Missing	Total	
	N	Percent	N	Percent	N	Percent	
Progression after two years of PSSE * Group No.	40	100.0%	0	0.0%	40	100.0%	


Progression after two years of PSSE * Group No. Crosstabulation	
	Group No.	Total	
	Group A	Group B		
Progression after two years of PSSE	stable	Count	20	12	32	
		% within Group No.	90.9%	66.7%	80.0%	
	Regress	Count	2	6	8	
		% within Group No.	9.1%	33.3%	20.0%	
Total	Count	22	18	40	
	% within Group No.	100.0%	100.0%	100.0%	


Chi-Square Tests	
	Value	df	Asymp. Sig. (2-sided)	Exact Sig. (2-sided)	Exact Sig. (1-sided)	
Pearson Chi-Square	3.636a	1	.057			
Continuity Correctionb	2.279	1	.131			
Likelihood Ratio	3.714	1	.054			
Fisher's Exact Test				.110	.065	
Linear-by-Linear Association	3.545	1	.060			
N of Valid Cases	40					

a. 2 cells (50.0%) have expected count less than 5. The minimum expected count is 3.60.	
b. Computed only for a 2x2 table	
