# Supplementary material for: Does curve pattern impact on the effects of physiotherapeutic scoliosis specific exercises on Cobb angles of participants with adolescent idiopathic scoliosis: A prospective clinical trial with two years follow-up
Source: PLoS One. 2021 Jan 25;16(1):e0245829. doi: 10.1371/journal.pone.0245829 (PMC7833215; doi:10.1371/journal.pone.0245829)
Supplement: S5 File — (PDF) [file pone.0245829.s005.pdf]

TREND Statement Checklist

Paper Section/ Topic	Item No	Descriptor	Reported? ✓	Pg #
**Title and Abstract**				
Title and Abstract	1	• Information on how unit were allocated to interventions	Yes	Pg2
		• Structured abstract recommended	Yes	Pg2-4
		• Information on target population or study sample	Yes	Pg3
**Introduction**				
Background	2	• Scientific background and explanation of rationale	Yes	Pg4-7
		• Theories used in designing behavioral interventions	Yes	Pg4-7
**Methods**				
Participants	3	• Eligibility criteria for participants, including criteria at different levels in recruitment/sampling plan (e.g., cities, clinics, subjects)	Yes	Pg7-8
		• Method of recruitment (e.g., referral, self-selection), including the sampling method if a systematic sampling plan was implemented	Yes	Pg7–9
		• Recruitment setting	Yes	Pg8
		• Settings and locations where the data were collected	Yes	Pg7
Interventions	4	• Details of the interventions intended for each study condition and how and when they were actually administered, specifically including:	Yes	Pg9-11
		○ Content: what was given?	Yes	Pg9-11
		○ Delivery method: how was the content given?	Yes	Pg9,11-12
		○ Unit of delivery: how were the subjects grouped during delivery?	Yes	Pg9
		○ Deliverer: who delivered the intervention?	Yes	Pg9
		○ Setting: where was the intervention delivered?	Yes	Pg10
		○ Exposure quantity and duration: how many sessions or episodes or events were intended to be delivered? How long were they intended to last?	Yes	Pg9
		○ Time span: how long was it intended to take to deliver the intervention to each unit?	Yes	Pg11
		○ Activities to increase compliance or adherence (e.g., incentives)	Yes	Pg11
Objectives	5	• Specific objectives and hypotheses	Yes	Pg7
Outcomes	6	• Clearly defined primary and secondary outcome measures	Yes	Pg13-14
		• Methods used to collect data and any methods used to enhance the quality of measurements	Yes	Pg13-14
		• Information on validated instruments such as psychometric and biometric properties	Yes	Pg13
Sample Size	7	• How sample size was determined and, when applicable, explanation of any interim analyses and stopping rules	Yes	Pg14
Assignment Method	8	• Unit of assignment (the unit being assigned to study condition, e.g., individual, group, community)	Yes	Pg14-15
		• Method used to assign units to study conditions, including details of any restriction (e.g., blocking, stratification, minimization)	Yes	Pg15
		• Inclusion of aspects employed to help minimize potential bias induced due to non-randomization (e.g., matching)	Yes	Pg15

TREND Statement Checklist

Blinding (masking)	9	• Whether or not participants, those administering the interventions, and those assessing the outcomes were blinded to study condition assignment; if so, statement regarding how the blinding was accomplished and how it was assessed.	Yes	Pg14,31
Unit of Analysis	10	• Description of the smallest unit that is being analyzed to assess intervention effects (e.g., individual, group, or community)	Yes	Pg14
		• If the unit of analysis differs from the unit of assignment, the analytical method used to account for this (e.g., adjusting the standard error estimates by the design effect or using multilevel analysis)	Yes	Pg12-14
Statistical Methods	11	• Statistical methods used to compare study groups for primary methods outcome(s), including complex methods of correlated data	Yes	Pg14,15
		• Statistical methods used for additional analyses, such as a subgroup analyses and adjusted analysis	Yes	Pg15
		• Methods for imputing missing data, if used	NA	NA
		• Statistical software or programs used	Yes	Pg15
**Results**				
Participant flow	12	• Flow of participants through each stage of the study: enrollment, assignment, allocation, and intervention exposure, follow-up, analysis (a diagram is strongly recommended)	Yes	Pg9
		○ Enrollment: the numbers of participants screened for eligibility, found to be eligible or not eligible, declined to be enrolled, and enrolled in the study	Yes	Pg9
		○ Assignment: the numbers of participants assigned to a study condition	Yes	Pg9
		○ Allocation and intervention exposure: the number of participants assigned to each study condition and the number of participants who received each intervention	Yes	Pg9
		○ Follow-up: the number of participants who completed the follow-up or did not complete the follow-up (i.e., lost to follow-up), by study condition	Yes	Pg9
		○ Analysis: the number of participants included in or excluded from the main analysis, by study condition	Yes	Pg9
		• Description of protocol deviations from study as planned, along with reasons	Yes	Pg14-15
Recruitment	13	• Dates defining the periods of recruitment and follow-up	Yes	Pg9
Baseline Data	14	• Baseline demographic and clinical characteristics of participants in each study condition	Yes	Pg15-16
		• Baseline characteristics for each study condition relevant to specific disease prevention research	Yes	Pg15-16
		• Baseline comparisons of those lost to follow-up and those retained, overall and by study condition	NA	Pg16-18
		• Comparison between study population at baseline and target population of interest	Yes	Pg15-18
Baseline equivalence	15	• Data on study group equivalence at baseline and statistical methods used to control for baseline differences	Yes	Pg16

TREND Statement Checklist

Numbers analyzed	16	• Number of participants (denominator) included in each analysis for each study condition, particularly when the denominators change for different outcomes; statement of the results in absolute numbers when feasible	Yes	Pg16-21
		• Indication of whether the analysis strategy was "intention to treat" or, if not, description of how non-compliers were treated in the analyses	Yes	Pg17-21
Outcomes and estimation	17	• For each primary and secondary outcome, a summary of results for each estimation study condition, and the estimated effect size and a confidence interval to indicate the precision	Yes	Pg17-21
		• Inclusion of null and negative findings	Yes	Pg19
		• Inclusion of results from testing pre-specified causal pathways through which the intervention was intended to operate, if any	NA	NA
Ancillary analyses	18	• Summary of other analyses performed, including subgroup or restricted analyses, indicating which are pre-specified or exploratory	Yes	Pg22-23
Adverse events	19	• Summary of all important adverse events or unintended effects in each study condition (including summary measures, effect size estimates, and confidence intervals)	Yes	Pg23-24
**DISCUSSION**				
Interpretation	20	• Interpretation of the results, taking into account study hypotheses, sources of potential bias, imprecision of measures, multiplicative analyses, and other limitations or weaknesses of the study	Yes	Pg25-29
		• Discussion of results taking into account the mechanism by which the intervention was intended to work (causal pathways) or alternative mechanisms or explanations	Yes	Pg25-29
		• Discussion of the success of and barriers to implementing the intervention, fidelity of implementation	Yes	Pg30-32
		• Discussion of research, programmatic, or policy implications	Yes	Pg32
Generalizability	21	• Generalizability (external validity) of the trial findings, taking into account the study population, the characteristics of the intervention, length of follow-up, incentives, compliance rates, specific sites/settings involved in the study, and other contextual issues	Yes	Pg31
Overall Evidence	22	• General interpretation of the results in the context of current evidence and current theory	Yes	Pg32

From: Des Jarlais, D. C., Lyles, C., Crepaz, N., & the Trend Group (2004). Improving the reporting quality of nonrandomized evaluations of behavioral and public health interventions: The TREND statement. *American Journal of Public Health*, 94, 361-366. For more information, visit: http://www.cdc.gov/trendstatement/
