# Supplementary material for: Does curve pattern impact on the effects of physiotherapeutic scoliosis specific exercises on Cobb angles of participants with adolescent idiopathic scoliosis: A prospective clinical trial with two years follow-up
Source: PLoS One. 2021 Jan 25;16(1):e0245829. doi: 10.1371/journal.pone.0245829 (PMC7833215; doi:10.1371/journal.pone.0245829)
Supplement: Attachment — Submitted filename: PONE-D-19-35759_reviewer (ep).pdf [file pone.0245829.s006.pdf]

PLOS ONE

Dose Curve Pattern Impact on the Effects of Physiotherapeutic Scoliosis Specific Exercises on Cobb angle of Adolescent Idiopathic Scoliosis Subjects: A prospective clinical controlled trial with two years follow-up

--Manuscript Draft--

**Manuscript Number:**	PONE-D-19-35759
**Article Type:**	Clinical Trial
**Full Title:**	Dose Curve Pattern Impact on the Effects of Physiotherapeutic Scoliosis Specific Exercises on Cobb angle of Adolescent Idiopathic Scoliosis Subjects: A prospective clinical controlled trial with two years follow-up
**Short Title:**	Dose Curve Pattern Impact on Effects of Physiotherapeutic Exercises Adolescent Idiopathic Scoliosis Subjects
**Corresponding Author:**	Yunli Fan<br>University of Hong Kong<br>Hong Kong, HONG KONG
**Keywords:**	Adolescent idiopathic scoliosis; Curve Pattern; Cobb Angle; Physiotherapeutic Scoliosis Specific Exercise; Conservative Management; Scoliosis Progression; Compliance
**Abstract:**	Background: The lumbar spine has higher mobility than the thoracic spine and may contribute to better curve correction with bracing or surgery. Current clinical evidence suggests that a well-planned Physiotherapeutic Scoliosis Specific Exercises (PSSE) program is an effective method for scoliosis regression. The aim of this study is to investigate the effect of curve pattern on Cobb angle changes with PSSE.<br><br>Methods: This was a prospective clinical controlled study recruiting ~~patients~~ with adolescent idiopathic scoliosis ~~since~~ January to June of 2017 and follow-up till December 2019. Subjects with thoracic ~~major~~ curves were enrolled into group A and lumbar ~~major~~ curves were enrolled into group B. An outpatient-based PSSE program was carried out with intensive exercise frequency in the first 6 months and reduced exercise frequency until two years after the PSSE program. Any radiographic Cobb angle progressions were identified at one-year, one and a half years and two-years follow-up. Comparison was made by repeated measures one-way analysis of variance to analyse differences between the two groups and intra-group analysis by multiple paired t tests was performed to detect any curve regression outcome differences between subjects with similar curve patterns. The level of significance was set to be 0.05 with the two-tailed test.<br><br>Results: There were 40 subjects recruited for this study with 22 thoracic major cases (5 males and 17 females, mean age 13.5±1.8 years) and 18 lumbar major cases (7 males and 11 females, mean age 12.7±1.7 years). No difference in initial Cobb angle was observed between groups. There was no statistical significant difference in the absolute value of difference (Absolute D-Value) in Cobb angles between the two groups (one year post-PSSE: 4.0±2.9 degrees vs 4.3±2.6 degrees; one and a half years post-PSSE: 3.6±3.2 degrees vs 5.2±2.9degrees; two years post-PSSE: 3.6±2.3 degrees vs 4.8±3.1 degrees; $F_{1,38}$ =1.69, p=0.2). Curve regression was noted in 9.1% of subjects in group A and 33.3% in group B at two-year follow-up. Statistically significant Cobb angle reduction was observed in group B after one and a half years (3.5±4.9 degrees) and maintained after two years of PSSE (3.8±4.3 degrees).<br><br>Conclusion: This was the first study to investigate whether PSSE can lead to curve regression based on location of the major curve. No significant differences in correction effect by PSSE were observed between thoracic and lumbar curves. However, regression effect and no curve deterioration were noted in both groups at two-year follow-up.<br><br>Keywords: Physiotherapeutic Scoliosis Specific Exercises (PSSE), Adolescent idiopathic scoliosis (AIS), Conservative management, Exercise compliance, Scoliosis deterioration.

Powered by Editorial Manager® and ProduXion Manager® from Aries Systems Corporation

	Trial Registration: ChiCTR1900028073 (Chinese Clinical Trial Registry)
**Order of Authors:**	Yunli Fan
	Eric HK Yeung
	Jianbin Wu
	Rong He
	Zhuoman Xu
	Ruiwen Zhang
	Guangshuo Li
	Kenneth MC Cheung
	Michael KT TO
	Jason PY Cheung
**Additional Information:**	
**Question**	**Response**
**Financial Disclosure**<br><br>Enter a financial disclosure statement that describes the sources of funding for the work included in this submission. Review the submission guidelines for detailed requirements. View published research articles from *PLOS ONE* for specific examples.<br><br>This statement is required for submission and **will appear in the published article** if the submission is accepted. Please make sure it is accurate.<br><br>**Unfunded studies**<br>Enter: *The author(s) received no specific funding for this work.*<br><br>**Funded studies**<br>Enter a statement with the following details:<br>• Initials of the authors who received each award<br>• Grant numbers awarded to each author<br>• The full name of each funder<br>• URL of each funder website<br>• Did the sponsors or funders play any role in the study design, data collection and analysis, decision to publish, or preparation of the manuscript?<br>• **NO** - Include this sentence at the end of your statement: *The funders had no role in study design, data collection and analysis, decision to publish, or preparation of the manuscript.*<br>• **YES** - Specify the role(s) played.	Funding Project: Sanming Project of Medicine (SZSM201612055) "Team of Excellence in Spinal Deformities and Spinal Degeneration Diseases" in Shenzhen, Guangdong province, China.<br><br>Receiver: Kenneth MC Cheung<br><br>Professor Kenneth Cheung participated in the methodology and preparation of the manuscript.

Powered by Editorial Manager® and ProduXion Manager® from Aries Systems Corporation

<table>
<tr><td>* typeset</td><td></td></tr>
<tr><td>Competing Interests

Use the instructions below to enter a competing interest statement for this submission. On behalf of all authors, disclose any competing interests that could be perceived to bias this work—acknowledging all financial support and any other relevant financial or non-financial competing interests.

This statement will appear in the published article if the submission is accepted. Please make sure it is accurate. View published research articles from PLOS ONE for specific examples.

NO authors have competing interests

Enter: The authors have declared that no competing interests exist.

Authors with competing interests

Enter competing interest details beginning with this statement:

I have read the journal's policy and the authors of this manuscript have the following competing interests: [insert competing interests here]

* typeset</td><td>The authors have declared that no competing interests exist.</td></tr>
<tr><td>Ethics Statement

Enter an ethics statement for this submission. This statement is required if the study involved:

• Human participants
• Human specimens or tissue
• Vertebrate animals or cephalopods
• Vertebrate embryos or tissues
• Field research

Write "N/A" if the submission does not</td><td>Human Subject Research
•Name of Committe:
The Ethic Committe Board of The University of Hong Kong - Shenzhen Hospital

•Approval number:No.:伦[2016]91
Original protocol: GNSF201603

•Form of consent obtained (written/oral) : Written</td></tr>
</table>

Powered by Editorial Manager® and ProduXion Manager® from Aries Systems Corporation

require an ethics statement.<br><br>General guidance is provided below. Consult the submission guidelines for detailed instructions. **Make sure that all information entered here is included in the Methods section of the manuscript.**<br><br>**Format for specific study types**<br><br>**Human Subject Research (involving human participants and/or tissue)**<br>• Give the name of the institutional review board or ethics committee that approved the study<br>• Include the approval number and/or a statement indicating approval of this research<br>• Indicate the form of consent obtained (written/oral) or the reason that consent was not obtained (e.g. the data were analyzed anonymously)<br><br>**Animal Research (involving vertebrate animals, embryos or tissues)**<br>• Provide the name of the Institutional Animal Care and Use Committee (IACUC) or other relevant ethics board that reviewed the study protocol, and indicate whether they approved this research or granted a formal waiver of ethical approval<br>• Include an approval number if one was obtained<br>• If the study involved *non-human primates*, add *additional details* about animal welfare and steps taken to ameliorate suffering<br>• If anesthesia, euthanasia, or any kind of animal sacrifice is part of the study, include briefly which substances and/or methods were applied<br><br>**Field Research**<br><br>Include the following details if this study involves the collection of plant, animal, or other materials from a natural setting:<br>• Field permit number<br>• Name of the institution or relevant body that granted permission	
**Data Availability**	Yes - all data are fully available without restriction

Powered by Editorial Manager® and ProduXion Manager® from Aries Systems Corporation

Authors are required to make all data underlying the findings described fully available, without restriction, and from the time of publication. PLOS allows rare exceptions to address legal and ethical concerns. See the PLOS Data Policy and FAQ for detailed information.<br><br>A Data Availability Statement describing where the data can be found is required at submission. Your answers to this question constitute the Data Availability Statement and **will be published in the article**, if accepted.<br><br>**Important:** Stating 'data available on request from the author' is not sufficient. If your data are only available upon request, select 'No' for the first question and explain your exceptional situation in the text box.<br><br>Do the authors confirm that all data underlying the findings described in their manuscript are fully available without restriction?	
**Describe where the data may be found in full sentences. If you are copying our sample text, replace any instances of XXX with the appropriate details.**<br><br>• If the data are **held or will be held in a public repository**, include URLs, accession numbers or DOIs. If this information will only be available after acceptance, indicate this by ticking the box below. For example: *All XXX files are available from the XXX database (accession number(s) XXX, XXX.*).<br>• If the data are all contained **within the manuscript and/or Supporting Information files**, enter the following: *All relevant data are within the manuscript and its Supporting Information files.*<br>• If neither of these applies but you are able to provide **details of access elsewhere**, with or without limitations, please do so. For example:	All relevant data are within the manuscript and its Supporting Information files.<br><br>data (same as in supporting information files) will be availbale on an open public access if paper recieved: ResMan<br>www.medresman.org

Powered by Editorial Manager® and ProduXion Manager® from Aries Systems Corporation

Data cannot be shared publicly because of [XXX]. Data are available from the XXX Institutional Data Access / Ethics Committee (contact via XXX) for researchers who meet the criteria for access to confidential data.<br><br>*The data underlying the results presented in the study are available from (include the name of the third party and contact information or URL).*<br>• This text is appropriate if the data are owned by a third party and authors do not have permission to share the data.<br><br>* typeset	
Additional data availability information:	Tick here if the URLs/accession numbers/DOIs will be available only after acceptance of the manuscript for publication so that we can ensure their inclusion before publication.

Powered by Editorial Manager® and ProduXion Manager® from Aries Systems Corporation

Manuscript

Click here to access/download;Manuscript;Curve_Pattern_Final_PlosOne_w

Title: Dose Curve Pattern Impact on the Effects of Physiotherapeutic Scoliosis Specific Exercise on the Cobb Angle in Adolescent Idiopathic Scoliosis Subjects: A prospective clinical trial with a two-year follow-up

Authors

1. Yunli Fan[1]
2. Eric HK Yeung[1]
3. Jianbin Wu[1]
4. Rong He[1]
5. Zhuoman Xu[1]
6. Ruiwen Zhang[1]
7. Guangshuo Li[1]
8. Kenneth MC Cheung[3]
9. Michael KT To[3]
10. Jason PY Cheung[3]

[1]The University of Hong Kong – Shenzhen Hospital, Shenzhen, Guangdong Province, People's Republic of China

[2]Department of Orthopaedics and Traumatology, Li Ka Shing Faculty of Medicine, The University of Hong Kong, Pokfulam, Hong Kong Special Administration Region, China

Correspondence (for review) for contact purposes only:

Name	Yunli Fan
Department	Department of Physiotherapy
Institution	The University of Hong Kong – Shenzhen Hospital
Country	China
Tel	(001-0755) 8691-3170
Mob	(00186) 13421836860
Fax	(001-0755) 8691-3333
Email	fanyl@hku-szh.org

Correspondence (for publication) for contact purposes only:

Name	Cheung, Jason Pui Yin
Department	Department of Orthopaedics and Traumatology
Institution	The University of Hong Kong
Country	Hong Kong Special Administration Region, China
Email	cheungjp@hku.hk

Dose Curve Pattern Impact on the Effects of Physiotherapeutic Scoliosis Specific Exercise on the Cobb Angle in Adolescent Idiopathic Scoliosis Subjects: A prospective clinical trial with a two-year follow-up

Yunli Fan[1], Eric HK Yeung[1], Jianbin Wu[1], Rong He[1], Zhuoman Xu[1], Ruiwen Zhang[1], Guangshuo Li[1], Kenneth MC Cheung[2], Michael KT To[2*], Jason PY Cheung[2*]

Abstract

Background: The lumbar spine has higher mobility than the thoracic spine and may contribute to better curve correction with bracing or surgery. Current clinical evidence suggests that a well-planned Physiotherapeutic Scoliosis Specific Exercise (PSSE) program is an effective method for scoliosis regression. The present study investigated the effect of the curve pattern on Cobb angle changes with PSSE.

Methods: This was a prospective clinical controlled study recruiting patients with adolescent idiopathic scoliosis from 14th January to 31st June 2017 and follow-up till 30th December 2019. Thoracic major curves were enrolled into group A, and lumbar major curves were enrolled into group B. An outpatient-based PSSE program was carried out with intensive exercise frequency in the first 6 months and reduced exercise frequency until two years after the PSSE program. Any radiographic Cobb angle progressions were identified at the one-year, one-and-a-half-year and two-year follow-ups. Comparisons were made by repeated measures one-way analysis of variance to analyze differences between the two groups, and intragroup analysis by multiple paired t-tests was performed to detect any curve regression outcome differences between subjects with similar curve patterns. The level of significance was set to 0.05 with the two-tailed test.

Results: Forty subjects were recruited for this study, including 22 thoracic-major cases (5 males and 17 females, mean age 13.5±1.8 years) and 18 lumbar-major cases (7 males and 11 females, mean age 12.7±1.7 years). No difference in initial Cobb angle was observed between groups. There was no significant difference in the absolute value of difference (absolute D-value) in Cobb angles between the two groups (one year post-PSSE: 4.0±2.9 degrees vs 4.3±2.6 degrees; 1.5 years post-PSSE: 3.6±3.2 degrees vs 5.2±2.9 degrees; two years post-PSSE: 3.6±2.3 degrees vs 4.8±3.1 degrees; $F_{1,38}=1.69$, $p=0.2$). Curve regression was noted in 9.1% of subjects in group A and 33.3% in group B at the two-year follow-up. A significant Cobb angle reduction was observed in group B after 1.5 years (3.5±4.9 degrees) and was maintained after two years of PSSE (3.8±4.3 degrees).

Conclusion: This is the first study to investigate whether PSSE can lead to curve regression based on the location of the major curve. No significant differences in the PSSE correction effect were observed between thoracic and lumbar curves. However, a regression effect and no curve deterioration were noted in both groups at the two-year follow-up.

Keywords: Physiotherapeutic Scoliosis Specific Exercise (PSSE), Adolescent idiopathic scoliosis (AIS), Conservative management, Exercise compliance, Scoliosis deterioration.

* Correspondence: cheungjp@hku.hk; mikektto@hku.hk

[1] The University of Hong Kong – Shenzhen Hospital, Shenzhen, Guangdong Province, People's Republic of China

[2] Department of Orthopaedics and Traumatology, Li Ka Shing Faculty of Medicine, The University of Hong Kong, Pokfulam, Hong Kong Special Administration Region, China

Trial Registration: ChiCTR1900028073

Background

Adolescent idiopathic scoliosis (AIS) is a three-dimensional spinal deformity with an unknown etiology, characterized by lateral deviation in the frontal plane, axial rotation in the horizontal plane and an abnormal sagittal curvature[1]. Surgery is usually recommended if the curve reaches 50 degrees, as this is associated with a continued progression risk into adulthood[2]. Surgical fusion undoubtedly leads to stiff spines and should be avoided if possible. Thus, the goal of conservative management is to prevent spinal deformity deterioration into the operative threshold.

The option for nonsurgical management varies widely and depends on the strength of the prognostic evaluation of curve progression[3]. The curve type is one of the established risk factors of scoliosis progression[4]. In particular, a larger Cobb angle with the presence of a thoracic curve shows higher progression odds than a single lumbar or thoracolumbar curve[4]. Therefore, understanding the impact of curvature on intervention is valuable and helpful for clinicians to choose proper treatments for patients. The brace method is one of the most established methods and has shown the ability to correct scoliotic deformities, especially for thoracolumbar or lumbar curves, which are more mobile[5]. Correction of deformities is highly influenced by increased mobility of spinal segments[6-9]. In addition, scoliosis-specific exercise is another nonoperative method that is well received by patients and parents[10]. There are several systematic reviews and randomized controlled trials reporting the positive effects of Physiotherapeutic Scoliosis Specific Exercise (PSSE) on slowing the curve progression as well as improving the cosmetic appearance and quality of life (QOL) outcomes[11-13]; however, in these studies, the relationship of curve location with correction effects was not clearly discussed, and there was only a short-term follow-up. Specifically, Schroth is the most commonly used PSSE approach, which adopts specific respiratory technique-asymmetrical breathing in the diagonal direction for vertebral and rib cage derotation. It utilizes muscle activation and emphasizes core muscle stabilization of the corrected posture throughout the day to change habitual postures and improve spinal alignment[14]. However, the influence of curve magnitude on exercise outcomes is unknown. The relationship between spinal flexibility and PSSE is also not well established. It is unclear which profile of spinal deformity leads to the best outcomes with PSSE. Thus, this study aimed to investigate the long-term therapeutic effect of PSSE on thoracic and lumbar major curves and to determine whether a more flexible lumbar curve would respond better to exercise. The results of this study will provide physiotherapists with additional information to evaluate individualized, curve magnitude-based exercises for patients and will form the basis for further controlled trials to evaluate the relationship between spinal flexibility and PSSE.

Methodology

<u>Study Design</u>

A prospective, clinical controlled trial was conducted on patients with AIS who met the International Society on Scoliosis Orthopaedic and Rehabilitation Treatment (SOSORT) guidelines for PSSE[15]. Patients were recruited for PSSE treatment and followed up for two years thereafter. The CONSORT flow diagram and checklist are available in Fig 1 and in S1, respectively. The inclusion criteria were as follows: age: 10 to 16 years; bone immaturity in terms of Sanders stage: less than 8[16]; Cobb angles: from 10 degrees to 50 degrees; and complete attendance to all physiotherapy sessions. Exclusion criteria were diagnoses other than

AIS, disabilities or systemic illnesses preventing exercise performance, hypermobility (Beighton score[17] greater than 4) and previous treatment for AIS. The Rigo Scoliosis Classification system was applied to categorize subjects and define the appropriate exercise program[18]. Subjects with 3C (thoracic major with or without a minor lumbar) or N3N4 curves (double curves but well balanced) were considered thoracic major (group A), and those with single lumbar/single thoracolumbar or 4C curves (lumbar major with a minor thoracic) were considered lumbar major (group B).

Subjects

40 subjects were recruited between January 2017 and June 2017 from the Out-patient clinic of the department of orthopaedics and referred to physiotherapy department for PSSE intervention. All subjects agreed and signed consent form before commencing the study, evaluations within the trial occurred between June 2017 and December 2019 and the local ethic committee board approved the study protocol (GNSF201603) prior to beginning subject recruitment. However, this trial was retrospective registered (December 2019) in registry of clinical trials (Chinese Clinical Trial Registry, Registration Number: ChiCTR1900028073), because we were not aware that the registration was needed beyond being approved by ethic committee. Despite of late registration, this study was conducted in consistency with original intervention protocol and no dropouts during the whole follow-up period.

Study Intervention

All subjects attended 6-month intensive PSSE supervised by two PSSE-Schroth approach certified physiotherapists. The frequency and time of supervised treatment during the 6 months were ≥1 time per month and one hour each time. The subjects were instructed to perform >30 minutes of home exercise/session with ≥5 sessions a week. After the first 6 months of PSSE, all subjects could reduce the frequency of supervised PSSE to once per three months to review the exercises performed until two years after completion of the PSSE program. During this time, the frequency of home exercise was maintained. Attendance of supervised PSSE in the outpatient setting was collected from the hospital prospective database system. Each session of treatment was one hour. One simple questionnaire was completed by all subjects themselves to check their home exercise compliance. Exercise compliance was calculated and documented in hours per week (Table 1) for each year. The PSSE intervention consisted of 50 breaths per exercise. Short semihanging (Fig. 3: c), corrective exercises in the standing (Fig. 3: f), sitting (Fig. 3: e) and lateral (Fig. 3: d) positions were prescribed for subjects in group A. Short semihanging (Fig. 4: c), muscle cylinder (Fig. 4: f), corrective exercises in the lateral (Fig. 4: d), sitting (Fig. 4: g) and standing (Fig. 4: e) positions were prescribed to subjects in group B. The difference between the two groups was that a muscle cylinder was only applied to the lumbar major patients (group B). Subjects were asked to record and report any discomfort, such as muscle fatigue or muscle sprain, to the on-duty physiotherapist during the study period.

Outcome Measurement

Subjects received whole-spine standing radiographs to assess their major curve Cobb angles. The assessor of the images was blinded to the patient information and treatment details. The Cobb angle was collected at the initial assessment and every 6 months after the PSSE program until the two-year follow-up. All subjects remained out of their braces overnight prior to obtaining radiographs. Curve regression was defined as a decrease in the Cobb angle by 6 degrees or more, stabilization was defined as a <6 degree change in the Cobb angle, and deterioration was defined as a Cobb angle increase of 6 degrees or more.

Statistics

Student's t test was used to compare continuous variables, including age and initial Cobb angles. The chi-square test was used to compare categorical variables including gender, Sanders stage, curvature types (single or double curves) and brace treatment for all subjects (Table 1). The absolute D-value of the change in the Cobb angle during the study period between the two groups was compared with repeated measures one-way analysis of variance (ANOVA), and intragroup analysis by multiple paired t tests was performed to detect any curve regression outcome differences in patients with similar curve patterns. The data were analyzed using SPSS version 20.0 (IBM, Chicago, IL). The level of significance was set to 0.05 with a two-tailed test.

Results:

Subjects

Forty subjects were recruited into this study. Twenty-two subjects (5 males and 17 females) were recruited into group A, and 18 subjects (7 males and 11 females) were recruited into group B. The mean age was 13.5±1.8 years in group A and 12.7±1.7 years in group B. The two groups were similar at baseline for age, gender, brace treatment, Sanders stage, proportion of curvature types and Cobb angle of the major curve (Table 1). There were more than 50% of subjects with Sanders stage 0-3 and five of them with Cobb angle ≧ 30 degrees in this study (Table 1). Those subjects started brace treatment before commencing the PSSE program except for one subject in group B (Fig. 5) who refused brace treatment.

Compliance

There was a significant difference in exercise compliance during the second year of follow-up between the two groups (3.0±0.8 hours/week vs 4.1±2.2 hours/week, p=0.05). In addition, the mean exercise compliance of group A was significantly decreased in the second year (5.5±2.2 hours/week vs 3.0±0.8 hours/week; p<0.01); the mean exercise compliance of group B was 5.5±2.1 hours per week in the first year and slightly decreased to 4.1±2.2 hours per week in the second year. There were no adverse effects detected during this study period.

Effects of the Intervention

In total, 57.5% of subjects refused to repeat radiographs at the 6-month follow-up after PSSE due

to concerns regarding radiation exposure; hence, no comparison was conducted to analyze the difference pre- and post-6-month follow-up of PSSE.

There was no deterioration of the major curvature in either group at the 2-year follow-up (group A: 1.0±4.2 degrees; group B: 3.8±4.3 degrees). In addition, no differences in the absolute D-value of the Cobb angle between group A and group B were noted (one year post-PSSE: 4.0±2.9 degrees vs 4.3±2.6 degrees; one and a half years post-PSSE: 3.6±3.2 degrees vs 5.2±2.9 degrees; two years post-PSSE: 3.6±2.3 degrees vs 4.8±3.1 degrees; $F_{1,38}$=1.69, p=0.2) after regular PSSE for two years Subjects (Table 1: 13.6% in group A; 5.6% in group B) with Cobb angle ≥ 30 degrees and Sanders stage 0-3 showed no scoliosis deterioration (3.6±4.9 degrees). Subjects (Table 1: 31.9% in group A; 22.2% in group B) who had a Cobb angle ≥ 30 degrees but were older at Sanders stage 4-7 showed no deterioration (4.5±3.6 degrees) at the two-year follow-up.

In group B, the intragroup analysis revealed a mild reduction in the Cobb angle (26.8±10.1 degrees vs 23.3±9.4 degrees, *p*=0.007) after training for one and a half years with a maintained reduction after two years of follow-up (26.8±10.1 degrees vs 22.9±9.2 degrees, *p*=0.001) (Fig. 2). In comparison to the initial Cobb angle in group B, the curves improved (D-value of Cobb angles: 9.5±4.9 degrees) in 11.1%, stabilized (absolute D-value of Cobb angles: 3.4±1.2 degrees) in 83.3%_and deteriorated by 7 degrees in one subject after the first year of training. In the second year of follow-up for group B, up to 33.3% of subjects had improved Cobb angles (D-value of Cobb angles: 8.2±2.5 degrees), while 66.7% stabilized (Absolute D-value of Cobb angles: 3.2±1.6 degrees), and no deterioration was noted. Of the subjects with curve regression in the first year, one maintained the curve regression (D-value of Cobb: 13 degrees) and one only gained an additional degree (D-value of Cobb: 5 degrees) in the second year. Moreover, one subject in group B with single lumbar scoliosis of 31 degrees (Fig. 5: a) and in Sanders stage 3 (Fig. 5: b) refused brace treatment but obtained in a Cobb angle of 23 degrees (Fig. 5: c) after one and a half years of PSSE. In addition, another subject in group B with single thoracolumbar scoliosis with a Cobb angle of 44 degrees (Fig. 6: a) at Sanders stage 6 (Fig. 6: b) obtained a Cobb angle of 31 degrees (Fig. 6: c) after two years of PSSE.

In comparison to the initial Cobb angle in group A, there was no significant difference in Cobb angle (28.5±8.4 degrees vs 27.0±7.2 degrees, 28.5±8.4 degrees vs 27.1±7.7 degrees, 28.5±8.4 degrees vs 27.5±7.0 degrees, *p*>0.05) after one year, one and a half years and two years of follow-up, respectively. However, after training, the spinal deformity improved (D-value of Cobb angles: 8.8±2.5 degrees) in 18.2% of subjects and stabilized (absolute D-value of Cobb angles: 2.9±1.6 degrees) in 81.8% of subjects, and none deteriorated in the first year. Only half of the subjects maintained curve regression in the second year (10.5±2.1 degrees vs 8.5±2.1 degrees), and only 9.1% of subjects showed regression (D-value of Cobb angles: 8.5±2.1 degrees). Up to 90.9% of subjects stabilized (absolute D-value of Cobb angles: 3.1±1.6 degrees), and no deterioration was detected at the two-year follow-up.

Discussion

This is the first study to investigate the outcomes of different curve types undergoing PSSE treatment for AIS. The findings of this study show that there was no significant difference in the

subjects' absolute D-values of the Cobb angle after regular PSSE for two years regardless of the thoracic or lumbar major curve, which implied that the curve pattern did not significantly impact the effects of PSSE in patients with AIS. No curve progression was found in either group at the end of this study, suggesting that a well-planned PSSE program can prevent scoliosis deterioration. This result is consistent with earlier findings of PSSE in reducing curve progression for patients with mild AIS[19-21]. By comparison, our study even observed regression effects in larger curves and with a longer follow-up. Moreover, it is important to note that most of our patients had a high progression risk[22], with more than 50% at Sanders stage 0-3 and 40% with a Cobb angle ≥ 30 degrees. Subjects with a ≥ 30 degree Cobb angle and in Sanders stage 0-3 started brace treatment at study initiation. The outcome of no deterioration may be attributed to the effects of both bracing and PSSE. Subjects who had a Cobb angle ≥ 30 degrees but were older with a Sanders stage between 4 and 7 still showed no deterioration after two years of PSSE regardless of brace treatment. Therefore, our results further revealed the superiority of PSSE programs in the stabilization and regression of scoliosis.

After two years of follow-up, no clinical deterioration was detected in either group, which suggested the promising effects of PSSE on the prevention of scoliosis progression. Beyond that, the regression effect was detected in both groups despite the curvature magnitude. Specifically, more subjects in group A showed curve regression in the first year, but a significant decrease in compliance may have led to less of a regression effect at the 2-year follow-up. In contrast, a significant reduction in the Cobb angle was noted, especially for subjects with lumbar major curves; moreover, a significant difference was observed as early as one and a half years after training, and reduction was maintained at the end of this study. This met our original study hypothesis that lumbar major curves are more flexible and thus respond better to exercises. However, this conclusion should be interpreted with caution, as the lumbar spine is inherently more flexible, with better exercise compliance and its associated effects of good core lumbar muscle stabilization. The superior flexibility of the lumbar spine compared to the thoracic spine has been proven in biomechanical studies[23-25]. The lumbar spine is mainly stabilized by core muscles that are flexible, stretchable and respond to external forces very well, whereas the stabilization mechanism in the thoracic spine is reliant on the rib cage, which contributes to rigidity and stability[24-26]. Additionally, according to the general principles of the Schroth method, correcting directions including side shift, axial elongation and derotation are created by muscle lengthening and eccentric contraction in the lumbar region, whereas spiral breathing is adopted in the thoracic region for correction[14]. Spiral breathing is difficult to learn and hard to control for most patients with AIS due to the requirement of breathing diagonally and asymmetrically not only in the frontal plane but also in the horizontal plane. Therefore, it is relatively easier for patients to correct the lumbar curvature via pelvic positioning, lengthening the curve concavity and stabilizing it in the proper position. In addition, stabilization effects from core muscles are crucial for maintaining correction in lumbar spines[14, 26]. Therefore, the lumbar curvature may respond better to the three-dimensional correction forces.

Our study revealed that administering an outpatient home-based PSSE program is a feasible protocol with reasonable compliance. The parents were asked to send monthly videos documenting the exercises performed to the on-duty therapist, which provided a reasonable

variation control with respect to exercise quality and compliance. The frequency of such home-based programs was collected via a questionnaire, and exercise compliance was interpreted by how long subjects spent on the PSSE program. The program included both supervised PSSE sessions and home exercise sessions that were calculated in total hours per week. Hence, we were unable to analyze specifically whether supervised PSSE is superior to home exercises in curve correction despite previous accounts of such a relationship[19-21]. Hence, this aspect will require further study. However, our finding is consistent with those of other studies supporting the positive effects of PSSE, which are strongly correlated with good exercise compliance[27-29]. In our study, significant differences in exercise compliance between the two groups were noted in the second year, and the better compliance may have contributed to the improved Cobb angle reduction in group B.

This study has several limitations. First, there was no untreated control group for the comparison of intervention effects but due to the proven benefits of PSSE on AIS[11-13], it was unethical to leave patients without treatment for as long as 2 years. Thus, the benefit observed may have been influenced by natural recovery or deterioration of the condition. Second, we only categorized patients into two groups, yet there were four scoliosis types according to the Rigo classification concept. For subjects having a double curve type such as N3N4 or 4C, the minor curve will cause limits to overstretching of the concavity from the major curve that weaken the effects of correction in the major curve. However, the proportion of double curves in this study was similar (Table 1: 40.9% vs 44.4%) between the two groups, which could not cause large variations in the data. However, it will be better for further studies to compare the effects of PSSE for all four curve types separately. Third, we were unable to blind the subjects or physiotherapists, which may have caused variations during the exercise prescription. However, the frequency and dosage of both supervised and home programs were strictly fixed, and the performed exercises were checked monthly by therapists, which limited systematic measurement errors such as learning effects. Finally, the hypothesis of this study assumed that the lumbar spine may respond better due to increased spinal flexibility, but no bending radiographic assessment was studied before commencing the PSSE program due to subjects' concerns regarding radiation exposure. This study only excluded patients with AIS who reported hypermobility, but without quantifying the segmental flexibility of the scoliosis.

Conclusion

There were no differences in outcomes of PSSE treatment based on either thoracic or lumbar curve types. In general, PSSE with reasonable exercise compliance has positive effects on preventing scoliosis progression. However, further studies are necessary to address the correlation between spinal flexibility and the correction effects of PSSE at different curve magnitudes.

Acknowledgment:

This study was financially supported by the Sanming Project of Medicine (SZSM201612055) "Team of Excellence in Spinal Deformities and Spinal Degeneration Diseases" in Shenzhen, Guangdong Province, China.

Fig.1: CONSORT flow Diagram

Table 1: Baseline characteristics of subjects from group A and group B.

Fig. 2: Intragroup analysis showing improved Cobb angles before ~~and~~ 1 year, 1.5 years and 2 years after the PSSE program. * $p<0.05$.

Fig. 3: a: Pre-exercise radiograph showing a thoracic major curve (T4 to 12) with a Cobb angle of 30 degrees. **b:** Posterior posture view of the ~~subject~~. **c:** Short semihanging. **d:** Corrective exercise in the lateral position. **e:** Corrective exercise in the sitting position. **f:** Corrective exercise in the standing position.

Fig. 4: a: Pre-exercise radiograph showing a lumbar major curve (T12 to L4) with a Cobb angle of 48 degrees. **b:** Posterior posture view of the ~~subject~~. **c:** Short semihanging. **d:** Corrective exercise in the lateral position. **e:** Corrective exercise in the standing position. **f:** Muscle cylinder applied in the standing position. **g:** Corrective exercise in the sitting position.

Fig. 5: Radiographic assessment of one subject with single lumbar scoliosis without brace treatment. **a:** Initial Cobb angle of 31 degrees from T12 to L4. **b:** Initial Sanders stage 3. **c:** Cobb angle of 23 degrees from T12 to L4 after one and a half years of follow-up.

Fig. 6: Radiographic assessment of one subject with single thoracolumbar scoliosis without brace treatment. **a:** Initial Cobb angle of 44 degrees from T5 to L2. **b:** Initial Sanders stage 6. **c:** Cobb angle of 31 degrees from T5 to L2 after the two-year follow-up.

References:

1. Cheng JC, Castelein RM, Chu WC, Danielsson AJ, Dobbs MB, Grivas TB, et al. Adolescent idiopathic scoliosis. Nature reviews Disease primers. 2015;1:15030. Epub 2015/01/01. doi: 10.1038/nrdp.2015.30. PubMed PMID: 27188385.

2. Weinstein SL. The Natural History of Adolescent Idiopathic Scoliosis. Journal of pediatric orthopedics. 2019;39(Issue 6, Supplement 1 Suppl 1):S44-s6. Epub 2019/06/07. doi: 10.1097/bpo.0000000000001350. PubMed PMID: 31169647.

3. Sy N, Bettany-Saltikov J, Moramarco M. Evidence for Conservative Treatment of Adolescent Idiopathic Scoliosis - Update 2015 (Mini-Review). Current pediatric reviews. 2016;12(1):6-11. Epub 2015/11/18. PubMed PMID: 26573167.

4. Dolan LA, Weinstein SL, Abel MF, Bosch PP, Dobbs MB, Farber TO, et al. Bracing in Adolescent Idiopathic Scoliosis Trial (BrAIST): Development and Validation of a Prognostic Model in Untreated Adolescent Idiopathic Scoliosis Using the Simplified Skeletal Maturity System. Spine Deform. 2019;7(6):890-8.e4. Epub 2019/11/17. doi: 10.1016/j.jspd.2019.01.011. PubMed PMID: 31731999.

5. Almansour H, Pepke W, Bruckner T, Diebo BG, Akbar M. Three-Dimensional Analysis of Initial Brace Correction in the Setting of Adolescent Idiopathic Scoliosis. Journal of clinical medicine. 2019;8(11). Epub 2019/10/31. doi: 10.3390/jcm8111804. PubMed PMID: 31661811.

6. He C, To MK, Cheung JP, Cheung KM, Chan CK, Jiang WW, et al. An effective assessment method of spinal flexibility to predict the initial in-orthosis correction on the patients with adolescent idiopathic scoliosis (AIS). PloS one. 2017;12(12):e0190141. Epub 2017/12/22. doi:

10.1371/journal.pone.0190141. PubMed PMID: 29267389; PubMed Central PMCID: PMCPMC5739463.
7. He C, Wong MS. Spinal Flexibility Assessment on the Patients With Adolescent Idiopathic Scoliosis: A Literature Review. Spine. 2018;43(4):E250-e8. Epub 2017/06/13. doi: 10.1097/brs.0000000000002276. PubMed PMID: 28604491.
8. Ohrt-Nissen S, Hallager DW, Gehrchen M, Dahl B. Flexibility Predicts Curve Progression in Providence Nighttime Bracing of Patients With Adolescent Idiopathic Scoliosis. Spine. 2016;41(22):1724-30. Epub 2016/04/15. doi: 10.1097/brs.0000000000001634. PubMed PMID: 27076435.
9. Yao G, Cheung JPY, Shigematsu H, Ohrt-Nissen S, Cheung KMC, Luk KDK, et al. Characterization and Predictive Value of Segmental Curve Flexibility in Adolescent Idiopathic Scoliosis Patients. Spine. 2017;42(21):1622-8. Epub 2016/12/21. doi: 10.1097/brs.0000000000002046. PubMed PMID: 27997505.
10. Negrini S, Carabalona R. Social acceptability of treatments for adolescent idiopathic scoliosis: a cross-sectional study. Scoliosis. 2006;1:14. Epub 2006/08/26. doi: 10.1186/1748-7161-1-14. PubMed PMID: 16930488; PubMed Central PMCID: PMCPMC1560163.
11. Burger M, Coetzee W, du Plessis LZ, Geldenhuys L, Joubert F, Myburgh E, et al. The effectiveness of Schroth exercises in adolescents with idiopathic scoliosis: A systematic review and meta-analysis. The South African journal of physiotherapy. 2019;75(1):904. Epub 2019/06/18. doi: 10.4102/sajp.v75i1.904. PubMed PMID: 31206094; PubMed Central PMCID: PMCPMC6556933.
12. Ceballos Laita L, Tejedor Cubillo C, Mingo Gomez T, Jimenez Del Barrio S. Effects of corrective, therapeutic exercise techniques on adolescent idiopathic scoliosis. A systematic review. Archivos argentinos de pediatria. 2018;116(4):e582-e9. Epub 2018/07/18. doi: 10.5546/aap.2018.eng.e582. PubMed PMID: 30016036.
13. Thompson JY, Williamson EM, Williams MA, Heine PJ, Lamb SE. Effectiveness of scoliosis-specific exercises for adolescent idiopathic scoliosis compared with other non-surgical interventions: a systematic review and meta-analysis. Physiotherapy. 2019;105(2):214-34. Epub 2019/03/03. doi: 10.1016/j.physio.2018.10.004. PubMed PMID: 30824243.
14. Lehnert-Schroth C. [Schroth's three dimensional treatment of scoliosis]. ZFA Zeitschrift fur Allgemeinmedizin. 1979;55(34):1969-76. Epub 1979/12/10. PubMed PMID: 547573.
15. Negrini S, Donzelli S, Aulisa AG, Czaprowski D, Schreiber S, de Mauroy JC, et al. 2016 SOSORT guidelines: orthopaedic and rehabilitation treatment of idiopathic scoliosis during growth. Scoliosis and spinal disorders. 2018;13:3. Epub 2018/02/13. doi: 10.1186/s13013-017-0145-8. PubMed PMID: 29435499; PubMed Central PMCID: PMCPMC5795289.
16. Sitoula P, Verma K, Holmes L, Jr., Gabos PG, Sanders JO, Yorgova P, et al. Prediction of Curve Progression in Idiopathic Scoliosis: Validation of the Sanders Skeletal Maturity Staging System. Spine. 2015;40(13):1006-13. Epub 2015/09/12. doi: 10.1097/brs.0000000000000952. PubMed PMID: 26356067.
17. Smits-Engelsman B, Klerks M, Kirby A. Beighton score: a valid measure for generalized hypermobility in children. The Journal of pediatrics. 2011;158(1):119-23, 23.e1-4. Epub 2010/09/21. doi: 10.1016/j.jpeds.2010.07.021. PubMed PMID: 20850761.
18. Rigo MD, Villagrasa M, Gallo D. A specific scoliosis classification correlating with brace treatment: description and reliability. Scoliosis. 2010;5(1):1. Epub 2010/03/09. doi: 10.1186/1748-7161-5-1. PubMed PMID: 20205842; PubMed Central PMCID: PMCPMC2825498.
19. Weiss HR, Lohschmidt K, el-Obeidi N, Verres C. Preliminary results and worst-case analysis of in patient scoliosis rehabilitation. Pediatric rehabilitation. 1997;1(1):35-40. Epub 1997/01/01. doi:

10.3109/17518429709060940. PubMed PMID: 9689236.
20. Weiss HR, Weiss G. Curvature progression in patients treated with scoliosis in-patient rehabilitation--a sex and age matched controlled study. Studies in health technology and informatics. 2002;91:352-6. Epub 2004/10/02. PubMed PMID: 15457754.
21. Weiss HR, Weiss G, Petermann F. Incidence of curvature progression in idiopathic scoliosis patients treated with scoliosis in-patient rehabilitation (SIR): an age- and sex-matched controlled study. Pediatric rehabilitation. 2003;6(1):23-30. Epub 2003/05/15. doi: 10.1080/1363849031000095288. PubMed PMID: 12745892.
22. Reamy BV, Slakey JB. Adolescent idiopathic scoliosis: review and current concepts. American family physician. 2001;64(1):111-6. Epub 2001/07/18. PubMed PMID: 11456428.
23. Hajihosseinali M, Arjmand N, Shirazi-Adl A, Farahmand F, Ghiasi MS. A novel stability and kinematics-driven trunk biomechanical model to estimate muscle and spinal forces. Medical engineering & physics. 2014;36(10):1296-304. Epub 2014/07/31. doi: 10.1016/j.medengphy.2014.07.009. PubMed PMID: 25074649.
24. Oxland TR. Fundamental biomechanics of the spine--What we have learned in the past 25 years and future directions. Journal of biomechanics. 2016;49(6):817-32. Epub 2015/12/27. doi: 10.1016/j.jbiomech.2015.10.035. PubMed PMID: 26706717.
25. Panjabi MM, White AA, 3rd. Basic biomechanics of the spine. Neurosurgery. 1980;7(1):76-93. Epub 1980/07/01. doi: 10.1227/00006123-198007000-00014. PubMed PMID: 7413053.
26. Eskandari AH, Arjmand N, Shirazi-Adl A, Farahmand F. Subject-specific 2D/3D image registration and kinematics-driven musculoskeletal model of the spine. Journal of biomechanics. 2017;57:18-26. Epub 2017/04/04. doi: 10.1016/j.jbiomech.2017.03.011. PubMed PMID: 28365064.
27. Schreiber S, Parent EC, Hill DL, Hedden DM, Moreau MJ, Southon SC. Patients with adolescent idiopathic scoliosis perceive positive improvements regardless of change in the Cobb angle - Results from a randomized controlled trial comparing a 6-month Schroth intervention added to standard care and standard care alone. SOSORT 2018 Award winner. BMC musculoskeletal disorders. 2019;20(1):319. Epub 2019/07/10. doi: 10.1186/s12891-019-2695-9. PubMed PMID: 31286903; PubMed Central PMCID: PMCPMC6615154.
28. Schreiber S, Parent EC, Khodayari Moez E, Hedden DM, Hill DL, Moreau M, et al. Schroth Physiotherapeutic Scoliosis-Specific Exercises Added to the Standard of Care Lead to Better Cobb Angle Outcomes in Adolescents with Idiopathic Scoliosis - an Assessor and Statistician Blinded Randomized Controlled Trial. PloS one. 2016;11(12):e0168746. Epub 2016/12/30. doi: 10.1371/journal.pone.0168746. PubMed PMID: 28033399; PubMed Central PMCID: PMCPMC5198985.
29. Kwan KYH, Cheng ACS, Koh HY, Chiu AYY, Cheung KMC. Effectiveness of Schroth exercises during bracing in adolescent idiopathic scoliosis: results from a preliminary study-SOSORT Award 2017 Winner. Scoliosis Spinal Disord. 2017;12:32. Epub 2017/10/21. doi: 10.1186/s13013-017-0139-6. PubMed PMID: 29051921; PubMed Central PMCID: PMCPMC5641990.

 Click here to access/download;Figure;Table.docx

	**Group A:** Thoracic Major N=22	**Group B:** Lumbar Major N=18
**Age (mean ± SD)**	13.5±1.8	12.7±1.7
**Gender**		
Female	17 (77.3%)	11 (61.1%)
Male	5 (22.7%)	7 (38.9%)
**Sanders Stage**		
0-3	11	12
4-7	11	6
**Braced**	7 (31.8%)	3 (16.7%)
**Initial Cobb Angle (degrees)**	28.5±8.4	26.8±10.1
**Sanders 0-3 & Cobb ≥30 degrees**	3 (13.6%)	2 (11.1%)
**Sanders 4-7 & Cobb ≥30 degrees**	7 (31.8%)	4 (22.2%)
**Curvature Types**		
Single curve	13 (59.1%)	10 (55.6%)
Double curves	9 (40.9%)	8 (44.4%)
**Exercise Compliance (mean ± SD)**		
1st year (hours/week)	5.5±2.2	5.5±2.1
2nd year (hours/week)	3.0±0.8	4.1±2.2

Table 1

Figure 1 consort chart

Click here to access/download;Figure;CONSORT Flow Diagram_220120.pdf

CONSORT
TRANSPARENT REPORTING of TRIALS

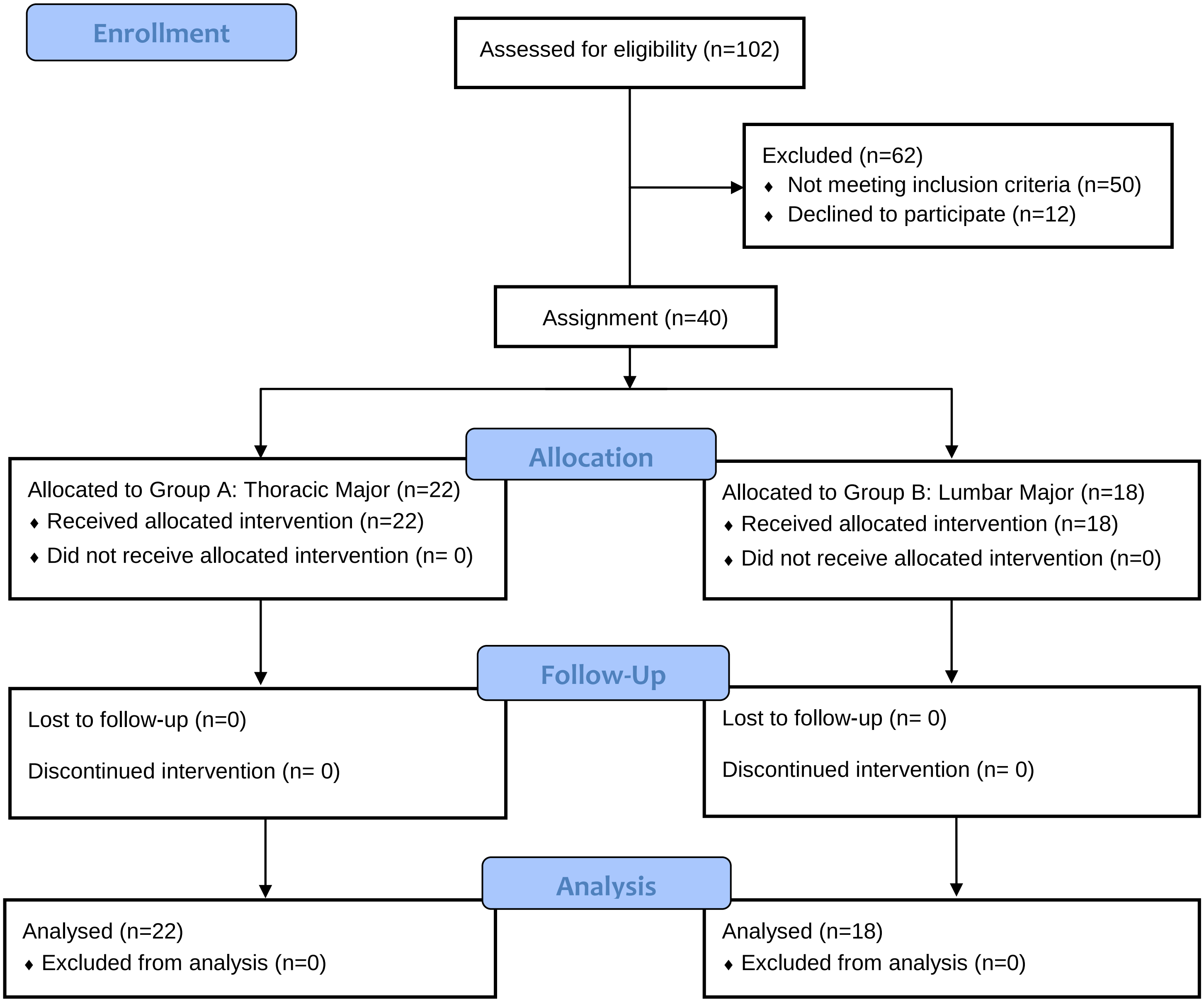

 Click here to access/download;Figure;Figure2-4.docx

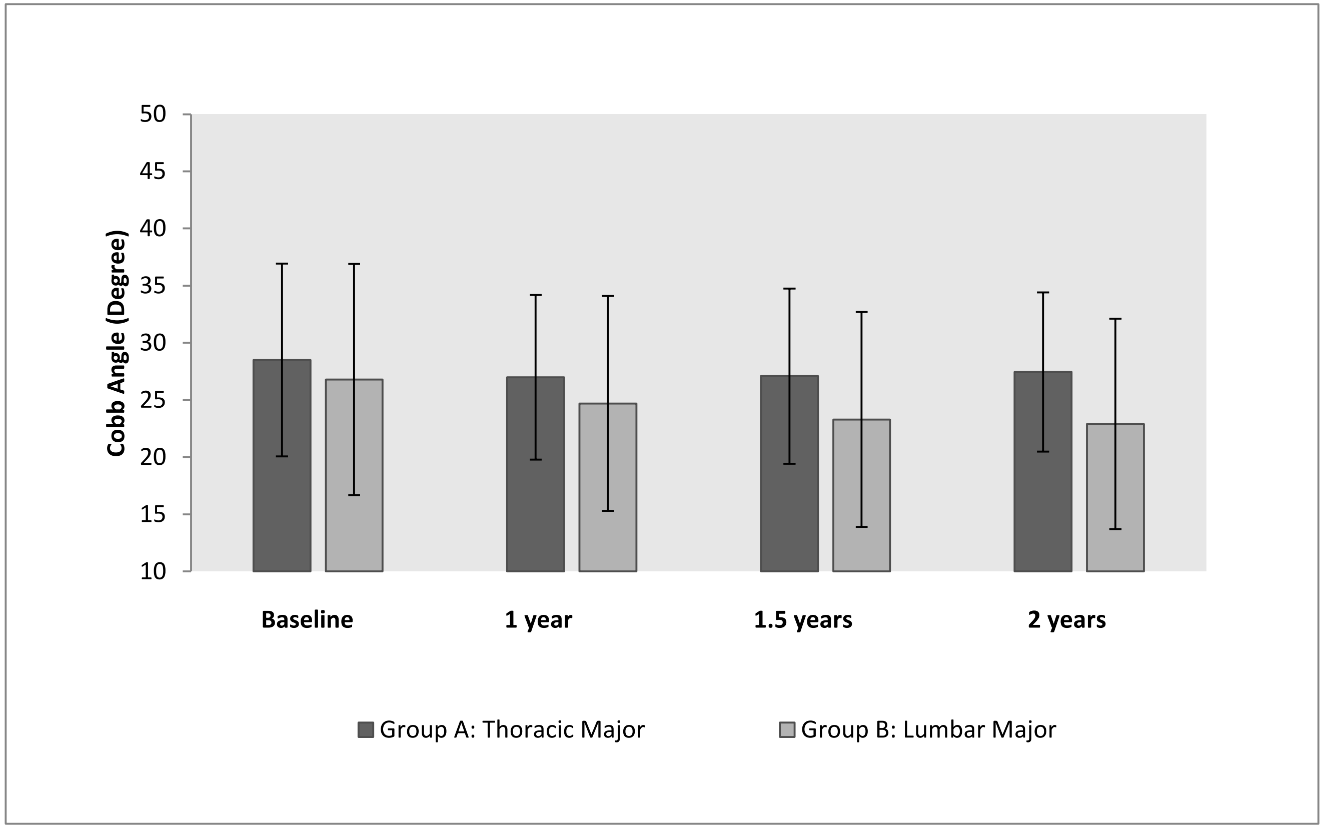

Fig 2

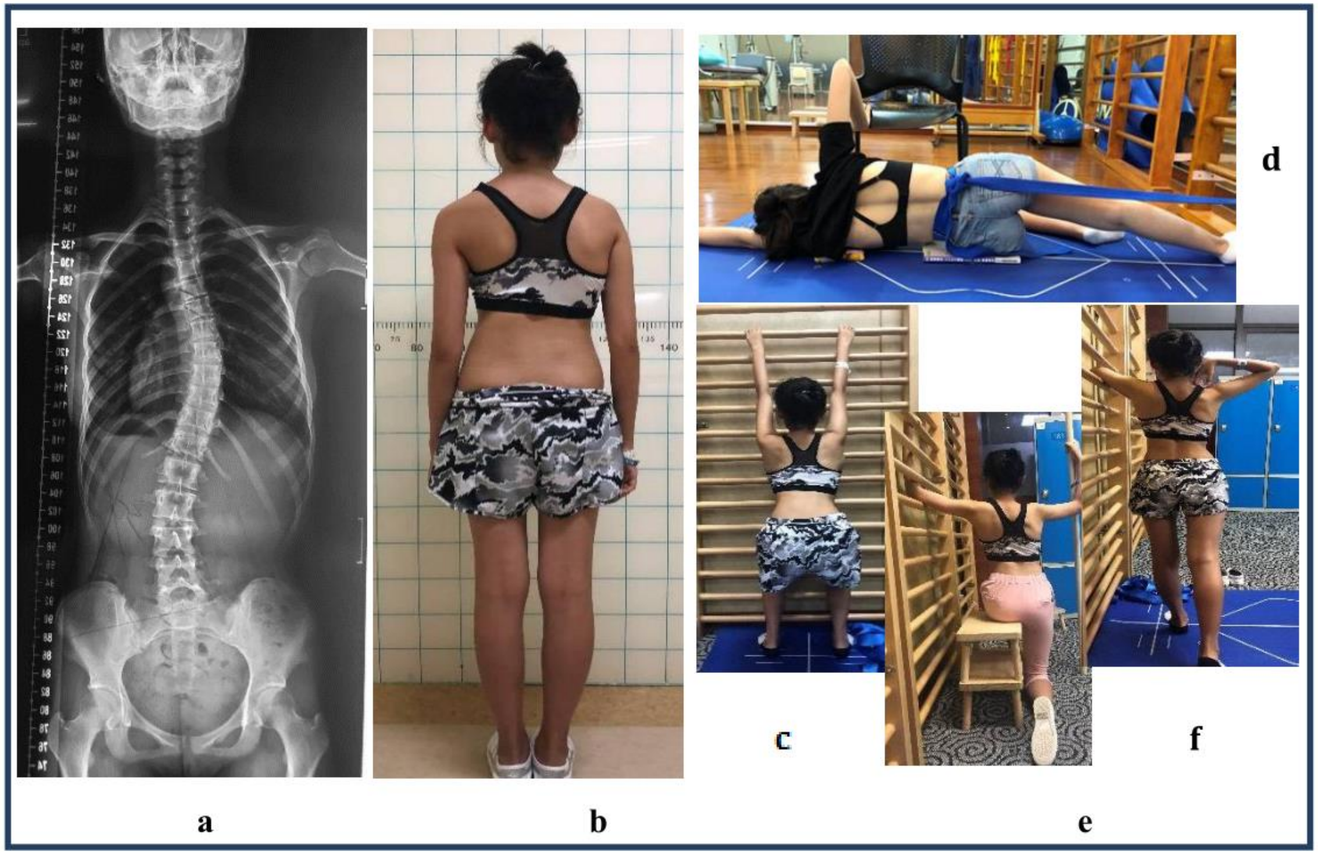

Fig 3

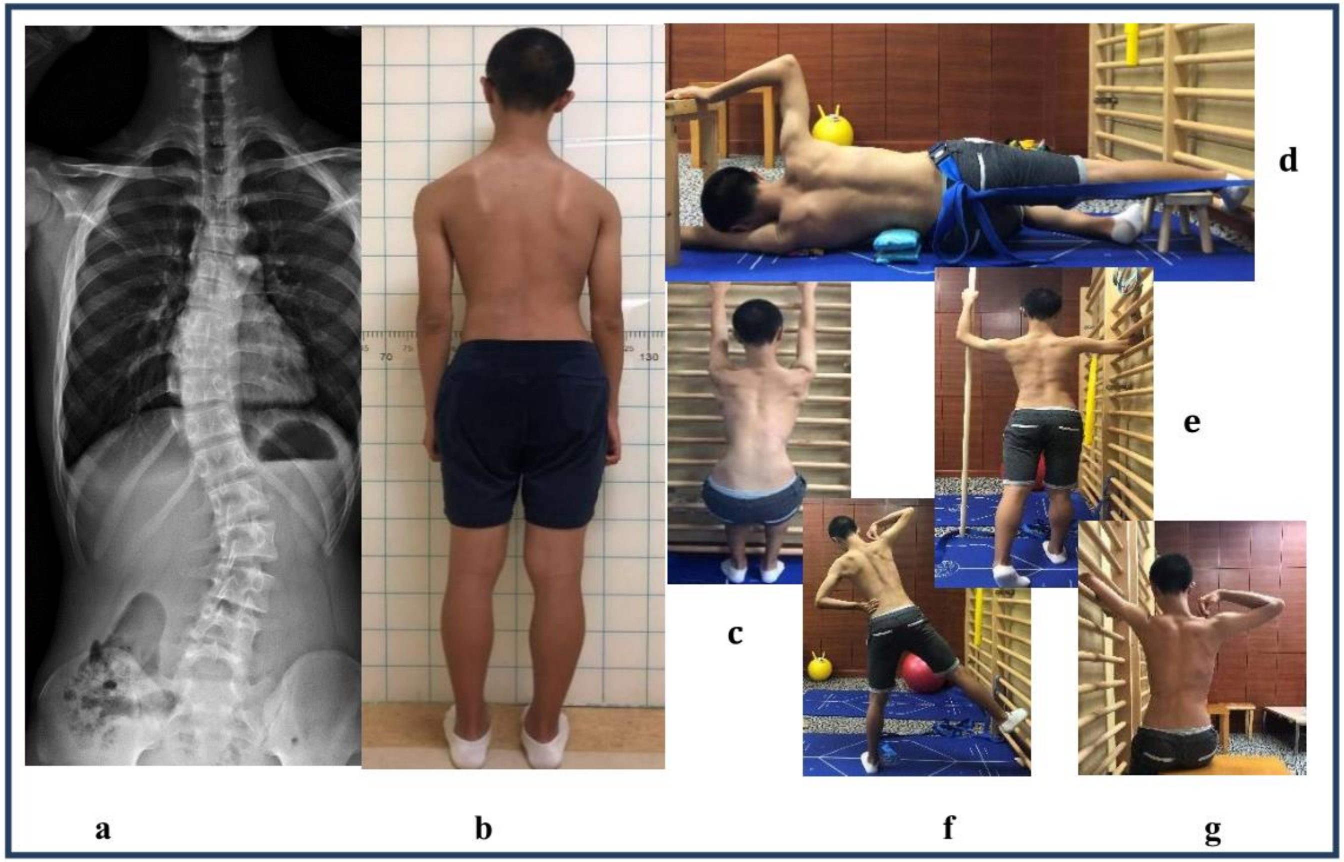

Fig4

Figure 5-6

Click here to access/download;Figure;Figures5-6.docx

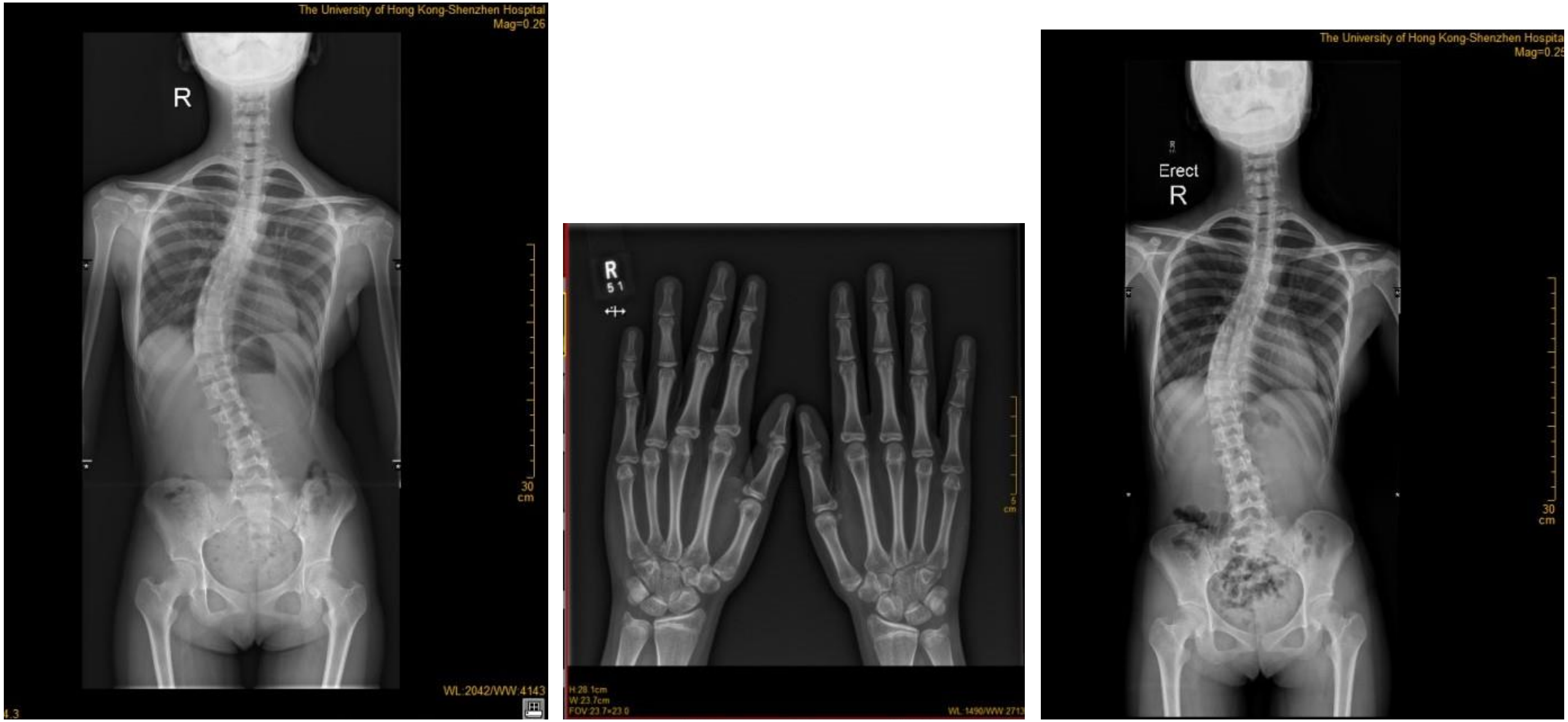

Figure 6: Subject LZY F

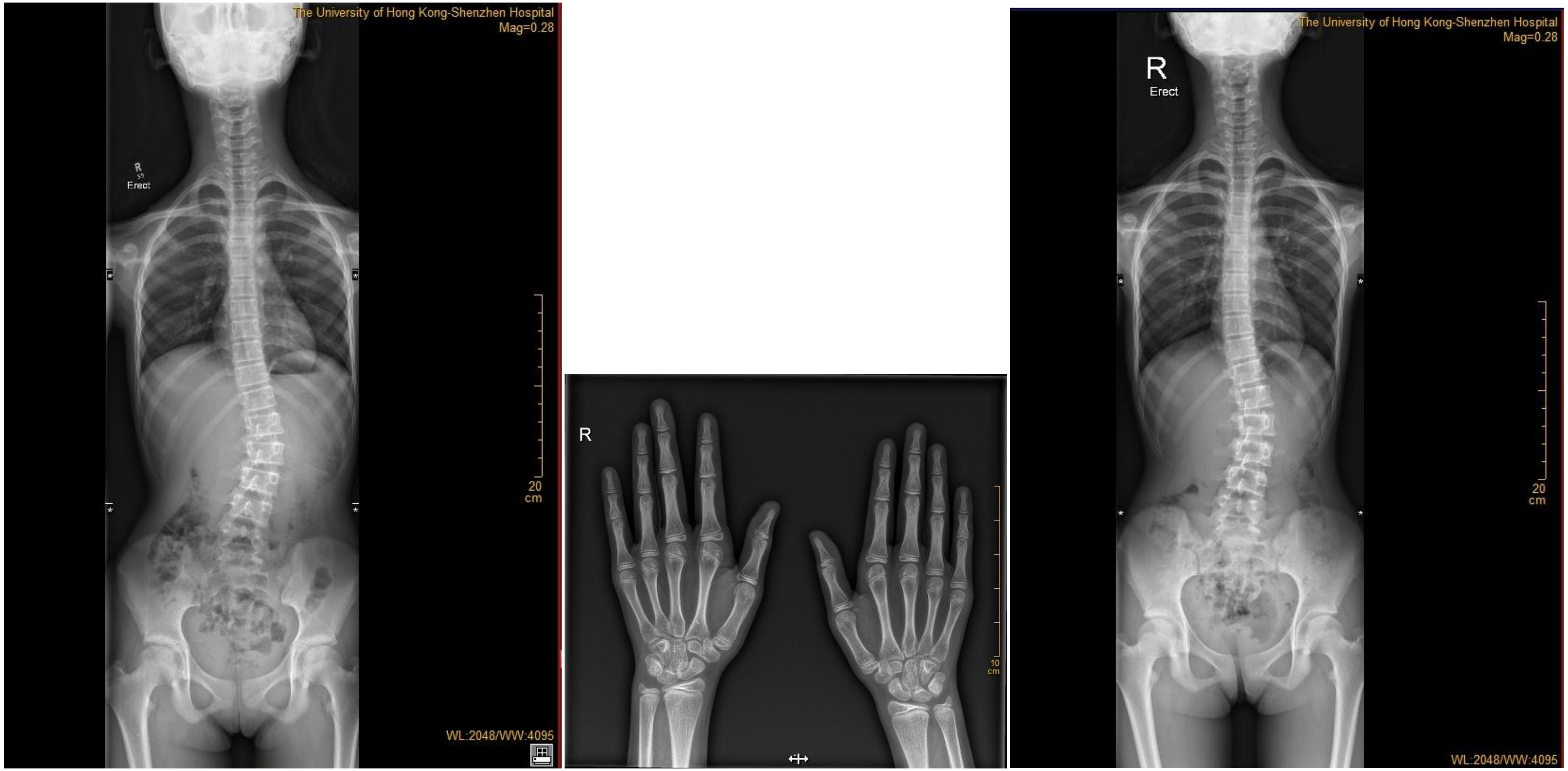

Fig 5: Subject WQY, F

raw data

Click here to access/download
Supporting Information
curvepatternRaw.xlsx

SPSS file

Click here to access/download
Supporting Information
SPSSResults.pdf

IRBaprrovalLetter

Click here to access/download
Supporting Information
renamed_b0e5d.pdf

Click here to access/download
Supporting Information
OriginalProtocol_HardCopy.pdf

Protocol in English

Click here to access/download
Supporting Information
StudyProtocolEnglish.pdf

S1 Trend checklist

Click here to access/download
Supporting Information
Trendchecklist.pdf

Registation information

Click here to access/download

Supporting Information

Registration.pdf

Registration infor

Click here to access/download
Supporting Information
ChiCtr2020012114514487 89.xml
