# Supplementary material for: Does curve pattern impact on the effects of physiotherapeutic scoliosis specific exercises on Cobb angles of participants with adolescent idiopathic scoliosis: A prospective clinical trial with two years follow-up
Source: PLoS One. 2021 Jan 25;16(1):e0245829. doi: 10.1371/journal.pone.0245829 (PMC7833215; doi:10.1371/journal.pone.0245829)
Supplement: Attachment — Submitted filename: PONE-D-19-35759_R1 Manuscript.pdf [file pone.0245829.s008.pdf]

PLOS ONE

Dose curve pattern impact on the effects of physiotherapeutic scoliosis specific exercises on Cobb angles of participants with adolescent idiopathic scoliosis: a prospective clinical trial with two years follow-up

--Manuscript Draft--

**Manuscript Number:**	PONE-D-19-35759R1
**Article Type:**	Clinical Trial
**Full Title:**	Dose curve pattern impact on the effects of physiotherapeutic scoliosis specific exercises on Cobb angles of participants with adolescent idiopathic scoliosis: a prospective clinical trial with two years follow-up
**Short Title:**	Dose curve pattern impact on effects of PSSE in treating AIS
**Corresponding Author:**	Yunli Fan<br>University of Hong Kong<br>Hong Kong, HONG KONG
**Keywords:**	Adolescent Idiopathic Scoliosis; Curve Pattern; Cobb Angle; Physiotherapeutic Scoliosis Specific Exercise; conservative management; Scoliosis Progression; Compliance
**Abstract:**	Background<br>Current clinical evidence suggests that a well-planned physiotherapeutic scoliosis specific exercise (PSSE) program is an effective method for scoliosis regression.<br><br>Objectives<br>We investigated the effect of the curve pattern on Cobb angles with PSSE.<br><br>Methods<br>This was a prospective clinical trial recruiting participant with adolescent idiopathic scoliosis between January and June 2017. Participants were grouped by curve pattern into major thoracic group and major lumbar group. An outpatient-based PSSE program was conducted with the following schedule of intensive exercise: ≥ 1 session of supervised PSSE per month and > 30min of home exercise 5 days/week in the first 6 months, after which exercise frequency was reduced to: 1 session of supervised PSSE every three months and > 30min of home exercise 5 days/week until 2 years after study initiation. Radiographic Cobb angle progressions were identified at the 1-, 1.5- and 2-year follow-ups. One-way mixed repeated measures analyses of variances were performed to examine the differences of Cobb angle between groups over 4 testing time points. The two-tailed significance level was set to 0.05.<br><br>Results<br>In total, 40 participants were recruited, including 22 with major thoracic curves (5 male and 17 female; mean age 13.5±1.8 years; Cobb angle of 18 to 45 degrees) and 18 major lumbar curves (7 male and 11 female; mean age 12.7±1.7 years; Cobb angle of 15 to 48 degrees). Curve regressions, namely the reduction of Cobb angles between 7 to 10 degrees were noted in 9.1% of participants in the major thoracic group; reductions of 6 to 13 degrees were noted in 33.3% of participants in the major lumbar group at the two-year follow-up. Repeated measurements revealed a significant time effect ($F_{2.2, 81.9}=6.9$, $p=0.001$), but no group ($F_{2.2, 81.9}=2.2$, $p=0.1$) or time × group ($F_{1,38}=1.4$, $p=0.2$) effects in reducing Cobb angles were observed after 2 years of PSSE. In addition, 65% of participants (n=26) reached skeletal maturity (Risser sign = 5) and achieved significant reductions of progression risk on the 2-year follow-up (Lonstein and Carlson Risk of progression: 0.45 ± 0.32 vs. 0.13 ± 0.14, $p<0.001$).<br><br>Conclusion<br>This was the first study to investigate the long-term effects of PSSE in reducing Cobb angles on the basis of major curve location. No significant differences in correction were observed between major thoracic and major lumbar curves. However, a regression effect and no curve deterioration were noted in both groups at the 2-year follow-up.

Powered by Editorial Manager® and ProduXion Manager® from Aries Systems Corporation

**Order of Authors:**	Yunli Fan<br>Michael KT TO<br>Eric HK Yeung<br>Jianbin Wu<br>Rong He<br>Zhuoman Xu<br>Ruiwen Zhang<br>Guangshuo Li<br>Kenneth MC Cheung<br>Jason PY Cheung
**Response to Reviewers:**	REPLY TO REVIEWERS<br><br>Re: Ms. No. PONE-D-19-35759 - "Dose Curve Pattern Impact on the Effects of Physiotherapeutic Scoliosis Specific Exercises on Cobb angle of Adolescent Idiopathic Scoliosis: A prospective clinical controlled trial with two years follow-up"<br><br>Dear Editor,<br><br>The authors would like to thank you and the Reviewers for all of your time and effort devoted to the review of our aforementioned manuscript. The Reviewers' comments were indeed extremely insightful and greatly appreciated. As such, the authors would like to take this opportunity to address each and every concern the Reviewers noted in their review of our submission. In addition, where appropriate, we have also revised our manuscript accordingly. Major changes are made in light gray.<br><br>We believe that the Reviewers' comments have helped improve the quality of our manuscript. We hope that you and the Reviewers will find our revised work suitable for publication in PLOS One.<br><br><br><br>Editor<br><br>Comment #1: Journal Requirements:<br>When submitting your revision, we need you to address these additional requirements.<br>1. Please ensure that your manuscript meets PLOS ONE's style requirements, including those for file naming. The PLOS ONE style templates can be found at https://journals.plos.org/plosone/s/file?id=wjVg/PLOSOne_formatting_sample_main_body.pdf and https://journals.plos.org/plosone/s/file?id=ba62/PLOSOne_formatting_sample_title_authors_affiliations.pdf<br><br>Response: We have revised the format accordingly.<br><br><br>Comment #2: 2. Thank you for submitting your clinical trial to PLOS ONE and for providing the name of the registry and the registration number. The information in the registry entry suggests that your trial was registered after patient recruitment began. PLOS ONE strongly encourages authors to register all trials before recruiting the first participant in a study.<br>As per the journal's editorial policy, please include in the Methods section of your paper:<br>Please give confirmation that all related trials are registered by stating: "The authors confirm that all ongoing and related trials for this drug/intervention are registered".<br><br>Response: We have revised this. Please see lines 108-110, page 7 (Materials and methods: study design)

Powered by Editorial Manager® and ProduXion Manager® from Aries Systems Corporation

Comment #3: 3. You indicated that you had ethical approval for your study.
In your Methods section, please ensure you have also stated whether you obtained consent from parents or guardians of the minors included in the study or whether the research ethics committee or IRB specifically waived the need for their consent.

Response: Thank you for the comment. Please see lines 105-107,page 7 (Materials and methods: study design)

Comment #4: 4. We note that you have stated that you will provide repository information for your data at acceptance. Should your manuscript be accepted for publication, we will hold it until you provide the relevant accession numbers or DOIs necessary to access your data. If you wish to make changes to your Data Availability statement, please describe these changes in your cover letter and we will update your Data Availability statement to reflect the information you provide.

Response: No change is needed.

Comment #5: 5. Please amend either the title on the online submission form (via Edit Submission) or the title in the manuscript so that they are identical.

Response: Thank you this has been changed. The title is now: Dose curve pattern impact on the effects of physiotherapeutic scoliosis specific exercises on Cobb angles of participants with adolescent idiopathic scoliosis: a prospective clinical trial with two years follow-up

Comment #6: 6. Please amend your list of authors on the manuscript to ensure that each author is linked to an affiliation. Authors' affiliations should reflect the institution where the work was done (if authors moved subsequently, you can also list the new affiliation stating "current affiliation:…." as necessary).

Response: This has been done.

Comment #7: 7. Please include your table as part of your main manuscript and remove the individual file.
Please note that supplementary tables should be uploaded as separate "supporting information" files.

Response: This has been added.

Comment #8: 8. We note that Figures 3 and 4 include images of patients.
As per the PLOS ONE policy (http://journals.plos.org/plosone/s/submission-guidelines#loc-human-subjects-research) on papers that include identifying, or potentially identifying, information, the individual(s) or parent(s)/guardian(s) must be informed of the terms of the PLOS open-access (CC-BY) license and provide specific permission for publication of these details under the terms of this license. Please download the Consent Form for Publication in a PLOS Journal (http://journals.plos.org/plosone/s/file?id=8ce6/plos-consent-form-english.pdf). The signed consent form should not be submitted with the manuscript, but should be securely filed in the individual's case notes.
Please amend the methods section and ethics statement of the manuscript to explicitly state that the patient/participant has provided consent for publication: "The individual in this manuscript has given written informed consent (as outlined in PLOS consent form) to publish these case details".
If you are unable to obtain consent from the subject of the photograph, you will need to remove the figure and any other textual identifying information or case descriptions for this individual.

Response: Thank you for the comment. We have included the necessary documents. The statement was made on lines 107 to 108, page 7 (Materials and methods: study design).

Comment #9: 9. Please include captions for your Supporting Information files at the end of your manuscript, and update any in-text citations to match accordingly. Please see our Supporting Information guidelines for more information: http://journals.plos.org/plosone/s/supporting-information

Response: These have been included.

Reviewer #1

Comment #1: The article entitled "Dose Curve Pattern Impact on the Effects of Physiotherapeutic Scoliosis Specific Exercises on Cobb angle of Adolescent Idiopathic Scoliosis Subjects: A prospective clinical controlled trial with two years follow-up" has a good topic and matter in the field. However, the methodology of the study needs to be revised. The authors investigate the effects of scoliosis-specific exercise method on different curve patterns (major thoracic and major lumbar) and in long term. The idea is valuable. But the cobb angle range is wide, therefore the inevitable effect of brace wearing for some participants on the results looks unnoticed. Randomization is needed for age, gender, cobb angle, curve pattern, brace wearing, in-brace correction, risk of progression... etc. Before consideration for publication, there may be following suggestions to consider again:
Abstract - In method, information regarding Cobb angle of the subjects are needed. Was curve magnitude similar between thoracic and lumbar curve groups?

Response: We thank the reviewer for the comments and we will try to amend our manuscript accordingly.
The curve magnitude was similar between groups: 28.5 ± 8.4 vs 26.8 ± 10.1, p=0.6.
Please refer to the 1) page 3, abstract: results: lines 29 - 31;
2)results, participants, lines 222 -227;
3)Table 1: line 237

Comment #2: Did patient undergo only exercise intervention or did they use bracing as well?

Response: A total of 10 participants were wearing brace before commencing the PSSE program and having comparable, 37% of correction effects from bracing.
Please refer to the 1) results: participants, lines 231 - 235. 2) Table 1: line 237

Comment #3: The first sentence of the background needs to be revised related with exercise. The statement is associated with brace and surgery.

Response: We thank the reviewer for this suggestion. This has been revised. Please refer to page 2, abstract: background, lines 10-11

Comment #4: What were patients' ages? Was age similar between groups?

Response: Yes, the age was under evenly distributed and was similar between groups. Please refer to the page 3, abstract: results, lines 30 to 31, Table 1 (line 237) and lines 227 to 228 of page 15.

Comment #5: Introduction
- The importance of curve progression risk in conservative treatment was extensively reported. But did you randomize your patients in terms of bone maturity among groups?

Response: Thanks for comment. This study was planned as a case control study. Participants were recruited from one scoliosis clinic and grouped into two groups according to their curve pattern. The progression related data was analyzed at the baseline and correlation analysis was performed to detect any influence of these

factors, including bone maturity, on the reduction of Cobb angles. Please refer to the methodology, study design, lines 119-123, line 125 (S2 Fig. 1: CONSORT flow diagram), line 237 (Table 1) and lines 210 to 213 (statistical analysis).

Comment #6: Did you measure curve flexibility? If so, how did you measure? Because you reported that this study aimed to investigate the long-term therapeutic effect of PSSE on thoracic and lumbar major curves and to determine whether a more flexible lumbar curve would respond better to exercise.

Response: This is an important point by the reviewer. Unfortunately we were unable to quantify spinal flexibility prior to treatment in this study due to patients' radiation concern, therefore, the hypothesis was re-centered on curve patterns. Please see the text at lines 94 to 99 (Introduction), lines 369 to 374 (discussion); lines 433 to 441 (Study limitation).

Comment #7: Methods
- One of your inclusion criteria was having cobb angles: from 10 degrees to 50 degrees. This means that some of your patients had bracing indication. How many of your patients did wear brace in this period? Then how did you differentiate exercise specific effect?

Response: Ten participants (5 were wearing full time brace whereas 5 were wearing only night time brace), were wearing brace and achieved comparable in-brace correction 37%, which explained the effects from bracing and allow this study emphasized on the effect from the PSSE.
Please check the Table 1 (line 237), lines 231 to 235 (results) and lines 349 to 353 (discussion).

Comment #8: Please add detailed explanation about The Rigo Scoliosis Classification system.

Response: Rigo clasification was adopted to rigo cheneau brace fabrication and also BSPT Schroth methods, in which 5 classifications with 8 sub-types were clearly introduced. two important references was cited accordingly, please see references 22 and 23.

Comment #9: You included major thoracic and major lumbar curves. I see from the method that some of them, who had major thoracic curve, had double curves or secondary lumbar curves. This would affect results. We know that single lumbar curves and double curves are more common than other curve pattern in idiopathic scoliosis. Single thoracic curves are rare. Therefore, curve distribution among groups would differ. In table 1, curve pattern was reported as double and single, only. But more information is needed regarding curve pattern distribution.

Response: We thank the reviewer for these comments. This information was revised in:
1.Lines 222 to 227.
2.S2: Fig.1 CONSORT flow chart
3.Table 1.
4.Please also see lines 416-426 of paragraph-study limitation.

Comment #10: For single curve, how many patient had thoracic, how many patient had lumbar? For double curve, how many had primary thoracic? how many had primary lumbar? What was curve pattern distribution of the participants?

Response: Thanks for your suggestion. The additional information has been added to:.
1.Lines 222 to 227.
2.S2: Fig.1 CONSORT flow chart
3.Table 1.
Please also see lines 416-426 of paragraph-study limitation.

Powered by Editorial Manager® and ProduXion Manager® from Aries Systems Corporation

Comment #11: How was curve range for thoracic and lumbar curves? How many vertebrae did curves include? This would affect results. What do you think?

Response: Fantastic comment, we do agree that the range of the curvature would definitely impact on the treatment effects, in terms of that may affect the spinal segmental flexibility. However, curve range should also be correlated with curve pattern, such as a short range should be observed in single lumbar curves whereas a long range should be observed in a thoracolumbar, etc. This idea is good but unfortunately out of scope of this study, the sample size of each curve pattern was small in our study, therefore, extra analysis of range of curvature may cause distraction from our primary outcome. Moreover, it will be too informative and overwhelming to readers of those who are not familiar with scoliosis radiographic classification. However, it will be interesting to study the effects of PSSE on the different radiographic parameters. Please see this amendings to lines 446-447 of paragraph conclusion.

Comment #12: Did you measure axial trunk rotation of the participants? Curve magnitude would affect from trunk rotation parameter as much as Cobb angle?

Response: This is an important parameter that will be studied in a future study. We wanted to mainly focus on the Cobb angle changes in this manuscript. Further investigation of the cosmesis and quality of life will also be performed. Please see lines 447-448 of paragraph Conclusion.

Comment #13: Did you use and patient-reported outcome measures? or Did you assess any clinical parameters such as trunk symmetry and cosmetic deformity?

Response: As discussed in comment #12, these are important parameters to study. We have not included them in this manuscript because we wanted to focus only on curve magnitude changes.

Comment #14: Did any of your patient use spinal brace in this period? When considering wide range of Cobb angle, this looks inevitable. How was the distribution of spinal brace usage among groups? How was in-brace correction for patients who underwent spinal brace intervention? Because if the correction amount differ among groups, this would increase or decrease the exercise effect.

Response: Please check the Table 1 and lines 231 to 235; lines 323 to 326 of paragraph results, discussion: lines 349 to 353; lines 405 to 408.

Comment #15: Male distribution is higher in group B. Do you think this would affect results? Also bone maturity distribution among groups seems to be different with 11 - 11 in group A, and 12-6 in group B. Group B looks to have participant who had low risk of progression.

Response: These are important confounders. Using the logistic correlation analysis, we did not find any significant relationship with gender and maturity.
Please refer to lines 323 to 326, Table 1.

Comment #16: In table one, minimum and maximum values are needed.

Response: Range was reported in the abstract; Mean ± SD and 95% CI have been added in the Table 1.

Comment #17: Did authors use any randomization method for age, gender, Cobb angle, curve progression risk (bone maturation)? If not, how would it be possible to make exact comparison of two different curve pattern groups (thoracic and lumbar)

Response: It was convenient sampling with a curve pattern controlled study, the assumption of normality was calculated before commencing the study and analysis

Powered by Editorial Manager® and ProduXion Manager® from Aries Systems Corporation

was conducted for baseline data between groups. Logistic regression analysis was adopted to detect any correlation of variables that differed on baseline between groups and treatment effect, which was reported on lines 210 to 213; lines 323 to 326, curve distribution can be found in S2 Fig 1 and table 1.

Comment #18: I am sending my suggestions until here. Discussion is also needed to be revised according to methodology.

Response: Thanks for your valuable suggestions and hope you will find the updated discussion interesting.

Reviewer #2

Comment #1: The curve title should be corrected to: Does Curve Pattern Impact the Effects of Physiotherapeutic Scoliosis Specific Exercises on Cobb angles Participants with Adolescent Idiopathic Scoliosis: A prospective clinical controlled trial with two years follow-up.

Response: We thank the reviewer for this suggestion and this has been revised.

Comment #2: It is best to refer to participants or persons with a diagnosis rather than subjects. Throughout the text please replace patients by participants or persons.

Response: These have been corrected.

Comment #3: ABSTRACT Please specify details of the intensive frequency and reduce frequency. (number of visits per week or month and their duration of exercising at minimum). Specify if a home program was performed.

Response: This has been done. Please refer to the page 2, abstract, methods, lines 19 to 23.

Comment #4: If the study is controlled. I assumed there are two groups. Specify what the control group did during the trial in the abstract.

Response: This was planned as a clinical, curve pattern controlled study. Please refer to the page 2, abstract, methods, lines 17 to 19. S2 Fig 1 Consort flow diagram.

Comment #5: Then the analysis should likely be a mixed model ANOVA with a between subject group factor and a within subject (repeated measures) time factor. There should not be multiple paired t-tests if you choose a relevant pairwise comparison over time within curve patterns. Using separate t-tests would increase your risk of type one error.

Response: We thank the reviewer for this astute comment. The analyses have been corrected according to your valuable suggestions, please see pages 2-3: abstract, method (abstract): lines 24 to 26; statistics lines (main text): 196 to 213; results (abstracts): line 34-39; results (main text): lines 259 to 291; Tables 2,3.

Comment #6: There are numerous English issues. I will not point all of them as English is a second language for me too. Please have the paper reviewed by an English editor. EG always use major thoracic curve and major lumbar curve.

Response: Thank you for the comments. This has been amended accordingly.

Comment #7: Specify the recruitment setting and method for the participants.

Powered by Editorial Manager® and ProduXion Manager® from Aries Systems Corporation

Response: This has been done. Please see page 7, methods, study design, lines 103 to 105; lines 110 to 123 and S2 (Fig. 1: CONSORT flow diagram).

Comment #8: Beyond stating no difference in the curve magnitude between groups please report the curve means and SD and how many patients if any were braced. If none wore a brace, specify.

Response: Ten participants (5 were wearing full time brace whereas 5 were wearing only night time brace), were wearing brace and achieved comparable in-brace correction 37%, which explained the effects from bracing and allow this study emphasized on the effect from the PSSE.
Please check lines 132 to 135 of page 9, the table 1 and results, lines 231 to 235; discussion, lines 348 to 351.
.

Comment #9: Spell out d on first use for D-value.

Response: The D-value stands for the difference value of Cobb angle over three testing times with the initial Cobb angle, to detect the regression, stabilization and deterioration between groups. This has been reiterated in lines 203-206 and table 2.

Comment #10: Since we don't know how you calculated d, we don't know in the abstract if the change over 1 year is a improvement or deterioration in Cobb angles in each group. The sentence with the change in COBB for the specific intervals should specify which group had which results. There should be multiple F statistics and p-values with your design: one for the interaction between groups and time, one for the main effect of group and one for the main effect of time.)

Response: We are sorry for this confusion and the analysis has been clarified. Please see the revised abstract, methods (lines 203-206), results (lines 259 to 365) and Table 2.

Comment #11: The value for the regressions in group B do not seem to map to any of the values in the sentence prior. How were these group be reductions calculated.

Response: Regression was defined as a decrease of Cobb angle ≥ 6 degrees, the percentage value in each group was reported in the results session, in addition, raw Cobb angle was also analyzed using the repeated measures, please refer to the outcome measurement (lines 184 to 186) and results (lines 259-265; lines 272 - 291). All results, including mean, SD, 95% CI of the D-value and of the raw Cobb angles, were reiterated and summarized in the Table 2 and Table 3, respectively.

Comment #12: Report data on the compliance and drop outs with the exercise program. Did all 40 participants attend all follow-up. If yes specify this is a per protocol analysis and report how many had started and were not included in the analyses.

Response: There were no dropouts through the whole studies. Per-protocol analysis was specified accordingly. Please see lines 253 - 257; S2: Fig.1 CONSORT flow diagram

Comment #13: The conclusion is inaccurate: This was the first study to investigate whether PSSE can lead to curve regression based on location of the major curve. The following study examined the effect of curve type in a multivariate analysis of the short-term effects of Schroth on Cobb angles. Schroth Physiotherapeutic Scoliosis-Specific Exercises Added to the Standard of Care Lead to Better Cobb Angle Outcomes in Adolescents with Idiopathic Scoliosis - an Assessor and Statistician Blinded Randomized Controlled Trial. Schreiber S, Parent EC, Khodayari Moez E, Hedden DM,

Powered by Editorial Manager® and ProduXion Manager® from Aries Systems Corporation

Hill DL, Moreau M, Lou E, Watkins EM, Southon SC.
PLoS One. 2016 Dec 29;11(12):e0168746. doi: 10.1371/journal.pone.0168746. eCollection 2016.

Response: Thanks for you pointing this out, the conclusion and discussion has been revised to “an extension of previous studies”.

Comment #14: Introduction
In the intro where you state the following about the reviews it may be important to review each paper in those reviews for discussions of the effects of curve type. The reviews did not address this question but some of the original reviewed trials did. however, in these studies, the relationship of curve location with correction effects was not clearly discussed, and there was only a short-term followup. There may be limitations to those as short follow-ups which would still justify your work that you could highlight.

Response: Thanks for this brilliant suggestion, the introduction was revised and followed your suggestions, please refer to the lines 77 to 85 (Introduction).

Comment #15: The following intro statement is also overreaching. Schreiber et al above did include baseline Cobb angle in their study of the effect of Schroth. There maybe limitations to this understanding but it is not completely unknown. You stated “the influence of curve magnitude on exercise outcomes is unknown.”

Response: We apologize for the oversight. This has been corrected.

Comment #16: The objective as stated focuses on curve magnitude and flexibility rather than relation between outcomes and curve types. Your focus seems to be on curve type in the abstract. Please re-center the objective on this topic.

Response: We thank the reviewer for this comment. It has been re-centered. Please see lines 94 - 98.

Comment #17: Specify your recruitment methods and setting. Were all consecutive eligible participants invited? Only referrals from some DRs…

Response: patients were recruited from a scoliosis clinic, therefore, all consecutive eligible participants were invited and decision was made according to the inclusion/exclusion criteria. Please see lines 111 to 123 and S2 Fig 1.

Comment #18: Please add a justification to the exclusion of hypermobility. This has not been recommended by SOSORT and has not been done in other study. Maybe, if possible, report how many patients were excluded on this basis.

Response: There were 18 subjects were detected with hypermobility, in form of Beighton score >4, who were excluded from this study. In addition, a total of sixty-two subjects were excluded after eligibility assessment, the exclusion reasons were specified on the lines 115-119; lines 218 to 222 (Results: participants) and S2 (Fig.1 CONSORT flow diagram). One previous study reported hypermobility as a dominant presence in the single curve type and should be taken into account during physiotherapy. Participants with hypermobility were excluded to control/limit the systematic error in this study, the related paper was cited in accordingly. Please see reference 21.

Comment #19: Please specify if patients could have been prescribed a brace at baseline with the exercises or not in the selection criteria.

Response: Yes, they could have. Please see line 115 (exclusion criteria: previous

Powered by Editorial Manager® and ProduXion Manager® from Aries Systems Corporation

treatment) and lines 132-135 (paragraph: study interventions).

Comment #20: From a Asklepios (German) Schroth trained perspective grouping N3N4 with 3C is not consistent with only grouping thoracic curves. Please acknowledge as a limitation that the N3N4 group likely had both thoracic and lumbar curves and may have needed different treatment than the 3C curves. For example the Chest twister exercise is not indicated for N3N4. Similarly. The type 4C curves do have a lumbar dominance but also have a thoracic curves. What did you do with the pure lumbar or thoracolumbar curves? Specify if you had any in your sample.

Response: N3N4, BSPT approach, is a bit different from the German approach, it mainly focuses on correction for thoracic curve, like gentle chest twisting was applicable but should perform without changing the balance and without over correction. By contrast, the double curve in the German approach, takes the lumbar curvature in priority, therefore using pelvic strategy more than chest twisting, etc. The principles are mostly same but the practical metric is a bit different. We did not explain too much or try to compare the two methods in this manuscript, we were trying to prove the effects of Schroth with Cobb correction instead of centering on how did we position patients. Explaining this practical metric is valuable but may be overwhelming for readers who are not familiar with Schroth, moreover, the dynamic feedback of the spine with exercise is unclear yet. Therefore, after all consideration, we did not include this explanation in our manuscript.

Comment #21: Please specify the certification of the Schroth therapists BSPTS -rigo, or Asklepios or ISST or Weiss…

Response: BSPTS approach, we firstly registered as BSPTS in 2016, then registered Asklepios in 2018, therefore, mainly BSPTS concept in this study. Please see lines 129.

Comment #22: When were participants asked to complete the simple home exercise compliance questionnaire (Weekly, monthly, once at then of the study…)

Response: Monthly. Please see lines 137-143.

Comment #23: In listing exercises could the figure order be arranged to match the order of presentation in the text.

Response: Sorry for the confusion. This has been amended.

Comment #24: There is a variation of the muscle cylinder for thoracic curves. Specify why it was not used.

Response: Please see lines 153-161. This was not considered due to the pain at the knee joint.

Comment #25: Describe how the those was chosen. Did the patient do the same exercise throughout the program? Acknowledge as a limitation why you did not use dynamic exercises such as walking or …

Response: We thank the reviewer for this astute comment. Please see lines 158-161.

Comment #26: Throughout the paper: I recommend avoiding the labels A and B for the groups and clearly stating thoracic vs Lumbar groups.

Response: We thank the reviewer for this comment. It has been changed.

Powered by Editorial Manager® and ProduXion Manager® from Aries Systems Corporation

Comment #27: Figure 5 and 6 Please add a justification for why these participants were not braced in addition to doing the exercises.

Response: Two patients were mostly considering about the cosmetic issue; especially one girl was at late Risser stage (Fig 5) and bracing was not compulsory for her, then this girl tried exercise for a half year and resulted with a significant correction, then she had never considered bracing again.

Comment #28: Table 1. Add units for age.

Response: Added. Table 1

Comment #29: Replace gender by Sex. I doubt you documented gender.

Response: This has been done.

Comment #30: Why not report count and percentage as you did for sex instead for Sanders stage.

Response: The Sanders stage has been replaced by Risser, because of the progression risk value in terms of the Lonstein and Carlson score, that needs the Risser for calculation. Please see the S4 File: raw data set and Table 1.

Comment #31: Can you specify brace types. This is a notable imbalance and surprisingly more braced in thoracic group?

Response: Thanks for the comment. Please see lines 231 - 233 on page 9. All (n=10) were wearing Cheneau brace (Table 1).

Comment #32: For future meta-analysis purpose it may be valuable to still measure thoracic and lumbar curves in both groups.

Response: We agree with the suggestion. Please see Table 1, S2 (Fig. 1: CONSORT flow Chart) and S4 File: study data.

Comment #33: Rather than or in addition to, please report the number of N3N4 vs 4C per group or add a note to each column showing the thoracic double curves were all N3N4 and the Lumbar were all 4C.

Response: This has been added. Please see S2 (Fig. 1: CONSORT flow Chart) and the Table 1.

Comment #34: Specify in the methods if the classification was clinical only or informed by radiographs also.

Response: Radiographic, BSPTS is using this classification system for bracing too, therefore the X-ray is in priority for classification and the clinical presentation was only for clinical reference.
Please see lines 119 to 120 and S2 (Fig.1 CONSORT flow diagram.)

Comment #35: Can you report if all thoracic curves were right side and all lumbar were left sides. If not please report the distribution.

Response: This has been clarified in lines 222-227, (S2 Fig.1 Consort flow diagram) and Table 1.

Powered by Editorial Manager® and ProduXion Manager® from Aries Systems Corporation

Comment #36: Figure 2 could present statistical significance of the comparison of the values as well with symbols.
Specify what the error bar represent (standard deviations or standard errors).

Response: The new figure 1 has been amended. The previous Figure 2 was replaced by the table 3 shows raw data of Cobb angles over four testing time points, in which mean +/- SD, 95% CI and range were all presented, which may be of value for future meta-analysis.

Comment #37: Figure 3. It is unfortunate that the patient has a wide racer back bra which hides much of the postural defect in thoracic spine. The semi-hanging picture has allowed too much hypokyphosis. Would you have a semi-hanging example where the sagittal kyphosis is better maintained. The aspect ratio of the photo f seems off (too wide not tall enough).
Can you add the left right marker to the radiograph. It show right thoracic to the right of the image.

Response: A new picture has been uploaded. Unfortunately, we cannot find a better photo as patients cannot come to our center due to COVID. Therefore, I used her another pic for demonstration, please check see whether it is acceptable.

Comment #38: Figure 4. Can you add the left right marker to the radiograph. This one shows the right thoracic curve on the left of the image. Once again in C the patient shows hypokyphosis and this time has lost physiological lordosis. Can you find a demonstration of semi-hanging in this group with better sagittal profile. For G, can you have an example with proper head alignment.

Response: This has been amended.

Comment #39: Figure 5 and 6 were presented with figure 6 first. No need to use initials in the figures. Label as example of a thoracic vs lumbar participants. Could you draw the Cobb measurements on the images? Could the scaling of the two spine images in fig 6 be made more similar?

Response: This has been added.

Comment #40: The dataset uploaded should also include a data dictionary or a legend. What values are coded 1 and 0 … Define cut point 30 deg. Is the data incomplete. What appears in columns V to AO??

Response: Done, the file has been amended. The other columns were including other data such as ATR, SRS-22 etc. We have deleted them as the data on radiographic measurements were used in this study. Please see S4 File: study data.

Comment #41: Specify in the selection criteria what was the criteria for brace prescription and brace termination and how you monitored brace compliance. Detail the kinds of brace that were prepared and possibly the targeted or achieved in brace correction if available.

Response: The SRS bracing criteria was used by doctors to prescribe brace treatment, see lines 132 to 135, and the reference was cited accordingly. Brace termination was defined with skeletal maturity, and bracing compliance was monitored using a monthly self-reported adherence checklist, see lines 139 to 143. In-brace correction and brace type were clarified on lines 233 to 235 and Table 1.

Comment #42: Can you detail the positioning instructions for the participants during the radiographs.

Powered by Editorial Manager® and ProduXion Manager® from Aries Systems Corporation

In clinical trials the study is not powered to compare group characteristics at baseline. It does not mean much to do statistical comparisons of those. Key is whether the estimate appear to present clinical differences before deciding whether to control for those.

Response: amended accordingly. Please see line 144 to 153. Picture legend of Fig. 2 and Fig. 3.

The comparison of the baseline data was to show the assumption of normality, if the baseline was evenly distributed and which can power our study quality. However, I fully agree with you that a clinical trial should look more on the factors that may affect results, but we have to do so according to suggestions from other reviews. In this study, with no deterioration after 2 years, the logistic regression was conducted to analyze the relationship of age, sex, brace etc. and treatment result, which further explained the influence of aforementioned factors on the Cobb reduction. See lines 323 to 326 (Results).

Comment #43: Clarify how the d-values were computed. Why not describe it as a mixed model anova with a between group factor and a time factor and use appropriate pairwise characteristics. It appears to be what you did given the output provided. Can you specify which pairwise was requested? LSD? The use of multiple t-tests to detect regression increases the type one error chances.

Response: LSD was used. We have corrected the analyses appropriately. We thank the reviewer for these valuable suggestions.

Comment #44: Results:
Subjects. I disagree that groups were sufficiently similar for bracing.

Response: Our comment was based on the results of the X2 test which showed no statistical difference. Nevertheless, logistic regression was performed to test whether bracing was an impact on the results. See lines 323 to 326 (Results).

Comment #45: From the selection criteria I believed no participant could have started any scoliosis treatment before enrollment. Here I learn that bracing could have been started prior to PSSE. This should be clear from the patient selection criteria.

Response: We thank for reviewer for this valuable suggestion. The patients started with either bracing or exercise would be excluded in this study, please see S2 (Fig. 1) and lines 132 - 135.

Comment #46: Can you report brace compliance in both groups?

Response: Please see line 233 to 235 and Table 1, because of there was no correlation of bracing with regression effects, the bracing hour, bracing compliance from those 10 participants were not further discussed.

Comment #47: Why only report the percent radiograph refused at 6mth into PSSE. Why not also for the other time points.

Response: All participants completed radiographic assessment on the baseline, 1-year, 1.5-year and 2-year follow-up, therefore per-protocol analysis was performed. Please see lines 256 – 257. lines 272 to 273 and S2 Fig.1.

Comment #48: Specify if the following statement applies only to group averages or also to patient individual data: There was no deterioration of the major curvature in either group at the 2-year follow-up.

Response: It applies to both average and individual data. No deterioration was noted for either group. Please see lines 259 to 260 and Table 2.

Powered by Editorial Manager® and ProduXion Manager® from Aries Systems Corporation

Comment #49: Where you report the F and P-values you need three F values. The key one is for the interaction between group and time, then you also need main effects on groups and on time. (your output show all were not significant.

Response: The analysis was re-performed by a statistician who was blinded to this study. We thank the reviewer for this important comment. Three F values of comparisons in D-value of Cobb and raw data of Cobb, respectively, were clarified and reported. See lines 263 to 265, table 2,3,4 and lines 275 – 277.

Comment #50: Report the Sanders secondary analysis as a new paragraph. Announce this interest in objective section as a secondary objective.

Response: The Sanders staging has been replaced by Risser as we needed the Risser for evaluation of progression risk.

Comment #51: If the interaction effect is not significant it is not good statistical practice to explore pairwise within groups with t-tests. IF you had set your mixed method anova with Cobb at baseline, 1 year, 1.5 yrs and 2 yrs you may have detected this effect already. Running a different approach with t-test exposes you to a higher risk of type 1 errors.

Response: Fully agree, beside of analysis of D-value of Cobb reduction over three testing times, we re-performed analysis on the Raw Cobb angle over four testing timepoints which suggested a significant time effects in reducing Cobb angles (repeated one way anova).

Comment #52: It would be possible to use a chi-square analysis to compare the distribution of the improved, stabilized and deteriorated in each group statistically in addition to just report the results. A statistician may be able to determine if the three time points could be compared in a single analysis for these proportions to protect against a type one error.

Response: We have proceeded to the reviewer's suggestions. Unfortunately, the X2 analysis also showed no significance, therefore, we further analyzed the relationship of influencing factors, including age, brace, sex etc and the regression effects using logistic regression analysis. See lines 210 to 213 and lines 323 to 326.

Comment #53: See above. The following opening statement of the discussion is overreaching: This is the first study to investigate the outcomes of different curve types undergoing PSSE treatment for AIS.

Response: The statement has been corrected. Please see lines 341 -342 (discussion).

Comment #54: You cannot state the following as all your groups received exercises. You did not show superiority to an alternative. And you did not discuss historical controls. Therefore, our results further revealed the superiority of PSSE programs in the stabilization and regression of scoliosis.

Response: We thank the reviewer for the sentiments.

Comment #55: In the discussion you state: This met our original study hypothesis that lumbar major curves are more flexible. I am not sure this is the only possible physiological explanation. You did not measure flexibility per say. Please justify your statement and address possible alternatives as well. Discuss limitations of not having measured flexibility directly.

Response: We thank the reviewer for these suggestions. Please see lines 369-389 and lines 433 – 441.

Powered by Editorial Manager® and ProduXion Manager® from Aries Systems Corporation

Comment #56: P13. Compare your results of lumbar effects being better to those of Schreiber et al. I believe they did not find the same curve pattern as the one with the best response. Could it be that some benefits for your thoracic group may have been missed due to not using the chest twister and prone/supine exercises

Response: It is possible, we thank the reviewer for this valuable discussion point. I remember that there were conference papers, presented in SOSORT 2019 in SF, there were evidences showing that the spine responded better during exercise in prone. However, to our knowledge, this evidence was not published yet and unfortunately our study was initiated before 2019. Positions were designed to cope with patients' daily activity as much as possible in this study. In Schreiber's study, they used chest twist for thoracic curve, however, their results also showed the 3CP was significantly correlated with the largest Cobb after treatment, which was explained by the proven higher progression risk was shown in thoracic type. Therefore, it will be not suitable to compare our study with theirs on this point, because they also found the less effects with 3CP. Therefore, we alternatively stated that our study extended their results for the long term effect (lines 341-344) Moreover, for the potential variation caused by varied exercises, we further explained in lines 372-389, which was more emphasizing on the spinal biomechanics instead of varied positioning during exercise.

Comment #57: Please discuss the limitation of self-reporting compliance and possible recall issues depending on when your participants completed the compliance questionnaires.

Response: Participant need to take a note and sign on his checklist on the day when they performed exercise, then hand the checklist to physiotherapist at their next visit. See lines 139 – 143, recall effects were discussed on lines 405 - 408.

Comment #58: Limitations. There are more than 4 patterns in RIGO classification. Type 1 and type 2 were not discussed here.

Response: Information added. See lines 416 to 426.

Comment #59: Please discuss not monitoring co-interventions such as manipulations, massage, other fitness activities or self-used of off the shelve bracing.

Response: The participants would agree with only PSSE but no alternative exercise before sign the study consent form, but we agree that participants are likely to take another therapy without informing us. Commencing any other treatment were excluded from this study, please see line 115 and S2 Figure.

Comment #60: Could you review how many patients in each group experience a change of curve type over time and report which changes occurred.

Response: There were no curve pattern changed in this group of participants. But curve pattern might be changed with long-term exercise and should be identified. Please see Table 4

Comment #61: Discuss the imbalanced in the number of braced participant and possible brace compliance effects on results.

Response: Only 10 subjects had brace treatment and half of them only used night time brace. The logistic regression showed bracing did not affect our outcome, therefore, it maybe not very valuable to go in details of this analysis due to our small sample size. Please see lines 405 – 408.

Comment #62: Discuss whether after 2 years of follow-up these participants had reached skeletal maturity. How many had reached discharge point or were sufficiently far after peak growth velocity to deem the results final or not.

Response: Fantastic point. Table 4 was edited to this purpose.

Comment #63: The SRS-SOSORT recommendations propose a number of analysis reporting guidelines. Could you report Risser signs in your dataset or table 1. Could you report number of patients with curve over 30 at each time point. I believe none exceeded surgery threshold of 45 or 50 but this could be specified.

Response: Table 1 has been amended and see an updated table 4.

Comment #64: The discussion could compare how your follow-up length and results compared to other Schroth studies. Are your results better than others or similar. Was the progression risk of your cohort similar or worse than others.

Response: Fantastic point, related result and discussion were addressed accordingly. See lines 339 to 344.

Comment #65: Please add a paragraph about whether there is a risk of overtreatment in this cohort. Could some of the patients have been left alone and avoid the burden of treatment. I can see that some would argue that for the fact that these patients possibly had no risk of progressing to bracing or surgery. However you showed some notable regression. This may appeal to some participants with small curves at low risk of progression. Still the possible overtreatment issue should be discussed.

Response: We thank the reviewer for this important point. The discussion was amended accordingly. See lines 353 to 356.

Comment #66: An important limitation in your study is that you may have been underpowered to detect difference between groups. It would be important to discuss how big a different in effects between curve types may be clinically important. This may be difficult to determine but a discussion of this topic should be presented.

Response: We agree. This has been added to line 417 to 425 and lines 446 to 449.

Comment #67: I would recommend against including the following in the discussion as you did not measure flexibility and did not study relation of effects of PSSE with baseline curve magnitude per say. However, further studies are necessary to address the correlation between spinal flexibility and the correction effects of PSSE at different curve magnitudes. IF you wish to keep then please move earlier in the discussion as suggestions for future research.

Response: We thank the reviewer for this comment. Please see lines 446 to 449. We re-centered our discussion point in the introduction and discussion, respectively.

Comment #68: I have annotated a few elements of the paper in an uploaded copy.

Response: We thank the reviewer for these comments. Thanks for nice editing, the format was amended accordingly.

Reviewer #3

Comment #1: The objective of this study is to investigate the comparative effectiveness of the PSSE correction effect on the Cobb angle between the thoracic and lumbar curves in AIS subjects. The authors considered a prospective clinical controlled study. While the study objectives sound interesting, a number of shotcomings were observed, in regards to abiding by the CONSORT guidelines for conducting and reporting results of high-quality randomized controlled trials (RCTs).

Response: We thank the reviewer for these comments.

Comment #2: 1. Abstract: The authors state results as: "A significant Cobb angle reduction was observed...", without any statement of p-values, estimated effect size, and its precision as confidence intervals, or CIs (say, 95\%). This can appear confusing, and half-baked to a reader. Check CONSORT checklist for Abstracts reporting of RCTs, and rewrite the Abstract following guidelines.

Response: This was not an RCT study, therefore no needs for randomization etc, nonetheless, the problems in the abstract was recognized and amended.

Comment #3: 2. Methods:
Methods reporting appeared very messy. An orderly manner is suggested, following CONSORT guidelines, without repeating information, such as Trial Design, Participant Eligibility Crtieria and settings, Interventions, Outcomes, sample size/power considerations, Interim analysis and stopping rules, Randomization (details on random number generation, allocation concealment, implementation), Blinding issues, etc. The authors are advised to create separate subsections for each of the possible topics (whichever necessary), and that way produce a very clear writeup.

Response: Thanks for the valuable suggestions. We have revised the formatting.

Comment #4: (a) For instance, the randomization and allocation concealment should be made very clear; the trial staff recruiting patients should not have the randomization list. Randomization should be prepared by the trial statistician, and he/she would not participate in the recruiting. I am confused; was randomization not done during the "Allocation" phase in your CONSORT diagram?

Response: This was not an RCT, it was a CCT hence no randomization was required. Instead we controlled the curve pattern for the study.

Comment #5: (b) I am surprized to see no statement on sample size/power in a manuscript proposing a (clinical) controlled trial. This is really the key here!

Response: We thank the reviewer for the comment. The sample size estimation is in lines 192-196.

Comment #6: (c) t-tests were used for assessing group differences for continuous variables (under the assumption of Normality). What if underlying normality assumptions are violated? Why not non-parametric (robust) tests were proposed?

Response: Related clarification was addressed; normality was measured before commencing the analysis. Please see lines 195 - 213.

Comment #7: (d) Similarly, the one-way repeated measures ANOVA may also be replaced/presented by a nonparametric Friedman-type test. I mean, justification is needed for the underlying Normality assumptions.

Response: These have been clarified in the statistical analysis section.

Comment #8: 3. Results & Conclusions:
(a) The authors should check that any statement of significance should be followed by a p-value in the entire Results section.

Response: We thank the reviewer for this comment. This has been added.

Comment #9: (b) The authors admitted a long list of limitations in their work, notably, the "...unable to blind the subjects, or physiotherapists...". Despite the justification provided, I am not sure how good the trial is! With 40 subjects recruited, the results

	stated, at best, can only be claimed as from a pilot study. This needs to be clearly stated, and the study cannot be claimed as a nicely planned randomized trial. Also, they need to state that future studies (with larger sample sizes) are warranted to really understand the comparative efficiacy.<br><br>Response: We thank the reviewer for these comments. Please see the additions in lines 410-441.
Additional Information:	
Question	Response
**Financial Disclosure**<br><br>Enter a financial disclosure statement that describes the sources of funding for the work included in this submission. Review the submission guidelines for detailed requirements. View published research articles from *PLOS ONE* for specific examples.<br><br>This statement is required for submission and **will appear in the published article** if the submission is accepted. Please make sure it is accurate.<br><br>**Unfunded studies**<br>Enter: *The author(s) received no specific funding for this work.*<br><br>**Funded studies**<br>Enter a statement with the following details:<br>• Initials of the authors who received each award<br>• Grant numbers awarded to each author<br>• The full name of each funder<br>• URL of each funder website<br>• Did the sponsors or funders play any role in the study design, data collection and analysis, decision to publish, or preparation of the manuscript?<br>• **NO** - Include this sentence at the end of your statement: *The funders had no role in study design, data collection and analysis, decision to publish, or preparation of the manuscript.*<br>• **YES** - Specify the role(s) played.<br><br>* typeset	Funding Project: Sanming Project of Medicine (SZSM201612055) “Team of Excellence in Spinal Deformities and Spinal Degeneration Diseases” in Shenzhen, Guangdong province, China.<br><br>Receiver: Kenneth MC Cheung<br><br>Professor Kenneth Cheung participated in the methodology and preparation of the manuscript.
**Competing Interests**<br><br>Use the instructions below to enter a competing interest statement for this submission. On behalf of all authors,	The authors have declared that no competing interests exist.

disclose any competing interests that could be perceived to bias this work—acknowledging all financial support and any other relevant financial or non-financial competing interests.<br><br>This statement **will appear in the published article** if the submission is accepted. Please make sure it is accurate. View published research articles from *PLOS ONE* for specific examples.<br><br>**NO authors have competing interests**<br><br>Enter: *The authors have declared that no competing interests exist.*<br><br>**Authors with competing interests**<br><br>Enter competing interest details beginning with this statement:<br><br>*I have read the journal's policy and the authors of this manuscript have the following competing interests: [insert competing interests here]*<br><br>* typeset	
**Ethics Statement**<br><br>Enter an ethics statement for this submission. This statement is required if the study involved:<br><br>• Human participants<br>• Human specimens or tissue<br>• Vertebrate animals or cephalopods<br>• Vertebrate embryos or tissues<br>• Field research<br><br>Write "N/A" if the submission does not require an ethics statement.<br><br>General guidance is provided below. Consult the submission guidelines for detailed instructions. **Make sure that all information entered here is included in the Methods section of the manuscript.**	Human Subject Research<br>•Name of Committe:<br>The Ethic Committe Board of The University of Hong Kong - Shenzhen Hospital<br><br>•Approval number:No.:伦[2016]91<br>Original protocol: GNSF201603<br><br>•Form of consent obtained (written/oral) : Written

Powered by Editorial Manager® and ProduXion Manager® from Aries Systems Corporation

**Format for specific study types**<br><br>**Human Subject Research (involving human participants and/or tissue)**<br>• Give the name of the institutional review board or ethics committee that approved the study<br>• Include the approval number and/or a statement indicating approval of this research<br>• Indicate the form of consent obtained (written/oral) or the reason that consent was not obtained (e.g. the data were analyzed anonymously)<br><br>**Animal Research (involving vertebrate animals, embryos or tissues)**<br>• Provide the name of the Institutional Animal Care and Use Committee (IACUC) or other relevant ethics board that reviewed the study protocol, and indicate whether they approved this research or granted a formal waiver of ethical approval<br>• Include an approval number if one was obtained<br>• If the study involved *non-human primates*, add *additional details* about animal welfare and steps taken to ameliorate suffering<br>• If anesthesia, euthanasia, or any kind of animal sacrifice is part of the study, include briefly which substances and/or methods were applied<br><br>**Field Research**<br><br>Include the following details if this study involves the collection of plant, animal, or other materials from a natural setting:<br>• Field permit number<br>• Name of the institution or relevant body that granted permission	
**Data Availability**<br><br>Authors are required to make all data underlying the findings described fully available, without restriction, and from the time of publication. PLOS allows rare exceptions to address legal and ethical concerns. See the PLOS Data Policy and FAQ for detailed information.	Yes - all data are fully available without restriction

A Data Availability Statement describing where the data can be found is required at submission. Your answers to this question constitute the Data Availability Statement and **will be published in the article**, if accepted.<br><br>**Important:** Stating 'data available on request from the author' is not sufficient. If your data are only available upon request, select 'No' for the first question and explain your exceptional situation in the text box.<br><br>Do the authors confirm that all data underlying the findings described in their manuscript are fully available without restriction?	
**Describe where the data may be found in full sentences. If you are copying our sample text, replace any instances of XXX with the appropriate details.**<br><br>• If the data are **held or will be held in a public repository**, include URLs, accession numbers or DOIs. If this information will only be available after acceptance, indicate this by ticking the box below. For example: *All XXX files are available from the XXX database (accession number(s) XXX, XXX.*).<br>• If the data are all contained **within the manuscript and/or Supporting Information files**, enter the following: *All relevant data are within the manuscript and its Supporting Information files.*<br>• If neither of these applies but you are able to provide **details of access elsewhere**, with or without limitations, please do so. For example:<br><br>*Data cannot be shared publicly because of [XXX]. Data are available from the XXX Institutional Data Access / Ethics Committee (contact via XXX) for researchers who meet the criteria for access to confidential data.*<br><br>*The data underlying the results presented in the study are available*	All relevant data are within the manuscript and its Supporting Information files.<br><br>data (same as in supporting information files) will be availbale on an open public access if paper recieved: ResMan<br>www.medresman.org

from (include the name of the third party and contact information or URL).<br>• This text is appropriate if the data are owned by a third party and authors do not have permission to share the data.<br><br>* typeset	
Additional data availability information:	Tick here if the URLs/accession numbers/DOIs will be available only after acceptance of the manuscript for publication so that we can ensure their inclusion before publication.

Powered by Editorial Manager® and ProduXion Manager® from Aries Systems Corporation

Manuscript Click here to access/download;Manuscript;Manuscript.docx

Title: Dose curve pattern impact on the effects of physiotherapeutic scoliosis specific exercises on Cobb angles of participants with adolescent idiopathic scoliosis: a prospective clinical trial with two years follow-up

Authors

1. Yunli Fan[1,2,3]
2. Michael KT To[2]
3. Eric HK Yeung[3]
4. Jianbin Wu[1]
5. Rong He[3]
6. Zhuoman Xu[3]
7. Ruiwen Zhang[3]
8. Guangshuo Li[3]
9. Kenneth MC Cheung[2]
10. Jason PY Cheung[2*]

[1]Department of Orthopedics, The University of Hong Kong – Shenzhen Hospital, Shenzhen, Guangdong Province, People's Republic of China

[2]Department of Orthopaedics and Traumatology, The University of Hong Kong, Hong Kong SAR, China

[3]Department of Physiotherapy, The University of Hong Kong – Shenzhen Hospital, Shenzhen, Guangdong Province, People's Republic of China

*** Corresponding author:**

Name	Cheung, Jason Pui Yin
Department	Department of Orthopaedics and Traumatology
Institution	The University of Hong Kong
Country	Hong Kong Special Administration Region, China
Email	cheungjp@hku.hk

Dose curve pattern impact on the effects of physiotherapeutic scoliosis specific exercises on Cobb angles of participants with adolescent idiopathic scoliosis: a prospective clinical trial with two years follow-up

Yunli Fan[1,2,3], Michael KT To[2], Eric HK Yeung[3], Jianbin Wu[1], Rong He[3], Zhuoman Xu[3], Ruiwen Zhang[3], Guangshuo Li[3], Kenneth MC Cheung[2], Jason PY Cheung[2*]

Abstract

Background

Current clinical evidence suggests that a well-planned physiotherapeutic scoliosis specific exercise (PSSE) program is effective for scoliosis regression.

Objectives

We investigated the effect of curve patterns on Cobb angles with PSSE.

Methods

This was a prospective clinical trial that recruited participants with adolescent idiopathic scoliosis between January and June 2017. Participants were grouped by curve pattern into major thoracic and major lumbar groups. An outpatient-based PSSE program was conducted with the following schedule of intensive exercise: ≥ 1 session of supervised PSSE per month and > 30min of home exercise 5 days/week in the first 6 months, after which exercise frequency was reduced to

1 session of supervised PSSE every three months and > 30min of home exercise 5 days/week until 2 years after study initiation. Radiographic Cobb angle progressions were identified at the 1, 1.5 and 2-year follow-ups. One-way mixed repeated measures analyses of variance were performed to examine the differences in Cobb angles between groups at four testing time points. The two-tailed significance level was set to 0.05.

Results

In total, 40 participants were recruited, including 22 with major thoracic curves (5 male and 17 female; mean age 13.5±1.8 years; Cobb angle 18 - 45 degrees) and 18 with major lumbar curves (7 male and 11 female; mean age 12.7±1.7 years; Cobb angle 15 - 48 degrees). Curve regressions, namely the reduction of Cobb angles between 7 to 10 degrees were noted in 9.1% of participants in the major thoracic group; reductions of 6 to 13 degrees were noted in 33.3% of participants in the major lumbar group at the 2-year follow-up. Repeated measurements revealed a significant time effect ($F_{2.2,81.9}$=6.9, p=0.001), but no group ($F_{2.2,81.9}$=2.2, p=0.1) or time × group ($F_{1,38}$=1.4, p=0.2) effects in reducing Cobb angles ~~were observed~~ after 2 years of PSSE. In addition, 65% of participants (n=26) reached skeletal maturity (Risser sign = 5) and achieved significant reductions in progression risk at the 2-year follow-up (Lonstein and Carlson Risk of progression: 0.45 ± 0.32 vs. 0.13 ± 0.14, p <0.001).

Conclusion

This was the first study to investigate the long-term effects of PSSE in reducing Cobb angles on the basis of major curve location. No significant differences in correction were observed between major thoracic and major lumbar curves. However, a regression effect and no curve deterioration were noted in both groups at the 2-year follow-up.

Trial Registration: ChiCTR1900028073

*** Corresponding author:** cheungjp@hku.hk

[1]Department of Orthopedics, The University of Hong Kong – Shenzhen Hospital, Shenzhen, Guangdong Province, People's Republic of China

[2]Department of Orthopaedics and Traumatology, The University of Hong Kong, Hong Kong SAR, China

[3]Department of Physiotherapy, The University of Hong Kong – Shenzhen Hospital, Shenzhen, Guangdong Province, People's Republic of China

Introduction

Adolescent idiopathic scoliosis (AIS) is a three-dimensional spinal deformity with an unknown etiology, characterized by lateral deviation in the frontal plane, axial rotation in the horizontal plane and an abnormal sagittal curvature.[1] Surgery is typically recommended if a spinal curve reaches 50 degrees, because such curves are associated with a continued progression risk into

adulthood.[1-3] Surgical fusion causes spine spine stiffness and should be avoided if possible.[3] Thus, the goal of conservative managements including bracing and exercises, is to prevent spinal deformity deterioration past the operative threshold.[4]

Options for nonsurgical management vary widely and depend on the prognostic evaluation of curve progression.[4] Curve type is an established risk factors of scoliosis progression.[5] In particular, a thoracic curve with a larger Cobb angle has a higher likehood of progression than single lumbar or thoracolumbar curves do.[2] Therefore, understanding the effect of curvature on interventions can help clinicians select appropriate treatments for patients.

Bracing is commonly prescribed for moderate scoliosis, and its corrective effect is influenced by the location of structural curves.[6, 7] In addition, scoliosis-specific exercise is a nonoperative option that is well received by patients and families.[8] Several systematic reviews and randomized controlled trials have reported the positive effects of physiotherapeutic scoliosis specific exercise (PSSE) on slowing curve progression as well as improving cosmetic and quality of life;[9-11] however, the relationship of curve location with correction effects has not been clearly discussed in these studies, in which small samples of participants have been recruited and followed up over the short-term.[12-14] In particular, one 6-month long randomized control trial (RCT) revealed a significant correlation between thoracic curvature with an imbalanced pelvis (n=15) and the largest curves after Schroth treatment;[12] one 4-month long RCT demonstrated the greater Cobb reduction was noted in the thoracic region (n=20) after body awareness exercise.[13]

Therefore, a clinical trial, in which curve pattern is controlled, is warranted to investigate the long-term effects of curve pattern and PSSE on reducing Cobb angles. This is valuable for clinical practice because the deterioration is possible after initial improvement in short-term follow-up.

According to current evidence from PSSE studies,[9, 10, 15] the Schroth is the most used PSSE approach. It adopts specific respiratory technique-asymmetrical breathing in the diagonal direction to achieve vertebral and rib cage derotation.[16] It utilizes muscle activation and emphasizes core muscle stabilization in a corrected posture throughout the day to change habitual postures and improve spinal alignment.[16, 17] However, the influence of curve magnitude on Schroth method outcomes is unclear. The spinal deformity profile that leads to optimal outcomes with Schroth exercise is also unclear. Thus, this study investigated the long-term therapeutic effects of Schroth exercises on major thoracic and major lumbar curves, respectively. We hypothesized that patients with lumbar curves, which have a lower reported risk of curve progression and higher spinal segmental flexibility, benefit more from long-term PSSE treatment than do patients with major thoracic curves. The current results can provide physiotherapists with additional information to develop individualized exercises based on curve magnitude. These results can also form the basis for further RCTs evaluating the relationship between curve patterns and PSSEs.

Materials and methods

Study design

This was a prospective clinical trial in which participants with AIS were recruited consecutively between January and June 2017 from one scoliosis clinic at the University of Hong Kong - Shenzhen Hospital (HKU - SZH), China. This study obtained ethical approval by the institutional review board of the HKU - SZH with reference number: GNSF201603 (**S1** File). Individuals in this manuscript have given written informed consent to publish these case details. The authors confirm that all ongoing and related trials for this intervention are registered in the Chinese clinical trial registry (www.chictr.org.cn, trail registration: ChiCTR1900028073) (**S1** File). Participants and legal guardians signed a written informed consent for PSSE treatment and participants were followed up for 2 years thereafter. The inclusion criteria were age 10 to 16 years; bone immaturity in terms of Risser sign was less than 5;[18] Cobb angles[19] from 10 degrees to 50 degrees; and complete attendance for all physiotherapy sessions. Exclusion criteria were diagnoses other than AIS, disabilities or systemic illnesses that prevent participants from performing exercise, hypermobility (Beighton score[20] greater than 4) and previous treatment for AIS. Joint hypermobility (JHM) is reported to have a higher prevalence in patients with single curves than in those with double-curve scoliosis; and this may affect physiotherapy outcomes.[21] Therefore, patients with hypermobility were excluded from this study to control for systematic error introduced by JHM differences between curve patterns. Participants were categorized into two groups according to their whole spine radiographic characteristics:[22, 23] participants with 3C (major thoracic with or without a minor lumbar curve) or N3N4 curves (double, well balanced curves) formed the major thoracic group; and those with single lumbar, single thoracolumbar, or 4C curves (a major lumbar curve with a minor thoracic) comprised the major lumbar group (**S2** Fig 1).

S2 Fig 1. CONSORT flow diagram.

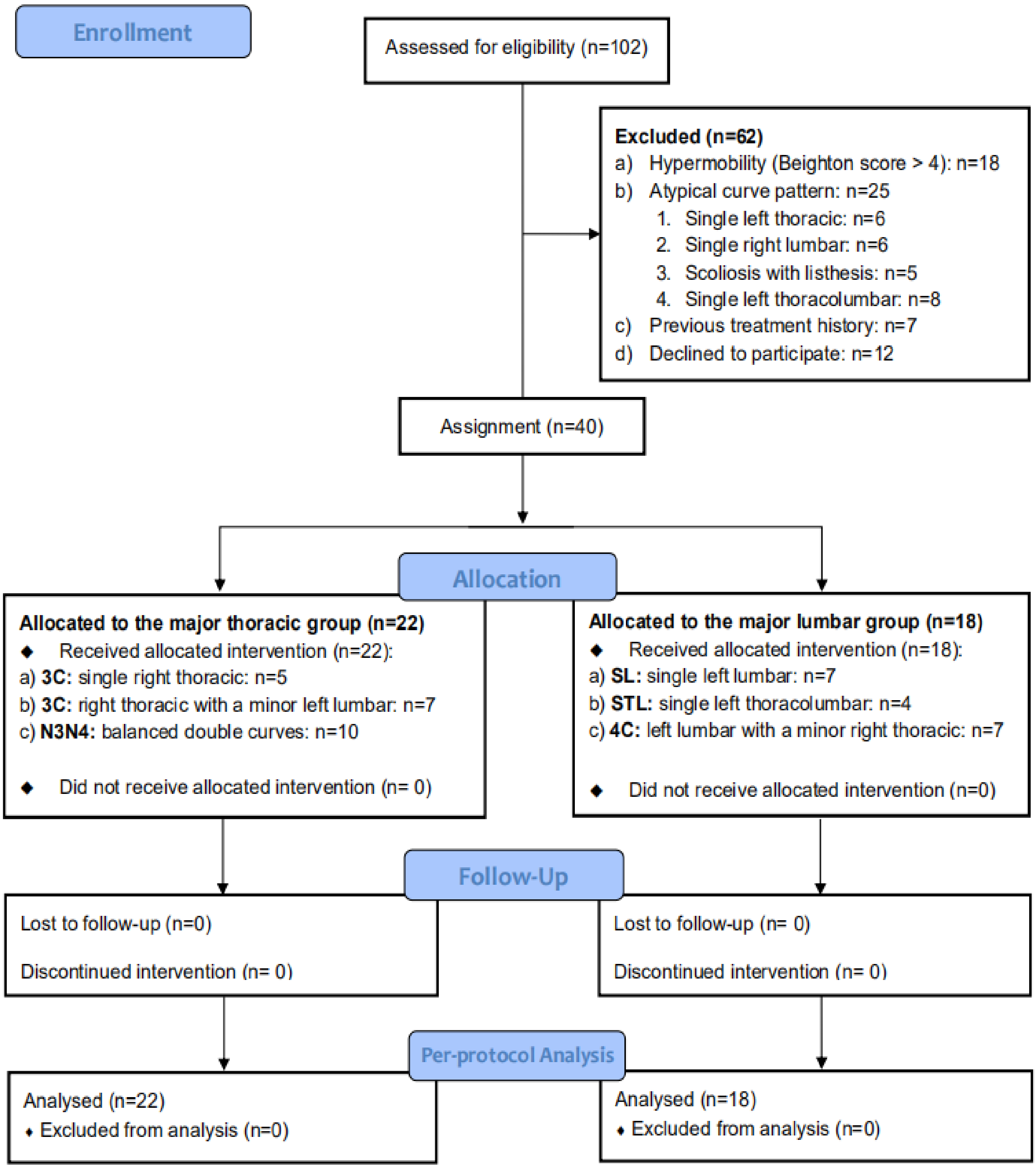

Study intervention

All participants participated in a 6-month intensive PSSE program supervised by two

PSSE-Schroth certified physiotherapists (Barcelona Scoliosis Physical Therapy School [BSPTS] approach).[22] Supervised treatment during the 6 months occurred ⩾1 time per month for 1 hour each time. Participants were instructed to perform >30min sessions of home exercise at least ⩾5 times per week. For participants who met Scoliosis Research Society bracing criteria, Cheneau bracing was prescribed prior to PSSE program;[24] in-brace correction was documented by a spine surgeon who was blinded to this study. Bracing compliance was reported by patients on their monthly treatment adherence checklist (**S3** File). After the first 6 months of PSSE, all participants reduced their frequency of supervised PSSE to once every 3 months to review their exercises, which they performed for 2 years after completing the PSSE program. During this time, the frequency of home exercise was maintained. Attendance for supervised PSSE in an outpatient setting was recorded in the hospital's prospective database system. Participants were requested to document home exercise and bracing compliance on daily basis using a treatment adherence checklist (**S3** File), and handed it to a physiotherapist assistant who was blinded to this study every month during the first 6-month study period; then they were required to submit the checklist every 3 months thereafter. Exercise compliance was ~~final~~ calculated in hours per week per year for analysis. The PSSE intervention consisted of 50 breaths per exercise. Short semihanging with caudal-cranial spine lengthening (Fig 2. c), corrective exercise in side lying with shoulder counter traction (Fig 2. d), corrective exercise in sitting with pelvic pulling strategy on the side of weak point and shoulder counter traction (Fig 2. e), corrective exercise in standing with pelvic derotation and shoulder counter traction (Fig 2. f) were prescribed for participants in the major thoracic group. Short semihanging with caudal-cranial spine lengthening (Fig 3. c), corrective exercise in side lying with passive correction on the lumbar prominence (Fig 3. d), corrective exercise in standing with

shoulder counter traction (Fig 3. e), muscle cylinder in standing for opening lumbar concavity (Fig 3. f) and the corrective exercise in sitting with shoulder counter traction (Fig 3. g) were prescribed to participants in the major lumbar group. The difference between the two groups' exercises was that a muscle cylinder was prescribed only to participants with major lumbar curves. The muscle cylinder exercise in a kneeling position is typical prescribed to patients with major thoracic curves; however, patients commonly complain that it causes knee pain. Therefore, consider the potential adverse effects during long-term follow-up, the muscle cylinder in a kneeling position was not prescribed in this study. Moreover, dynamic exercise, such as Schroth walking was not used in this study because of limited space in our center. In addition, participants were asked to record and report any discomfort, such as muscle fatigue and muscle sprain, to the on-duty physiotherapist during the study period.

Fig 2. Exercises demonstration for major right thoracic curves

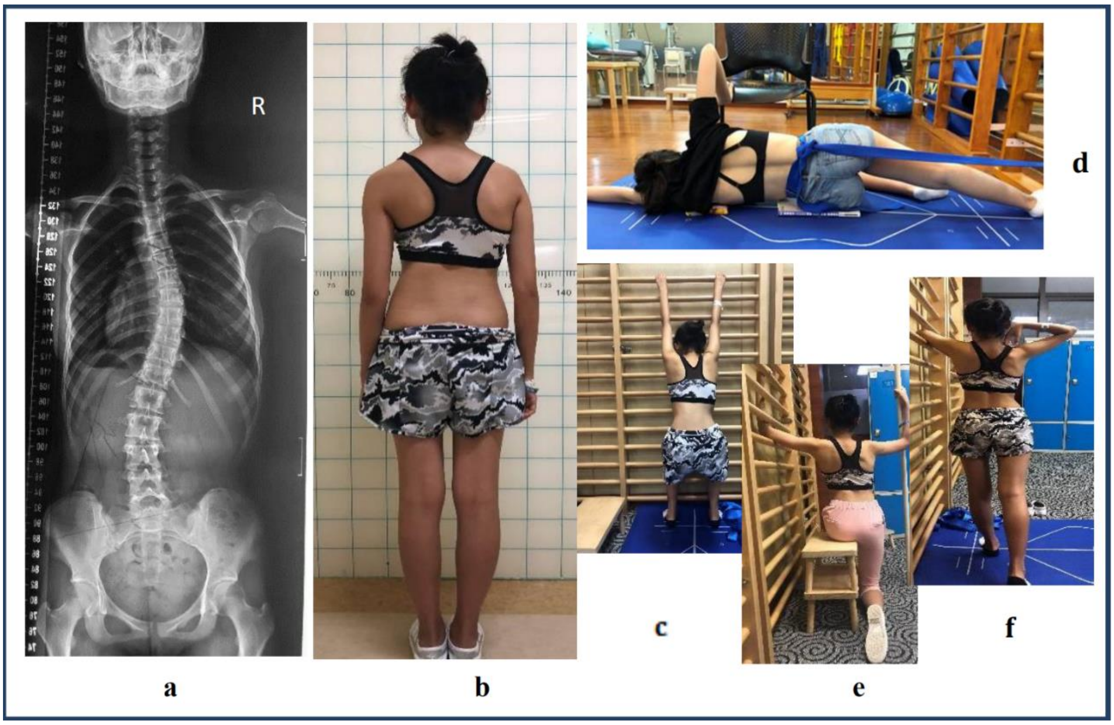

Fig 2: a: a major right thoracic (T4 to 12) with a Cobb angle of 30 degrees. **b:** posterior posture view of the participant. **c:** short semihanging with caudal-cranial spine lengthening. **d:** corrective exercise in side lying with shoulder counter traction. **e:** corrective exercise in sitting with pelvic pulling strategy on the side of weak point and shoulder counter traction. **f: c**orrective exercise in standing with pelvic derotation and shoulder counter traction.

Fig 3. Exercises demonstration for major left lumbar curves

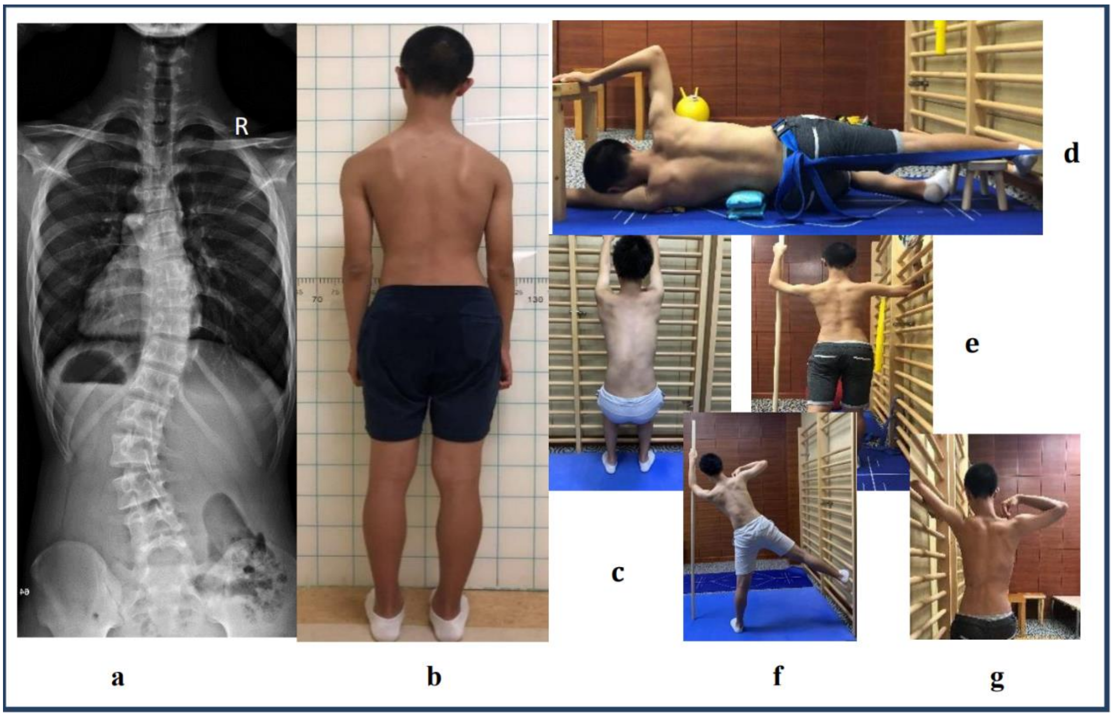

Fig 3: a: a major left lumbar curve (T12 to L4) with a Cobb angle of 48 degrees. **b:** posterior posture view of the participant. **c:** short semihanging with caudal-cranial spine lengthening. **d:** corrective exercise in side lying with passive correction on the lumbar prominence. **e:** corrective exercise in standing with shoulder counter traction. **f: m**uscle cylinder in standing for opening lumbar concavity. **g: c**orrective exercise in sitting with shoulder counter traction.

Outcome measurement

Participants underwent whole-spine radiography in a standing position with arms slightly abducted at the sides of body. The Cobb angles of major curves were assessed by a spine surgeon who was blinded to this study. Cobb angles were recorded in the initial assessment and every 6 months after the PSSE program until the 2-year follow-up. Participants remained out of their braces overnight prior to obtaining radiography. Curve regression was defined as a decrease in the Cobb

angle by 6 degrees or more, stabilization was defined as a <6 degrees change in Cobb angle, and deterioration was defined as a Cobb angle increase of 6 degrees or more.[2, 25]

Statistics

Sample size estimation

To obtain an effect size of 0.50, 80% power for a two-tailed test, and a correlation of 0.6 for repeated measures of Cobb angles over 4 time points, 13 participants were required for each group.[26] Considering possible 35% dropout rate during the 2-year study period, each group were planned to have 18 participants (**S2** Fig 1).

Statistical analysis

The baseline, continuous variables, including age, Lonstein and Carlson Risk of progression value (LCR-value)[27] and initial Cobb angles were evaluated using independent t test if assumptions of normality was met; otherwise the Kruskal-Wallis test was performed if normality assumptions were violated. The chi-square test was used to compare categorical variables including sex, Risser sign (stage: 0-2 or 3-4), curvature types (the single thoracic curve and the major thoracic with a minor lumbar curve vs the single lumbar/thoracolumbar and the major lumbar with a minor thoracic curve) and brace treatment (yes or no) for all participants (Table 1). Changes in Cobb angles, in the form of the difference value (D-value) between testing times, were measured to detect curve progression, stabilization and regression between groups; raw Cobb

angles were repeatedly compared over four testing time points to detect time, group and time × group interaction effects in reducing Cobb angles. One-way analysis of variance (ANOVA) was performed if the normality assumptions of Cobb angles were met whereas a nonparametric Friedman-type test would be performed if normality was violated. Post hoc pairwise comparison was conducted if any significant differences were detected in either within-subjects or between-subjects comparisons. A Logistic regression was performed to detect correlations of age, sex, brace treatment, curve pattern with the regression, stabilization or deterioration after 2 years of PSSE. The data were analyzed using SPSS version 20.0 (IBM, Chicago, IL). The level of significance was set to 0.05 with a two-tailed test.

Results

Participants

In total, 102 patients were screened to confirm eligibility, after which 62 patients were excluded for the following reasons: hypermobility (Beighton score > 4) was detected in 18 patients; 25 patients had atypical curve patterns[28, 29] (single left thoracic; n=6, single left lumbar: n=6; scoliosis ~~was~~ presented with vertebral listhesis: n=5, single left thoracolumbar: n=8); 7 patients were previously treated with either bracing or exercise; 12 patients declined to participate in this study. Therefore, 40 participants were recruited and all completed follow-up (**S2** Fig 1). The major thoracic group had 22 participants (5 males and 17 females). Of them, 12 had 3C curves (single right thoracic: n=5; right thoracic with a minor left lumbar: n=7) and 10 had N3N4 curves (balanced right thoracic with a left lumbar). The major lumbar group had 18 participants (7 males

and 11 females). Of them, 7 had single left lumbar curves, 4 had right thoracolumbar curves and 7 had 4C curves (left lumbar with a minor right thoracic). The mean age of participants in the major thoracic and major lumbar groups was 13.5±1.8 years and 12.7±1.7 years, respectively (Table 1). At baseline, the two groups demonstrate~~d non~~ significant difference in age, sex, brace treatment, Risser sign (0-2 / 3-4), proportion of sub-curvature types, LCR-value, or initial Cobb angle of the major curve (Table 1). Participants with brace treatment (n=10) who started Cheneau orthosis before commencing the PSSE and achieved comparable 37% of curve correction between groups (Table 1). In particular, 5 of them were wearing night-time brace only (≤ 8 hours/day) whereas 5 were wearing full-time brace (21 hours/day) (Table 1). In addition, two participants in the major lumbar group ~~who~~ refused brace treatment for cosmetic reasons.

Table 1. Baseline characteristics of participants

	~~The major~~ **thoracic group** (95% confidence interval)	~~The~~ **major lumbar group** (95% confidence interval)	***P* value**
**Age** (years: Mean±SD)	13.5 ± 1.8 (12.8 - 14.6)	12.7 ± 1.7 (12.6 - 13.7)	0.1
**Sex**			
Female	17 (77.3%)	11 (61.1%)	0.3
Male	5 (22.7%)	7 (38.9%)	

**Risser sign**			0.6
0-2 / 3-4	9 / 13	9 / 9	
**Initial Cobb Angle** (min-max)	18 - 45	15 - 48	0.6
(degrees:Mean±SD)	28.5 ± 8.4 (24.5 - 32.5)	26.8 ± 10.1 (22.4 - 31.2)	
**Curve pattern**			
3C / SL or STL	12	11	0.7
N3N4 / 4C	10	7	
**Braced** (yes)	7	3*	0.5
In-brace hours (hours/day)	13.6 ± 6.9	16.7 ± 7.5	
full-time brace (≥ 21hr/day)	3	2	
night-time brace (≥8hr/day)	4	1	
**In-brace Cobb angle**			
(degrees: Mean ± SD)	23 ± 4.4	26 ± 6	0.9
correction rate (%)	37	37	
**Progression risk**	44 %	46%	0.8
Lostein and Carlson risk			

**Exercise compliance**			
1-year: hours/week	5.5 ± 2.2	5.5 ± 2.1	0.9
2-year: hours/week	3.0 ± 0.8§	4.1± 2.1	**0.04**

3C: A single right thoracic curve or a major right thoracic with a minor left lumbar. **SL:** A single left lumbar. **STL:** A single right thoracolumbar. N3N4: double curves but well balanced. **4C:** A major left lumbar with a minor right thoracic. * Not including two participants who refused brace treatment. § significantly differ from the compliance value at the 1-year follow-up in the major thoracic group.

Exercise compliance

The two groups differed significantly in exercise compliance during the second year of follow-up (3.0±0.8 hours/week vs 4.1±2.1 hours/week, p=0.04). In addition, mean exercise compliance of the major thoracic group was significantly decreased in the second year (5.5±2.2 hours/week vs 3.0±0.8 hours/week; p<0.01); the mean exercise compliance of the major lumbar group was 5.5±2.1 hours/week in the first year and slightly decreased to 4.1±2.1 hours/week in the second year (Table 1). There were no adverse effects detected during this study period.

Intervention effects

In total, 23 participants (57.5%) with Cobb angle < 30 degrees refused to repeat

radiography at the 6-month follow-up after the PSSE program because of radiation exposure concerns; therefore, no comparison of participants before and at the 6-month follow-up was performed. Nonetheless, all participants completed radiography at the 1-year, 1.5-year and 2-year follow-up, respectively. Full set of raw data was available in the **S4** File.

No deterioration of the major curvature occurred in either group (both average and individual data) at the 2-year follow-up (Table 2). Specifically, curve regressions, reduction of Cobb angles between 7 to 10 degrees were noted in 9.1% of participants in the major thoracic group; reductions of 6 to 13 degrees were noted in 33.3% of participants in the major lumbar group (Table 2). However, no statistical effects of group ($F_{1.5,56.5}$=2.7, p=0.09) and time ($F_{1.5,56}$=1.2, p=0.3) or the interaction effect of time × group ($F_{1,38}$=1.8, p=0.2) with the D-value angles of Cobb were observed after 2 years of regular PSSE.

Table 2. Repeated analysis for the D-value of Cobb angles over three testing time points

Testing times	Group	Mean	Std. Deviation	95% Confidence Interval	Min value	Max value	Regressed (n)	Stabilized (n)	Deteriorated (n)
**1-year**	0	-1.5	4.7	-3.5 - 0.53	5	-12	4	18	0
	1	-2.1	4.8	-4.4 - 0.2	7	-13	2	15	1
	**Total**	**-1.8**	**4.7**	**-3.8 - 0.3**	**5**	**-13**	**6**	**33**	**1**
**1.5-year**	0	-1.4	4.7	-3.5 - 0.6	5	-15	3	19	0

	1	-3.5	4.9	-5.8 - -1.2	5	-13	6	12	0
	**Total**	**-2.4**	**4.8**	**-4.6 - 0.3**	**5**	**-15**	**9**	**31**	**0**
**2-year**	0	-1.0	4.2	-2.9 - 0.8	5	-10	2	20	0
	1	-3.8	4.3	-5.9 - -1.8	3	-13	6	12	0
	**Total**	**-2.3**	**4.4**	**-4.1- 1.3**	**3**	**-13**	**8**	**32**	**0**

Group 0: the major thoracic group. **Group 1:** the major lumbar group. **Regressed:** a decrease of Cobb angle ≥ 6 degrees. **Stabilized:** a change of Cobb angle < 6 degrees. **Deteriorated:** an increase of Cobb angle ≥ 6 degrees.

Per-protocol analysis was performed to analyze the raw value of Cobb angles over four testing time-points between groups. Mauchly test indicated that the assumption of sphericity was violated (χ^2 =32.5 , df =5 , p < 0.001); hence, the degrees of freedom were corrected using Greenhouse-Geisser estimates of sphericity. Repeated analysis showed a significant time effect ($F_{2.2,81.9}$=6.9, p=0.001), but no group ($F_{2.2,81.9}$=2.2, p=0.1) or time × group ($F_{1,38}$=1.4, p=0.2) effects were detected in reducing Cobb angles after 2 years of treatment (Table 3). In the major lumbar group, a reduction was noted in the Cobb angle (26.8±10.1 degrees vs 23.3±9.4 degrees, p=0.007) after training for one and a half years with a maintained reduction after two years of follow-up (26.8±10.1 degrees vs 22.9±9.2 degrees, p<0.001) (Table 3). In comparison to initial Cobb angles within participants with the major lumbar curve, the curves improved (Cobb angles: -9.5±4.9 degrees) in 11.1%, stabilized (Cobb angles: -3.4±1.2 degrees) in 83.3% and deteriorated by 7 degrees in one subject after the first year of training (Table 2). In the second year of follow-up, up to 33.3% of participants had improved Cobb angles (Cobb angles: -8.2±2.5 degrees), while 66.7%

stabilized (Cobb angles: -3.2±1.6 degrees), and no deterioration was noted (Table 2). Of the participants with curve regression in the first year, one maintained the curve regression, reducing Cobb angle of 13 degrees, and one only gained an additional degree in the second year. Moreover, one participant with a single left lumbar curve of 31 degrees (Fig. 4: a) who refused brace treatment ~~but~~ obtained ~~in~~ a Cobb angle of 23 degrees (Fig. 4: b) after one and a half years of PSSE. In addition, another participant with a single right thoracolumbar curve of 44 degrees (Fig. 5: a) ~~who~~ obtained a Cobb angle of 31 degrees (Fig. 5: b) after two years of PSSE.

Table 3. Per-protocol repeated measures of Cobb angles over four testing time points

Testing timepoint	Group	Mean	Std. Deviation	95% Confidence Interval	Minimum	Maximum	*P*
**Baseline**	0	28.5	8.4	24.5-32.5	18	45	-
	1	26.8	10.1	22.4-31.2	15	48	-
	Total	27.7	9.1	24.7-30.6	16	46	-
**1-year**	0	27	7.2	23.4-30.6	18	38	0.152
	1	24.7	9.4	20.8-28.7	10	42	0.079
	Total	**26.0**‡	8.2	23.2-28.5	14	40	**0.022**
**1.5-year**	0	27.1	7.7	23.4-30.9	10	40	0.17
	1	**23.3***	9.4	19.2-27.3	10	45	**0.007**
	Total	**25.4**‡	8.6	22.5-27.9	10	43	**0.002**
**2-year**	0	27.5	7.0	24.0-31.0	15	40	0.254

1	**22.9***	9.2	19.1-26.8	10	43	**0.001**
Total	**25.4**ŧ	8.2	22.6-27.8	13	42	**0.001**

Group 0: the major thoracic group. **Group 1:** the major lumbar group. ***** significantly differ from the baseline in the major lumbar group. ŧ significantly differ from the mean value at baseline.

Fig 4. Radiographic assessment (posterior anterior view) of one participant with a single left lumbar scoliosis who refused brace treatment for cosmetic reasons.

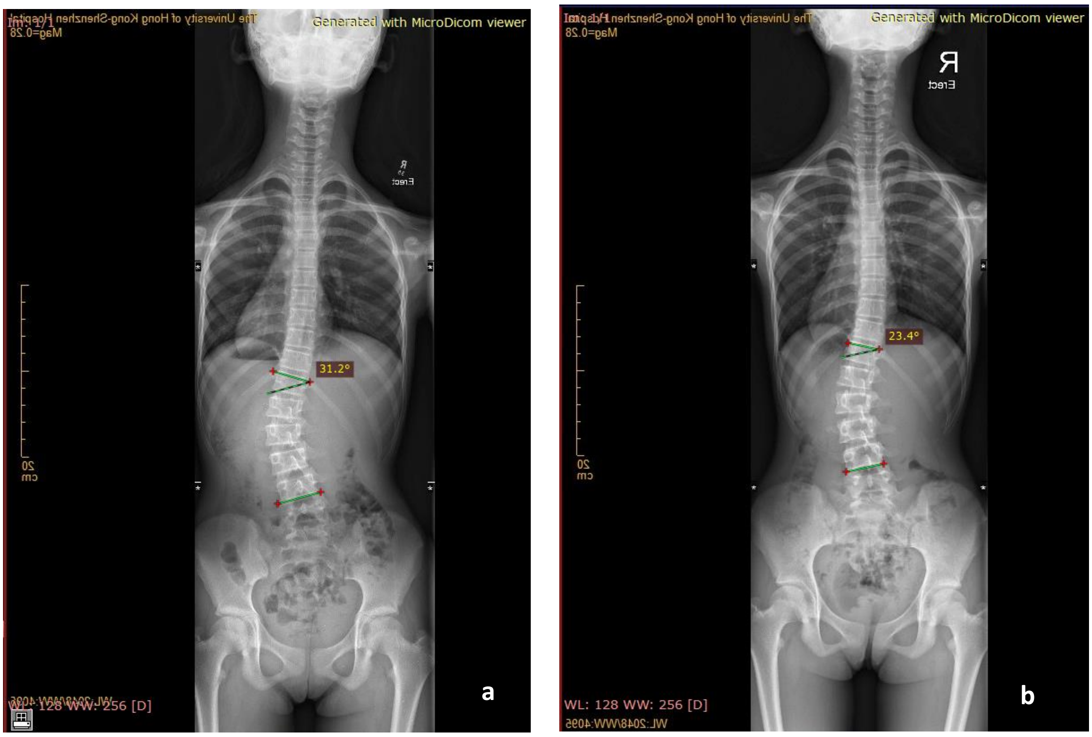

a: an initial Cobb angle of 31 degrees from T12 to L4. **b:** a Cobb angle of 23 degrees from T12 to L4 at the 1.5-year of follow-up.

Fig 5. Radiographic assessment (posterior anterior view) of one participant with a single right

thoracolumbar scoliosis who refused brace treatment for cosmetic reasons.

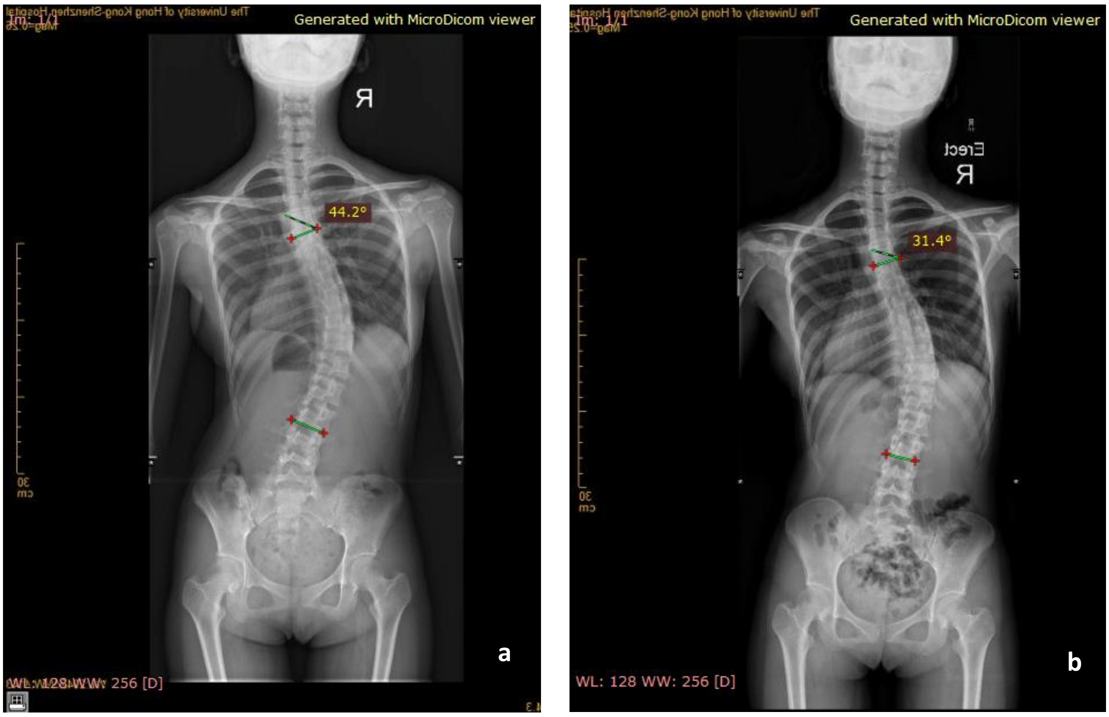

a: an initial Cobb angle of 44 degrees from T5 to L2. **b:** a Cobb angle of 31 degrees from T5 to L2 at the two-year follow-up.

In the major thoracic group, no difference was observed in Cobb angle (28.5±8.4 degrees vs 27.0±7.2 degrees, *p*=0.15; 28.5±8.4 degrees vs 27.1±7.7 degrees, *p*=0.17; 28.5±8.4 degrees vs 27.5±7.0 degrees, *p*=0.25) after one year, one and a half years and two years of follow-up, respectively (Table 3). However, after training, the spinal deformity improved (Cobb angles: -8.8±2.5 degrees) in 18.2% of participants and stabilized (Cobb angle: -2.9±1.6 degrees) in 81.8% of participants, and none deteriorated in the first year (Table 2). Only 2 participants maintained curve regression in the second year, which resulted 9.1% of participants showed regression (Cobb angles: -8.5±2.1 degrees) (Table 2). Thus, up to 90.9% of participants stabilized (Cobb angles: -

3.1±1.6 degrees), and no deterioration was detected at the 2-year follow-up.

With 20% regression (n=8) and 80% stabilization in this study, in which 65% of participants (n=26) reached bone maturity (Risser: 5) after two years of PSSE and no curve pattern was changed (Table 4). The risk of progression (LCR-value) significantly decreased in both groups (major thoracic: 44% vs 12%, $p<0.001$; major lumbar: 46% vs 11%, $p<0.001$) at the 2-year follow-up. Logistic regression analysis revealed that there was no correlation of age (OR: 0.7, 95% CI: 0.3 - 1.7, p=0.49), sex (OR: 0.9, 95% CI: 0.2 - 5.7, p=0.94), skeletal maturity (OR: 0.69, 95% CI: 0.05 - 10.6, p=0.79), bracing (OR: 0.4, 95% CI: 0.35-4.05, p=0.42) or curve patterns (OR: 4.2, 95% CI: 0.46 - 37.9, p=0.2) with curve regression or stabilization at the 2-year follow-up.

Table 4 Demographic and radiographic characteristics of participants at the 2-year follow-up.

**The 2-year follow-up**	**Major thoracic curves (n=22)**	**Major lumbar curves (n=18)**
**Age** (Mean±SD)	15.6 ± 1.8	14.7 ± 1.7
**Risser sign** 0-2,3-4,5 (n)	0,4,18	0, 10,8
**Cobb angle** (Mean±SD)	27.5 ± 7.0	22.9 ± 9.2
≥ 30 degrees: n (%): min – max	8 (36.4%): 31° - 40°	5 (27.8%): 30° - 43°
<30 degrees: n (%): min – max	14 (63.6%):15°- 28°	13 (72.2%): 10° -27°
**Curve pattern:** 3C / SL or STL (n)	12	11
N3N4 / 4C (n)	10	7

**Progression risk:** LCR-value (%)	12	11

LCR-value: Lostein and Carlson Risk of progression value.

Discussion

This was the first study to investigate the long-term outcomes of patients with different AIS curve patterns undergoing PSSE treatment. The findings of this study indicate no significant difference in Cobb angle changes (D-value of Cobb angles) between groups after regular PSSE for two years, regardless of the curve pattern. This implies that the curve pattern does not significantly impact the effects of PSSE in participants with AIS. A previous 6-month long study suggested that the curve pattern, a thoracic curvature with an imbalanced pelvis (n=15), is significantly correlated with the largest sum of curves after Schroth exercise, and that study further explained this might be due to a higher risk of progression in thoracic scoliosis.[12] However, with a short-term follow-up and limited sample size, that study could not draw any firm conclusions on the relationship of curve pattern and Schroth exercise in treating AIS. Our results extend this discussion with a longer follow-up period, and curve patterns were controlled before PSSE commenced. In this study, no curve progression was observed in either group, suggesting that a well-planned PSSE program can prevent scoliosis deterioration regardless of curve patterns. This result is consistent with previous findings that PSSE reduces curve progression in patients with mild curve.[30-32] Compared with those studies, we observed regression effects in larger curves and with a longer follow-up period. Moreover, participants in our study presented a higher progression risk (LCR-value) at the study initiation; additionally, participants with brace treatment and all received the comparable in-brace

correction of 37% improvement. The lack of deterioration may be attributable to the effects of both bracing and PSSE; however, because comparable in-brace correction explained the effects of bracing, the effects from the PSSE could be highlighted in our study. Therefore, our results revealed the superiority of PSSE programs in curve stabilization and regression of scoliosis for patients with mild to moderate scoliosis. However, a risk of overtreatment exists because patients with only mild curves may not experience progression. Therefore, the burden of treatment should be avoided as much as possible through periodical~~ly~~ assessments.[12] Optimal time points for assessment require future study.

After two years of follow-up, no clinical deterioration was detected in either group, indicating a promising effect of PSSE on the prevention of scoliosis progression. Moreover, the regression effect accompanied by a notable decease of progression risk after 2 years. In particular, progression risk decreased to 12% in the thoracic curves and 11% in the lumbar curves, respectively. Moreover, ~~in which~~ 67% of them presented <30 degrees of Cobb with Risser 5 after two years of treatment. This is promising because of curve less than 30 degrees ~~was~~ unlikely to progress after skeletal maturity.[2] In particular, more participants in the major thoracic group showed curve regression in the first year (Table 2), but a decrease in compliance at the second year may have led to less of a regression effect at the 2-year follow-up. By contrast, a significant reduction in the Cobb angle was noted for participants with the major lumbar curves; moreover, a difference was observed as early as one and a half years after training, and reductions were maintained at the end of the study period. This was parallel with our original study hypothesis that major lumbar curves benefited more

from long-term exercise in terms of having a lower progression risk; however, this should be interpreted with caution because the progression value was comparable between groups at the study initiation. Hence, this observed promising result with lumbar curves might be explained by the better flexibility of the lumbar spine, confounding with a better exercise compliance and its associated effects of good core lumbar muscle stabilization. The superior flexibility of the lumbar spine compared to the thoracic spine has been proven in biomechanical studies[33-35]. The lumbar spine is mainly stabilized by core muscles that are flexible, stretchable and respond to external forces very well, whereas the stabilization mechanism in the thoracic spine is reliant on the rib cage, which contributes to rigidity and stability[34-36]. Additionally, according to the general principles of the Schroth method, correcting directions including side shift, axial elongation and derotation are created by muscle lengthening and eccentric contraction in the lumbar region, whereas spiral breathing is adopted in the thoracic region for correction[16]. Spiral breathing is difficult to learn and hard to control for most participants with AIS due to the requirement of breathing diagonally and asymmetrically not only in the frontal plane but also in the horizontal plane. Therefore, it is relatively easier for participants to correct the lumbar curvature via pelvic positioning, lengthening the curve concavity and stabilizing it in the proper position. In addition, stabilization effects from core muscles are crucial for maintaining correction in lumbar spines[16, 36]. Therefore, the lumbar curvature may respond better to the three-dimensional correction forces from a musculoskeletal functional perspective; however, real-time feedback of the spine during different corrective exercises requires future study.

In our study, significant differences between groups in exercise compliance were noted in the second year. Higher compliance in the major lumbar curve might contribute to the larger Cobb angle reduction. This extends the previous long-term studies of mild scoliosis that reported the only exercise intensity without reporting compliance.[15, 37] These previous studies have not addressed the relationship of compliance and treatment effects, which is of clinical valuable to define exercise protocols for subjects with PSSE treatment. In addition, our study revealed that an outpatient home-based PSSE program is a feasible protocol with reasonable compliance. The parents were asked to send monthly videos documenting the exercises performed to the Schroth therapist, which provided a reasonable variation control with respect to exercise quality and compliance. The frequency of such home-based programs was collected via a treatment adherence checklist, and exercise compliance was interpreted by how long participants spent on the PSSE program. This study calculated the supervised PSSE sessions and home exercise sessions in a sum number, in form of the total hours per week. Hence, we were unable to analyze specifically whether supervised PSSE is superior to home exercises in curve correction despite previous accounts of such a relationship[30-32]. Besides, the bracing compliance was self-reported and this might introduce recall effects, which can be improved if using a pressure sensor to monitor bracing hours. Nonetheless, only 10 participants and 5 of them were wearing nighttime brace only, hence, bracing would not cause a big distraction in our study.

This study had few inevitable limitations in addition to the small sample size. First, no untreated control group was included for comparison of intervention effects. However, because of

the proven benefits of PSSE on AIS [11-13], it was unethical to leave participants without treatment for as long as 2 years. Thus, the benefit observed may have been influenced by natural recovery of the condition. However, the progression risk at study initiation was evaluated with approximately 45% of progression rate, and with a moderate Cobb angles of 29 degrees with major thoracic and 27 degrees with major lumbar curves, hence, the natural recovery was unlikely in our study. Second, we only categorized participants into two groups, yet there were five scoliosis types according to the BSPTS concept, including 3C, 4C, N3N4, single lumbar/thoracolumbar and double thoracic.[22, 23] For participants having a double curve type such as N3N4 or 4C, the minor curve will cause limits to overstretching and twisting of the concavity from the major curve that weaken the effects of correction in the major curve. However, the proportion of curves pattern in this study was similar (Table 1: 40.9% vs 44.4%) between the two groups, which could not cause large variations in the data. Besides, there were only 6 participants with single right thoracic curve and no double upper thoracic curves were presented in this study, therefore, it would decrease the observed power if we extra analysis the single thoracic curves independently. However, a comparison of the effects of PSSE for all five curve types separately remains valuable for further study. Third, we were unable to blind the participants or physiotherapists due to the nature of PSSE studies, the exercise was prescribed and performed based on the understanding of the curve pattern by both therapists and patients, which may introduce methodological bias but also limited systematic errors such as learning effects. Performing exercise precisely is crucial to reach and maintain treatment effect, therefore, blinding of subjects and therapists was commonly sacrificed in most PSSE studies.[10, 11] However, the frequency and dosage of both supervised and home programs were strictly fixed, and we blinded the result assessor which controlled bias of statically analysis. Finally, we

hypothesized that the lumbar spine responds more to PSSE because of its lower progression rate and higher segmental spinal flexibility relative to the thoracic spine. However, because of radiation exposure concerns, no bending radiographic assessment conducted to quantify spinal flexibility before the PSSE program. Nonetheless, this study excluded participants with hypermobility that may cause systematic error during exercise. This controlled for potential heterogeneity when the spine started to respond to exercise. However, future study should address the relationship of spinal flexibility with PSSE, to define the correlation of curve location, segmental flexibility and PSSE in treating AIS.

Conclusion

PSSE treatment outcomes did not differ by thoracic or lumbar curve type. In general, PSSE with reasonable exercise compliance has positive effects on preventing scoliosis progression. However, further studies are necessary to address the correlation between spinal flexibility and the correction effects of PSSE ~~at~~ different curve magnitudes, curve types and levels involved. The cosmetic and quality of life outcomes of PSSE should also be studied. This study formed the base for future larger scale studies and randomized trials.

Supporting information

S1 File. Study protocol. (PDF)

S2 Fig. CONSORT flow diagram. (DOCX)

S3 File. Treatment adherence checklist. (PDF)

S4 File. Study data. (XLSX)

Acknowledgments

This study was financially supported by the Sanming Project of Medicine (SZSM201612055) “Team of Excellence in Spinal Deformities and Spinal Degeneration Diseases” and the Seeding Project (HKUSZH201902042) of the HKU - SZH in Shenzhen, Guangdong Province, China.

Authors’ contributions

YF and JPYC designed the study. JW and HR recruited participants. YF and EHKY performed PSSE teaching. ZX, GL and RZ collected data. MKTT performed statistics. YF drafted the manuscript. JPYC supervised the study and final edited the manuscript. MKTT, KMCC and JPYC obtained funding support. All authors read and approved the submitted version of the manuscript.

References:

1. Weinstein SL, Dolan LA, Cheng JC, Danielsson A, Morcuende JA. Adolescent idiopathic scoliosis. Lancet. 2008;371(9623):1527-37. Epub 2008/05/06. doi: 10.1016/s0140-6736(08)60658-3. PubMed PMID: 18456103.

2. Weinstein SL. The Natural History of Adolescent Idiopathic Scoliosis. J Pediatr Orthop. 2019;39(Issue 6, Supplement 1 Suppl 1):S44-s6. Epub 2019/06/07. doi: 10.1097/bpo.0000000000001350. PubMed PMID: 31169647.

3. Coe JD, Arlet V, Donaldson W, Berven S, Hanson DS, Mudiyam R, et al. Complications in spinal fusion for adolescent idiopathic scoliosis in the new millennium. A report of the Scoliosis Research Society Morbidity and Mortality Committee. Spine (Phila Pa 1976). 2006;31(3):345-9.

4. Korbel K, Kozinoga M, Stoliński Ł, Kotwicki T. Scoliosis Research Society (SRS) criteria and Society of Scoliosis Orthopaedic and Rehabilitation Treatment (SOSORT) 2008 guidelines in non-operative treatment of idiopathic scoliosis. Polish orthopedics and traumatology. 2014;79:118-22.

5. Dolan LA, Weinstein SL, Abel MF, Bosch PP, Dobbs MB, Farber TO, et al. Bracing in Adolescent Idiopathic Scoliosis Trial (BrAIST): Development and Validation of a Prognostic Model in Untreated Adolescent Idiopathic Scoliosis Using the Simplified Skeletal Maturity System. Spine Deform. 2019;7(6):890-8. e4.

6. Almansour H, Pepke W, Bruckner T, Diebo BG, Akbar M. Three-Dimensional Analysis of Initial Brace Correction in the Setting of Adolescent Idiopathic Scoliosis. Journal of clinical medicine. 2019;8(11):1804.

7. Thompson RM, Hubbard EW, Jo CH, Virostek D, Karol LA. Brace Success Is Related to Curve Type in Patients with Adolescent Idiopathic Scoliosis. J Bone Joint Surg Am. 2017;99(11):923-8. Epub 2017/06/08. doi: 10.2106/jbjs.16.01050. PubMed PMID: 28590377.

8. Negrini S, Carabalona R. Social acceptability of treatments for adolescent idiopathic scoliosis: a cross-sectional study. Scoliosis. 2006;1:14. Epub 2006/08/26. doi: 10.1186/1748-7161-1-14. PubMed PMID: 16930488; PubMed Central PMCID: PMCPMC1560163.

9. Burger M, Coetzee W, du Plessis LZ, Geldenhuys L, Joubert F, Myburgh E, et al. The effectiveness of Schroth exercises in adolescents with idiopathic scoliosis: A systematic review and meta-analysis. S Afr J Physiother. 2019;75(1):904. Epub 2019/06/18. doi: 10.4102/sajp.v75i1.904. PubMed PMID: 31206094; PubMed Central PMCID: PMCPMC6556933.

10. Day JM, Fletcher J, Coghlan M, Ravine T. Review of scoliosis-specific exercise methods used to correct adolescent idiopathic scoliosis. Arch Physiother. 2019;9:8. Epub 2019/08/30. doi: 10.1186/s40945-019-0060-9. PubMed PMID: 31463082; PubMed Central PMCID: PMCPMC6708126.

11. Thompson JY, Williamson EM, Williams MA, Heine PJ, Lamb SE. Effectiveness of scoliosis-specific exercises for adolescent idiopathic scoliosis compared with other non-surgical interventions: a systematic review and meta-analysis. Physiotherapy. 2019;105(2):214-34. Epub 2019/03/03. doi: 10.1016/j.physio.2018.10.004. PubMed PMID: 30824243.

12. Schreiber S, Parent EC, Khodayari Moez E, Hedden DM, Hill DL, Moreau M, et al. Schroth Physiotherapeutic Scoliosis-Specific Exercises Added to the Standard of Care Lead to Better Cobb Angle Outcomes in Adolescents with Idiopathic Scoliosis - an Assessor and Statistician Blinded Randomized Controlled Trial. PLoS One. 2016;11(12):e0168746. Epub 2016/12/30. doi: 10.1371/journal.pone.0168746. PubMed PMID: 28033399; PubMed Central PMCID: PMCPMC5198985.

13. Yagci G, Ayhan C, Yakut Y. Effectiveness of basic body awareness therapy in adolescents with idiopathic scoliosis: A randomized controlled study1. J Back Musculoskelet Rehabil. 2018;31(4):693-701. Epub 2018/04/10. doi: 10.3233/bmr-170868. PubMed PMID: 29630516.

14. Yagci G, Yakut Y. Core stabilization exercises versus scoliosis-specific exercises in moderate idiopathic scoliosis treatment. Prosthet Orthot Int. 2019;43(3):301-8. Epub 2019/01/11. doi: 10.1177/0309364618820144. PubMed PMID: 30628526.

15. Liu D, Huang S, Yu X, Yang J, Xuan X, Yang J, et al. Effects of Specific Exercise Therapy on Adolescent Patients with Idiopathic Scoliosis: A Prospective Controlled Cohort Study. Spine (Phila Pa 1976). 2020. Epub 2020/02/26. doi: 10.1097/brs.0000000000003451. PubMed PMID: 32097267.

16. Lehnert-Schroth C. [Schroth's three dimensional treatment of scoliosis]. ZFA (Stuttgart). 1979;55(34):1969-76. Epub 1979/12/10. PubMed PMID: 547573.

17. Lehnert-Schroth C. [The "four-curved" scoliosis. The effect on the additional lumbosacral spinal curve by the three dimensional Schroth's scoliosis treatment]. ZFA (Stuttgart). 1981;57(32):2227-31. Epub 1981/11/20. PubMed PMID: 7314859.

18. Little DG, Sussman MD. The Risser sign: a critical analysis. Journal of Pediatric Orthopaedics. 1994;14(5):569-75.

19. Cobb JR. Scoliosis—Quo Vadis? JBJS. 1958;40(3):507-10.

20. Smits-Engelsman B, Klerks M, Kirby A. Beighton score: a valid measure for generalized hypermobility in children. J Pediatr. 2011;158(1):119-23, 23.e1-4. Epub 2010/09/21. doi: 10.1016/j.jpeds.2010.07.021. PubMed PMID: 20850761.

21. Czaprowski D, Kotwicki T, Pawłowska P, Stoliński L. Joint hypermobility in children with idiopathic scoliosis: SOSORT award 2011 winner. Scoliosis. 2011;6(1):22.

22. Berdishevsky H, Lebel VA, Bettany-Saltikov J, Rigo M, Lebel A, Hennes A, et al. Physiotherapy scoliosis-specific exercises–a comprehensive review of seven major schools. Scoliosis Spinal Disord. 2016;11(1):20.

23. Rigo MD, Villagrasa M, Gallo D. A specific scoliosis classification correlating with brace treatment: description and reliability. Scoliosis. 2010;5(1):1.

24. Negrini S, Atanasio S, Fusco C, Zaina F. Effectiveness of complete conservative treatment for adolescent idiopathic scoliosis (bracing and exercises) based on SOSORT management criteria: results according to the SRS criteria for bracing studies-SOSORT Award 2009 Winner. Scoliosis. 2009;4(1):19.

25. Malfair D, Flemming AK, Dvorak MF, Munk PL, Vertinsky AT, Heran MK, et al. Radiographic evaluation of scoliosis. American Journal of Roentgenology. 2010;194(3_supplement):S8-S22.

26. Fitzmaurice GM, Laird NM, Ware JH. Applied longitudinal analysis: John Wiley & Sons; 2012.

27. Lonstein J, Carlson J. The prediction of curve progression in untreated idiopathic scoliosis. J Bone Jt Surg. 1984;3(2):1061-71.

28. Barnes P, Brody J, Jaramillo D, Akbar J, Emans J. Atypical idiopathic scoliosis: MR imaging evaluation.

Radiology. 1993;186(1):247-53.

29. Wang W, Zhu Z, Zhu F, Sun C, Wang Z, Sun X, et al. Different curve pattern and other radiographical characteristics in male and female patients with adolescent idiopathic scoliosis. Spine (Phila Pa 1976). 2012;37(18):1586-92.

30. Weiss HR, Lohschmidt K, el-Obeidi N, Verres C. Preliminary results and worst-case analysis of in patient scoliosis rehabilitation. Pediatr Rehabil. 1997;1(1):35-40. Epub 1997/01/01. doi: 10.3109/17518429709060940. PubMed PMID: 9689236.

31. Weiss HR, Weiss G. Curvature progression in patients treated with scoliosis in-patient rehabilitation--a sex and age matched controlled study. Stud Health Technol Inform. 2002;91:352-6. Epub 2004/10/02. PubMed PMID: 15457754.

32. Weiss HR, Weiss G, Petermann F. Incidence of curvature progression in idiopathic scoliosis patients treated with scoliosis in-patient rehabilitation (SIR): an age- and sex-matched controlled study. Pediatr Rehabil. 2003;6(1):23-30. Epub 2003/05/15. doi: 10.1080/1363849031000095288. PubMed PMID: 12745892.

33. Hajihosseinali M, Arjmand N, Shirazi-Adl A, Farahmand F, Ghiasi MS. A novel stability and kinematics-driven trunk biomechanical model to estimate muscle and spinal forces. Medical engineering & physics. 2014;36(10):1296-304. Epub 2014/07/31. doi: 10.1016/j.medengphy.2014.07.009. PubMed PMID: 25074649.

34. Oxland TR. Fundamental biomechanics of the spine--What we have learned in the past 25 years and future directions. Journal of biomechanics. 2016;49(6):817-32. Epub 2015/12/27. doi: 10.1016/j.jbiomech.2015.10.035. PubMed PMID: 26706717.

35. Panjabi MM, White AA, 3rd. Basic biomechanics of the spine. Neurosurgery. 1980;7(1):76-93. Epub 1980/07/01. doi: 10.1227/00006123-198007000-00014. PubMed PMID: 7413053.

36. Eskandari AH, Arjmand N, Shirazi-Adl A, Farahmand F. Subject-specific 2D/3D image registration and kinematics-driven musculoskeletal model of the spine. Journal of biomechanics. 2017;57:18-26. Epub 2017/04/04. doi: 10.1016/j.jbiomech.2017.03.011. PubMed PMID: 28365064.

37. Monticone M, Ambrosini E, Cazzaniga D, Rocca B, Ferrante S. Active self-correction and task-oriented exercises reduce spinal deformity and improve quality of life in subjects with mild adolescent idiopathic scoliosis. Results of a randomised controlled trial. Eur Spine J. 2014;23(6):1204-14. Epub 2014/04/01. doi: 10.1007/s00586-014-3241-y. PubMed PMID: 24682356.

S1 File_Study protocol

Click here to access/download

Supporting Information

S1_File.pdf

S2 Figure_CONSORT flow diagram

Click here to access/download
Supporting Information
S2_Fig..doc

S3 File_Home Exercise adherence checklist

Click here to access/download
Supporting Information
S3_File.pdf

S4 File_raw data set

Click here to access/download
Supporting Information
S4_File.xlsx

S5 File_SPSSoutput

Click here to access/download
Supporting Information
S5_SPSSoutputs.rtf

TREND checklist

Click here to access/download
Supporting Information
S6_File_trendstatement_TREND_Checklist.pdf

Click here to access/download
Supporting Information
renamed_b0e5d.pdf

Protocol in English

Click here to access/download
Supporting Information
StudyProtocolEnglish.pdf

Registation information

Click here to access/download
Supporting Information
Registration.pdf

Registration infor

Click here to access/download
Supporting Information
ChiCtr202001211451448789.xml

Title: Dose curve pattern impact on the effects of physiotherapeutic scoliosis specific exercises on Cobb angles of participants with adolescent idiopathic scoliosis: a prospective clinical trial with two years follow-up

Authors

1. Yunli Fan[1,2,3]
2. Michael KT To[2]
3. Eric HK Yeung[3]
4. Jianbin Wu[1]
5. Rong He[3]
6. Zhuoman Xu[3]
7. Ruiwen Zhang[3]
8. Guangshuo Li[3]
9. Kenneth MC Cheung[2]
10. Jason PY Cheung[2*]

[1]Department of Orthopedics, The University of Hong Kong – Shenzhen Hospital, Shenzhen, Guangdong Province, People’s Republic of China

[2]Department of Orthopaedics and Traumatology, The University of Hong Kong, Hong Kong SAR, China

[3]Department of Physiotherapy, The University of Hong Kong – Shenzhen Hospital, Shenzhen, Guangdong Province, People’s Republic of China

*** Corresponding author:**

Name	Cheung, Jason Pui Yin
Department	Department of Orthopaedics and Traumatology
Institution	The University of Hong Kong
Country	Hong Kong Special Administration Region, China
Email	cheungjp@hku.hk

Dose curve pattern impact on the effects of physiotherapeutic scoliosis specific exercises on Cobb angles of participants with adolescent idiopathic scoliosis: a prospective clinical trial with two years follow-up

Yunli Fan[1,2,3], Michael KT To[2], Eric HK Yeung[3], Jianbin Wu[1], Rong He[3], Zhuoman Xu[3], Ruiwen Zhang[3], Guangshuo Li[3], Kenneth MC Cheung[2], Jason PY Cheung[2*]

Abstract

Background

Current clinical evidence suggests that a well-planned physiotherapeutic scoliosis specific exercise (PSSE) program is effective for scoliosis regression.

Objectives

We investigated the effect of curve patterns on Cobb angles with PSSE.

Methods

This was a prospective clinical trial that recruited participants with adolescent idiopathic scoliosis between January and June 2017. Participants were grouped by curve pattern into major thoracic and major lumbar groups. An outpatient-based PSSE program was conducted with the following schedule of intensive exercise: ≥ 1 session of supervised PSSE per month and > 30min of home exercise 5 days/week in the first 6 months, after which exercise frequency was reduced to

1 session of supervised PSSE every three months and > 30min of home exercise 5 days/week until 2 years after study initiation. Radiographic Cobb angle progressions were identified at the 1, 1.5 and 2-year follow-ups. One-way mixed repeated measures analyses of variance were performed to examine the differences in Cobb angles between groups at four testing time points. The two-tailed significance level was set to 0.05.

Results

In total, 40 participants were recruited, including 22 with major thoracic curves (5 male and 17 female; mean age 13.5±1.8 years; Cobb angle 18 - 45 degrees) and 18 with major lumbar curves (7 male and 11 female; mean age 12.7±1.7 years; Cobb angle 15 - 48 degrees). Curve regressions, namely the reduction of Cobb angles between 7 to 10 degrees were noted in 9.1% of participants in the major thoracic group; reductions of 6 to 13 degrees were noted in 33.3% of participants in the major lumbar group at the 2-year follow-up. Repeated measurements revealed a significant time effect ($F_{2.2,81.9}$=6.9, p=0.001), but no group ($F_{2.2,81.9}$=2.2, p=0.1) or time × group ($F_{1,38}$=1.4, p=0.2) effects in reducing Cobb angles were observed after 2 years of PSSE. In addition, 65% of participants (n=26) reached skeletal maturity (Risser sign = 5) and achieved significant reductions in progression risk at the 2-year follow-up (Lonstein and Carlson Risk of progression: 0.45 ± 0.32 vs. 0.13 ± 0.14, p <0.001).

Conclusion

This was the first study to investigate the long-term effects of PSSE in reducing Cobb angles on the basis of major curve location. No significant differences in correction were observed between major thoracic and major lumbar curves. However, a regression effect and no curve deterioration were noted in both groups at the 2-year follow-up.

Trial Registration: ChiCTR1900028073

*** Corresponding author:** cheungjp@hku.hk

[1]Department of Orthopedics, The University of Hong Kong – Shenzhen Hospital, Shenzhen, Guangdong Province, People's Republic of China

[2]Department of Orthopaedics and Traumatology, The University of Hong Kong, Hong Kong SAR, China

[3]Department of Physiotherapy, The University of Hong Kong – Shenzhen Hospital, Shenzhen, Guangdong Province, People's Republic of China

Introduction

Adolescent idiopathic scoliosis (AIS) is a three-dimensional spinal deformity with an unknown etiology, characterized by lateral deviation in the frontal plane, axial rotation in the horizontal plane and an abnormal sagittal curvature.[1] Surgery is typically recommended if a spinal curve reaches 50 degrees, because such curves are associated with a continued progression risk into

adulthood.[1-3] Surgical fusion causes spine spine stiffness and should be avoided if possible.[3] Thus, the goal of conservative managements including bracing and exercises, is to prevent spinal deformity deterioration past the operative threshold.[4]

Options for nonsurgical management vary widely and depend on the prognostic evaluation of curve progression.[4] Curve type is an established risk factors of scoliosis progression.[5] In particular, a thoracic curve with a larger Cobb angle has a higher likehood of progression than single lumbar or thoracolumbar curves do.[2] Therefore, understanding the effect of curvature on interventions can help clinicians select appropriate treatments for patients.

Bracing is commonly prescribed for moderate scoliosis, and its corrective effect is influenced by the location of structural curves.[6, 7] In addition, scoliosis-specific exercise is a nonoperative option that is well received by patients and families.[8] Several systematic reviews and randomized controlled trials have reported the positive effects of physiotherapeutic scoliosis specific exercise (PSSE) on slowing curve progression as well as improving cosmetic and quality of life;[9-11] however, the relationship of curve location with correction effects has not been clearly discussed in these studies, in which small samples of participants have been recruited and followed up over the short-term.[12-14] In particular, one 6-month long randomized control trial (RCT) revealed a significant correlation between thoracic curvature with an imbalanced pelvis (n=15) and the largest curves after Schroth treatment;[12] one 4-month long RCT demonstrated the greater Cobb reduction was noted in the thoracic region (n=20) after body awareness exercise.[13]

Therefore, a clinical trial, in which curve pattern is controlled, is warranted to investigate the long-term effects of curve pattern and PSSE on reducing Cobb angles. This is valuable for clinical practice because the deterioration is possible after initial improvement in short-term follow-up.

According to current evidence from PSSE studies,[9, 10, 15] the Schroth is the most used PSSE approach. It adopts specific respiratory technique-asymmetrical breathing in the diagonal direction to achieve vertebral and rib cage derotation.[16] It utilizes muscle activation and emphasizes core muscle stabilization in a corrected posture throughout the day to change habitual postures and improve spinal alignment.[16, 17] However, the influence of curve magnitude on Schroth method outcomes is unclear. The spinal deformity profile that leads to optimal outcomes with Schroth exercise is also unclear. Thus, this study investigated the long-term therapeutic effects of Schroth exercises on major thoracic and major lumbar curves, respectively. We hypothesized that patients with lumbar curves, which have a lower reported risk of curve progression and higher spinal segmental flexibility, benefit more from long-term PSSE treatment than do patients with major thoracic curves. The current results can provide physiotherapists with additional information to develop individualized exercises based on curve magnitude. These results can also form the basis for further RCTs evaluating the relationship between curve patterns and PSSEs.

Materials and methods

Study design

This was a prospective clinical trial in which participants with AIS were recruited consecutively between January and June 2017 from one scoliosis clinic at the University of Hong Kong - Shenzhen Hospital (HKU-SZH), China. This study obtained ethical approval by the institutional review board of the HKU-SZH with reference number: GNSF201603 (**S1** File). Individuals in this manuscript have given written informed consent to publish these case details. The authors confirm that all ongoing and related trials for this intervention are registered in the Chinese clinical trial registry (www.chictr.org.cn, trail registration: ChiCTR1900028073) (**S1** File). Participants and legal guardians signed a written informed consent for PSSE treatment and participants were followed up for 2 years thereafter. The inclusion criteria were age 10 to 16 years; bone immaturity in terms of Risser sign was less than 5;[18] Cobb angles[19] from 10 degrees to 50 degrees; and complete attendance for all physiotherapy sessions. Exclusion criteria were diagnoses other than AIS, disabilities or systemic illnesses that prevent participants from performing exercise, hypermobility (Beighton score[20] greater than 4) and previous treatment for AIS. Joint hypermobility (JHM) is reported to have a higher prevalence in patients with single curves than in those with double-curve scoliosis; and this may affect physiotherapy outcomes.[21] Therefore, patients with hypermobility were excluded from this study to control for systematic error introduced by JHM differences between curve patterns. Participants were categorized into two groups according to their whole spine radiographic characteristics:[22, 23] participants with 3C (major thoracic with or without a minor lumbar curve) or N3N4 curves (double, well balanced curves) formed the major thoracic group; and those with single lumbar, single thoracolumbar, or 4C curves (a major lumbar curve with a minor thoracic) comprised the major lumbar group (**S2** Fig 1).

S2 Fig 1. CONSORT flow diagram.

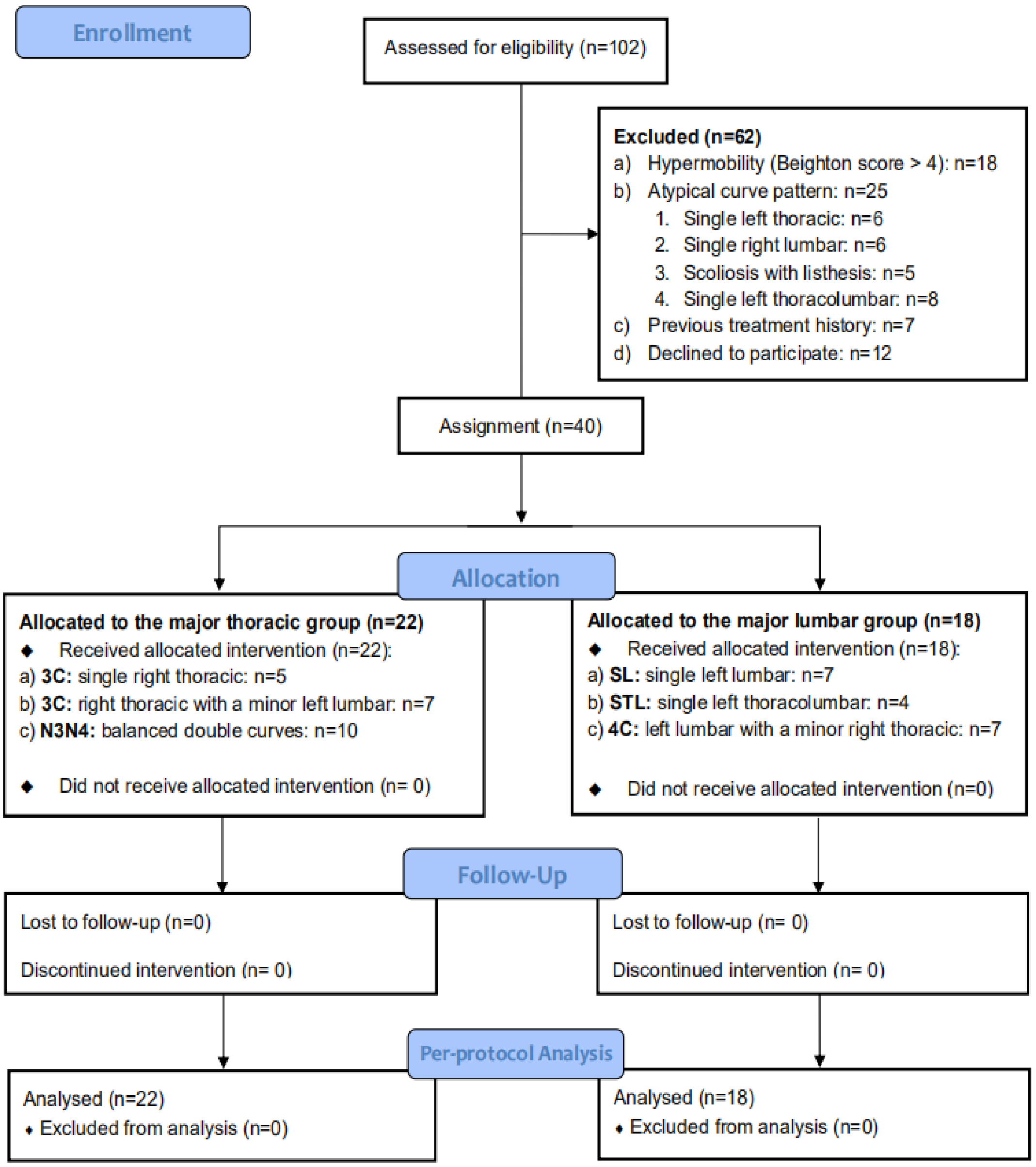

Study intervention

All participants participated in a 6-month intensive PSSE program supervised by two

PSSE-Schroth certified physiotherapists (Barcelona Scoliosis Physical Therapy School [BSPTS] approach).[22] Supervised treatment during the 6 months occurred ≥1 time per month for 1 hour each time. Participants were instructed to perform >30min sessions of home exercise at least ≥5 times per week. For participants who met Scoliosis Research Society bracing criteria, Cheneau bracing was prescribed prior to PSSE program;[24] in-brace correction was documented by a spine surgeon who was blinded to this study. Bracing compliance was reported by patients on their monthly treatment adherence checklist (**S3** File). After the first 6 months of PSSE, all participants reduced their frequency of supervised PSSE to once every 3 months to review their exercises, which they performed for 2 years after completing the PSSE program. During this time, the frequency of home exercise was maintained. Attendance for supervised PSSE in an outpatient setting was recorded in the hospital's prospective database system. Participants were requested to document home exercise and bracing compliance on daily basis using a treatment adherence checklist (**S3** File), and handed it to a physiotherapist assistant who was blinded to this study every month during the first 6-month study period; then they were required to submit the checklist every 3 months thereafter. Exercise compliance was final calculated in hours per week per year for analysis. The PSSE intervention consisted of 50 breaths per exercise. Short semihanging with caudal-cranial spine lengthening (Fig 2. c), corrective exercise in side lying with shoulder counter traction (Fig 2. d), corrective exercise in sitting with pelvic pulling strategy on the side of weak point and shoulder counter traction (Fig 2. e), corrective exercise in standing with pelvic derotation and shoulder counter traction (Fig 2. f) were prescribed for participants in the major thoracic group. Short semihanging with caudal-cranial spine lengthening (Fig 3. c), corrective exercise in side lying with passive correction on the lumbar prominence (Fig 3. d), corrective exercise in standing with

shoulder counter traction (Fig 3. e), muscle cylinder in standing for opening lumbar concavity (Fig 3. f) and the corrective exercise in sitting with shoulder counter traction (Fig 3. g) were prescribed to participants in the major lumbar group. The difference between the two groups' exercises was that a muscle cylinder was prescribed only to participants with major lumbar curves. The muscle cylinder exercise in a kneeling position is typical prescribed to patients with major thoracic curves; however, patients commonly complain that it causes knee pain. Therefore, consider the potential adverse effects during long-term follow-up, the muscle cylinder in a kneeling position was not prescribed in this study. Moreover, dynamic exercise, such as Schroth walking was not used in this study because of limited space in our center. In addition, participants were asked to record and report any discomfort, such as muscle fatigue and muscle sprain, to the on-duty physiotherapist during the study period.

Fig 2. Exercises demonstration for major right thoracic curves

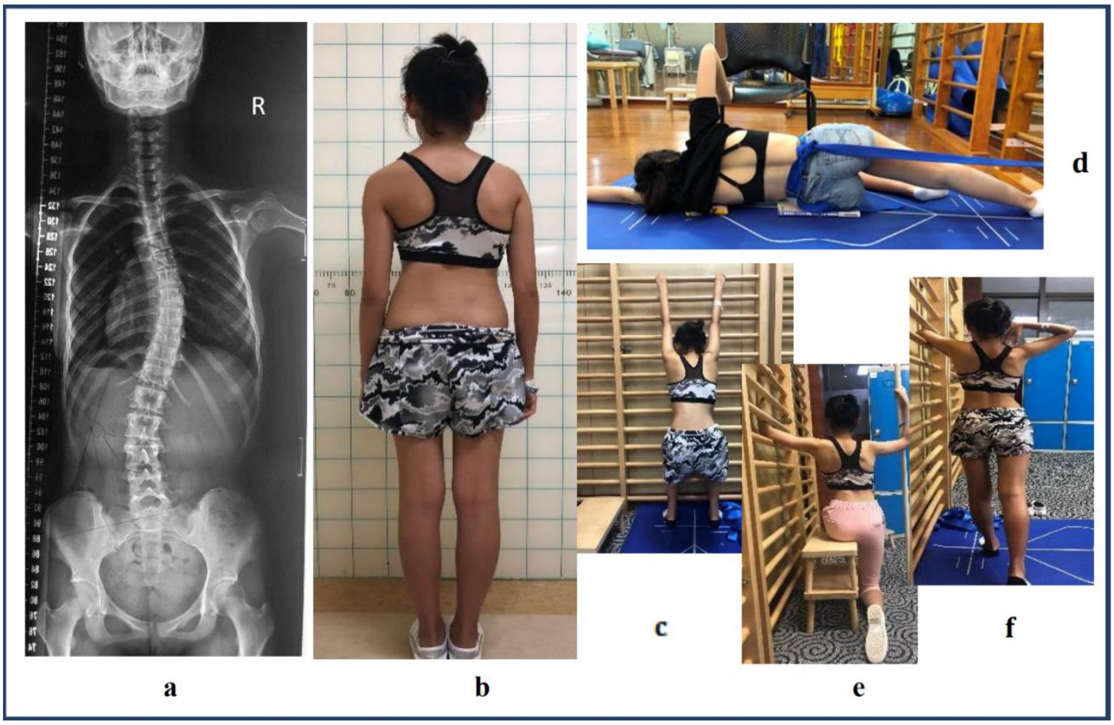

Fig 2: **a:** a major right thoracic (T4 to 12) with a Cobb angle of 30 degrees. **b:** posterior posture view of the participant. **c:** short semihanging with caudal-cranial spine lengthening. **d:** corrective exercise in side lying with shoulder counter traction. **e:** corrective exercise in sitting with pelvic pulling strategy on the side of weak point and shoulder counter traction. **f:** **c**orrective exercise in standing with pelvic derotation and shoulder counter traction.

Fig 3. Exercises demonstration for major left lumbar curves

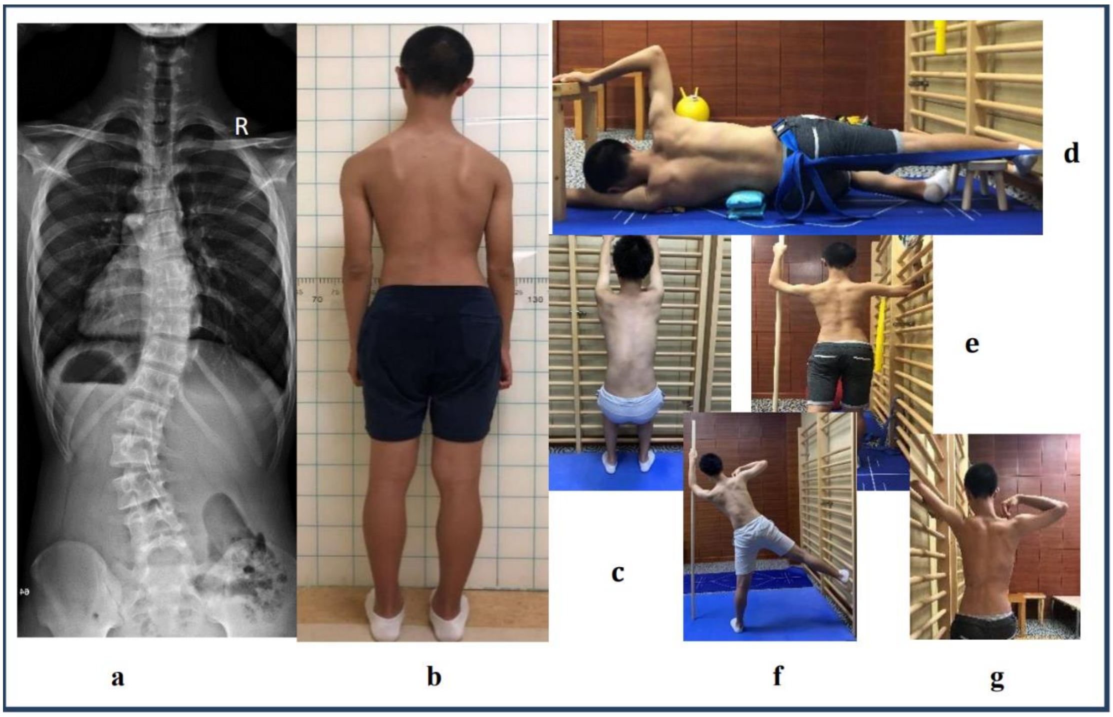

Fig 3: a: a major left lumbar curve (T12 to L4) with a Cobb angle of 48 degrees. **b:** posterior posture view of the participant. **c:** short semihanging with caudal-cranial spine lengthening. **d:** corrective exercise in side lying with passive correction on the lumbar prominence. **e:** corrective exercise in standing with shoulder counter traction. **f: m**uscle cylinder in standing for opening lumbar concavity. **g: c**orrective exercise in sitting with shoulder counter traction.

Outcome measurement

Participants underwent whole-spine radiography in a standing position with arms slightly abducted at the sides of body. The Cobb angles of major curves were assessed by a spine surgeon who was blinded to this study. Cobb angles were recorded in the initial assessment and every 6 months after the PSSE program until the 2-year follow-up. Participants remained out of their braces overnight prior to obtaining radiography. Curve regression was defined as a decrease in the Cobb

angle by 6 degrees or more, stabilization was defined as a <6 degrees change in Cobb angle, and deterioration was defined as a Cobb angle increase of 6 degrees or more.[2, 25]

Statistics

Sample size estimation

To obtain an effect size of 0.50, 80% power for a two-tailed test, and a correlation of 0.6 for repeated measures of Cobb angles over 4 time points, 13 participants were required for each group.[26] Considering possible 35% dropout rate during the 2-year study period, each group were planned to have 18 participants (**S2** Fig 1).

Statistical analysis

The baseline, continuous variables, including age, Lonstein and Carlson Risk of progression value (LCR-value)[27] and initial Cobb angles were evaluated using independent t test if assumptions of normality was met; otherwise the Kruskal-Wallis test was performed if normality assumptions were violated. The chi-square test was used to compare categorical variables including sex, Risser sign (stage: 0-2 or 3-4), curvature types (the single thoracic curve and the major thoracic with a minor lumbar curve vs the single lumbar/thoracolumbar and the major lumbar with a minor thoracic curve) and brace treatment (yes or no) for all participants (Table 1). Changes in Cobb angles, in the form of the difference value (D-value) between testing times, were measured to detect curve progression, stabilization and regression between groups; raw Cobb

angles were repeatedly compared over four testing time points to detect time, group and time × group interaction effects in reducing Cobb angles. One-way analysis of variance (ANOVA) was performed if the normality assumptions of Cobb angles were met whereas a nonparametric Friedman-type test would be performed if normality was violated. Post hoc pairwise comparison was conducted if any significant differences were detected in either within-subjects or between-subjects comparisons. A Logistic regression was performed to detect correlations of age, sex, brace treatment, curve pattern with the regression, stabilization or deterioration after 2 years of PSSE. The data were analyzed using SPSS version 20.0 (IBM, Chicago, IL). The level of significance was set to 0.05 with a two-tailed test.

Results

Participants

In total, 102 patients were screened to confirm eligibility, after which 62 patients were excluded for the following reasons: hypermobility (Beighton score > 4) was detected in 18 patients; 25 patients had atypical curve patterns[28, 29] (single left thoracic; n=6, single left lumbar: n=6; scoliosis was presented with vertebral listhesis: n=5, single left thoracolumbar: n=8); 7 patients were previously treated with either bracing or exercise; 12 patients declined to participate in this study. Therefore, 40 participants were recruited and all completed follow-up (**S2** Fig 1). The major thoracic group had 22 participants (5 males and 17 females). Of them, 12 had 3C curves (single right thoracic: n=5; right thoracic with a minor left lumbar: n=7) and 10 had N3N4 curves (balanced right thoracic with a left lumbar). The major lumbar group had 18 participants (7 males

and 11 females). Of them, 7 had single left lumbar curves, 4 had right thoracolumbar curves and 7 had 4C curves (left lumbar with a minor right thoracic). The mean age of participants in the major thoracic and major lumbar groups was 13.5±1.8 years and 12.7±1.7 years, respectively (Table 1). At baseline, the two groups demonstrated non significant difference in age, sex, brace treatment, Risser sign (0-2 / 3-4), proportion of sub-curvature types, LCR-value, or initial Cobb angle of the major curve (Table 1). Participants with brace treatment (n=10) who started Cheneau orthosis before commencing the PSSE and achieved comparable 37% of curve correction between groups (Table 1). In particular, 5 of them were wearing night-time brace only (≤ 8 hours/day) whereas 5 were wearing full-time brace (21 hours/day) (Table 1). In addition, two participants in the major lumbar group who refused brace treatment for cosmetic reasons.

Table 1. Baseline characteristics of participants

	**The major thoracic group** (95% confidence interval)	**The major lumbar group** (95% confidence interval)	***P* value**
**Age** (years: Mean±SD)	13.5 ± 1.8 (12.8 - 14.6)	12.7 ± 1.7 (12.6 - 13.7)	0.1
**Sex**			
Female	17 (77.3%)	11 (61.1%)	0.3
Male	5 (22.7%)	7 (38.9%)	

**Risser sign**			0.6
0-2 / 3-4	9 / 13	9 / 9	
**Initial Cobb Angle** (min-max)	18 - 45	15 - 48	0.6
(degrees:Mean±SD)	28.5 ± 8.4 (24.5 - 32.5)	26.8 ± 10.1 (22.4 - 31.2)	
**Curve pattern**			
3C / SL or STL	12	11	0.7
N3N4 / 4C	10	7	
**Braced** (yes)	7	3*	0.5
In-brace hours (hours/day)	13.6 ± 6.9	16.7 ± 7.5	
full-time brace (≥ 21hr/day)	3	2	
night-time brace (≥8hr/day)	4	1	
**In-brace Cobb angle**			
(degrees: Mean ± SD)	23 ± 4.4	26 ± 6	0.9
correction rate (%)	37	37	
**Progression risk**	44 %	46%	0.8
Lostein and Carlson risk			

**Exercise compliance**			
1-year: hours/week	5.5 ± 2.2	5.5 ± 2.1	0.9
2-year: hours/week	3.0 ± 0.8§	4.1± 2.1	**0.04**

3C: A single right thoracic curve or a major right thoracic with a minor left lumbar. **SL:** A single left lumbar. **STL:** A single right thoracolumbar. N3N4: double curves but well balanced. **4C:** A major left lumbar with a minor right thoracic. * Not including two participants who refused brace treatment. § significantly differ from the compliance value at the 1-year follow-up in the major thoracic group.

Exercise compliance

The two groups differed significantly in exercise compliance during the second year of follow-up (3.0±0.8 hours/week vs 4.1±2.1 hours/week, p=0.04). In addition, mean exercise compliance of the major thoracic group was significantly decreased in the second year (5.5±2.2 hours/week vs 3.0±0.8 hours/week; p<0.01); the mean exercise compliance of the major lumbar group was 5.5±2.1 hours/week in the first year and slightly decreased to 4.1±2.1 hours/week in the second year (Table 1). There were no adverse effects detected during this study period.

Intervention effects

In total, 23 participants (57.5%) with Cobb angle < 30 degrees refused to repeat

radiography at the 6-month follow-up after the PSSE program because of radiation exposure concerns; therefore, no comparison of participants before and at the 6-month follow-up was performed. Nonetheless, all participants completed radiography at the 1-year, 1.5-year and 2-year follow-up, respectively. Full set of raw data was available in the **S4** File.

No deterioration of the major curvature occurred in either group (both average and individual data) at the 2-year follow-up (Table 2). Specifically, curve regressions, reduction of Cobb angles between 7 to 10 degrees were noted in 9.1% of participants in the major thoracic group; reductions of 6 to 13 degrees were noted in 33.3% of participants in the major lumbar group (Table 2). However, no statistical effects of group ($F_{1.5,56.5}$=2.7, p=0.09) and time ($F_{1.5,56}$=1.2, p=0.3) or the interaction effect of time × group ($F_{1,38}$=1.8, p=0.2) with the D-value angles of Cobb were observed after 2 years of regular PSSE.

Table 2. Repeated analysis for the D-value of Cobb angles over three testing time points

**Testing times**	**Group**	**Mean**	**Std. Deviation**	**95% Confidence Interval**	**Min value**	**Max value**	**Regressed (n)**	**Stabilized (n)**	**Deteriorated (n)**
**1-year**	0	-1.5	4.7	-3.5 - 0.53	5	-12	4	18	0
	1	-2.1	4.8	-4.4 - 0.2	7	-13	2	15	1
	**Total**	**-1.8**	**4.7**	**-3.8 - 0.3**	**5**	**-13**	**6**	**33**	**1**
**1.5-year**	0	-1.4	4.7	-3.5 - 0.6	5	-15	3	19	0

	1	-3.5	4.9	-5.8 - -1.2	5	-13	6	12	0
	**Total**	**-2.4**	**4.8**	**-4.6 - 0.3**	**5**	**-15**	**9**	**31**	**0**
**2-year**	0	-1.0	4.2	-2.9 - 0.8	5	-10	2	20	0
	1	-3.8	4.3	-5.9 - -1.8	3	-13	6	12	0
	**Total**	**-2.3**	**4.4**	**-4.1- 1.3**	**3**	**-13**	**8**	**32**	**0**

Group 0: the major thoracic group. **Group 1:** the major lumbar group. **Regressed:** a decrease of Cobb angle ≥ 6 degrees. **Stabilized:** a change of Cobb angle < 6 degrees. **Deteriorated:** an increase of Cobb angle ≥ 6 degrees.

Per-protocol analysis was performed to analyze the raw value of Cobb angles over four testing time-points between groups. Mauchly test indicated that the assumption of sphericity was violated (χ^2 =32.5 , df =5 , p < 0.001); hence, the degrees of freedom were corrected using Greenhouse-Geisser estimates of sphericity. Repeated analysis showed a significant time effect ($F_{2.2,81.9}$=6.9, p=0.001), but no group ($F_{2.2,81.9}$=2.2, p=0.1) or time × group ($F_{1,38}$=1.4, p=0.2) effects were detected in reducing Cobb angles after 2 years of treatment (Table 3). In the major lumbar group, a reduction was noted in the Cobb angle (26.8±10.1 degrees vs 23.3±9.4 degrees, p=0.007) after training for one and a half years with a maintained reduction after two years of follow-up (26.8±10.1 degrees vs 22.9±9.2 degrees, p<0.001) (Table 3). In comparison to initial Cobb angles within participants with the major lumbar curve, the curves improved (Cobb angles: -9.5±4.9 degrees) in 11.1%, stabilized (Cobb angles: -3.4±1.2 degrees) in 83.3% and deteriorated by 7 degrees in one subject after the first year of training (Table 2). In the second year of follow-up, up to 33.3% of participants had improved Cobb angles (Cobb angles: -8.2±2.5 degrees), while 66.7%

stabilized (Cobb angles: -3.2±1.6 degrees), and no deterioration was noted (Table 2). Of the participants with curve regression in the first year, one maintained the curve regression, reducing Cobb angle of 13 degrees, and one only gained an additional degree in the second year. Moreover, one participant with a single left lumbar curve of 31 degrees (Fig. 4: a) who refused brace treatment but obtained in a Cobb angle of 23 degrees (Fig. 4: b) after one and a half years of PSSE. In addition, another participant with a single right thoracolumbar curve of 44 degrees (Fig. 5: a) who obtained a Cobb angle of 31 degrees (Fig. 5: b) after two years of PSSE.

Table 3. Per-protocol repeated measures of Cobb angles over four testing time points

**Testing timepoint**	**Group**	**Mean**	**Std. Deviation**	**95% Confidence Interval**	**Minimum**	**Maximum**	***P***
**Baseline**	0	28.5	8.4	24.5-32.5	18	45	-
	1	26.8	10.1	22.4-31.2	15	48	-
	Total	27.7	9.1	24.7-30.6	16	46	-
**1-year**	0	27	7.2	23.4-30.6	18	38	0.152
	1	24.7	9.4	20.8-28.7	10	42	0.079
	Total	**26.0**†	8.2	23.2-28.5	14	40	**0.022**
**1.5-year**	0	27.1	7.7	23.4-30.9	10	40	0.17
	1	**23.3***	9.4	19.2-27.3	10	45	**0.007**
	Total	**25.4**†	8.6	22.5-27.9	10	43	**0.002**
**2-year**	0	27.5	7.0	24.0-31.0	15	40	0.254

1	**22.9***	9.2	19.1-26.8	10	43	**0.001**
Total	**25.4ŧ**	8.2	22.6-27.8	13	42	**0.001**

Group 0: the major thoracic group. **Group 1:** the major lumbar group. ***** significantly differ from the baseline in the major lumbar group. ŧ significantly differ from the mean value at baseline.

Fig 4. Radiographic assessment (posterior anterior view) of one participant with a single left lumbar scoliosis who refused brace treatment for cosmetic reasons.

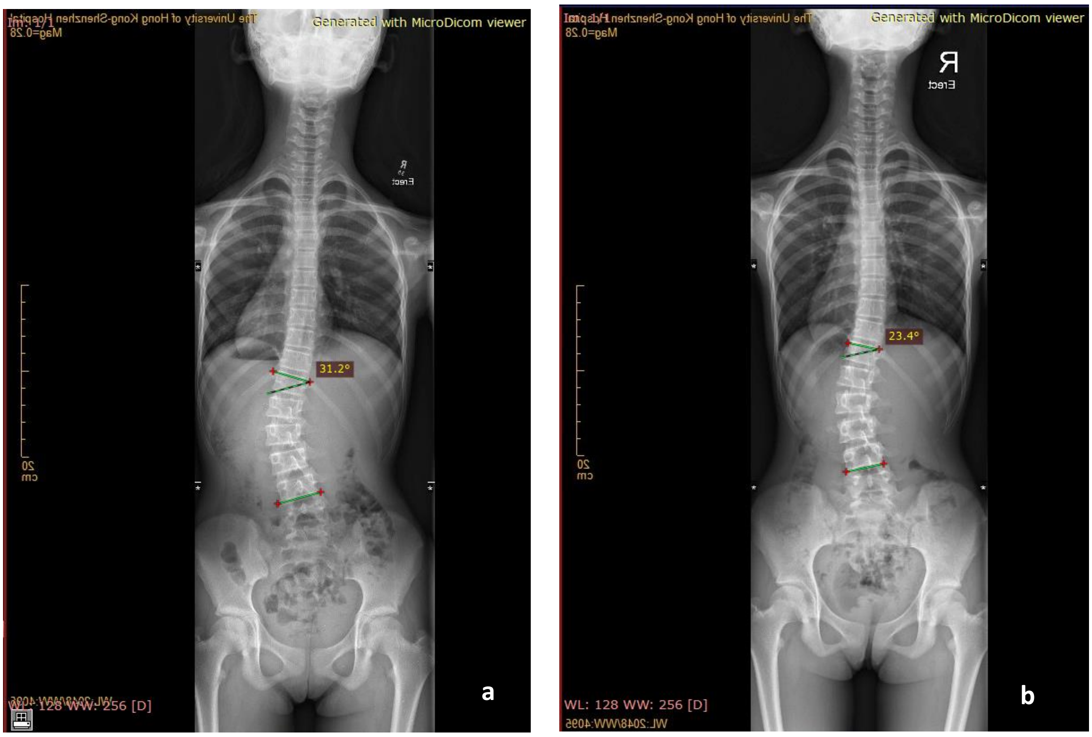

a: an initial Cobb angle of 31 degrees from T12 to L4. **b:** a Cobb angle of 23 degrees from T12 to L4 at the 1.5-year of follow-up.

Fig 5. Radiographic assessment (posterior anterior view) of one participant with a single right

thoracolumbar scoliosis who refused brace treatment for cosmetic reasons.

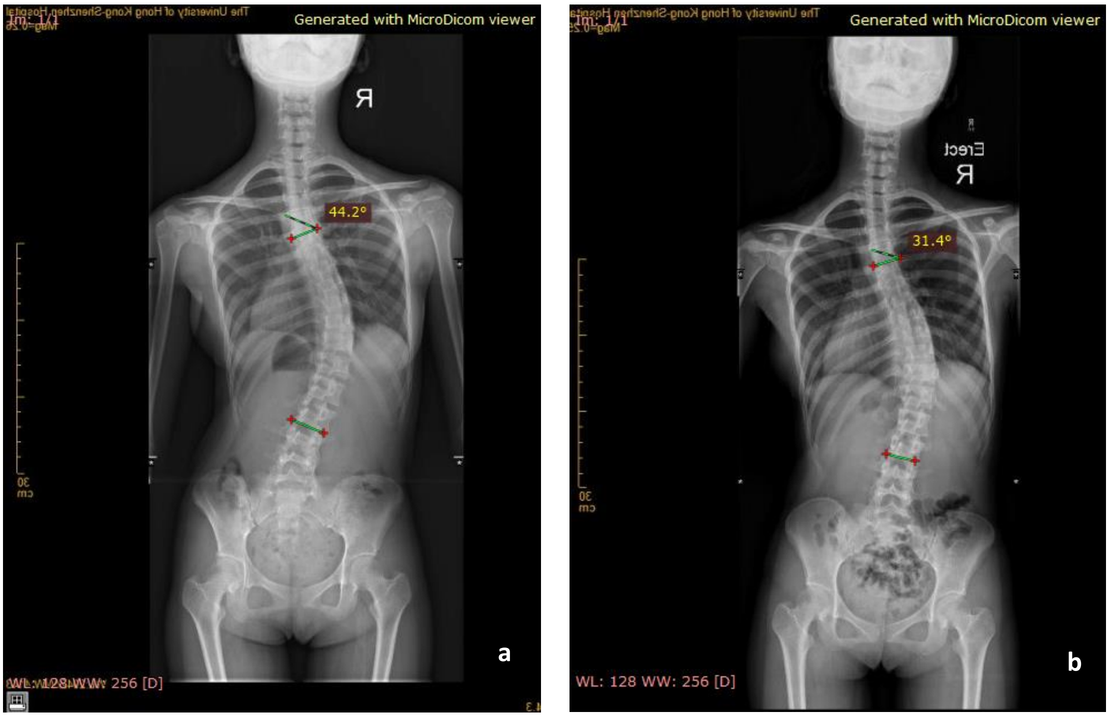

a: an initial Cobb angle of 44 degrees from T5 to L2. **b:** a Cobb angle of 31 degrees from T5 to L2 at the two-year follow-up.

In the major thoracic group, no difference was observed in Cobb angle (28.5±8.4 degrees vs 27.0±7.2 degrees, *p*=0.15; 28.5±8.4 degrees vs 27.1±7.7 degrees, *p*=0.17; 28.5±8.4 degrees vs 27.5±7.0 degrees, *p*=0.25) after one year, one and a half years and two years of follow-up, respectively (Table 3). However, after training, the spinal deformity improved (Cobb angles: -8.8±2.5 degrees) in 18.2% of participants and stabilized (Cobb angle: -2.9±1.6 degrees) in 81.8% of participants, and none deteriorated in the first year (Table 2). Only 2 participants maintained curve regression in the second year, which resulted 9.1% of participants showed regression (Cobb angles: -8.5±2.1 degrees) (Table 2). Thus, up to 90.9% of participants stabilized (Cobb angles: -

3.1±1.6 degrees), and no deterioration was detected at the 2-year follow-up.

With 20% regression (n=8) and 80% stabilization in this study, in which 65% of participants (n=26) reached bone maturity (Risser: 5) after two years of PSSE and no curve pattern was changed (Table 4). The risk of progression (LCR-value) significantly decreased in both groups (major thoracic: 44% vs 12%, p<0.001; major lumbar: 46% vs 11%, p<0.001) at the 2-year follow-up. Logistic regression analysis revealed that there was no correlation of age (OR: 0.7, 95% CI: 0.3 - 1.7, *p*=0.49), sex (OR: 0.9, 95% CI: 0.2 - 5.7, *p*=0.94), skeletal maturity (OR: 0.69, 95% CI: 0.05 - 10.6, *p*=0.79), bracing (OR: 0.4, 95% CI: 0.35-4.05, *p*=0.42) or curve patterns (OR: 4.2, 95% CI: 0.46 - 37.9, *p*=0.2) with curve regression or stabilization at the 2-year follow-up.

Table 4 Demographic and radiographic characteristics of participants at the 2-year follow-up.

**The 2-year follow-up**	**Major thoracic curves (n=22)**	**Major lumbar curves (n=18)**
**Age** (Mean±SD)	15.6 ± 1.8	14.7 ± 1.7
**Risser sign** 0-2,3-4,5 (n)	0,4,18	0, 10,8
**Cobb angle** (Mean±SD)	27.5 ± 7.0	22.9 ± 9.2
≥ 30 degrees: n (%): min – max	8 (36.4%): 31° - 40°	5 (27.8%): 30° - 43°
<30 degrees: n (%): min – max	14 (63.6%):15°- 28°	13 (72.2%): 10° -27°
**Curve pattern:** 3C / SL or STL (n)	12	11
N3N4 / 4C (n)	10	7

**Progression risk:** LCR-value (%)	12	11

LCR-value: Lostein and Carlson Risk of progression value.

Discussion

This was the first study to investigate the long-term outcomes of patients with different AIS curve patterns undergoing PSSE treatment. The findings of this study indicate no significant difference in Cobb angle changes (D-value of Cobb angles) between groups after regular PSSE for two years, regardless of the curve pattern. This implies that the curve pattern does not significantly impact the effects of PSSE in participants with AIS. A previous 6-month long study suggested that the curve pattern, a thoracic curvature with an imbalanced pelvis (n=15), is significantly correlated with the largest sum of curves after Schroth exercise, and that study further explained this might be due to a higher risk of progression in thoracic scoliosis.[12] However, with a short-term follow-up and limited sample size, that study could not draw any firm conclusions on the relationship of curve pattern and Schroth exercise in treating AIS. Our results extend this discussion with a longer follow-up period, and curve patterns were controlled before PSSE commenced. In this study, no curve progression was observed in either group, suggesting that a well-planned PSSE program can prevent scoliosis deterioration regardless of curve patterns. This result is consistent with previous findings that PSSE reduces curve progression in patients with mild curve.[30-32] Compared with those studies, we observed regression effects in larger curves and with a longer follow-up period. Moreover, participants in our study presented a higher progression risk (LCR-value) at the study initiation; additionally, participants with brace treatment and all received the comparable in-brace

correction of 37% improvement. The lack of deterioration may be attributable to the effects of both bracing and PSSE; however, because comparable in-brace correction explained the effects of bracing, the effects from the PSSE could be highlighted in our study. Therefore, our results revealed the superiority of PSSE programs in curve stabilization and regression of scoliosis for patients with mild to moderate scoliosis. However, a risk of overtreatment exists because patients with only mild curves may not experience progression. Therefore, the burden of treatment should be avoided as much as possible through periodically assessments.[12] Optimal time points for assessment require future study.

After two years of follow-up, no clinical deterioration was detected in either group, indicating a promising effect of PSSE on the prevention of scoliosis progression. Moreover, the regression effect accompanied by a notable decease of progression risk after 2 years. In particular, progression risk decreased to 12% in the thoracic curves and 11% in the lumbar curves, respectively. Moreover, in which 67% of them presented <30 degrees of Cobb with Risser 5 after two years of treatment. This is promising because of curve less than 30 degrees was unlikely to progress after skeletal maturity.[2] In particular, more participants in the major thoracic group showed curve regression in the first year (Table 2), but a decrease in compliance at the second year may have led to less of a regression effect at the 2-year follow-up. By contrast, a significant reduction in the Cobb angle was noted for participants with the major lumbar curves; moreover, a difference was observed as early as one and a half years after training, and reductions were maintained at the end of the study period. This was parallel with our original study hypothesis that major lumbar curves benefited more

from long-term exercise in terms of having a lower progression risk; however, this should be interpreted with caution because the progression value was comparable between groups at the study initiation. Hence, this observed promising result with lumbar curves might be explained by the better flexibility of the lumbar spine, confounding with a better exercise compliance and its associated effects of good core lumbar muscle stabilization. The superior flexibility of the lumbar spine compared to the thoracic spine has been proven in biomechanical studies[33-35]. The lumbar spine is mainly stabilized by core muscles that are flexible, stretchable and respond to external forces very well, whereas the stabilization mechanism in the thoracic spine is reliant on the rib cage, which contributes to rigidity and stability[34-36]. Additionally, according to the general principles of the Schroth method, correcting directions including side shift, axial elongation and derotation are created by muscle lengthening and eccentric contraction in the lumbar region, whereas spiral breathing is adopted in the thoracic region for correction[16]. Spiral breathing is difficult to learn and hard to control for most participants with AIS due to the requirement of breathing diagonally and asymmetrically not only in the frontal plane but also in the horizontal plane. Therefore, it is relatively easier for participants to correct the lumbar curvature via pelvic positioning, lengthening the curve concavity and stabilizing it in the proper position. In addition, stabilization effects from core muscles are crucial for maintaining correction in lumbar spines[16, 36]. Therefore, the lumbar curvature may respond better to the three-dimensional correction forces from a musculoskeletal functional perspective; however, real-time feedback of the spine during different corrective exercises requires future study.

In our study, significant differences between groups in exercise compliance were noted in the second year. Higher compliance in the major lumbar curve might contribute to the larger Cobb angle reduction. This extends the previous long-term studies of mild scoliosis that reported the only exercise intensity without reporting compliance.[15, 37] These previous studies have not addressed the relationship of compliance and treatment effects, which is of clinical valuable to define exercise protocols for subjects with PSSE treatment. In addition, our study revealed that an outpatient home-based PSSE program is a feasible protocol with reasonable compliance. The parents were asked to send monthly videos documenting the exercises performed to the Schroth therapist, which provided a reasonable variation control with respect to exercise quality and compliance. The frequency of such home-based programs was collected via a treatment adherence checklist, and exercise compliance was interpreted by how long participants spent on the PSSE program. This study calculated the supervised PSSE sessions and home exercise sessions in a sum number, in form of the total hours per week. Hence, we were unable to analyze specifically whether supervised PSSE is superior to home exercises in curve correction despite previous accounts of such a relationship[30-32]. Besides, the bracing compliance was self-reported and this might introduce recall effects, which can be improved if using a pressure sensor to monitor bracing hours. Nonetheless, only 10 participants and 5 of them were wearing nighttime brace only, hence, bracing would not cause a big distraction in our study.

This study had few inevitable limitations in addition to the small sample size. First, no untreated control group was included for comparison of intervention effects. However, because of

the proven benefits of PSSE on AIS [11-13], it was unethical to leave participants without treatment for as long as 2 years. Thus, the benefit observed may have been influenced by natural recovery of the condition. However, the progression risk at study initiation was evaluated with approximately 45% of progression rate, and with a moderate Cobb angles of 29 degrees with major thoracic and 27 degrees with major lumbar curves, hence, the natural recovery was unlikely in our study. Second, we only categorized participants into two groups, yet there were five scoliosis types according to the BSPTS concept, including 3C, 4C, N3N4, single lumbar/thoracolumbar and double thoracic.[22, 23] For participants having a double curve type such as N3N4 or 4C, the minor curve will cause limits to overstretching and twisting of the concavity from the major curve that weaken the effects of correction in the major curve. However, the proportion of curves pattern in this study was similar (Table 1: 40.9% vs 44.4%) between the two groups, which could not cause large variations in the data. Besides, there were only 6 participants with single right thoracic curve and no double upper thoracic curves were presented in this study, therefore, it would decrease the observed power if we extra analysis the single thoracic curves independently. However, a comparison of the effects of PSSE for all five curve types separately remains valuable for further study. Third, we were unable to blind the participants or physiotherapists due to the nature of PSSE studies, the exercise was prescribed and performed based on the understanding of the curve pattern by both therapists and patients, which may introduce methodological bias but also limited systematic errors such as learning effects. Performing exercise precisely is crucial to reach and maintain treatment effect, therefore, blinding of subjects and therapists was commonly sacrificed in most PSSE studies.[10, 11] However, the frequency and dosage of both supervised and home programs were strictly fixed, and we blinded the result assessor which controlled bias of statically analysis. Finally, we

hypothesized that the lumbar spine responds more to PSSE because of its lower progression rate and higher segmental spinal flexibility relative to the thoracic spine. However, because of radiation exposure concerns, no bending radiographic assessment conducted to quantify spinal flexibility before the PSSE program. Nonetheless, this study excluded participants with hypermobility that may cause systematic error during exercise. This controlled for potential heterogeneity when the spine started to respond to exercise. However, future study should address the relationship of spinal flexibility with PSSE, to define the correlation of curve location, segmental flexibility and PSSE in treating AIS.

Conclusion

PSSE treatment outcomes did not differ by thoracic or lumbar curve type. In general, PSSE with reasonable exercise compliance has positive effects on preventing scoliosis progression. However, further studies are necessary to address the correlation between spinal flexibility and the correction effects of PSSE at different curve magnitudes, curve types and levels involved. The cosmetic and quality of life outcomes of PSSE should also be studied. This study formed the base for future larger scale studies and randomized trials.

Supporting information

S1 File. Study protocol. (PDF)

S2 Fig. CONSORT flow diagram. (DOCX)

S3 File. Treatment adherence checklist. (PDF)

S4 File. Study data. (XLSX)

Acknowledgments

This study was financially supported by the Sanming Project of Medicine (SZSM201612055) "Team of Excellence in Spinal Deformities and Spinal Degeneration Diseases" and the Seeding Project (HKUSZH201902042) in Shenzhen, Guangdong Province, China.

Authors' contributions

YF and JPYC designed the study. JW and HR recruited participants. YF and EHKY performed PSSE teaching. ZX, GL and RZ collected data. MKTT performed statistics. YF drafted the manuscript. JPYC supervised the study and final edited the manuscript. MKTT, KMCC and JPYC obtained funding support. All authors read and approved the submitted version of the manuscript.

References:

1. Weinstein SL, Dolan LA, Cheng JC, Danielsson A, Morcuende JA. Adolescent idiopathic scoliosis. Lancet. 2008;371(9623):1527-37. Epub 2008/05/06. doi: 10.1016/s0140-6736(08)60658-3. PubMed PMID: 18456103.

2. Weinstein SL. The Natural History of Adolescent Idiopathic Scoliosis. J Pediatr Orthop. 2019;39(Issue 6, Supplement 1 Suppl 1):S44-s6. Epub 2019/06/07. doi: 10.1097/bpo.0000000000001350. PubMed PMID: 31169647.

3. Coe JD, Arlet V, Donaldson W, Berven S, Hanson DS, Mudiyam R, et al. Complications in spinal fusion for adolescent idiopathic scoliosis in the new millennium. A report of the Scoliosis Research Society Morbidity and Mortality Committee. Spine (Phila Pa 1976). 2006;31(3):345-9.

4. Korbel K, Kozinoga M, Stoliński Ł, Kotwicki T. Scoliosis Research Society (SRS) criteria and Society

of Scoliosis Orthopaedic and Rehabilitation Treatment (SOSORT) 2008 guidelines in non-operative treatment of idiopathic scoliosis. Polish orthopedics and traumatology. 2014;79:118-22.

5. Dolan LA, Weinstein SL, Abel MF, Bosch PP, Dobbs MB, Farber TO, et al. Bracing in Adolescent Idiopathic Scoliosis Trial (BrAIST): Development and Validation of a Prognostic Model in Untreated Adolescent Idiopathic Scoliosis Using the Simplified Skeletal Maturity System. Spine Deform. 2019;7(6):890-8. e4.

6. Almansour H, Pepke W, Bruckner T, Diebo BG, Akbar M. Three-Dimensional Analysis of Initial Brace Correction in the Setting of Adolescent Idiopathic Scoliosis. Journal of clinical medicine. 2019;8(11):1804.

7. Thompson RM, Hubbard EW, Jo CH, Virostek D, Karol LA. Brace Success Is Related to Curve Type in Patients with Adolescent Idiopathic Scoliosis. J Bone Joint Surg Am. 2017;99(11):923-8. Epub 2017/06/08. doi: 10.2106/jbjs.16.01050. PubMed PMID: 28590377.

8. Negrini S, Carabalona R. Social acceptability of treatments for adolescent idiopathic scoliosis: a cross-sectional study. Scoliosis. 2006;1:14. Epub 2006/08/26. doi: 10.1186/1748-7161-1-14. PubMed PMID: 16930488; PubMed Central PMCID: PMCPMC1560163.

9. Burger M, Coetzee W, du Plessis LZ, Geldenhuys L, Joubert F, Myburgh E, et al. The effectiveness of Schroth exercises in adolescents with idiopathic scoliosis: A systematic review and meta-analysis. S Afr J Physiother. 2019;75(1):904. Epub 2019/06/18. doi: 10.4102/sajp.v75i1.904. PubMed PMID: 31206094; PubMed Central PMCID: PMCPMC6556933.

10. Day JM, Fletcher J, Coghlan M, Ravine T. Review of scoliosis-specific exercise methods used to correct adolescent idiopathic scoliosis. Arch Physiother. 2019;9:8. Epub 2019/08/30. doi: 10.1186/s40945-019-0060-9. PubMed PMID: 31463082; PubMed Central PMCID: PMCPMC6708126.

11. Thompson JY, Williamson EM, Williams MA, Heine PJ, Lamb SE. Effectiveness of scoliosis-specific exercises for adolescent idiopathic scoliosis compared with other non-surgical interventions: a systematic review and meta-analysis. Physiotherapy. 2019;105(2):214-34. Epub 2019/03/03. doi: 10.1016/j.physio.2018.10.004. PubMed PMID: 30824243.

12. Schreiber S, Parent EC, Khodayari Moez E, Hedden DM, Hill DL, Moreau M, et al. Schroth Physiotherapeutic Scoliosis-Specific Exercises Added to the Standard of Care Lead to Better Cobb Angle Outcomes in Adolescents with Idiopathic Scoliosis - an Assessor and Statistician Blinded Randomized Controlled Trial. PLoS One. 2016;11(12):e0168746. Epub 2016/12/30. doi: 10.1371/journal.pone.0168746. PubMed PMID: 28033399; PubMed Central PMCID: PMCPMC5198985.

13. Yagci G, Ayhan C, Yakut Y. Effectiveness of basic body awareness therapy in adolescents with idiopathic scoliosis: A randomized controlled study1. J Back Musculoskelet Rehabil. 2018;31(4):693-701. Epub 2018/04/10. doi: 10.3233/bmr-170868. PubMed PMID: 29630516.

14. Yagci G, Yakut Y. Core stabilization exercises versus scoliosis-specific exercises in moderate

idiopathic scoliosis treatment. Prosthet Orthot Int. 2019;43(3):301-8. Epub 2019/01/11. doi: 10.1177/0309364618820144. PubMed PMID: 30628526.

15. Liu D, Huang S, Yu X, Yang J, Xuan X, Yang J, et al. Effects of Specific Exercise Therapy on Adolescent Patients with Idiopathic Scoliosis: A Prospective Controlled Cohort Study. Spine (Phila Pa 1976). 2020. Epub 2020/02/26. doi: 10.1097/brs.0000000000003451. PubMed PMID: 32097267.

16. Lehnert-Schroth C. [Schroth's three dimensional treatment of scoliosis]. ZFA (Stuttgart). 1979;55(34):1969-76. Epub 1979/12/10. PubMed PMID: 547573.

17. Lehnert-Schroth C. [The "four-curved" scoliosis. The effect on the additional lumbosacral spinal curve by the three dimensional Schroth's scoliosis treatment]. ZFA (Stuttgart). 1981;57(32):2227-31. Epub 1981/11/20. PubMed PMID: 7314859.

18. Little DG, Sussman MD. The Risser sign: a critical analysis. Journal of Pediatric Orthopaedics. 1994;14(5):569-75.

19. Cobb JR. Scoliosis—Quo Vadis? JBJS. 1958;40(3):507-10.

20. Smits-Engelsman B, Klerks M, Kirby A. Beighton score: a valid measure for generalized hypermobility in children. J Pediatr. 2011;158(1):119-23, 23.e1-4. Epub 2010/09/21. doi: 10.1016/j.jpeds.2010.07.021. PubMed PMID: 20850761.

21. Czaprowski D, Kotwicki T, Pawłowska P, Stoliński L. Joint hypermobility in children with idiopathic scoliosis: SOSORT award 2011 winner. Scoliosis. 2011;6(1):22.

22. Berdishevsky H, Lebel VA, Bettany-Saltikov J, Rigo M, Lebel A, Hennes A, et al. Physiotherapy scoliosis-specific exercises–a comprehensive review of seven major schools. Scoliosis Spinal Disord. 2016;11(1):20.

23. Rigo MD, Villagrasa M, Gallo D. A specific scoliosis classification correlating with brace treatment: description and reliability. Scoliosis. 2010;5(1):1.

24. Negrini S, Atanasio S, Fusco C, Zaina F. Effectiveness of complete conservative treatment for adolescent idiopathic scoliosis (bracing and exercises) based on SOSORT management criteria: results according to the SRS criteria for bracing studies-SOSORT Award 2009 Winner. Scoliosis. 2009;4(1):19.

25. Malfair D, Flemming AK, Dvorak MF, Munk PL, Vertinsky AT, Heran MK, et al. Radiographic evaluation of scoliosis. American Journal of Roentgenology. 2010;194(3_supplement):S8-S22.

26. Fitzmaurice GM, Laird NM, Ware JH. Applied longitudinal analysis: John Wiley & Sons; 2012.

27. Lonstein J, Carlson J. The prediction of curve progression in untreated idiopathic scoliosis. J Bone Jt Surg. 1984;3(2):1061-71.

28. Barnes P, Brody J, Jaramillo D, Akbar J, Emans J. Atypical idiopathic scoliosis: MR imaging evaluation. Radiology. 1993;186(1):247-53.

29. Wang W, Zhu Z, Zhu F, Sun C, Wang Z, Sun X, et al. Different curve pattern and other radiographical characteristics in male and female patients with adolescent idiopathic scoliosis. Spine (Phila Pa 1976). 2012;37(18):1586-92.

30. Weiss HR, Lohschmidt K, el-Obeidi N, Verres C. Preliminary results and worst-case analysis of in patient scoliosis rehabilitation. Pediatr Rehabil. 1997;1(1):35-40. Epub 1997/01/01. doi: 10.3109/17518429709060940. PubMed PMID: 9689236.

31. Weiss HR, Weiss G. Curvature progression in patients treated with scoliosis in-patient rehabilitation--a sex and age matched controlled study. Stud Health Technol Inform. 2002;91:352-6. Epub 2004/10/02. PubMed PMID: 15457754.

32. Weiss HR, Weiss G, Petermann F. Incidence of curvature progression in idiopathic scoliosis patients treated with scoliosis in-patient rehabilitation (SIR): an age- and sex-matched controlled study. Pediatr Rehabil. 2003;6(1):23-30. Epub 2003/05/15. doi: 10.1080/1363849031000095288. PubMed PMID: 12745892.

33. Hajihosseinali M, Arjmand N, Shirazi-Adl A, Farahmand F, Ghiasi MS. A novel stability and kinematics-driven trunk biomechanical model to estimate muscle and spinal forces. Medical engineering & physics. 2014;36(10):1296-304. Epub 2014/07/31. doi: 10.1016/j.medengphy.2014.07.009. PubMed PMID: 25074649.

34. Oxland TR. Fundamental biomechanics of the spine--What we have learned in the past 25 years and future directions. Journal of biomechanics. 2016;49(6):817-32. Epub 2015/12/27. doi: 10.1016/j.jbiomech.2015.10.035. PubMed PMID: 26706717.

35. Panjabi MM, White AA, 3rd. Basic biomechanics of the spine. Neurosurgery. 1980;7(1):76-93. Epub 1980/07/01. doi: 10.1227/00006123-198007000-00014. PubMed PMID: 7413053.

36. Eskandari AH, Arjmand N, Shirazi-Adl A, Farahmand F. Subject-specific 2D/3D image registration and kinematics-driven musculoskeletal model of the spine. Journal of biomechanics. 2017;57:18-26. Epub 2017/04/04. doi: 10.1016/j.jbiomech.2017.03.011. PubMed PMID: 28365064.

37. Monticone M, Ambrosini E, Cazzaniga D, Rocca B, Ferrante S. Active self-correction and task-oriented exercises reduce spinal deformity and improve quality of life in subjects with mild adolescent idiopathic scoliosis. Results of a randomised controlled trial. Eur Spine J. 2014;23(6):1204-14. Epub 2014/04/01. doi: 10.1007/s00586-014-3241-y. PubMed PMID: 24682356.

REPLY TO REVIEWERS

Re: Ms. No. PONE-D-19-35759 - "Dose Curve Pattern Impact on the Effects of Physiotherapeutic Scoliosis Specific Exercises on Cobb angle of Adolescent Idiopathic Scoliosis: A prospective clinical controlled trial with two years follow-up"

Dear Editor,

The authors would like to thank you and the Reviewers for all of your time and effort devoted to the review of our aforementioned manuscript. The Reviewers' comments were indeed extremely insightful and greatly appreciated. As such, the authors would like to take this opportunity to address each and every concern the Reviewers noted in their review of our submission. In addition, where appropriate, we have also revised our manuscript accordingly. Major changes are made in light gray.

We believe that the Reviewers' comments have helped improve the quality of our manuscript. We hope that you and the Reviewers will find our revised work suitable for publication in *PLOS One.*

Editor

Comment #1: Journal Requirements:

When submitting your revision, we need you to address these additional requirements.

1. Please ensure that your manuscript meets PLOS ONE's style requirements, including those for file naming. The PLOS ONE style templates can be found at

https://journals.plos.org/plosone/s/file?id=wjVg/PLOSOne_formatting_sample_main_body.pdf and

https://journals.plos.org/plosone/s/file?id=ba62/PLOSOne_formatting_sample_title_authors_affiliations.pdf

Response: We have revised the format accordingly.

Comment #2: 2. Thank you for submitting your clinical trial to PLOS ONE and for providing the name of the registry and the registration number. The information in the registry entry suggests that your trial was registered after patient recruitment began. PLOS ONE strongly encourages authors to register all trials before recruiting the first participant in a study.

As per the journal's editorial policy, please include in the Methods section of your paper: Please give confirmation that all related trials are registered by stating: "The authors confirm that all ongoing and related trials for this drug/intervention are registered".

Response: We have revised this. Please see **lines 108-110, page 7** (Materials and methods: study design)

Comment #3: 3. You indicated that you had ethical approval for your study.

In your Methods section, please ensure you have also stated whether you obtained consent from parents or guardians of the minors included in the study or whether the research ethics committee or IRB specifically waived the need for their consent.

Response: Thank you for the comment. Please see **lines 105-107,page 7** (Materials and methods: study design)

Comment #4: 4. We note that you have stated that you will provide repository information for your data at acceptance. Should your manuscript be accepted for publication, we will hold it until you provide the relevant accession numbers or DOIs necessary to access your data. If you wish to make changes to your Data Availability statement, please describe these changes in your cover letter and we will update your Data Availability statement to reflect the information you provide.

Response: No change is needed.

Comment #5: 5. Please amend either the title on the online submission form (via Edit Submission) or the title in the manuscript so that they are identical.

Response: Thank you this has been changed. The title is now: **Dose curve pattern impact on the effects of physiotherapeutic scoliosis specific exercises on Cobb angles of participants with adolescent idiopathic scoliosis: a prospective clinical trial with two years follow-up**

Comment #6: 6. Please amend your list of authors on the manuscript to ensure that each author is linked to an affiliation. Authors' affiliations should reflect the institution where the work was done (if authors moved subsequently, you can also list the new affiliation stating "current affiliation:…." as necessary).

Response: This has been done.

Comment #7: 7. Please include your table as part of your main manuscript and remove the individual file.

Please note that supplementary tables should be uploaded as separate "supporting information" files.

Response: This has been added.

Comment #8: 8. We note that Figures 3 and 4 include images of patients.

As per the PLOS ONE policy (http://journals.plos.org/plosone/s/submission-guidelines#loc-human-subjects-research) on papers that include identifying, or potentially identifying, information, the individual(s) or parent(s)/guardian(s) must be informed of the terms of the PLOS open-access (CC-BY) license and provide specific permission for publication of these details under the terms of this license. Please download the Consent Form for Publication in a PLOS Journal (http://journals.plos.org/plosone/s/file?id=8ce6/plos-consent-form-english.pdf). The signed consent form should not be submitted with the manuscript, but should be securely filed in the individual's case notes.

Please amend the methods section and ethics statement of the manuscript to explicitly state that the patient/participant has provided consent for publication: "The individual in this manuscript has given written informed consent (as outlined in PLOS consent form) to publish these case details".

If you are unable to obtain consent from the subject of the photograph, you will need to remove the figure and any other textual identifying information or case descriptions for this individual.

Response: Thank you for the comment. We have included the necessary documents. The statement was made on **lines 107 to 108, page 7** (Materials and methods: study design).

Comment #9: 9. Please include captions for your Supporting Information files at the end of your manuscript, and update any in-text citations to match accordingly. Please see our Supporting Information guidelines for more information: http://journals.plos.org/plosone/s/supporting-information

Response: These have been included.

Reviewer #1

Comment #1: The article entitled "Dose Curve Pattern Impact on the Effects of Physiotherapeutic Scoliosis Specific Exercises on Cobb angle of Adolescent Idiopathic Scoliosis Subjects: A prospective clinical controlled trial with two years follow-up" has a good topic and matter in the field. However, the methodology of the study needs to be revised. The authors investigate the effects of scoliosis-specific exercise method on different curve patterns (major thoracic and major lumbar) and in long term. The idea is valuable. But the cobb angle range is wide, therefore the inevitable effect of brace wearing for some participants on the results looks unnoticed. Randomization is needed for age, gender, cobb angle, curve pattern, brace wearing, in-brace correction, risk of progression... etc. Before consideration for publication, there may be following suggestions to consider again:

Abstract - In method, information regarding Cobb angle of the subjects are needed. Was curve magnitude similar between thoracic and lumbar curve groups?

Response: We thank the reviewer for the comments and we will try to amend our manuscript accordingly.

The curve magnitude was similar between groups: 28.5 ± 8.4 vs 26.8 ± 10.1, p=0.6.

Please refer to the 1) page 3, **abstract:** results: **lines 29 - 31;**

2) **results,** participants, **lines 222 -227;**

3) Table 1: line 237

Comment #2: Did patient undergo only exercise intervention or did they use bracing as well?

Response: A total of **10 participants** were wearing brace before commencing the PSSE program and having comparable, 37% of correction effects from bracing.

Please refer to the 1) **results: participants, lines 231 - 235. 2) Table 1: line 237**

Comment #3: The first sentence of the background needs to be revised related with exercise. The statement is associated with brace and surgery.

Response: We thank the reviewer for this suggestion. This has been revised. Please refer to **page 2, abstract: background, lines 10-11**

Comment #4: What were patients' ages? Was age similar between groups?

Response: Yes, the age was under evenly distributed and was similar between groups.

Please refer to the **page 3, abstract: results, lines 30 to 31, Table 1 (line 237) and lines 227 to 228 of page 15.**

Comment #5: Introduction

- The importance of curve progression risk in conservative treatment was extensively reported. But did you randomize your patients in terms of bone maturity among groups?

Response: Thanks for comment. This study was planned as a case control study. Participants were recruited from one scoliosis clinic and grouped into two groups according to their curve pattern. The progression related data was analyzed at the baseline and correlation analysis was performed

to detect any influence of these factors, including bone maturity, on the reduction of Cobb angles. Please refer to the methodology, study design, **lines 119-123, line 125** (**S2** Fig. 1: CONSORT flow diagram)**, line 237 (Table 1) and lines 210 to 213 (statistical analysis).**

Comment #6: Did you measure curve flexibility? If so, how did you measure? Because you reported that this study aimed to investigate the long-term therapeutic effect of PSSE on thoracic and lumbar major curves and to determine whether a more flexible lumbar curve would respond better to exercise.

Response: This is an important point by the reviewer. Unfortunately we were unable to quantify spinal flexibility prior to treatment in this study due to patients' radiation concern, therefore, **the hypothesis was re-centered on curve patterns.** Please see the text at **lines 94 to 99 (Introduction), lines 369 to 374 (discussion); lines 433 to 441 (Study limitation).**

Comment #7: Methods

- One of your inclusion criteria was having cobb angles: from 10 degrees to 50 degrees. This means that some of your patients had bracing indication. How many of your patients did wear brace in this period? Then how did you differentiate exercise specific effect?

Response: Ten participants (5 were wearing full time brace whereas 5 were wearing only night time brace), were wearing brace and achieved comparable in-brace correction 37%, which explained the effects from bracing and allow this study emphasized on the effect from the PSSE.

Please check the **Table 1 (line 237)**, **lines 231 to 235 (results) and lines 349 to 353 (discussion).**

Comment #8: Please add detailed explanation about The Rigo Scoliosis Classification system.

Response: Rigo clasification was adopted to rigo cheneau brace fabrication and also BSPT Schroth methods, in which 5 classifications with 8 sub-types were clearly introduced. two important references was cited accordingly, **please see references 22 and 23.**

Comment #9: You included major thoracic and major lumbar curves. I see from the method that some of them, who had major thoracic curve, had double curves or secondary lumbar curves. This would affect results. We know that single lumbar curves and double curves are more common than other curve pattern in idiopathic scoliosis. Single thoracic curves are rare. Therefore, curve distribution among groups would differ. In table 1, curve pattern was reported as double and single, only. But more information is needed regarding curve pattern distribution.

Response: We thank the reviewer for these comments. This information was revised in:

1. Lines 222 to 227.

2. S2: Fig.1 CONSORT flow chart

3. Table 1.

4. Please also see lines 416-426 of paragraph-study limitation.

Comment #10: For single curve, how many patient had thoracic, how many patient had lumbar? For double curve, how many had primary thoracic? how many had primary lumbar? What was curve pattern distribution of the participants?

Response: Thanks for your suggestion. The additional information has been added to:.

1. **Lines 222 to 227.**
2. **S2: Fig.1 CONSORT flow chart**
3. **Table 1.**

Please also see lines 416-426 of paragraph-study limitation.

Comment #11: How was curve range for thoracic and lumbar curves? How many vertebrae did curves include? This would affect results. What do you think?

Response: Fantastic comment, we do agree that the range of the curvature would definitely impact on the treatment effects, in terms of that may affect the spinal segmental flexibility. However, curve range should also be correlated with curve pattern, such as a short range should be observed in single lumbar curves whereas a long range should be observed in a thoracolumbar, etc. This idea is good but unfortunately out of scope of this study, the sample size of each curve pattern was

small in our study, therefore, extra analysis of range of curvature may cause distraction from our primary outcome. Moreover, it will be too informative and overwhelming to readers of those who are not familiar with scoliosis radiographic classification. However, it will be interesting to study the effects of PSSE on the different radiographic parameters. Please see this amendings to **lines 446-447 of paragraph conclusion.**

Comment #12: Did you measure axial trunk rotation of the participants? Curve magnitude would affect from trunk rotation parameter as much as Cobb angle?

Response: This is an important parameter that will be studied in a future study. We wanted to mainly focus on the Cobb angle changes in this manuscript. Further investigation of the cosmesis and quality of life will also be performed. Please see **lines 447-448 of paragraph Conclusion.**

Comment #13: Did you use and patient-reported outcome measures? or Did you assess any clinical parameters such as trunk symmetry and cosmetic deformity?

Response: As discussed in comment #12, these are important parameters to study. We have not included them in this manuscript because we wanted to focus only on curve magnitude changes.

Comment #14: Did any of your patient use spinal brace in this period? When considering wide range of Cobb angle, this looks inevitable. How was the distribution of spinal brace usage among groups? How was in-brace correction for patients who underwent spinal brace intervention? Because if the correction amount differ among groups, this would increase or decrease the exercise effect.

Response: Please check the **Table 1** and **lines 231 to 235; lines 323 to 326** of paragraph results, **discussion: lines 349 to 353; lines 405 to 408.**

Comment #15: Male distribution is higher in group B. Do you think this would affect results? Also bone maturity distribution among groups seems to be different with 11 - 11 in group A, and 12-6 in group B. Group B looks to have participant who had low risk of progression.

Response: These are important confounders. Using the logistic correlation analysis, we did not find any significant relationship with gender and maturity.

Please refer to **lines 323 to 326, Table 1.**

Comment #16: In table one, minimum and maximum values are needed.

Response: Range was reported **in the abstract;** Mean ± SD and 95% CI have been added **in the Table 1.**

Comment #17: Did authors use any randomization method for age, gender, Cobb angle, curve progression risk (bone maturation)? If not, how would it be possible to make exact comparison of two different curve pattern groups (thoracic and lumbar)

Response: It was convenient sampling with a curve pattern controlled study, the assumption of normality was calculated before commencing the study and analysis was conducted for baseline data between groups. Logistic regression analysis was adopted to detect any correlation of variables that differed on baseline between groups and treatment effect, which was reported on **lines 210 to 213; lines 323 to 326,** curve distribution can be found in **S2 Fig 1** and **table 1.**

Comment #18: I am sending my suggestions until here. Discussion is also needed to be revised according to methodology.

Response: Thanks for your valuable suggestions and hope you will find the updated discussion interesting.

<u>Reviewer #2</u>

Comment #1: The curve title should be corrected to: Does Curve Pattern Impact the Effects of Physiotherapeutic Scoliosis Specific Exercises on Cobb angles Participants with Adolescent Idiopathic Scoliosis: A prospective clinical controlled trial with two years follow-up.

Response: We thank the reviewer for this suggestion and this has been revised.

Comment #2: It is best to refer to participants or persons with a diagnosis rather than subjects. Throughout the text please replace patients by participants or persons.

Response: These have been corrected.

Comment #3: ABSTRACT Please specify details of the intensive frequency and reduce frequency. (number of visits per week or month and their duration of exercising at minimum). Specify if a home program was performed.

Response: This has been done. Please refer to the **page 2, abstract, methods, lines 19 to 23.**

Comment #4: If the study is controlled. I assumed there are two groups. Specify what the control group did during the trial in the abstract.

Response: This was planned as a clinical, curve pattern controlled study. Please refer to the **page 2, abstract, methods, lines 17 to 19. S2 Fig 1 Consort flow diagram.**

Comment #5: Then the analysis should likely be a mixed model ANOVA with a between subject group factor and a within subject (repeated measures) time factor. There should not be multiple paired t-tests if you choose a relevant pairwise comparison over time within curve patterns. Using separate t-tests would increase your risk of type one error.

Response: We thank the reviewer for this astute comment. The analyses have been corrected according to your valuable suggestions, please see **pages 2-3: abstract, method (abstract): lines 24 to 26; statistics lines (main text): 196 to 213; results (abstracts): line 34-39; results (main text): lines 259 to 291; Tables 2,3.**

Comment #6: There are numerous English issues. I will not point all of them as English is a second language for me too. Please have the paper reviewed by an English editor. EG always use major thoracic curve and major lumbar curve.

Response: Thank you for the comments. This has been amended accordingly.

Comment #7: Specify the recruitment setting and method for the participants.

Response: This has been done. Please see **page 7, methods, study design, lines 103 to 105; lines 110 to 123** and **S2 (Fig. 1: CONSORT flow diagram).**

Comment #8: Beyond stating no difference in the curve magnitude between groups please report the curve means and SD and how many patients if any were braced. If none wore a brace, specify.

Response: Ten participants (5 were wearing full time brace whereas 5 were wearing only night time brace), were wearing brace and achieved comparable in-brace correction 37%, which explained the effects from bracing and allow this study emphasized on the effect from the PSSE.

Please check **lines 132 to 135 of page 9**, the **table 1** and results, **lines 231 to 235**; discussion, **lines 348 to 351.**

.

Comment #9: Spell out d on first use for D-value.

Response: The D-value stands for the difference value of Cobb angle over three testing times with the initial Cobb angle, to detect the regression, stabilization and deterioration between groups. **This has been reiterated in lines 203-206 and table 2.**

Comment #10: Since we don't know how you calculated d, we don't know in the abstract if the change over 1 year is a improvement or deterioration in Cobb angles in each group. The sentence with the change in COBB for the specific intervals should specify which group had which results. There should be multiple F statistics and p-values with your design: one for the interaction between groups and time, one for the main effect of group and one for the main effect of time.)

Response: We are sorry for this confusion and the analysis has been clarified. Please see the **revised abstract, methods (lines 203-206), results (lines 259 to 365) and Table 2.**

Comment #11: The value for the regressions in group B do not seem to map to any of the values in the sentence prior. How were these group be reductions calculated.

Response: Regression was defined as a decrease of Cobb angle $\geq$ 6 degrees, the percentage value in each group was reported in the results session, in addition, raw Cobb angle was also analyzed using the repeated measures, please refer to the **outcome measurement (lines 184 to 186) and results (lines 259-265; lines 272 - 291)**. All results, including mean, SD, 95% CI of the D-value and of the raw Cobb angles, were reiterated and summarized in the **Table 2 and Table 3, respectively.**

Comment #12: Report data on the compliance and drop outs with the exercise program. Did all 40 participants attend all follow-up. If yes specify this is a per protocol analysis and report how many had started and were not included in the analyses.

Response: There were no dropouts through the whole studies. Per-protocol analysis was specified accordingly. Please see **lines 253 - 257**; **S2: Fig.1 CONSORT flow diagram**

Comment #13: The conclusion is inaccurate: This was the first study to investigate whether PSSE can lead to curve regression based on location of the major curve. The following study examined the effect of curve type in a multivariate analysis of the short-term effects of Schroth on Cobb angles. Schroth Physiotherapeutic Scoliosis-Specific Exercises Added to the Standard of Care Lead to Better Cobb Angle Outcomes in Adolescents with Idiopathic Scoliosis - an Assessor and Statistician Blinded Randomized Controlled Trial. Schreiber S, Parent EC, Khodayari Moez E, Hedden DM, Hill DL, Moreau M, Lou E, Watkins EM, Southon SC. PLoS One. 2016 Dec 29;11(12):e0168746. doi: 10.1371/journal.pone.0168746. eCollection 2016.

Response: Thanks for you pointing this out, the conclusion and discussion has been revised to "**an extension of previous studies".**

Comment #14: Introduction

In the intro where you state the following about the reviews it may be important to review each paper in those reviews for discussions of the effects of curve type. The reviews did not address this question but some of the original reviewed trials did. however, in these studies, the relationship of curve location with correction effects was not clearly discussed, and there was only a short-term followup. There may be limitations to those as short follow-ups which would still justify your work that you could highlight.

Response: Thanks for this brilliant suggestion, the introduction was revised and followed your suggestions, please refer to the **lines 77 to 85 (Introduction).**

Comment #15: The following intro statement is also overreaching. Schreiber et al above did include baseline Cobb angle in their study of the effect of Schroth. There maybe limitations to this understanding but it is not completely unknown. You stated "the influence of curve magnitude on exercise outcomes is unknown."

Response: We apologize for the oversight. This has been corrected.

Comment #16: The objective as stated focuses on curve magnitude and flexibility rather than

relation between outcomes and curve types. Your focus seems to be on curve type in the abstract. Please re-center the objective on this topic.

Response: We thank the reviewer for this comment. **It has been re-centered.** Please see **lines 94 - 98.**

Comment #17: Specify your recruitment methods and setting. Were all consecutive eligible participants invited? Only referrals from some DRs…

Response: patients were recruited from a scoliosis clinic, therefore, all consecutive eligible participants were invited and decision was made according to the inclusion/exclusion criteria. Please **see lines 111 to 123 and <u>S2 Fig 1.</u>**

Comment #18: Please add a justification to the exclusion of hypermobility. This has not been recommended by SOSORT and has not been done in other study. Maybe, if possible, report how many patients were excluded on this basis.

Response: There were 18 subjects were detected with hypermobility, in form of Beighton score >4, who were excluded from this study. In addition, a total of sixty-two subjects were excluded after eligibility assessment, the exclusion reasons were specified on the **lines 115-119; lines 218 to 222 (Results: participants) and S2 (Fig.1 CONSORT flow diagram).** One previous study reported hypermobility as a dominant presence in the single curve type and should be taken into account during physiotherapy. Participants with hypermobility were excluded to control/limit the systematic error in this study, the related paper was cited in accordingly. **Please see reference 21.**

Comment #19: Please specify if patients could have been prescribed a brace at baseline with the exercises or not in the selection criteria.

Response: Yes, they could have. Please see line 115 (exclusion criteria: previous treatment) and **lines 132-135 (paragraph: study interventions).**

Comment #20: From a Asklepios (German) Schroth trained perspective grouping N3N4 with 3C is not consistent with only grouping thoracic curves. Please acknowledge as a limitation that the N3N4 group likely had both thoracic and lumbar curves and may have needed different treatment than the 3C curves. For example the Chest twister exercise is not indicated for N3N4. Similarly. The type 4C curves do have a lumbar dominance but also have a thoracic curves. What did you do with the pure lumbar or thoracolumbar curves? Specify if you had any in your sample.

Response: N3N4, BSPT approach, is a bit different from the German approach, it mainly focuses on correction for thoracic curve, like gentle chest twisting was applicable but should perform without changing the balance and without over correction. By contrast, the double curve in the German approach, takes the lumbar curvature in priority, therefore using pelvic strategy more than chest twisting, etc. The principles are mostly same but the practical metric is a bit different. We did not explain too much or try to compare the two methods in this manuscript, we were trying to prove the effects of Schroth with Cobb correction instead of centering on how did we position patients. Explaining this practical metric is valuable but may be overwhelming for readers who are not familiar with Schroth, moreover, the dynamic feedback of the spine with exercise is unclear yet. Therefore, after all consideration, we did not include this explanation in our manuscript.

Comment #21: Please specify the certification of the Schroth therapists BSPTS -rigo, or Asklepios or ISST or Weiss…

Response: BSPTS approach, we firstly registered as BSPTS in 2016, then registered Asklepios in 2018, therefore, mainly BSPTS concept in this study. Please see **lines 129.**

Comment #22: When were participants asked to complete the simple home exercise compliance questionnaire (Weekly, monthly, once at then of the study…)

Response: Monthly. Please see **lines 137-143.**

Comment #23: In listing exercises could the figure order be arranged to match the order of presentation in the text.

Response: Sorry for the confusion. This has been amended.

Comment #24: There is a variation of the muscle cylinder for thoracic curves. Specify why it was not used.

Response: Please see lines **153-161**. This was not considered due to the pain at the knee joint.

Comment #25: Describe how the those was chosen. Did the patient do the same exercise throughout the program? Acknowledge as a limitation why you did not use dynamic exercises such as walking or …

Response: We thank the reviewer for this astute comment. Please see **lines 158-161**.

Comment #26: Throughout the paper: I recommend avoiding the labels A and B for the groups and clearly stating thoracic vs Lumbar groups.

Response: We thank the reviewer for this comment. It has been changed.

Comment #27: Figure 5 and 6 Please add a justification for why these participants were not braced in addition to doing the exercises.

Response: Two p**atients were mostly considering about the cosmetic issue;** especially one girl was at late Risser stage (Fig 5) and bracing was not compulsory for her, then this girl tried exercise for a half year and resulted with a significant correction, then she had never considered bracing

again.

Comment #28: Table 1. Add units for age.

Response: Added. **Table 1**

Comment #29: Replace gender by Sex. I doubt you documented gender.

Response: This has been done.

Comment #30: Why not report count and percentage as you did for sex instead for Sanders stage.

Response: The Sanders stage has been replaced by Risser, because of the progression risk value in terms of the Lonstein and Carlson score, that needs the Risser for calculation. **Please see the S4 File: raw data set and Table 1.**

Comment #31: Can you specify brace types. This is a notable imbalance and surprisingly more braced in thoracic group?

Response: Thanks for the comment. Please see **lines 231 - 233** on page 9. All (n=10) were wearing Cheneau brace **(Table 1).**

Comment #32: For future meta-analysis purpose it may be valuable to still measure thoracic and lumbar curves in both groups.

Response: We agree with the suggestion. Please see **Table 1**, **S2 (Fig. 1: CONSORT flow Chart) and S4 File: study data.**

Comment #33: Rather than or in addition to, please report the number of N3N4 vs 4C per group or add a note to each column showing the thoracic double curves were all N3N4 and the Lumbar were all 4C.

Response: This has been added. Please see **S2 (Fig. 1: CONSORT flow Chart) and the Table 1.**

Comment #34: Specify in the methods if the classification was clinical only or informed by radiographs also.

Response: Radiographic, BSPTS is using this classification system for bracing too, therefore the X-ray is in priority for classification and the clinical presentation was only for clinical reference.

Please see **lines 119 to 120 and S2 (Fig.1 CONSORT flow diagram.)**

Comment #35: Can you report if all thoracic curves were right side and all lumbar were left sides. If not please report the distribution.

Response: This has been clarified in **lines 222-227, (S2** Fig.1 Consort flow diagram) and **Table 1.**

Comment #36: Figure 2 could present statistical significance of the comparison of the values as well with symbols.

Specify what the error bar represent (standard deviations or standard errors).

Response: The new figure 1 has been amended. The previous **Figure 2 was replaced by the table 3** shows raw data of Cobb angles over four testing time points, in which <u>mean +/- SD, 95% CI and</u>

range were all presented, which may be of value for future meta-analysis.

Comment #37: Figure 3. It is unfortunate that the patient has a wide racer back bra which hides much of the postural defect in thoracic spine. The semi-hanging picture has allowed too much hypokyphosis. Would you have a semi-hanging example where the sagittal kyphosis is better maintained. The aspect ratio of the photo f seems off (too wide not tall enough). Can you add the left right marker to the radiograph. It show right thoracic to the right of the image.

Response: **A new picture has been uploaded**. Unfortunately, we cannot find a better photo as patients cannot come to our center due to COVID. Therefore, I used her another pic for demonstration, please check see whether it is acceptable.

Comment #38: Figure 4. Can you add the left right marker to the radiograph. This one shows the right thoracic curve on the left of the image. Once again in C the patient shows hypokyphosis and this time has lost physiological lordosis. Can you find a demonstration of semi-hanging in this group with better sagittal profile. For G, can you have an example with proper head alignment.

Response: This has been amended.

Comment #39: Figure 5 and 6 were presented with figure 6 first. No need to use initials in the figures. Label as example of a thoracic vs lumbar participants. Could you draw the Cobb measurements on the images? Could the scaling of the two spine images in fig 6 be made more similar?

Response: This has been added.

Comment #40: The dataset uploaded should also include a data dictionary or a legend. What values are coded 1 and 0 … Define cut point 30 deg. Is the data incomplete. What appears in columns V to AO??

Response: Done, the file has been amended. The other columns were including other data such as ATR, SRS-22 etc. We have deleted them as the data on radiographic measurements were used in this study. Please see **S4 File: study data.**

Comment #41: Specify in the selection criteria what was the criteria for brace prescription and brace termination and how you monitored brace compliance. Detail the kinds of brace that were prepared and possibly the targeted or achieved in brace correction if available.

Response: The SRS bracing criteria was used by doctors to prescribe brace treatment, see **lines 132 to 135,** and the reference was cited accordingly. Brace termination was defined with skeletal maturity, and bracing compliance was monitored using a monthly self-reported adherence checklist, **see lines 139 to 143**. In-brace correction and brace type were clarified on **lines 233 to 235** and **Table 1.**

Comment #42: Can you detail the positioning instructions for the participants during the radiographs.
In clinical trials the study is not powered to compare group characteristics at baseline. It does not mean much to do statistical comparisons of those. Key is whether the estimate appear to present clinical differences before deciding whether to control for those.

Response: amended accordingly. **Please see line 144 to 153. Picture legend of Fig. 2 and Fig. 3.**

The comparison of the baseline data was to show the assumption of normality, if the baseline was evenly distributed and which can power our study quality. However, I fully agree with you that a clinical trial should look more on the factors that may affect results, but we have to do so according to suggestions from other reviews. In this study, with no deterioration after 2 years, the logistic regression was conducted to analyze the relationship of age, sex, brace etc. and treatment result, which further explained the influence of aforementioned factors on the Cobb reduction. **See lines 323 to 326 (Results).**

Comment #43: Clarify how the d-values were computed. Why not describe it as a mixed model anova with a between group factor and a time factor and use appropriate pairwise characteristics.

It appears to be what you did given the output provided. Can you specify which pairwise was requested? LSD? The use of multiple t-tests to detect regression increases the type one error chances.

Response: LSD was used. We have corrected the analyses appropriately. We thank the reviewer for these valuable suggestions.

Comment #44: Results:

Subjects. I disagree that groups were sufficiently similar for bracing.

Response: Our comment was based on the results of the X^2 test which showed no statistical difference. Nevertheless, logistic regression was performed to test whether bracing was an impact on the results. **See lines 323 to 326 (Results).**

Comment #45: From the selection criteria I believed no participant could have started any scoliosis treatment before enrollment. Here I learn that bracing could have been started prior to PSSE. This should be clear from the patient selection criteria.

Response: We thank for reviewer for this valuable suggestion. The patients started with either bracing or exercise would be excluded in this study, please see **S2 (Fig. 1) and lines 132 - 135.**

Comment #46: Can you report brace compliance in both groups?

Response: **Please see line 233 to 235 and Table 1,** because of there was no correlation of bracing with regression effects, the bracing hour, bracing compliance from those 10 participants were not further discussed.

Comment #47: Why only report the percent radiograph refused at 6mth into PSSE. Why not also for the other time points.

Response: All participants completed radiographic assessment on the baseline, 1-year, 1.5-year and 2-year follow-up, therefore per-protocol analysis was performed. Please see **lines 256 – 257. lines 272 to 273 and S2 Fig.1.**

Comment #48: Specify if the following statement applies only to group averages or also to patient individual data: There was no deterioration of the major curvature in either group at the 2-year follow-up.

Response: It applies to both average and individual data. No deterioration was noted for either group. Please see **lines 259 to 260 and Table 2.**

Comment #49: Where you report the F and P-values you need three F values. The key one is for the interaction between group and time, then you also need main effects on groups and on time. (your output show all were not significant.

Response: The analysis was re-performed by a statistician who was blinded to this study. We thank the reviewer for this important comment. Three F values of comparisons in D-value of Cobb and raw data of Cobb, respectively, were clarified and reported. **See lines 263 to 265, table 2,3,4 and lines 275 – 277.**

Comment #50: Report the Sanders secondary analysis as a new paragraph. Announce this interest in objective section as a secondary objective.

Response: The Sanders staging has been replaced by Risser as we needed the Risser for evaluation of progression risk.

Comment #51: If the interaction effect is not significant it is not good statistical practice to explore pairwise within groups with t-tests. IF you had set your mixed method anova with Cobb at baseline, 1 year, 1.5 yrs and 2 yrs you may have detected this effect already. Running a different approach with t-test exposes you to a higher risk of type 1 errors.

Response: Fully agree, beside of analysis of D-value of Cobb reduction over three testing times, we re-performed analysis on the Raw Cobb angle over four testing timepoints which suggested a significant time effects in reducing Cobb angles (repeated one way anova).

Comment #52: It would be possible to use a chi-square analysis to compare the distribution of the improved, stabilized and deteriorated in each group statistically in addition to just report the results. A statistician may be able to determine if the three time points could be compared in a single analysis for these proportions to protect against a type one error.

Response: We have proceeded to the reviewer's suggestions. Unfortunately, the X^2 analysis also showed no significance, therefore, we further analyzed the relationship of influencing factors, including age, brace, sex etc and the regression effects using logistic regression analysis. See **lines 210 to 213 and lines 323 to 326.**

Comment #53: See above. The following opening statement of the discussion is overreaching: This is the first study to investigate the outcomes of different curve types undergoing PSSE treatment for AIS.

Response: The statement has been corrected. Please see **lines 341 -342** (discussion).

Comment #54: You cannot state the following as all your groups received exercises. You did not show superiority to an alternative. And you did not discuss historical controls. Therefore, our results further revealed the superiority of PSSE programs in the stabilization and regression of scoliosis.

Response: We thank the reviewer for the sentiments.

Comment #55: In the discussion you state: This met our original study hypothesis that lumbar major curves are more flexible. I am not sure this is the only possible physiological explanation. You did not measure flexibility per say. Please justify your statement and address possible alternatives as well. Discuss limitations of not having measured flexibility directly.

Response: We thank the reviewer for these suggestions. Please see **lines 369-389** and **lines 433 – 441.**

Comment #56: P13. Compare your results of lumbar effects being better to those of Schreiber et al. I believe they did not find the same curve pattern as the one with the best response. Could it be that some benefits for your thoracic group may have been missed due to not using the chest twister and prone/supine exercises

Response: It is possible, we thank the reviewer for this valuable discussion point. I remember that there were conference papers, presented in SOSORT 2019 in SF, there were evidences showing that the spine responded better during exercise in prone. However, to our knowledge, this evidence was not published yet and unfortunately our study was initiated before 2019. Positions were designed to cope with patients' daily activity as much as possible in this study. In Schreiber's study, they used chest twist for thoracic curve, however, their results also showed the 3CP was significantly correlated with the largest Cobb after treatment, which was explained by the proven higher progression risk was shown in thoracic type. Therefore, it will be not suitable to compare our study with theirs on this point, because they also found the less effects with 3CP. Therefore, <u>we alternatively stated that our study extended their results for the long term effect</u> (**lines 341-344**) Moreover, for the potential variation caused by varied exercises, we further explained in **lines 372-389,** which was more emphasizing on the spinal biomechanics instead of varied positioning during exercise.

Comment #57: Please discuss the limitation of self-reporting compliance and possible recall issues depending on when your participants completed the compliance questionnaires.

Response: Participant need to take a note and sign on his checklist on the day when they performed exercise, then hand the checklist to physiotherapist at their next visit. See **lines 139 – 143,** recall effects were discussed on **lines 405 - 408.**

Comment #58: Limitations. There are more than 4 patterns in RIGO classification. Type 1 and type 2 were not discussed here.

Response: Information added. **See lines 416 to 426.**

Comment #59: Please discuss not monitoring co-interventions such as manipulations, massage, other fitness activities or self-used of off the shelve bracing.

Response: The participants would agree with only PSSE but no alternative exercise before sign the study consent form, but we agree that participants are likely to take another therapy without informing us. Commencing any other treatment were excluded from this study, please see **line 115 and S2 Figure.**

Comment #60: Could you review how many patients in each group experience a change of curve type over time and report which changes occurred.

Response: There were no curve pattern changed in this group of participants. But curve pattern might be changed with long-term exercise and should be identified. Please see **Table 4**

Comment #61: Discuss the imbalanced in the number of braced participant and possible brace compliance effects on results.

Response: Only 10 subjects had brace treatment and half of them only used night time brace. The logistic regression showed bracing did not affect our outcome, therefore, it maybe not very valuable to go in details of this analysis due to our small sample size. Please see **lines 405 – 408.**

Comment #62: Discuss whether after 2 years of follow-up these participants had reached skeletal maturity. How many had reached discharge point or were sufficiently far after peak growth velocity to deem the results final or not.

Response: Fantastic point. **Table 4** was edited to this purpose.

Comment #63: The SRS-SOSORT recommendations propose a number of analysis reporting

guidelines. Could you report Risser signs in your dataset or table 1. Could you report number of patients with curve over 30 at each time point. I believe none exceeded surgery threshold of 45 or 50 but this could be specified.

Response: **Table 1 has been amended** and see an updated **table 4.**

Comment #64: The discussion could compare how your follow-up length and results compared to other Schroth studies. Are your results better than others or similar. Was the progression risk of your cohort similar or worse than others.

Response: Fantastic point, related result and discussion were addressed accordingly. See **lines 339 to 344.**

Comment #65: Please add a paragraph about whether there is a risk of overtreatment in this cohort. Could some of the patients have been left alone and avoid the burden of treatment. I can see that some would argue that for the fact that these patients possibly had no risk of progressing to bracing or surgery. However you showed some notable regression. This may appeal to some participants with small curves at low risk of progression. Still the possible overtreatment issue should be discussed.

Response: We thank the reviewer for this important point. The discussion was amended accordingly. See **lines 353 to 356.**

Comment #66: An important limitation in your study is that you may have been underpowered to detect difference between groups. It would be important to discuss how big a different in effects between curve types may be clinically important. This may be difficult to determine but a discussion of this topic should be presented.

Response: We agree. This has been added to **line 417 to 425 and lines 446 to 449.**

Comment #67: I would recommend against including the following in the discussion as you did not measure flexibility and did not study relation of effects of PSSE with baseline curve magnitude per say. However, further studies are necessary to address the correlation between spinal flexibility and the correction effects of PSSE at different curve magnitudes. IF you wish to keep then please

move earlier in the discussion as suggestions for future research.

Response: We thank the reviewer for this comment. Please see **lines 446 to 449.** We **re-centered** our discussion point in the introduction and discussion, respectively.

Comment #68: I have annotated a few elements of the paper in an uploaded copy.

Response: We thank the reviewer for these comments. Thanks for nice editing, the format was amended accordingly.

Reviewer #3

Comment #1: The objective of this study is to investigate the comparative effectiveness of the PSSE correction effect on the Cobb angle between the thoracic and lumbar curves in AIS subjects. The authors considered a prospective clinical controlled study. While the study objectives sound interesting, a number of shotcomings were observed, in regards to abiding by the CONSORT guidelines for conducting and reporting results of high-quality randomized controlled trials (RCTs).

Response: We thank the reviewer for these comments.

Comment #2: 1. Abstract: The authors state results as: "A significant Cobb angle reduction was observed...", without any statement of p-values, estimated effect size, and its precision as confidence intervals, or CIs (say, 95\%). This can appear confusing, and half-baked to a reader. Check CONSORT checklist for Abstracts reporting of RCTs, and rewrite the Abstract following guidelines.

Response: This was not an RCT study, therefore no needs for randomization etc, nonetheless, the problems in the abstract was recognized and amended.

Comment #3: 2. Methods:

Methods reporting appeared very messy. An orderly manner is suggested, following CONSORT guidelines, without repeating information, such as Trial Design, Participant Eligibility Crtieria and settings, Interventions, Outcomes, sample size/power considerations, Interim analysis and stopping rules, Randomization (details on random number generation, allocation concealment, implementation), Blinding issues, etc. The authors are advised to create separate subsections for each of the possible topics (whichever necessary), and that way produce a very clear writeup.

Response: Thanks for the valuable suggestions. We have revised the formatting.

Comment #4: (a) For instance, the randomization and allocation concealment should be made very clear; the trial staff recruiting patients should not have the randomization list. Randomization should be prepared by the trial statistician, and he/she would not participate in the recruiting. I am confused; was randomization not done during the "Allocation" phase in your CONSORT diagram?

Response: This was not an RCT, it was a CCT hence no randomization was required. Instead we controlled the curve pattern for the study.

Comment #5: (b) I am surprized to see no statement on sample size/power in a manuscript proposing a (clinical) controlled trial. This is really the key here!

Response: We thank the reviewer for the comment. The sample size estimation is in lines 192-196.

Comment #6: (c) t-tests were used for assessing group differences for continuous variables (under the assumption of Normality). What if underlying normality assumptions are violated? Why not non-parametric (robust) tests were proposed?

Response: Related clarification was addressed; normality was measured before commencing the analysis. Please see **lines 195 - 213**.

Comment #7: (d) Similarly, the one-way repeated measures ANOVA may also be

replaced/presented by a nonparametric Friedman-type test. I mean, justification is needed for the underlying Normality assumptions.

Response: These have been clarified in the statistical analysis section.

Comment #8: 3. Results & Conclusions:

(a) The authors should check that any statement of significance should be followed by a p-value in the entire Results section.

Response: We thank the reviewer for this comment. This has been added.

Comment #9: (b) The authors admitted a long list of limitations in their work, notably, the "...unable to blind the subjects, or physiotherapists...". Despite the justification provided, I am not sure how good the trial is! With 40 subjects recruited, the results stated, at best, can only be claimed as from a pilot study. This needs to be clearly stated, and the study cannot be claimed as a nicely planned randomized trial. Also, they need to state that future studies (with larger sample sizes) are warranted to really understand the comparative efficiacy.

Response: We thank the reviewer for these comments. Please see the additions in **lines 410-441**.
