# Supplementary material for: Does curve pattern impact on the effects of physiotherapeutic scoliosis specific exercises on Cobb angles of participants with adolescent idiopathic scoliosis: A prospective clinical trial with two years follow-up
Source: PLoS One. 2021 Jan 25;16(1):e0245829. doi: 10.1371/journal.pone.0245829 (PMC7833215; doi:10.1371/journal.pone.0245829)
Supplement: Attachment — Submitted filename: PONE-D-19-35759_R2_reviewer.pdf [file pone.0245829.s010.pdf]

PLOS ONE

Does curve pattern impact on the effects of physiotherapeutic scoliosis specific exercises on Cobb angles of participants with adolescent idiopathic scoliosis: a prospective clinical trial with two years follow-up

--Manuscript Draft--

Manuscript Number: PONE-D-19-35759R2

Article Type: Clinical Trial

Full Title: Does curve pattern impact on the effects of physiotherapeutic scoliosis specific exercises on Cobb angles of participants with adolescent idiopathic scoliosis: a prospective clinical trial with two years follow-up

Short Title: Does curve pattern impact on effects of PSSE in treating AIS

Corresponding Author: Yunli Fan
University of Hong Kong
Hong Kong, HONG KONG

Keywords: adolescent idiopathic scoliosis; Curve Pattern; Cobb Angle; Physiotherapeutic Scoliosis Specific Exercise; conservative management; Scoliosis Progression; Compliance

Abstract:

Background
Current clinical evidence suggests that a well-planned physiotherapeutic scoliosis specific exercise (PSSE) program is effective for scoliosis regression.

Objectives
We investigated the effect of curve patterns on Cobb angles with PSSE.

Methods
This was a non-randomized prospective clinical trial that recruited participants with adolescent idiopathic scoliosis between January and June 2017. Participants were grouped by curve pattern into major thoracic and major lumbar groups. An outpatient-based PSSE program was conducted with the following schedule of intensive exercise: ≥ 1 session of supervised PSSE per month and > 30min of home exercise 5 days/week in the first 6 months, after which exercise frequency was reduced to 1 session of supervised PSSE every three months and > 30min of home exercise 5 days/week until 2 years after study initiation. Radiographic Cobb angle progressions were identified at the 1, 1.5 and 2-year follow-ups. A mixed model analysis of variance (ANOVA) was performed to examine the differences in Cobb angles between groups at four testing time points. The two-tailed significance level was set to 0.05.

Results
In total, 40 participants were recruited, including 22 with major thoracic curves (5 males and 17 females; mean age 13.5±1.8 years; Cobb angle 18 - 45 degrees) and 18 with major lumbar curves (7 males and 11 females; mean age 12.7±1.7 years; Cobb angle 15 - 48 degrees). Curve regressions, namely the reduction of Cobb angles between 7 to 10 degrees were noted in 9.1% of participants in the major thoracic group; reductions of 6 to 13 degrees were noted in 33.3% of participants in the major lumbar group at the 2-year follow-up. Repeated measurements revealed a significant time effect ($F_{2.2,79.8}=4.1$, $p=0.02$), but no group ($F_{2.2,79.8}=2.3$, $p=0.1$) or time × group ($F_{1,37}=0.97$, $p=0.3$) effects in reducing Cobb angles after 2 years of PSSE. A logistic regression analysis revealed that no correlation was observed between curve pattern and curve regression or stabilization (OR: 0.2, 95% CI: 0.31 - 1.1, $p=0.068$) at the 2-year follow-up.

Conclusion
This was the first study to investigate the long-term effects of PSSE in reducing Cobb angles on the basis of major curve location. No significant differences in correction were observed between major thoracic and major lumbar curves. A regression effect and no curve deterioration were noted in both groups at the 2-year follow-up.

Order of Authors: Yunli Fan

Powered by Editorial Manager® and ProduXion Manager® from Aries Systems Corporation

	Michael KT TO
	Eric HK Yeung
	Jianbin Wu
	Rong He
	Zhuoman Xu
	Ruiwen Zhang
	Guangshuo Li
	Kenneth MC Cheung
	Jason PY Cheung

Response to Reviewers:

REPLY TO REVIEWERS

Re: Ms. No. PONE-D-19-35759 - "Does Curve Pattern Impact on the Effects of Physiotherapeutic Scoliosis Specific Exercises on Cobb angle of Adolescent Idiopathic Scoliosis: A prospective clinical controlled trial with two years follow-up"

Dear Editor Alison Rushton,

The authors would like to thank you and the Reviewers for all of your time and effort devoted to the review of our aforementioned manuscript. The Reviewers' comments were indeed extremely insightful and greatly appreciated. As such, the authors would like to take this opportunity to address each and every concern the Reviewers noted in their review of our submission. In addition, where appropriate, we have also revised our manuscript accordingly. Major changes are made in light gray.

We believe that the Reviewers' comments have helped improve the quality of our manuscript. We hope that you and the Reviewers will find our revised work suitable for publication in PLOS One.

Editor

Recommendation#1：If applicable, we recommend that you deposit your laboratory protocols in protocols.io to enhance the reproducibility of your results. Protocols.io assigns your protocol its own identifier (DOI) so that it can be cited independently in the future.

Response：Thanks for recommendation. This is a clinical trial without laboratory protocols, this trial was online registered and the study protocol was open access on www.chictr.org.cn, trial registration: ChiCTR1900028073.

Reviewer #2

Comment #1: The Title should be corrected and show DOES not DOSE
Does curve pattern impact on the effects of physiotherapeutic scoliosis specific exercises on Cobb angles of participants with adolescent idiopathic scoliosis: a prospective clinical trial with two years follow-up
Response：We apologize for the typo. The title has been corrected.

Comment #2: Specify in the abstract that the study is a non-randomized prospective trial.
Response：Study design is specified in line 17.

Comment #3: In the abstract and the text line 203 The analysis should be announced as a mixed model anova (2 group by N time). Avoid stating 1 way as you have two factors. Group is a between groups factor and time is a within group factor.
Response：Analysis method was renamed accordingly, please see line 24-25.

Comment #4：Your sentence on line 231 to 235 is unclear. There is an important

Powered by Editorial Manager® and ProduXion Manager® from Aries Systems Corporation

	difference between groups in the numbers of patients with brace. Specify that in the text.<br>Response：This was specified on lines 241 to 244.<br><br>Comments #5: Also did all braced patients have a Cheneau? the sentence suggests you only talk about those wearing a Cheneau brace but does not clearly state whether other types were used. Specify at what point correction was measured. Do you mean out of brace at the end of the study or in-brace and if yes then when was in-brace correction measured.<br>Response:<br>1. All braced subjects were wearing Cheneau brace, sorry for this confusion, clarification was made on lines 237 to 239.<br>2.The in-brace correction was the initial in-brace correction (a percentage value of Cobb reduction during an X-ray with the brace fitted on the participant more than two hours), which was measured after a brace adaption period (two weeks). Please see lines 136 to 138 and lines 188 to 190.<br>3.Regarding the Cobb angle at the end of the study, it was measured without brace and participants were required off-bracing at night prior to X-ray measurement in the next day. Please see lines 190 to 191.<br><br>Comment #6: Abstract. The following sentence in the abstract could be replaced by results of your regression. Your objective was focused on effect of curve type. This sentence does not add to this objective: In addition, 65% of 37 participants (n=26) reached skeletal maturity (Risser sign = 5) and achieved significant reductions 38 in progression risk at the 2-year follow-up (Lonstein and Carlson Risk of progression: 0.45 ± 0.32 39 vs. 0.13 ± 0.14, p <0.001).<br>Obviously, patients reaching maturity would have significantly reduced progression risk. If you keep this idea in the abstract, please specify what this means with regards to their curve types or having done exercises.<br>Response: Fantastic suggestion. We re-structured the result of abstract and emphasized result of correlation of curve pattern with curve regression. Please see lines 36 to 38.<br><br>Comment #7: In the consort flow chart please specify the number of cases that were excluded because they did not attend all the prescribed exercises sessions. (specify none did if that is the case)<br>Response: All participants completed prescribed exercise sessions. The clarification was further edited in S2 Fig. CONSORT flow diagram and line 127.<br><br>Comment #8: Figure 2 f. The picture is stretched too wide. Please present in the original aspect ratio.<br>Response: The picture (Fig.2) is updated with original aspect ratio. Please check line 168.<br><br>Comment #9: Page 13 line 203. Specify how you calculated D-value. Did you do for example 6 months minus baseline or basline minus 6 months. Specifying will help interpret mean d-values as indicating improved curves or deterioration.<br>Response: Thanks for this valuable suggestion. Specifying was made on lines 206 to 209.<br><br>Comment #10: P14 Line 206 Please refer to comment above about using mixed model anova and not one way ANOVA.<br>Response: Thanks for this reminding and correction was addressed on line 211.<br><br>Comment #11: L218 It would be interesting to see the distribution of the hypermobility cases excluded in terms of their presenting a thoracic or lumbar dominant pattern given that you suspected a higher relation with single thoracic curves.<br>Response: The distribution of noted hypermobility in this study was reported on lines 224 to 225. We did not reproduce the same distribution as the previous study, yet we did find a few of patients presenting hypermobility regarding the Beighton score. The distribution of hypermobility in AIS can be explained better with a population level, cross-sectional study.<br><br>Comment #12: P14 Is single left lumbar atupical? Single right lumbar would be more

Powered by Editorial Manager® and ProduXion Manager® from Aries Systems Corporation

atypical? Is this an error? Similarly, with single left thoracolumbar. Is this really atypical?
Response: This is a really good question. Proven atypical curve patterns include a single left thoracic curve, a curvature with a loss of kyphosis, a rapid progression and ≤ 6 segmental scoliosis, etc. The single right lumbar and a left thoracolumbar were reported as atypical pattern in previous studies and in which subjects were found having an underlying syrinx presented with a MRI assessment. Therefore, cases with aforementioned curve pattern were excluded to control study heterogeneity yet prescribed with either a neurological assessment or an MRI assessment before commencing any treatments. We cited the related studies, please check lines 225 to 227 and references 35 to 38.

Comment #13: L229 to 231. I continue to disagree that you should compare baseline characteristics between groups statistically. Instead you should report what appears to be clinically important differences and maybe announced that this would be justification to explore these characteristics as covariables later. The study did not plan its sample size to detect such differences. The key is to explore if they play a role in confounding the results later.
Response: Sure, we do agree that defining confounding factors is way more important than comparing the baseline statistically in a clinical trial, Thus, baseline characteristics were reported by descriptive statistics in this updated manuscript. Please check Table 1. In addition, bracing was set as a covariate with repeated measures to evaluate effects of PSSE in reducing Cobb angles.

Comment #14: L230 Please spell out what LCR stands for.
Response: Lonstein Carlson Risk of progression (LCR-value) was firstly explained on the line 205, then the abbreviated term, LCR-value was adopted throughout manuscript.

Comment #15: L231. The imbalance in bracing may not have reached significance but it should be pointed out.
Response: Sure indeed, this was emphasized on the lines 241 to 244.

Comments #16: L237 Table 1. If you keep the p values add which test, they came from in the table legend on in the column with the p-values. This would help the informed reader determine if your test likely had enough power.
Response: We used descriptive statistics for baseline characteristics in this updated manuscript, thus no p value was reported in the table 1, but we adopted t-test and paired t-test to compare the exercise compliance of each year, which would help us on discussing how did exercise compliance affect the outcome. Thus, a table legend was made accordingly on the lines 250 to 252.

Comment #17: L237 Here or in the methods state when the in-brace Cobb angle was measured.
Response: The timepoint of measuring in-brace Cobb angle was stated on the lines 136 to 138.

Comment #18: L237 Refer to 1st year and 2nd year in the exercise compliance please.
Response: Done. Please check the Table 1.

Comment #19: L246 Since in the analysis section you left 2 choices for testing this different please report what test was used to compare the groups here with the p-value.
Response: This is a subgroup analysis to detect any changes of exercise compliance between groups on the 1st and 2nd year follow-up. Hence, an independent t-test, and paired t-test were used to compare the exercise compliance between and within groups. Please check lines 256 to 257.

Comment #20: L248 Similarly. Please report the test used from comparisons within the thoracic group.
Response: This is a subgroup analysis to detect any changes of exercise compliance between groups on the 1st and 2nd year follow-up. Hence, an independent t-test, and paired t-test were used to compare the exercise compliance between and within groups. Please check the lines 256 to 257.

	Comment #21: L267 Table 2. Instead of group labels of 0 and 1 can you spell thoracic and lumbar or use T and L for clarity?<br>Response: Thanks for this suggestion. Revision was made on the Table 2.<br><br>Comment #22: L272 to 279. This analysis offers more information than that of the D-values. I would only report this ANOVA and possibly compared the frequencies of deterioration, stabilized or improved statistically as well. There is in fact a lot of redundancy between the analyses in table 2 and 3.<br>Response: Agree. Thanks for this fantastic suggestion to make our points much clearer. Hence, we withdrew the previous table 2 (repeated measures of D-value) and re-centered our primary analysis on the raw Cobb angles. Please see lines 269 to 286, and the updated table 2.<br><br>Comment #23: L289. It is less clear why this second case is highlighted. Only because of the large baseline curve? If yes specify that this case it highlighted because or the large curve. I was wondering if it was because it was the second case having refused the brace. Please clarify.<br>Response: The second case refused the brace. The clarification was made on the lines 320 - 321.<br><br>Comment #24: L309 To 311. It is redundant to repeat the baseline mean in each of the parentheses. Clarify which test produced the p-values. This set of results is surprising as it would suggest that the interaction should be significant the pattern of differences between timepoints was not the same between the two groups, yet you report that the interactions was not significant. Since the interaction was not significant you should not put emphasis on the different in changes over time between the groups.<br>Response: Thanks for this comment, the redundancy was deleted. Please check the re-structured paragraphs on lines 294 to 321, using descriptive statistics to illustrate the trend of scoliosis regression and stabilization in each group. The repeated measures with Bonferroni adjustment showed a significant time-effect in reducing Cobb angles (raw value) over four testing times. Additionally, post hoc analysis showed the significant reductions of Cobb were occurred on the 1.5- and 2-year follow-up, respectively. Therefore, we used descriptive statistics, additionally to illustrate how many participants in which group showed clinical curve regression. In case of we withdrew the repeated measures of D-value of Cobb angles, hence the previous redundancy was completely deleted.<br><br>Comment #25: L280 to 287. 312. To 317 Ideally the frequency results of the two groups would be reported together in terms of whether the distributions of improved, stable or deteriorated were similar or not between groups. Specify which test was used to test for those differences in the distributions.<br>Response: Fantastic suggestion, please see the re-structured paragraphs on lines 294 to 321. The distribution of scoliosis progression, stabilization and regression was analysed using Chi-square analysis. Please see lines 296 to 298.<br><br>Comment #26: L321 322. Focusing on a reduction in the progression risk is possibly misusing the formula of Lonstein and Carlson since it uses age and Risser so heavily and you did not report the formula in your paper. The change in angles would not play a clear role and this may mislead readers to think the reduction is due to the PSSE when maturity plays a big role. Their study possibly did not examine patients starting this late to really use the equation at this stage of treatment. If you keep this analysis, please report the full formula and add a discussion related to maturation in the discussion in relation to this reduced risk.<br>Response: Fantastic point, thanks for pointing it out, we realized that comparing LCR-value after two years follow-up was inappropriate. Therefore, we withdrew the comparison of LCR-value at the 2-year follow-up and replaced it to a description of how much participants reached skeletal maturity (Risser 5) and presented with a curve less than 30 degrees. Please see lines 294 to 295 and Table 3.<br><br>Comment #27: L328. Please add units for Age, the Cobb angle mean lines<br>Response: Done, Thanks for this suggestion. Please see line 323 (Table 3).

Powered by Editorial Manager® and ProduXion Manager® from Aries Systems Corporation

Comment #28: L323 Your study likely did not have the power to examine so many variables in the logistic regression. Since your focus is on the curve type. Can you report the OR for the association between curve type and the 2 year curve outcomes? Once you enter multiple categorical predictors there really isn't a lot of observations with the regression outcome to determine prediction estimates.
Response: Nice point, thanks for pointing it out, we now only enrolled curve pattern and bracing (since we enrolled bracing into repeated measures) into the logistic regression model to further illustrate the effects of bracing and curve pattern in scoliosis regression with PSSE. Please see lines 299 to 302. However, this logistic regression was underpowered since this study was not originally designed for a regression analysis, thus, we acknowledged this limitation on the lines 449 to 454.

Comment #29: L351 to 353. You cannot claim you demonstrated Superiority of PSSE. You did not compare PSSE to an alternate intervention. You showed all patients receiving PSSE have avoided curve progression but not superiority to other treatments or to natural history (unless you refer to historical control and predictions given the progression risk.)
Response: Agree, we replaced "superiority of PSSE" by "effectiveness of PSSE" in preventing scoliosis progression. Please see lines 358 to 359.
Comment #30: L359 to 361. Again, I disagree with emphasizing the change in progression risk. I do not believe the formula can be used this way. Further once again you omit to emphasize that much of this reduce regression risk is due to the change in age and Risser. It is misleading to imply the change is mostly due to the effect of PSSE and reduced Cobb angles. It is good indeed that curves did not progress, and patients are now close to maturity but here it would be more relevant to refer to natural history data showing how patients with certain curve magnitude at maturity progress into adulthood. EG.
Response: Fantastic point, thanks for pointing it out, we realized that comparing LCR-value after two years follow-up was inappropriate. Therefore, we withdrew the comparison of LCR-value at the 2-year follow-up and replaced it to a description of how much participants reached skeletal maturity (Risser 5) and presented with a curve less than 30 degrees. Please see lines 366 to 368 and Table 3.

Comment #31: L366 to 369 again you emphasise a different in the patterns of curve angles changes over time between groups when you ANOVA suggested that there was no interaction. There could be data distribution issues affecting the results here. Which ANOVA model did you end up using? Was your data meeting all other assumptions than sphericity?
Response: Sorry for this confusion, agree that if there were no interaction effects in reducing Cobb angles, it was inappropriate to further compare variables between groups. In this study, the significance was only detected in the within-subjects comparison, having a time effect in reducing Cobb angles for all participants (observed power 0.7, lines 275 - 278). A separate repeated analysis with a Bonferroni adjustment (control type I error) further revealed that the significant reduction of Cobb angles was dominant in the lumbar group (observed power 0.95, lines 282 - 283). Therefore, we rewrote this part (please see lines 371 to 372). Levene's test indicated that Cobb angles at the baseline met homogeneity of variance assumption ($p=0.5$). Mauchly test indicated that the assumption of sphericity was violated ($\chi2 =32.1$, $df =5$, $p < 0.001$), hence, the degrees of freedom were corrected using Greenhouse-Geisser estimates of sphericity. Please see lines 271 to 276. The assumption of Greenhouse-Geisser was met with an Epsilon value 0.72.

Comment #32: L371 What do you refer to when stating "Progression value was comparable between groups at the study initiation"?
Response: Sorry for the confusion, the comparable here means the proportion was similar between groups, the clarification was made on lines 376 to 378.

Comment #33: L372 373 Since you did not measure spinal flexibility please refer instead to "by the assumed more important flexibility of the lumbar spine than the thoracic spine.
Response: Fantastic points. Please see the reediting on lines 378 to 379.

Comment #34: L378 to 381. I would avoid suggesting that muscle actions have a bigger role for lumbar corrections and that only spiral breathing play a role in the

	thoracic regions. Modern Schroth instruction does not make assumptions about what muscles do or how corrections are done it simply offers a variety of cues and correction instructions and facilitation technique to get the desired movement without emphasis on how.Maybe the key is that breathing is more related to thoracic ok. The axial elongation, side shift are done in both regions. The difficulty with breathing is likely relevant.<br>Response: Nice points, thanks for this explanation, the revised discussion was made on the lines 385 to 387.<br><br>Comment #35: L405. Please cite Kuru et al for the effects of supervision vs not.<br>Response: cited. Thanks for this recommendation. Please check reference 49.<br><br>Comment #36: L417 There are more types in the whole BSPTS classification. Maybe instead rephrase to suggest that your sample included patients representing 5 different types from 8 in the BSPTS classification.<br>Response: Nice points, thanks for this explanation, the revised discussion was made on lines 423-424.<br><br>Comment #37: L435 Refer to hypothesized higher flexibility or previously shown higher flexibility. You did not show that in your study.<br>Response: Thanks for this reminding, we acknowledge this limitation on lines 444 to 445.<br><br>Comment #38: In this limitation section please clearly acknowledge that your regression is underpowered.<br>Response: Indeed. The acknowledgement was made on lines 448 to 454.<br><br>Comment #39: In the data supplementary file please replace the variable name by study id. Remove initials and replace by participant id numbers to fully comply with confidentiality of study data.<br>Response: Done, thanks for this important recommendation. Please check the updated S4 File. Study data.<br><br>Comment #39: I have added edits in the text using the PDF comment function with strikethrough for deletions and insertions in blue.<br>Response: We have revised the format accordingly. Deeply appreciate your detailed comments.
**Additional Information:**	
**Question**	**Response**
**Financial Disclosure**<br><br>Enter a financial disclosure statement that describes the sources of funding for the work included in this submission. Review the submission guidelines for detailed requirements. View published research articles from *PLOS ONE* for specific examples.<br><br>This statement is required for submission and **will appear in the published article** if the submission is accepted. Please make sure it is accurate.	Funding Project: Sanming Project of Medicine (SZSM201612055) “Team of Excellence in Spinal Deformities and Spinal Degeneration Diseases” in Shenzhen, Guangdong province, China.<br><br>Receiver: Kenneth MC Cheung<br><br>Professor Kenneth Cheung participated in the methodology and preparation of the manuscript.

**Unfunded studies**<br>Enter: *The author(s) received no specific funding for this work.*<br><br>**Funded studies**<br>Enter a statement with the following details:<br>• Initials of the authors who received each award<br>• Grant numbers awarded to each author<br>• The full name of each funder<br>• URL of each funder website<br>• Did the sponsors or funders play any role in the study design, data collection and analysis, decision to publish, or preparation of the manuscript?<br>• **NO** - Include this sentence at the end of your statement: *The funders had no role in study design, data collection and analysis, decision to publish, or preparation of the manuscript.*<br>• **YES** - Specify the role(s) played.<br><br>* typeset	
**Competing Interests**<br><br>Use the instructions below to enter a competing interest statement for this submission. On behalf of all authors, disclose any competing interests that could be perceived to bias this work—acknowledging all financial support and any other relevant financial or non-financial competing interests.<br><br>This statement **will appear in the published article** if the submission is accepted. Please make sure it is accurate. View published research articles from *PLOS ONE* for specific examples.	The authors have declared that no competing interests exist.

**NO authors have competing interests**<br><br>Enter: *The authors have declared that no competing interests exist.*<br><br>**Authors with competing interests**<br><br>Enter competing interest details beginning with this statement:<br><br>*I have read the journal's policy and the authors of this manuscript have the following competing interests: [insert competing interests here]*<br><br>* typeset	
**Ethics Statement**<br><br>Enter an ethics statement for this submission. This statement is required if the study involved:<br><br>• Human participants<br>• Human specimens or tissue<br>• Vertebrate animals or cephalopods<br>• Vertebrate embryos or tissues<br>• Field research<br><br>Write "N/A" if the submission does not require an ethics statement.<br><br>General guidance is provided below. Consult the submission guidelines for detailed instructions. **Make sure that all information entered here is included in the Methods section of the manuscript.**	Human Subject Research<br>•Name of Committe:<br>The Ethic Committe Board of The University of Hong Kong - Shenzhen Hospital<br><br>•Approval number:No.:伦[2016]91<br>Original protocol: GNSF201603<br><br>•Form of consent obtained (written/oral) : Written

**Format for specific study types**<br><br>**Human Subject Research (involving human participants and/or tissue)**<br>• Give the name of the institutional review board or ethics committee that approved the study<br>• Include the approval number and/or a statement indicating approval of this research<br>• Indicate the form of consent obtained (written/oral) or the reason that consent was not obtained (e.g. the data were analyzed anonymously)<br><br>**Animal Research (involving vertebrate animals, embryos or tissues)**<br>• Provide the name of the Institutional Animal Care and Use Committee (IACUC) or other relevant ethics board that reviewed the study protocol, and indicate whether they approved this research or granted a formal waiver of ethical approval<br>• Include an approval number if one was obtained<br>• If the study involved *non-human primates*, add *additional details* about animal welfare and steps taken to ameliorate suffering<br>• If anesthesia, euthanasia, or any kind of animal sacrifice is part of the study, include briefly which substances and/or methods were applied<br><br>**Field Research**<br><br>Include the following details if this study involves the collection of plant, animal, or other materials from a natural setting:<br>• Field permit number<br>• Name of the institution or relevant body that granted permission	
**Data Availability**<br><br>Authors are required to make all data underlying the findings described fully available, without restriction, and from the time of publication. PLOS allows rare exceptions to address legal and ethical concerns. See the PLOS Data Policy and FAQ for detailed information.	Yes - all data are fully available without restriction

A Data Availability Statement describing where the data can be found is required at submission. Your answers to this question constitute the Data Availability Statement and **will be published in the article**, if accepted.<br><br>**Important:** Stating 'data available on request from the author' is not sufficient. If your data are only available upon request, select 'No' for the first question and explain your exceptional situation in the text box.<br><br>Do the authors confirm that all data underlying the findings described in their manuscript are fully available without restriction?	
**Describe where the data may be found in full sentences. If you are copying our sample text, replace any instances of XXX with the appropriate details.**<br><br>• If the data are **held or will be held in a public repository**, include URLs, accession numbers or DOIs. If this information will only be available after acceptance, indicate this by ticking the box below. For example: *All XXX files are available from the XXX database (accession number(s) XXX, XXX.)*.<br>• If the data are all contained **within the manuscript and/or Supporting Information files**, enter the following: *All relevant data are within the manuscript and its Supporting Information files.*<br>• If neither of these applies but you are able to provide **details of access elsewhere**, with or without limitations, please do so. For example:<br><br>*Data cannot be shared publicly because of [XXX]. Data are available from the XXX Institutional Data Access / Ethics Committee (contact via XXX) for researchers who meet the criteria for access to confidential data.*<br><br>*The data underlying the results presented in the study are available from (include the name of the third party*	All relevant data are within the manuscript and its Supporting Information files.<br><br>data (same as in supporting information files) will be availbale on an open public access if paper recieved: ResMan<br>www.medresman.org

and contact information or URL).<br>• This text is appropriate if the data are owned by a third party and authors do not have permission to share the data.<br><br>* typeset	
Additional data availability information:	Tick here if the URLs/accession numbers/DOIs will be available only after acceptance of the manuscript for publication so that we can ensure their inclusion before publication.

Manuscript Click here to access/download;Manuscript;manuscript.docx

Title: Does curve pattern impact on the effects of physiotherapeutic scoliosis specific exercises on Cobb angles of participants with adolescent idiopathic scoliosis: a prospective clinical trial with two years follow-up

Authors

1. Yunli Fan[1,2,3]
2. Michael KT To[2]
3. Eric HK Yeung[3]
4. Jianbin Wu[1]
5. Rong He[3]
6. Zhuoman Xu[3]
7. Ruiwen Zhang[3]
8. Guangshuo Li[3]
9. Kenneth MC Cheung[2]
10. Jason PY Cheung[2*]

[1]Department of Orthopedics, The University of Hong Kong – Shenzhen Hospital, Shenzhen, Guangdong Province, People's Republic of China

[2]Department of Orthopaedics and Traumatology, The University of Hong Kong, Hong Kong SAR, China

[3]Department of Physiotherapy, The University of Hong Kong – Shenzhen Hospital, Shenzhen, Guangdong Province, People's Republic of China

*** Corresponding author:**

Name	Cheung, Jason Pui Yin
Department	Department of Orthopaedics and Traumatology
Institution	The University of Hong Kong
Country	Hong Kong Special Administration Region, China
Email	cheungjp@hku.hk

Does curve pattern impact on the effects of physiotherapeutic scoliosis specific exercises on Cobb angles of participants with adolescent idiopathic scoliosis: a prospective clinical trial with two years follow-up

Yunli Fan[1,2,3], Michael KT To[2], Eric HK Yeung[3], Jianbin Wu[1], Rong He[3], Zhuoman Xu[3], Ruiwen Zhang[3], Guangshuo Li[3], Kenneth MC Cheung[2], Jason PY Cheung[2*]

Abstract

Background

Current clinical evidence suggests that a well-planned physiotherapeutic scoliosis specific exercise (PSSE) program is effective for scoliosis regression.

Objectives

We investigated the effect of curve patterns on Cobb angles with PSSE.

Methods

This was a non-randomized prospective clinical trial that recruited participants with adolescent idiopathic scoliosis between January and June 2017. Participants were grouped by curve pattern into major thoracic and major lumbar groups. An outpatient-based PSSE program was conducted with the following schedule of intensive exercise: ≥ 1 session of supervised PSSE per month and > 30min of home exercise 5 days/week in the first 6 months, after which exercise frequency was reduced to 1 session of supervised PSSE every three months and > 30min of home

exercise 5 days/week until 2 years after study initiation. Radiographic Cobb angle progressions were identified at the 1, 1.5 and 2-year follow-ups. A mixed model analysis of variance (ANOVA) was performed to examine the differences in Cobb angles between groups at four testing time points. The two-tailed significance level was set to 0.05.

Results

In total, 40 participants were recruited, including 22 with major thoracic curves (5 males and 17 females; mean age 13.5±1.8 years; Cobb angle 18 - 45 degrees) and 18 with major lumbar curves (7 males and 11 females; mean age 12.7±1.7 years; Cobb angle 15 - 48 degrees). Curve regressions, namely the reduction of Cobb angles between 7 to 10 degrees were noted in 9.1% of participants in the major thoracic group; reductions of 6 to 13 degrees were noted in 33.3% of participants in the major lumbar group at the 2-year follow-up. Repeated measurements revealed a significant time effect ($F_{2.2,79.8}$=4.1, p=0.02), but no group ($F_{2.2,79.8}$=2.3, p=0.1) or time × group ($F_{1,37}$=0.97, p=0.3) effects in reducing Cobb angles after 2 years of PSSE. A logistic regression analysis revealed that no correlation was observed between curve pattern and curve regression or stabilization (OR: 0.2, 95% CI: 0.31 - 1.1, p=0.068) at the 2-year follow-up.

Conclusion

This was the first study to investigate the long-term effects of PSSE in reducing Cobb angles on the basis of major curve location. No significant differences in correction were observed between major thoracic and major lumbar curves. A regression effect and no curve deterioration were noted in both groups at the 2-year follow-up.

Trial Registration: ChiCTR1900028073

*** Corresponding author:** cheungjp@hku.hk

[1]Department of Orthopedics, The University of Hong Kong – Shenzhen Hospital, Shenzhen, Guangdong Province, People's Republic of China

[2]Department of Orthopaedics and Traumatology, The University of Hong Kong, Hong Kong SAR, China

[3]Department of Physiotherapy, The University of Hong Kong – Shenzhen Hospital, Shenzhen, Guangdong Province, People's Republic of China

Introduction

Adolescent idiopathic scoliosis (AIS) is a three-dimensional spinal deformity with an unknown etiology, characterized by lateral deviation in the frontal plane, axial rotation in the horizontal plane and an abnormal sagittal curvature.[1] Surgery is typically recommended if a

spinal curve reaches 50 degrees, because such curves are associated with a continued progression risk into adulthood.[1-3] Surgical fusion causes spinal stiffness and should be avoided if possible.[3] Thus, the goal of conservative managements including bracing and exercises, is to prevent spinal deformity deterioration past the operative threshold.[4]

Options for nonsurgical management vary widely and depend on the prognostic evaluation of curve progression.[4] Curve type is an established risk factors of scoliosis progression.[5] In particular, a thoracic curve with a larger Cobb angle has a higher likehood of progression than single lumbar or thoracolumbar curves do.[2] Therefore, understanding the effect of curvature on interventions can help clinicians select appropriate treatments for patients.

Bracing is commonly prescribed for moderate scoliosis, and its corrective effect is influenced by the location of structural curves.[6, 7] In addition, scoliosis-specific exercise is a nonoperative option that is well received by patients and families.[8] Several systematic reviews and randomized controlled trials have reported the positive effects of physiotherapeutic scoliosis specific exercise (PSSE) on slowing curve progression as well as improving cosmetic and quality of life;[9-12] however, the relationship of curve location with correction effects has not been clearly discussed in these studies, in which small samples of participants have been recruited and

followed up over the short-term.[13-15] In particular, one 6-month long randomized control trial (RCT) revealed a significant correlation between thoracic curvature with an imbalanced pelvis (n=15) and the largest curves after Schroth treatment;[13] one 4-month long RCT demonstrated the greater Cobb reduction was noted in the thoracic region (n=20) after body awareness exercise.[14] Therefore, a clinical trial, in which curve pattern is controlled, is warranted to investigate the long-term effects of curve pattern and PSSE on reducing Cobb angles. This is valuable for clinical practice because the deterioration is possible after initial improvement in short-term follow-up.

According to current evidence from PSSE studies,[9, 10, 16] ~~the~~ Schroth is the most used PSSE approach. It adopts a specific respiratory technique, asymmetrical breathing in the diagonal direction, to achieve vertebral and rib cage derotation.[17] It utilizes muscle activation and emphasizes core muscle stabilization in a corrected posture throughout the day to change habitual postures and improve spinal alignment.[17, 18] However, the influence of curve magnitude on Schroth method outcomes is unclear. The spinal deformity profile that leads to optimal outcomes with Schroth exercise is also unclear. Thus, this study investigated the long-term therapeutic effects of Schroth exercises on major thoracic and major lumbar curves, respectively. We hypothesized that patients with lumbar curves, which have a lower reported risk of curve

progression and higher spinal segmental flexibility, benefit more from long-term PSSE treatment than do patients with major thoracic curves. The current results can provide physiotherapists with additional information to develop individualized exercises based on curve magnitude. These results can also form the basis for further RCTs evaluating the relationship between curve patterns and PSSEs.

Materials and methods

Study design

This was a non-randomized prospective clinical trial in which participants with AIS were recruited consecutively between January and June 2017 from one scoliosis clinic at the University of Hong Kong - Shenzhen Hospital (HKU - SZH), China. This study obtained ethical approval by the institutional review board of the HKU - SZH with reference number: GNSF201603 (**S1** File). Individuals in this manuscript have given written informed consent to publish these case details. The authors confirm that all ongoing and related trials for this intervention are registered in the Chinese clinical trial registry (www.chictr.org.cn, trial registration: ChiCTR1900028073) (**S1** File). Participants and legal guardians signed a written informed consent for PSSE treatment and participants were followed up for 2 years thereafter. The inclusion criteria were age 10 to 16 years; bone immaturity in terms of Risser sign was less than 5;[19] Cobb angles[20] from 10 degrees to 50 degrees; and complete attendance for all

physiotherapy sessions. Exclusion criteria were diagnoses other than AIS, disabilities or systemic illnesses that prevent participants from performing exercise, hypermobility (Beighton score[21] greater than 4) and previous treatment for AIS. Joint hypermobility (JHM) is reported to have a higher prevalence in patients with single curves than in those with double-curve scoliosis; and this may affect physiotherapy outcomes.[22] Therefore, patients with hypermobility were excluded from this study to control for systematic error introduced by JHM differences between curve patterns. Participants were categorized into two groups according to their whole spine radiographic characteristics:[23, 24] participants with 3C (major thoracic with or without a minor lumbar curve) or N3N4 curves (double, well balanced curves) formed the major thoracic group; and those with single lumbar, single thoracolumbar, or 4C curves (a major lumbar curve with a minor thoracic) comprised the major lumbar group (**S2** Fig 1).

S2 Fig 1. CONSORT flow diagram.

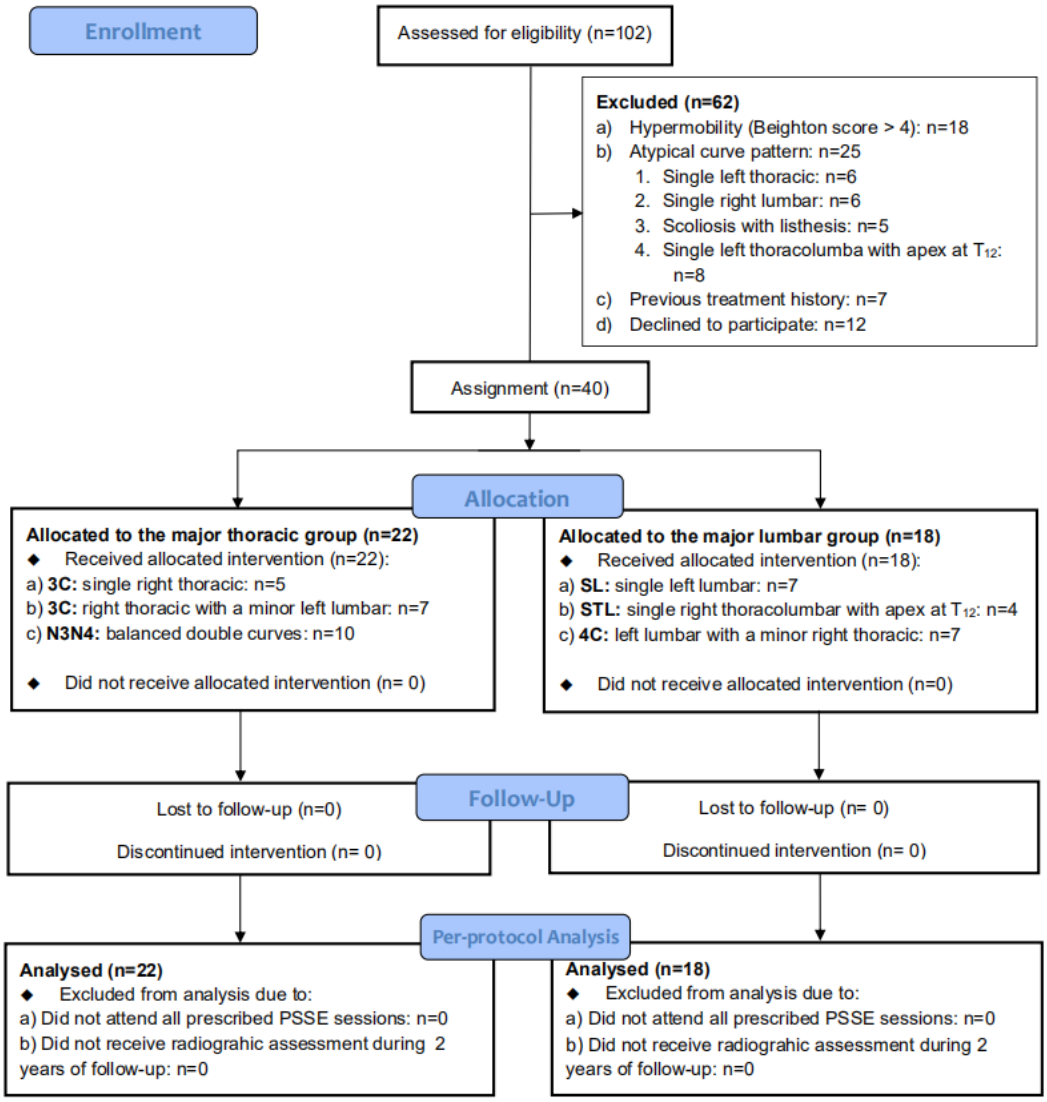

Study intervention

All participants participated in a 6-month intensive PSSE program supervised by two PSSE-Schroth certified physiotherapists (Barcelona Scoliosis Physical Therapy School [BSPTS] approach).[23] Supervised treatment during the 6 months occurred ≥1 time per month for 1 hour each time. Participants were instructed to perform >30min sessions of home exercise at least ≥5

times per week. For participants who met Scoliosis Research Society bracing criteria, Cheneau bracing was prescribed prior to the PSSE program;[25] after an adaption period (2 weeks) of wearing a brace, an initial in-brace angle[26-28] was measured by a spine surgeon who was blinded to this study, using the X-ray with a brace fitted on the participant more than two hours.[28] Bracing compliance was reported by patients on their monthly treatment adherence checklist (**S3** File). After the first 6 months of PSSE, all participants reduced their frequency of supervised PSSE to once every 3 months to review their exercises, which they performed for 2 years after completing the PSSE program. During this time, the frequency of home exercise was maintained. Attendance for supervised PSSE in an outpatient setting was recorded in the hospital's prospective database system. Participants were requested to document home exercise and bracing compliance on daily basis using a treatment adherence checklist (**S3** File), and handed it to a physiotherapist assistant who was blinded to this study every month during the first 6-month study period; then they were required to submit the checklist every 3 months thereafter. Exercise compliance was calculated in hours per week per year for analysis. The PSSE intervention consisted of 50 breaths per exercise. Short semihanging with caudal-cranial spine lengthening (Fig 2. c), corrective exercise in side-lying with shoulder counter traction (Fig 2. d), corrective exercise in sitting with pelvic pulling strategy on the side of the weak point and shoulder counter traction (Fig 2. e), corrective exercise in standing with pelvic derotation and shoulder counter traction (Fig 2. f) were

prescribed for participants in the major thoracic group. Short semihanging with caudal-cranial spine lengthening (Fig 3. c), corrective exercise in side-lying with passive correction on the lumbar prominence (Fig 3. d), corrective exercise in standing with shoulder counter traction (Fig 3. e), muscle cylinder in standing for opening lumbar concavity (Fig 3. f) and the corrective exercise in sitting with shoulder counter traction (Fig 3. g) were prescribed to participants in the major lumbar group. The difference between the two groups' exercises was that a muscle cylinder was prescribed only to participants with major lumbar curves. The muscle cylinder exercise in a kneeling position is typically prescribed to patients with major thoracic curves;[17] however, patients commonly complain that it causes knee pain. Therefore, considering the potential adverse effects during long-term follow-up, the muscle cylinder in a kneeling position was not prescribed in this study. Moreover, dynamic exercises, such as Schroth walking was not used in this study because of limited space in our center. In addition, participants were asked to record and report any discomfort, such as muscle fatigue and muscle sprain, to the on-duty physiotherapist during the study period.

Fig 2. Exercises demonstration for major right thoracic curves

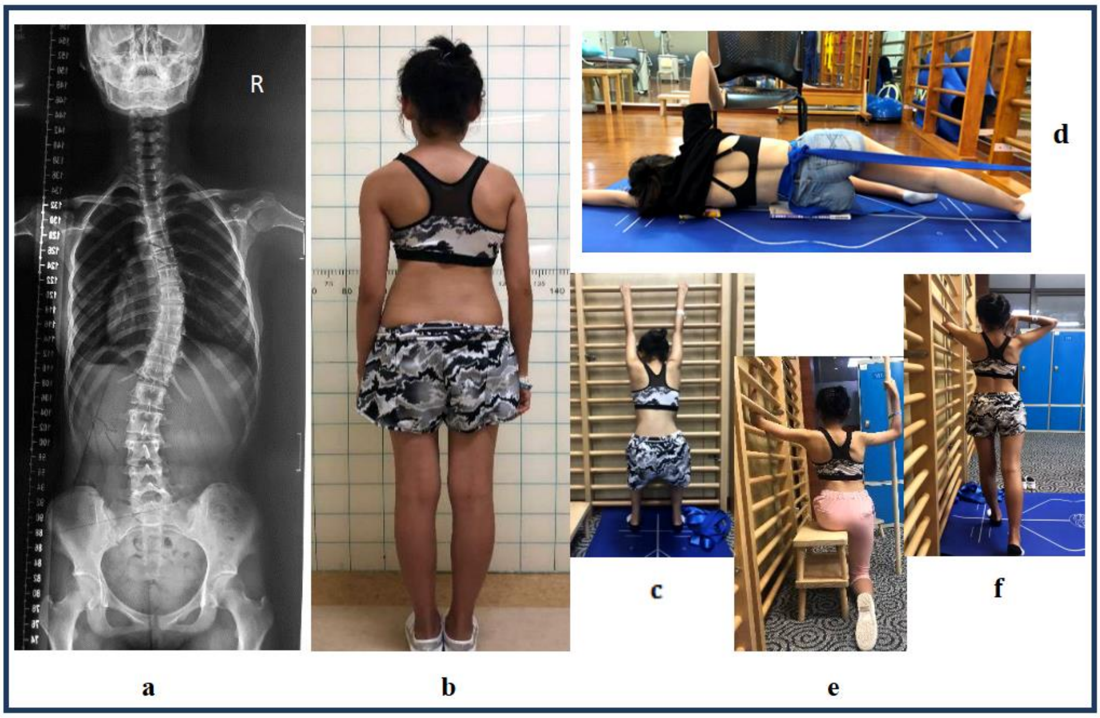

Fig 2: a: a major right thoracic (T4 to 12) with a Cobb angle of 30 degrees. **b:** posterior posture view of the participant. **c:** short semihanging with caudal-cranial spine lengthening. **d:** corrective exercise in side-lying with shoulder counter traction. **e:** corrective exercise in sitting with pelvic pulling strategy on the side of weak point and shoulder counter traction. **f:** corrective exercise in standing with pelvic derotation and shoulder counter traction.

Fig 3. Exercises demonstration for major left lumbar curves

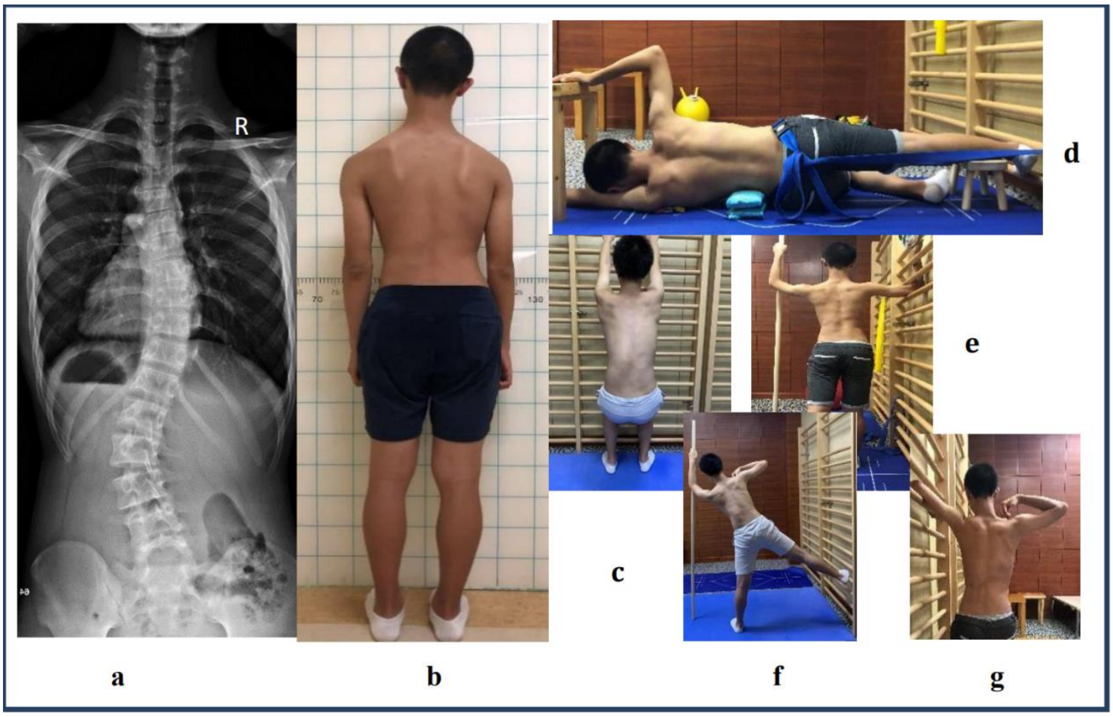

Fig 3: **a:** a major left lumbar curve (T12 to L4) with a Cobb angle of 48 degrees. **b:** posterior posture view of the participant. **c:** short semihanging with caudal-cranial spine lengthening. **d:** corrective exercise in side-lying with passive correction on the lumbar prominence. **e:** corrective exercise in standing with shoulder counter traction. **f:** muscle cylinder in standing for opening lumbar concavity. **g:** **c**orrective exercise in sitting with shoulder counter traction.

Outcome measurement

Participants underwent whole-spine radiography in a standing position with arms slightly abducted at the sides of body. The Cobb angles of major curves were assessed by a spine surgeon who was blinded to this study. Cobb angles were recorded in the initial assessment and every 6 months after the PSSE program until the 2-year follow-up. An in-brace Cobb angle was

measured using a radiography with the brace fitted on the participant. A percentage value of the initial in-brace correction ($IBC = \frac{Cobb\_in-brace}{Cobb\_baseline}$) was compared between groups to evaluate the bracing effect.[29] Participants remained out of their braces overnight prior to obtaining radiography ~~during~~ two years of follow-up. [28] Curve regression was defined as a decrease in the Cobb angle by 6 degrees or more, stabilization was defined as a <6 degrees change in Cobb angle, and deterioration was defined as a Cobb angle increase of 6 degrees or more.[2, 30]

Statistics

Sample size estimation

To ~~obtain~~ an effect size of 0.50, 80% power for a two-tailed test, and a correlation of 0.6 for repeated measures of Cobb angles over 4 time points, 13 participants were required for each group.[31] Considering a possible 35% dropout rate during the 2-year study period, each group were planned to have 18 participants (**S2** Fig 1).

Statistical analysis

Descriptive statistics with 95% Confidence Interval (CI) were calculated for baseline variables including demography, radiography, exercise compliance and Lonstein and Carlson Risk of progression value ($\mathrm{LCR\text{-}value} = \frac{\mathrm{Cobb} - 3 \times \mathrm{Risser}}{\mathrm{Age}}$)[32] for all participants (Table 1).[33] Changes in Cobb angles, in the form of the difference value

[$\text{D-value} = (\text{Cobb}_{\text{\_6-month/1-/1.5-/2-year}}) - (\text{Cobb}_{\text{\_baseline}})$] were measured to detect curve progression (D-value ≥ 6 degrees), stabilization (D-value < 6 degrees, or D-value > -6 degrees) and regression (D-value ≤ -6 degrees) between groups;[34] raw Cobb angles were repeatedly compared over four testing time points to detect time, group and time × group interaction effects in reducing Cobb angles. A mixed model analysis of variance (ANOVA) was performed if the normality assumptions of Cobb angles were met whereas a nonparametric Friedman-type test would be performed if normality was violated. Post hoc pairwise comparison was conducted if any significant differences were detected in either within-subjects or between-subjects comparisons. A Logistic regression was performed to detect correlations of brace treatment and curve pattern with the regression, stabilization or deterioration after 2 years of PSSE. The data were analyzed using SPSS version 20.0 (IBM, Chicago, IL). The level of significance was set to 0.05 with a two-tailed test.

Results

Participants

In total, 102 patients were screened to confirm eligibility, after which 62 patients were excluded for the following reasons: hypermobility (Beighton score > 4) was detected in 18 patients (single right thoracic: n=7, single left lumbar: n=5; right thoracic with a minor lumbar: n=6); 25 patients had reported, atypical curve patterns[35-38] (single left thoracic: n=6,[36]

single right lumbar: n=6,[38] scoliosis presented with vertebral listhesis: n=5,[37] single left thoracolumbar with apex at T_{12}: n=8[35]); 7 patients were previously treated with either bracing or exercise; 12 patients declined to participate in this study. Therefore, 40 participants were recruited and all completed follow-up (**S2** Fig 1). The major thoracic group had 22 participants (5 males and 17 females). Of them, 12 had 3C curves (single right thoracic: n=5; right thoracic with a minor left lumbar: n=7) and 10 had N3N4 curves (balanced right thoracic with a left lumbar). The major lumbar group had 18 participants (7 males and 11 females). Of them, 7 had single left lumbar curves, 4 had right thoracolumbar curves with apex at T_{12} and 7 had 4C curves (left lumbar with a minor right thoracic). The mean age of participants in the major thoracic and major lumbar groups was 13.5±1.8 years and 12.7±1.7 years, respectively (Table 1). At baseline, the two groups did not differ statistically in age, sex, Risser sign (0-2 / 3-4), proportion of sub-curvature types, LCR-value, or initial Cobb angle of the major curve (Table 1). Ten participants with brace treatment who all started Cheneau orthosis[39] before commencing the PSSE and achieved 37% of initial in-brace correction[27] in each group (Table 1). In particular, 5 of them were wearing a night-time brace only (≤8 hours/day) whereas 5 were wearing a full-time brace (≥21 hours/day) (Table 1). Two participants in the major lumbar group refused brace treatment due to cosmetic concerns, which introduced an imbalance in bracing between groups. Thus, bracing was set as a covariate with the repeated analysis to evaluate effects of PSSE in reducing

Cobb angles.

Table 1. Baseline characteristics of participants

	**Major thoracic group** (95% confidence interval)	**Major Lumbar group** (95% confidence interval)
**Age** (years: Mean ± SD)	13.5 ± 1.8 (12.8 - 14.6)	12.7 ± 1.7 (12.6 - 13.7)
**Sex**		
Female	17 (77.3%)	11 (61.1%)
Male	5 (22.7%)	7 (38.9%)
**Risser sign**		
0-2 / 3-4	9 / 13	9 / 9
**Initial Cobb Angle** (min-max)	18 - 45	15 - 48
(degrees: Mean ± SD)	28.5 ± 8.4 (24.5 - 32.5)	26.8 ± 10.1 (22.4 - 31.2)
**Curve pattern**		
3C / SL or STL	12	11
N3N4 / 4C	10	7
**Braced** (yes)	7	3*

In-brace hours (hours/day)	13.6 ± 6.9	16.7 ± 7.5*
full-time brace (≥ 21hr/day): n	3	2
night-time brace (≤ 8hr/day): n	4	1*
**Initial in-brace Cobb angle**		
(degrees: Mean ± SD)	23 ± 4.4	26 ± 6
**Initial in-brace correction rate (%)**	37	37
**Initial progression risk**		
Lonstein and Carlson Risk of progression	44 %	46%
**Exercise compliance**		
1st year: hours/week	5.5 ± 2.2	5.5 ± 2.1
2nd year: hours/week	3.0 ± 0.8[§ ¶]	4.1± 2.1

3C: A single right thoracic curve or a major right thoracic with a minor left lumbar. **SL:** A single left lumbar. **STL:** A single right thoracolumbar. N3N4: double curves but well balanced. **4C:** A major left lumbar with a minor right thoracic. * two participants refused brace treatment due to cosmetic concerns and introduced an imbalance in bracing between groups. § An independent *t-test* revealed a significant difference between groups. ¶ A paired *t-test* revealed a significant difference in the major thoracic group.

Exercise compliance

The two groups differed significantly in exercise compliance during the second year of follow-up (independent *t-test*, p=0.04) (Table 1). In addition, mean exercise compliance of the major thoracic group was significantly decreased in the second year (paired *t-test*, p<0.01) (Table 1); the mean exercise compliance of the major lumbar group was 5.5±2.1 hours/week in the first year and slightly decreased to 4.1±2.1 hours/week in the second year (Table 1). There were no adverse effects detected during this study period.

Intervention effects

In total, 23 participants (57.5%) with Cobb angle < 30 degrees refused to repeat radiography at the 6-month follow-up after the PSSE program because of radiation exposure concerns; therefore, no comparison of participants before and at the 6-month follow-up was performed. Nonetheless, all participants completed radiography at the 1-year, 1.5-year and 2-year follow-up, respectively. Full set of raw data is available in the **S4** File.

A per-protocol analysis was performed to analyze the raw value of Cobb angles over four testing time-points between groups. Bracing (hours/day) was set as a covariate with a mixed model repeated analysis since an imbalance of bracing was noted at study initiation. Levene's test indicated that Cobb angles at the baseline met homogeneity of variance assumption (p=0.5);

Mauchly test indicated that the assumption of sphericity was violated (χ^2 =32.1 , df =5 , p < 0.001); hence, the degrees of freedom were corrected using Greenhouse-Geisser estimates of sphericity.[40] With current sample size (n=40) and by adjusting for bracing as a covariate, the repeated analysis showed a significant within-subjects time effect ($F_{2.2,79.8}$=4.1, p=0.02), but no group ($F_{2.2,79.8}$=2.3, p=0.1), bracing ($F_{2.2,79.8}$=1.4, p=0.3) or time × group ($F_{1,37}$=0.97, p=0.3) effects were detected in reducing Cobb angles after 2 years of treatment (Table 2). A post hoc pairwise comparison (within-subjects) with a Bonferroni adjustment revealed a significant reduction of Cobb angle, regardless of curve pattern, was observed ~~on~~ the 1.5-year (27.7±9.1 degrees vs 25.4±8.6 degrees, p=0.015) and the 2-year (27.7±9.1 ±9.1 degrees vs 25.4±8.2 degrees, p<0.01) follow-up (Table 2). A separate repeated analysis with a Bonferroni adjustment further suggested that the significant reduction of Cobb angle was observed in the major lumbar group ($F_{3,51}$=6.2, p<0.01), in which a reduction of Cobb angle was noted after training for one and a half years (26.8±10.1 degrees vs 23.3±9.4 degrees, p=0.04) with a maintained reduction after two years of follow-up (26.8±10.1 degrees vs 22.9±9.2 degrees, p<0.01) (Table 2).

Table 2. Per-protocol repeated measures of Cobb angles over four testing time points

Testing timepoint	Group	Mean	Std. Deviation	95% Confidence Interval	Minimum	Maximum	*P*
**Baseline**	T	28.5	8.4	24.8-32.2	18	45	-

	L	26.8	10.1	21.8-31.8	15	48	-
	Total	27.7	9.1	25.6-29.9	16	46	-
**1-year**	T	27	7.2	23.8-30.2	18	38	0.910
	L	24.7	9.4	20.0-29.4	10	42	0.476
	Total	26.0	8.2	23.8-28.0	14	40	0.107
**1.5-year**	T	27.1	7.7	23.7-30.5	10	40	1.0
	L	**23.3**[*]	9.4	18.6-28.0	10	45	**0.043**
	Total	**25.4**[ƚ]	8.6	23.2-27.3	10	43	**0.015**
**2-year**	T	27.5	7.0	24.4-30.5	15	40	1.0
	L	**22.9**[*]	9.2	18.4-27.5	10	43	**0.009**
	Total	**25.4**[ƚ]	8.2	23.4-27.1	13	42	**0.005**

A Bonferroni adjustment was performed with repeated measures to control familywise Type I error.

Group T: major thoracic group. **Group L:** major lumbar group. * significantly differ from the baseline in the major lumbar group. ƚ significantly differ from the mean value at baseline for all participants.

Overall, we observed 20% with regression (n=8) and 80% with stabilization in this study, in which 65% of participants (n=26) reached bone maturity (Risser: 5) after two years of PSSE and no curve pattern was changed (Table 3). A chi-square analysis revealed that there was no difference (χ^2 =3.6, df =1, p=0.057) in distributions of curve regression and stabilization at the 2nd year follow-up between groups. No deterioration of the major curvature occurred in either group

(both average and individual data) at the 2-year follow-up. A logistic regression analysis further revealed that there was no correlation of bracing (OR: 0.69, 95% CI: 0.10-4.82, p=0.71) or curve patterns (OR: 0.2, 95% CI: 0.31 - 1.1, p=0.068) with curve regression or stabilization at the 2-year follow-up.

In the major thoracic group, descriptive statistics revealed that curve regressed (Cobb angles: -8.8±2.5 degrees) in 18.2% of participants and stabilized (Cobb angle: -2.9±1.6 degrees) in 81.8%, none deteriorated in the first year. Only 2 participants maintained curve regression in the second year, which resulted 9.1% of participants showed regression (Cobb angles: -8.5±2.1 degrees) (Table 3). Thus, up to 90.9% of participants stabilized (Cobb angles: -3.1±1.6 degrees), and no deterioration was detected (Table 3).

In the major lumbar group, descriptive statistics revealed that curves regressed (Cobb angles: -9.5±4.9 degrees) in 11.1%, stabilized (Cobb angles: -3.4±1.2 degrees) in 83.3% and deteriorated by 7 degrees in one subject after the first year of training. In the second year of follow-up, up to 33.3% of participants had improved Cobb angles (Cobb angles: -8.2±2.5 degrees), while 66.7% stabilized (Cobb angles: -3.2±1.6 degrees), and no deterioration was noted (Table 3). Of the participants with curve regression in the first year, one maintained the curve regression,

reducing Cobb angle ~~of~~ 13 degrees, and one only gained an additional degree in the second year. Moreover, one participant with a single left lumbar curve of 31 degrees (Fig. 4: a) who refused brace treatment obtained a Cobb angle of 23 degrees (Fig. 4: b) after one and a half years of PSSE. In addition, another participant with a single right thoracolumbar curve of 44 degrees who refused brace treatment (Fig. 5: a) obtained a Cobb angle of 31 degrees (Fig. 5: b) after two years of PSSE.

Table 3 Descriptive statistics of demographic and radiographic variables at the 2-year follow-up.

**The 2-year follow-up**	**Thoracic curves (n=22)**	**Lumbar curves (n=18)**
**Age** (years: Mean ± SD)	15.6 ± 1.8	14.7 ± 1.7
**Risser sign** 0-2,3-4,5 (n)	0,4,18	0, 10,8
**Cobb angle** (degrees: Mean ± SD)	27.5 ± 7.0	22.9 ± 9.2
≥ 30 degrees: n (%): min – max	8 (36.4%): 31° - 40°	5 (27.8%): 30° - 43°
<30 degrees: n (%): min – max	14 (63.6%):15°- 28°	13 (72.2%): 10° -27°
a) **Regressed**: n (%): min to max of D-value	2 (9.1%): -6° to -10°	6 (33.3%): -6° to -13°
b) **Stabilized**: n (%): min to max of D-value	22 (90.9%): 5° to -5°	12 (66.7%): 3° to -5°
c) **Progressed**: n (%): min to max of D-value	0	0
**Curve pattern:** 3C / SL or STL (n)	12	11
N3N4 / 4C (n)	10	7

Fig 4. Radiographic assessment (posterior anterior view) of one participant with a single left lumbar scoliosis who refused brace treatment for cosmetic reasons.

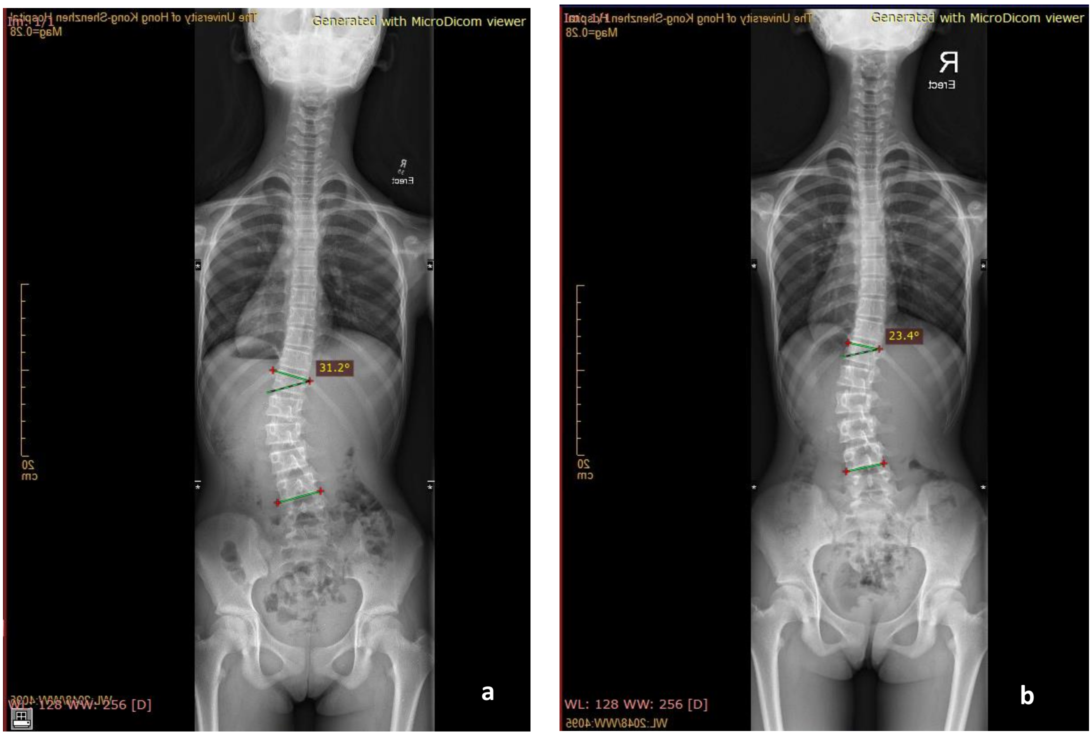

a: an initial Cobb angle of 31 degrees from T12 to L4. **b:** a Cobb angle of 23 degrees from T12 to L4 at the 1.5-year of follow-up.

Fig 5. Radiographic assessment (posterior anterior view) of one participant with a single right thoracolumbar scoliosis who refused brace treatment for cosmetic reasons.

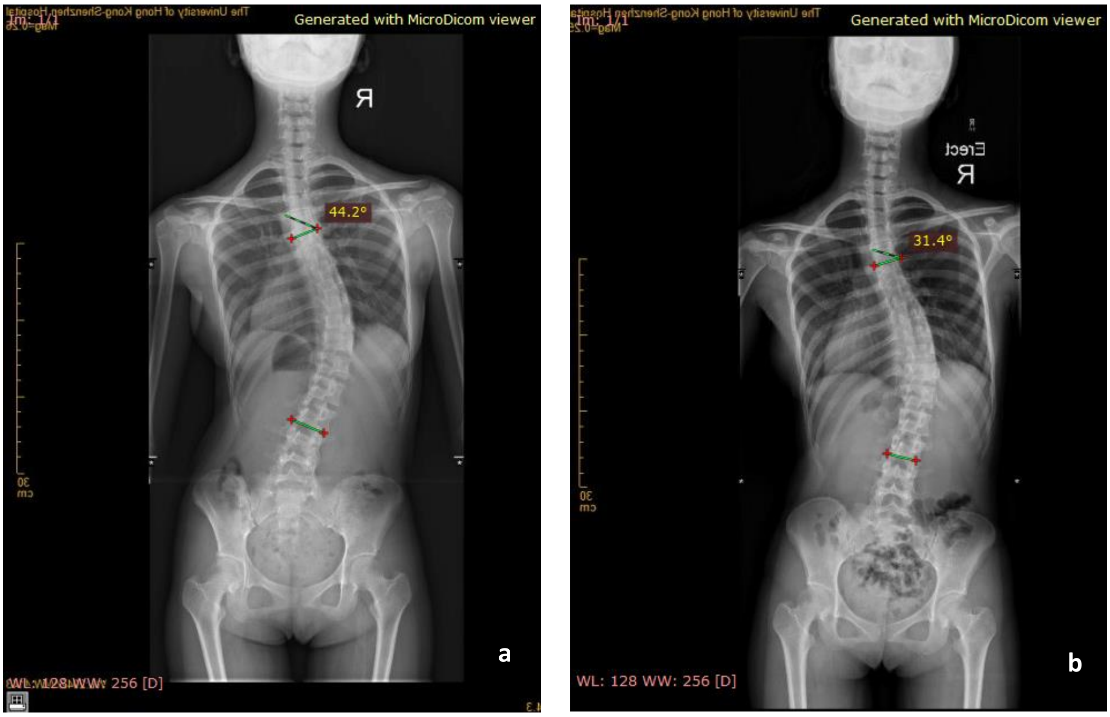

a: an initial Cobb angle of 44 degrees from T5 to L2. **b:** a Cobb angle of 31 degrees from T5 to L2 at the two-year follow-up.

Discussion

This was the first study to investigate the long-term outcomes of patients with different AIS curve patterns undergoing PSSE treatment. The findings of this study indicate no significant difference in Cobb angle changes between groups after regular PSSE for two years, regardless of the curve pattern. This implies that the curve pattern does not significantly impact the effects of PSSE in participants with AIS. A previous 6-month long study suggested that the curve pattern, a thoracic curvature with an imbalanced pelvis (n=15), is significantly correlated with the largest sum of curves after Schroth exercise, and that study further explained this might be due to a higher

risk of progression in thoracic scoliosis.[13] However, with a short-term follow-up and limited sample size, that study could not draw any firm conclusions on the relationship of curve pattern and Schroth exercise in treating AIS. Our results extend this discussion with a longer follow-up period, and curve patterns were controlled before PSSE commenced. In this study, no curve progression was observed in either group, suggesting that a well-planned PSSE program can prevent scoliosis deterioration regardless of curve patterns. This result is consistent with previous findings that PSSE reduces curve progression in patients with mild curve.[41-43] Compared with those studies, we observed regression effects in larger curves and with a longer follow-up period. Moreover, participants in our study presented a higher progression risk (Table 1: LCR-value: 44% vs 46%) at the study initiation; additionally, participants with brace treatment and all received the same initial in-brace correction of 37% improvement. The lack of deterioration may be attributable to the effects of both bracing and PSSE; however, because comparable initial in-brace correction explained the effects of bracing, the effects from the PSSE could be highlighted in our study. Therefore, our results revealed the effectiveness of PSSE programs in curve stabilization and regression of scoliosis for patients with mild to moderate scoliosis. However, a risk of overtreatment exists because patients with only mild curves may not experience progression. Therefore, the burden of treatment should be avoided as much as possible through periodical assessments.[13] Optimal time points for assessment require future study.

After two years of follow-up, no clinical deterioration was detected in either group, indicating a promising effect of PSSE on the prevention of scoliosis progression. Moreover, the regression effect accompanied by a notable decease of progression risk after 2 years. In particular, 65% of participants reached skeletal maturity (Risser 5) and 68% of participants presented <30 degrees of Cobb angle after two years of treatment (Table 3). This is promising because of curve less than 30 degrees are unlikely to progress after skeletal maturity.[2] In particular, a decrease of exercise compliance at the second year may have led to less of a regression effect at the 2-year follow-up in the major thoracic group; a significant time effect in reduction of Cobb angles was only noted for participants with the major lumbar curves; moreover, a difference was observed as early as one and a half years after training, and reductions were maintained at the end of the study period. This might be parallel with our original study hypothesis that major lumbar curves benefited more from long-term exercise in terms of having a lower progression risk; however, this should be interpreted with caution because progression values, in terms of LCR-value was 44% with thoracic curves and 46% with lumbar curves, were comparable between groups at the study initiation. Hence, this observed promising result with lumbar curves might be explained by the assumed more important flexibility of the lumbar spine than the thoracic spine, confounded with a better exercise compliance and its associated effects of good core lumbar muscle stabilization.

The superior flexibility of the lumbar spine compared to the thoracic spine has been proven in biomechanical studies[44-46]. The lumbar spine is mainly stabilized by core muscles that are flexible, stretchable and respond to external forces very well, whereas the stabilization mechanism in the thoracic spine is reliant on the rib cage, which contributes to rigidity and stability[45-47]. Additionally, according to the general principles of the Schroth method, corrective strategies including side shift and axial elongation are done in both thoracic and lumbar regions, whereas spiral breathing is specifically adopted in the thoracic region[17]. Spiral breathing is difficult to learn and hard to control for most participants with AIS due to the requirement of breathing diagonally and asymmetrically not only in the frontal plane but also in the horizontal plane. Therefore, it is relatively easier for participants to correct the lumbar curvature via pelvic positioning, lengthening the curve concavity and stabilizing it in the proper position. In addition, stabilization effects from core muscles are crucial for maintaining correction in lumbar spines[17, 47]. Therefore, the lumbar curvature may respond better to the three-dimensional correction forces from a musculoskeletal functional perspective; however, real-time feedback of the spine during different corrective exercises requires future study.

In our study, significant differences between groups in exercise compliance were noted in the second year. Higher compliance in the major lumbar curve might contribute to the larger

Cobb angle reduction. This extends the results of some previous long-term studies of mild scoliosis that reported the only exercise intensity without reporting compliance.[16, 48] These previous studies have not addressed the relationship of compliance and treatment effects, which is of clinical value to define exercise protocols for subjects with PSSE treatment. In addition, our study revealed that an outpatient home-based PSSE program is a feasible protocol with reasonable compliance. The parents were asked to send monthly videos documenting the exercises performed to the Schroth therapist, which provided a reasonable variation control with respect to exercise quality and compliance. The frequency of such home-based programs was collected via a treatment adherence checklist, and exercise compliance was interpreted by how long participants spent on the PSSE program. This study calculated the supervised PSSE sessions and home exercise sessions in a sum number, in the form of the total hours per week. Hence, we were unable to analyze specifically whether supervised PSSE is superior to home exercises in curve correction despite previous accounts of such a relationship[49-52]. Besides, the bracing compliance was self-reported, and this might introduce recall effects, which can be improved if using a pressure sensor to monitor bracing hours. Nonetheless, only 10 participants wore a brace with 5 of which were wearing nighttime brace only, hence, bracing would not cause a big distraction in our study.

This study had a few inevitable limitations in addition to the small sample size. First, no

untreated control group was included for comparison of intervention effects. However, because of the proven benefits of PSSE on AIS [11-13], it was unethical to leave participants without treatment for as long as 2 years. Thus, the benefit observed may have been influenced by natural recovery of the condition. However, the progression risk at study initiation was evaluated with approximately 45% of progression rate, and with a moderate Cobb angles of 29 degrees with major thoracic and 27 degrees with major lumbar curves, hence, the natural recovery was unlikely in our study. Second, we only enrolled participants representing 5 different types from 8 in the BSPTS classification.[24] The BSPTS concept categorizes scoliosis into 3C (A1, A2 and A3), 4C (B1 and B2), N3N4, single lumbar/thoracolumbar and double thoracic.[23, 24] For participants having a double curve type such as N3N4 or 4C, the minor curve will cause limits to overstretching and twisting of the concavity from the major curve that weaken the effects of correction in the major curve. However, the proportion of curves pattern in this study was similar (Table 1: 40.9% vs 44.4%) between the two groups, which could not cause large variations in the data. Besides, there were only 6 participants with single right thoracic curve and no double upper thoracic curves were presented in this study, therefore, it would decrease the observed power if we extra analysed the single thoracic curves independently. However, a comparison of the effects of PSSE for all eight curve types separately remains valuable for further study. Third, we were unable to blind the participants or physiotherapists due to the nature of PSSE studies, the exercise was prescribed and

performed based on the understanding of the curve pattern by both therapists and patients, which may introduce methodological bias but also limited systematic errors such as learning effects. Performing exercise precisely is crucial to reach and maintain treatment effect, therefore, blinding of subjects and therapists was commonly sacrificed in most PSSE studies.[10, 12] However, the frequency and dosage of both supervised and home programs were strictly fixed, and we blinded the result assessor which controlled bias in the statistical analysis. Finally, we assumed the lumbar spine responded more to PSSE because of its lower progression rate and with proven better segmental spinal flexibility than the thoracic spine. However, because of radiation exposure concerns, no bending radiographic assessment conducted to quantify spinal flexibility before the PSSE program. Thus, this study could not interpret the relationship between spinal flexibility and PSSE. Nonetheless, this study excluded participants with hypermobility that may cause systematic error during exercise. This controlled for potential heterogeneity when the spine started to respond to exercise. However, future study should address the relationship of spinal flexibility with PSSE, to define the correlation of curve location, segmental flexibility and PSSE in treating AIS. In addition to repeated measures of Cobb angles (raw value), we adopted a logistic regression analysis to investigate the correlation of curve pattern with scoliosis progression after 2 years of PSSE using a clinical significance threshold (D-value of a change in Cobb angle: ≥ 6 degrees). This showed a value of clinical practice, but the analysis was underpowered regarding the small

sample size.[53] Thus, a study with a bigger sample size is required to detect the correlation of curve pattern with scoliosis progression after PSSE treatment.

Conclusion

PSSE treatment outcomes did not differ by thoracic or lumbar curve type. In general, PSSE with reasonable exercise compliance has positive effects on preventing scoliosis progression. However, further studies are necessary to address the correlation between spinal flexibility and the correction effects of PSSE when different curve magnitudes, curve types and levels are involved. The cosmetic and quality of life outcomes of PSSE should also be studied. This study formed the base for future larger scale studies and randomized trials.

Supporting information

S1 File. Study protocol. (PDF)

S2 Fig. CONSORT flow diagram. (DOCX)

S3 File. Treatment adherence checklist. (PDF)

S4 File. Study data. (XLSX)

Acknowledgments

This study was financially supported by the Sanming Project of Medicine (SZSM201612055) “Team of Excellence in Spinal Deformities and Spinal Degeneration Diseases” and the Seeding Project (HKUSZH201902042) of the HKU - SZH in Shenzhen, Guangdong

Province, China.

Authors' contributions

YF and JPYC designed the study. JW and HR recruited participants. YF and EHKY performed PSSE teaching. ZX, GL and RZ collected data. MKTT performed statistics. YF drafted the manuscript. JPYC supervised the study and final edited the manuscript. MKTT, KMCC and JPYC obtained funding support. All authors read and approved the submitted version of the manuscript.

References:

1. Weinstein SL, Dolan LA, Cheng JC, Danielsson A, Morcuende JA. Adolescent idiopathic scoliosis. Lancet. 2008;371(9623):1527-37. Epub 2008/05/06. doi: 10.1016/s0140-6736(08)60658-3. PubMed PMID: 18456103.
2. Weinstein SL. The Natural History of Adolescent Idiopathic Scoliosis. J Pediatr Orthop. 2019;39(Issue 6, Supplement 1 Suppl 1):S44-s6. Epub 2019/06/07. doi: 10.1097/bpo.0000000000001350. PubMed PMID: 31169647.
3. Coe JD, Arlet V, Donaldson W, Berven S, Hanson DS, Mudiyam R, et al. Complications in spinal fusion for adolescent idiopathic scoliosis in the new millennium. A report of the Scoliosis Research Society Morbidity and Mortality Committee. Spine (Phila Pa 1976). 2006;31(3):345-9.
4. Korbel K, Kozinoga M, Stoliński Ł, Kotwicki T. Scoliosis Research Society (SRS) criteria and Society of Scoliosis Orthopaedic and Rehabilitation Treatment (SOSORT) 2008 guidelines in non-operative treatment of idiopathic scoliosis. Polish orthopedics and traumatology. 2014;79:118-22.
5. Dolan LA, Weinstein SL, Abel MF, Bosch PP, Dobbs MB, Farber TO, et al. Bracing in Adolescent Idiopathic Scoliosis Trial (BrAIST): Development and Validation of a Prognostic Model in Untreated Adolescent Idiopathic Scoliosis Using the Simplified Skeletal Maturity System. Spine Deform. 2019;7(6):890-8. e4.
6. Almansour H, Pepke W, Bruckner T, Diebo BG, Akbar M. Three-Dimensional Analysis of Initial Brace Correction in the Setting of Adolescent Idiopathic Scoliosis. Journal of clinical medicine. 2019;8(11):1804.
7. Thompson RM, Hubbard EW, Jo CH, Virostek D, Karol LA. Brace Success Is Related to Curve Type

in Patients with Adolescent Idiopathic Scoliosis. J Bone Joint Surg Am. 2017;99(11):923-8. Epub 2017/06/08. doi: 10.2106/jbjs.16.01050. PubMed PMID: 28590377.

8. Negrini S, Carabalona R. Social acceptability of treatments for adolescent idiopathic scoliosis: a cross-sectional study. Scoliosis. 2006;1:14. Epub 2006/08/26. doi: 10.1186/1748-7161-1-14. PubMed PMID: 16930488; PubMed Central PMCID: PMCPMC1560163.

9. Burger M, Coetzee W, du Plessis LZ, Geldenhuys L, Joubert F, Myburgh E, et al. The effectiveness of Schroth exercises in adolescents with idiopathic scoliosis: A systematic review and meta-analysis. S Afr J Physiother. 2019;75(1):904. Epub 2019/06/18. doi: 10.4102/sajp.v75i1.904. PubMed PMID: 31206094; PubMed Central PMCID: PMCPMC6556933.

10. Day JM, Fletcher J, Coghlan M, Ravine T. Review of scoliosis-specific exercise methods used to correct adolescent idiopathic scoliosis. Arch Physiother. 2019;9:8. Epub 2019/08/30. doi: 10.1186/s40945-019-0060-9. PubMed PMID: 31463082; PubMed Central PMCID: PMCPMC6708126.

11. Fan Y, Ren Q, To MKT, Cheung JPY. Effectiveness of scoliosis-specific exercises for alleviating adolescent idiopathic scoliosis: a systematic review. BMC Musculoskelet Disord. 2020;21(1):495. Epub 2020/07/29. doi: 10.1186/s12891-020-03517-6. PubMed PMID: 32718339; PubMed Central PMCID: PMCPMC7385878.

12. Thompson JY, Williamson EM, Williams MA, Heine PJ, Lamb SE. Effectiveness of scoliosis-specific exercises for adolescent idiopathic scoliosis compared with other non-surgical interventions: a systematic review and meta-analysis. Physiotherapy. 2019;105(2):214-34. Epub 2019/03/03. doi: 10.1016/j.physio.2018.10.004. PubMed PMID: 30824243.

13. Schreiber S, Parent EC, Khodayari Moez E, Hedden DM, Hill DL, Moreau M, et al. Schroth Physiotherapeutic Scoliosis-Specific Exercises Added to the Standard of Care Lead to Better Cobb Angle Outcomes in Adolescents with Idiopathic Scoliosis - an Assessor and Statistician Blinded Randomized Controlled Trial. PLoS One. 2016;11(12):e0168746. Epub 2016/12/30. doi: 10.1371/journal.pone.0168746. PubMed PMID: 28033399; PubMed Central PMCID: PMCPMC5198985.

14. Yagci G, Ayhan C, Yakut Y. Effectiveness of basic body awareness therapy in adolescents with idiopathic scoliosis: A randomized controlled study1. J Back Musculoskelet Rehabil. 2018;31(4):693-701. Epub 2018/04/10. doi: 10.3233/bmr-170868. PubMed PMID: 29630516.

15. Yagci G, Yakut Y. Core stabilization exercises versus scoliosis-specific exercises in moderate idiopathic scoliosis treatment. Prosthet Orthot Int. 2019;43(3):301-8. Epub 2019/01/11. doi: 10.1177/0309364618820144. PubMed PMID: 30628526.

16. Liu D, Huang S, Yu X, Yang J, Xuan X, Yang J, et al. Effects of Specific Exercise Therapy on Adolescent Patients with Idiopathic Scoliosis: A Prospective Controlled Cohort Study. Spine (Phila Pa 1976). 2020. Epub 2020/02/26. doi: 10.1097/brs.0000000000003451. PubMed PMID: 32097267.

17. Lehnert-Schroth C. [Schroth's three dimensional treatment of scoliosis]. ZFA (Stuttgart). 1979;55(34):1969-76. Epub 1979/12/10. PubMed PMID: 547573.

18. Lehnert-Schroth C. [The "four-curved" scoliosis. The effect on the additional lumbosacral spinal curve by the three dimensional Schroth's scoliosis treatment]. ZFA (Stuttgart). 1981;57(32):2227-31. Epub 1981/11/20. PubMed PMID: 7314859.

19. Little DG, Sussman MD. The Risser sign: a critical analysis. Journal of Pediatric Orthopaedics. 1994;14(5):569-75.

20. Cobb JR. Scoliosis—Quo Vadis? JBJS. 1958;40(3):507-10.

21. Smits-Engelsman B, Klerks M, Kirby A. Beighton score: a valid measure for generalized hypermobility in children. J Pediatr. 2011;158(1):119-23, 23.e1-4. Epub 2010/09/21. doi: 10.1016/j.jpeds.2010.07.021. PubMed PMID: 20850761.

22. Czaprowski D, Kotwicki T, Pawłowska P, Stoliński L. Joint hypermobility in children with idiopathic scoliosis: SOSORT award 2011 winner. Scoliosis. 2011;6(1):22.

23. Berdishevsky H, Lebel VA, Bettany-Saltikov J, Rigo M, Lebel A, Hennes A, et al. Physiotherapy scoliosis-specific exercises–a comprehensive review of seven major schools. Scoliosis Spinal Disord. 2016;11(1):20.

24. Rigo MD, Villagrasa M, Gallo D. A specific scoliosis classification correlating with brace treatment: description and reliability. Scoliosis. 2010;5(1):1.

25. Negrini S, Atanasio S, Fusco C, Zaina F. Effectiveness of complete conservative treatment for adolescent idiopathic scoliosis (bracing and exercises) based on SOSORT management criteria: results according to the SRS criteria for bracing studies-SOSORT Award 2009 Winner. Scoliosis. 2009;4(1):19.

26. Almansour H, Pepke W, Bruckner T, Diebo BG, Akbar M. Three-Dimensional Analysis of Initial Brace Correction in the Setting of Adolescent Idiopathic Scoliosis. J Clin Med. 2019;8(11). Epub 2019/10/31. doi: 10.3390/jcm8111804. PubMed PMID: 31661811; PubMed Central PMCID: PMCPMC6912396.

27. Dolan LA, Weinstein SL, Abel MF, Bosch PP, Dobbs MB, Farber TO, et al. Bracing in Adolescent Idiopathic Scoliosis Trial (BrAIST): Development and Validation of a Prognostic Model in Untreated Adolescent Idiopathic Scoliosis Using the Simplified Skeletal Maturity System. Spine Deform. 2019;7(6):890-8.e4. Epub 2019/11/17. doi: 10.1016/j.jspd.2019.01.011. PubMed PMID: 31731999; PubMed Central PMCID: PMCPMC6939758.

28. Li M, Wong M, Luk KD, Wong KW, Cheung KM. Time-dependent response of scoliotic curvature to orthotic intervention: when should a radiograph be obtained after putting on or taking off a spinal orthosis? Spine. 2014;39(17):1408-16.

29. Chan SL, Cheung KM, Luk KD, Wong KW, Wong MS. A correlation study between in-brace correction, compliance to spinal orthosis and health-related quality of life of patients with Adolescent Idiopathic Scoliosis. Scoliosis. 2014;9(1):1-. doi: 10.1186/1748-7161-9-1. PubMed PMID: 24559234.

30. Malfair D, Flemming AK, Dvorak MF, Munk PL, Vertinsky AT, Heran MK, et al. Radiographic evaluation of scoliosis. American Journal of Roentgenology. 2010;194(3_supplement):S8-S22.

31. Fitzmaurice GM, Laird NM, Ware JH. Applied longitudinal analysis: John Wiley & Sons; 2012.

32. Lonstein J, Carlson J. The prediction of curve progression in untreated idiopathic scoliosis. J Bone Jt Surg. 1984;3(2):1061-71.
33. Mishra P, Pandey CM, Singh U, Gupta A, Sahu C, Keshri A. Descriptive statistics and normality tests for statistical data. Ann Card Anaesth. 2019;22(1):67-72. doi: 10.4103/aca.ACA_157_18. PubMed PMID: 30648682.
34. Gstoettner M, Sekyra K, Walochnik N, Winter P, Wachter R, Bach CM. Inter- and intraobserver reliability assessment of the Cobb angle: manual versus digital measurement tools. Eur Spine J. 2007;16(10):1587-92. Epub 2007/06/05. doi: 10.1007/s00586-007-0401-3. PubMed PMID: 17549526.
35. Barnes P, Brody J, Jaramillo D, Akbar J, Emans J. Atypical idiopathic scoliosis: MR imaging evaluation. Radiology. 1993;186(1):247-53.
36. Davids JR, Chamberlin E, Blackhurst DW. Indications for magnetic resonance imaging in presumed adolescent idiopathic scoliosis. JBJS. 2004;86(10):2187-95.
37. Malfair D, Flemming AK, Dvorak MF, Munk PL, Vertinsky AT, Heran MK, et al. Radiographic evaluation of scoliosis: review. AJR Am J Roentgenol. 2010;194(3 Suppl):S8-22. Epub 2010/03/05. doi: 10.2214/ajr.07.7145. PubMed PMID: 20173177.
38. Wang W, Zhu Z, Zhu F, Sun C, Wang Z, Sun X, et al. Different curve pattern and other radiographical characteristics in male and female patients with adolescent idiopathic scoliosis. Spine (Phila Pa 1976). 2012;37(18):1586-92. Epub 2012/03/07. doi: 10.1097/BRS.0b013e3182511d0c. PubMed PMID: 22391440.
39. De Giorgi S, Piazzolla A, Tafuri S, Borracci C, Martucci A, De Giorgi G. Chêneau brace for adolescent idiopathic scoliosis: long-term results. Can it prevent surgery? Eur Spine J. 2013;22 Suppl 6(Suppl 6):S815-S22. Epub 2013/09/17. doi: 10.1007/s00586-013-3020-1. PubMed PMID: 24043341.
40. van Ginkel JR, Kroonenberg PM. Analysis of Variance of Multiply Imputed Data. Multivariate Behav Res. 2014;49(1):78-91. doi: 10.1080/00273171.2013.855890. PubMed PMID: 24860197.
41. Weiss HR, Lohschmidt K, el-Obeidi N, Verres C. Preliminary results and worst-case analysis of in patient scoliosis rehabilitation. Pediatr Rehabil. 1997;1(1):35-40. Epub 1997/01/01. doi: 10.3109/17518429709060940. PubMed PMID: 9689236.
42. Weiss HR, Weiss G. Curvature progression in patients treated with scoliosis in-patient rehabilitation--a sex and age matched controlled study. Stud Health Technol Inform. 2002;91:352-6. Epub 2004/10/02. PubMed PMID: 15457754.
43. Weiss HR, Weiss G, Petermann F. Incidence of curvature progression in idiopathic scoliosis patients treated with scoliosis in-patient rehabilitation (SIR): an age- and sex-matched controlled study. Pediatr Rehabil. 2003;6(1):23-30. Epub 2003/05/15. doi: 10.1080/1363849031000095288. PubMed PMID: 12745892.
44. Hajihosseinali M, Arjmand N, Shirazi-Adl A, Farahmand F, Ghiasi MS. A novel stability and kinematics-driven trunk biomechanical model to estimate muscle and spinal forces. Medical engineering & physics. 2014;36(10):1296-304. Epub 2014/07/31. doi:

10.1016/j.medengphy.2014.07.009. PubMed PMID: 25074649.

45. Oxland TR. Fundamental biomechanics of the spine--What we have learned in the past 25 years and future directions. Journal of biomechanics. 2016;49(6):817-32. Epub 2015/12/27. doi: 10.1016/j.jbiomech.2015.10.035. PubMed PMID: 26706717.

46. Panjabi MM, White AA, 3rd. Basic biomechanics of the spine. Neurosurgery. 1980;7(1):76-93. Epub 1980/07/01. doi: 10.1227/00006123-198007000-00014. PubMed PMID: 7413053.

47. Eskandari AH, Arjmand N, Shirazi-Adl A, Farahmand F. Subject-specific 2D/3D image registration and kinematics-driven musculoskeletal model of the spine. Journal of biomechanics. 2017;57:18-26. Epub 2017/04/04. doi: 10.1016/j.jbiomech.2017.03.011. PubMed PMID: 28365064.

48. Monticone M, Ambrosini E, Cazzaniga D, Rocca B, Ferrante S. Active self-correction and task-oriented exercises reduce spinal deformity and improve quality of life in subjects with mild adolescent idiopathic scoliosis. Results of a randomised controlled trial. Eur Spine J. 2014;23(6):1204-14. Epub 2014/04/01. doi: 10.1007/s00586-014-3241-y. PubMed PMID: 24682356.

49. Kuru T, Yeldan I, Dereli EE, Ozdincler AR, Dikici F, Colak I. The efficacy of three-dimensional Schroth exercises in adolescent idiopathic scoliosis: a randomised controlled clinical trial. Clin Rehabil. 2016;30(2):181-90. Epub 2015/03/18. doi: 10.1177/0269215515575745. PubMed PMID: 25780260.

50. Weiss H, Lohschmidt K, El-Obeidi N, Verres C. Preliminary results and worst-case analysis of in patient scoliosis rehabilitation. Pediatric Rehabilitation. 1997;1(1):35-40.

51. Weiss H-R, Weiss G. Curvature progression in patients treated with scoliosis in-patient rehabilitation--a sex and age matched controlled study. Studies in health technology and informatics. 2002;91:352.

52. Weiss H-R, Weiss G, Petermann F. Incidence of curvature progression in idiopathic scoliosis patients treated with scoliosis in-patient rehabilitation (SIR): an age-and sex-matched controlled study. Pediatric rehabilitation. 2003;6(1):23-30.

53. Kim S, Heath E, Heilbrun L. Sample size determination for logistic regression on a logit-normal distribution. Stat Methods Med Res. 2017;26(3):1237-47. Epub 2015/03/04. doi: 10.1177/0962280215572407. PubMed PMID: 25744106.

S1 File_Study protocol

Click here to access/download
Supporting Information
S1_File.pdf

S2 Fig. CONSORT flow diagram

Click here to access/download
Supporting Information
S2_Fig.CONSORT_flow_diagram.doc

S3 File_Home Exercise adherence checklist

Click here to access/download

Supporting Information

S3_File.pdf

S4 File. Study data

Click here to access/download
Supporting Information
S4_File.xlsx

S5 File. SPSS output

Click here to access/download
Supporting Information
S5_SPSS_Repeated&Bonferroni.rtf

S6 File. TREND checklist

Click here to access/download
Supporting Information
S6_File_trendstatement_TREND_Checklist.pdf

Title: Does curve pattern impact on the effects of physiotherapeutic scoliosis specific exercises on Cobb angles of participants with adolescent idiopathic scoliosis: a prospective clinical trial with two years follow-up

Authors

1. Yunli Fan[1,2,3]
2. Michael KT To[2]
3. Eric HK Yeung[3]
4. Jianbin Wu[1]
5. Rong He[3]
6. Zhuoman Xu[3]
7. Ruiwen Zhang[3]
8. Guangshuo Li[3]
9. Kenneth MC Cheung[2]
10. Jason PY Cheung[2*]

[1]Department of Orthopedics, The University of Hong Kong – Shenzhen Hospital, Shenzhen, Guangdong Province, People's Republic of China

[2]Department of Orthopaedics and Traumatology, The University of Hong Kong, Hong Kong SAR, China

[3]Department of Physiotherapy, The University of Hong Kong – Shenzhen Hospital, Shenzhen, Guangdong Province, People's Republic of China

*** Corresponding author:**

Name	Cheung, Jason Pui Yin
Department	Department of Orthopaedics and Traumatology
Institution	The University of Hong Kong
Country	Hong Kong Special Administration Region, China
Email	cheungjp@hku.hk

Does curve pattern impact on the effects of physiotherapeutic scoliosis specific exercises on Cobb angles of participants with adolescent idiopathic scoliosis: a prospective clinical trial with two years follow-up

Yunli Fan[1,2,3], Michael KT To[2], Eric HK Yeung[3], Jianbin Wu[1], Rong He[3], Zhuoman Xu[3], Ruiwen Zhang[3], Guangshuo Li[3], Kenneth MC Cheung[2], Jason PY Cheung[2*]

Abstract

Background

Current clinical evidence suggests that a well-planned physiotherapeutic scoliosis specific exercise (PSSE) program is effective for scoliosis regression.

Objectives

We investigated the effect of curve patterns on Cobb angles with PSSE.

Methods

This was a non-randomized prospective clinical trial that recruited participants with adolescent idiopathic scoliosis between January and June 2017. Participants were grouped by curve pattern into major thoracic and major lumbar groups. An outpatient-based PSSE program was conducted with the following schedule of intensive exercise: ≥ 1 session of supervised PSSE per month and > 30min of home exercise 5 days/week in the first 6 months, after which exercise frequency was reduced to 1 session of supervised PSSE every three months and > 30min of home

exercise 5 days/week until 2 years after study initiation. Radiographic Cobb angle progressions were identified at the 1, 1.5 and 2-year follow-ups. A mixed model analysis of variance (ANOVA) was performed to examine the differences in Cobb angles between groups at four testing time points. The two-tailed significance level was set to 0.05.

Results

In total, 40 participants were recruited, including 22 with major thoracic curves (5 males and 17 females; mean age 13.5±1.8 years; Cobb angle 18 - 45 degrees) and 18 with major lumbar curves (7 males and 11 females; mean age 12.7±1.7 years; Cobb angle 15 - 48 degrees). Curve regressions, namely the reduction of Cobb angles between 7 to 10 degrees were noted in 9.1% of participants in the major thoracic group; reductions of 6 to 13 degrees were noted in 33.3% of participants in the major lumbar group at the 2-year follow-up. Repeated measurements revealed a significant time effect ($F_{2.2,79.8}$=4.1, *p*=0.02), but no group ($F_{2.2,79.8}$=2.3, *p*=0.1) or time × group ($F_{1,37}$=0.97, *p*=0.3) effects in reducing Cobb angles after 2 years of PSSE. A logistic regression analysis revealed that no correlation was observed between curve pattern and curve regression or stabilization (OR: 0.2, 95% CI: 0.31 - 1.1, *p*=0.068) at the 2-year follow-up.

Conclusion

This was the first study to investigate the long-term effects of PSSE in reducing Cobb angles on the basis of major curve location. No significant differences in correction were observed between major thoracic and major lumbar curves. A regression effect and no curve deterioration were noted in both groups at the 2-year follow-up.

Trial Registration: ChiCTR1900028073

*** Corresponding author:** cheungjp@hku.hk

[1]Department of Orthopedics, The University of Hong Kong – Shenzhen Hospital, Shenzhen, Guangdong Province, People's Republic of China

[2]Department of Orthopaedics and Traumatology, The University of Hong Kong, Hong Kong SAR, China

[3]Department of Physiotherapy, The University of Hong Kong – Shenzhen Hospital, Shenzhen, Guangdong Province, People's Republic of China

Introduction

Adolescent idiopathic scoliosis (AIS) is a three-dimensional spinal deformity with an unknown etiology, characterized by lateral deviation in the frontal plane, axial rotation in the horizontal plane and an abnormal sagittal curvature.[1] Surgery is typically recommended if a

spinal curve reaches 50 degrees, because such curves are associated with a continued progression risk into adulthood.[1-3] Surgical fusion causes spinal stiffness and should be avoided if possible.[3] Thus, the goal of conservative managements including bracing and exercises, is to prevent spinal deformity deterioration past the operative threshold.[4]

Options for nonsurgical management vary widely and depend on the prognostic evaluation of curve progression.[4] Curve type is an established risk factors of scoliosis progression.[5] In particular, a thoracic curve with a larger Cobb angle has a higher likehood of progression than single lumbar or thoracolumbar curves do.[2] Therefore, understanding the effect of curvature on interventions can help clinicians select appropriate treatments for patients.

Bracing is commonly prescribed for moderate scoliosis, and its corrective effect is influenced by the location of structural curves.[6, 7] In addition, scoliosis-specific exercise is a nonoperative option that is well received by patients and families.[8] Several systematic reviews and randomized controlled trials have reported the positive effects of physiotherapeutic scoliosis specific exercise (PSSE) on slowing curve progression as well as improving cosmetic and quality of life;[9-12] however, the relationship of curve location with correction effects has not been clearly discussed in these studies, in which small samples of participants have been recruited and

followed up over the short-term.[13-15] In particular, one 6-month long randomized control trial (RCT) revealed a significant correlation between thoracic curvature with an imbalanced pelvis (n=15) and the largest curves after Schroth treatment;[13] one 4-month long RCT demonstrated the greater Cobb reduction was noted in the thoracic region (n=20) after body awareness exercise.[14] Therefore, a clinical trial, in which curve pattern is controlled, is warranted to investigate the long-term effects of curve pattern and PSSE on reducing Cobb angles. This is valuable for clinical practice because the deterioration is possible after initial improvement in short-term follow-up.

According to current evidence from PSSE studies,[9, 10, 16] the Schroth is the most used PSSE approach. It adopts a specific respiratory technique, asymmetrical breathing in the diagonal direction, to achieve vertebral and rib cage derotation.[17] It utilizes muscle activation and emphasizes core muscle stabilization in a corrected posture throughout the day to change habitual postures and improve spinal alignment.[17, 18] However, the influence of curve magnitude on Schroth method outcomes is unclear. The spinal deformity profile that leads to optimal outcomes with Schroth exercise is also unclear. Thus, this study investigated the long-term therapeutic effects of Schroth exercises on major thoracic and major lumbar curves, respectively. We hypothesized that patients with lumbar curves, which have a lower reported risk of curve

progression and higher spinal segmental flexibility, benefit more from long-term PSSE treatment than do patients with major thoracic curves. The current results can provide physiotherapists with additional information to develop individualized exercises based on curve magnitude. These results can also form the basis for further RCTs evaluating the relationship between curve patterns and PSSEs.

Materials and methods

Study design

This was a non-randomized prospective clinical trial in which participants with AIS were recruited consecutively between January and June 2017 from one scoliosis clinic at the University of Hong Kong - Shenzhen Hospital (HKU - SZH), China. This study obtained ethical approval by the institutional review board of the HKU - SZH with reference number: GNSF201603 (**S1** File). Individuals in this manuscript have given written informed consent to publish these case details. The authors confirm that all ongoing and related trials for this intervention are registered in the Chinese clinical trial registry (www.chictr.org.cn, trial registration: ChiCTR1900028073) (**S1** File). Participants and legal guardians signed a written informed consent for PSSE treatment and participants were followed up for 2 years thereafter. The inclusion criteria were age 10 to 16 years; bone immaturity in terms of Risser sign was less than 5;[19] Cobb angles[20] from 10 degrees to 50 degrees; and complete attendance for all

physiotherapy sessions. Exclusion criteria were diagnoses other than AIS, disabilities or systemic illnesses that prevent participants from performing exercise, hypermobility (Beighton score[21] greater than 4) and previous treatment for AIS. Joint hypermobility (JHM) is reported to have a higher prevalence in patients with single curves than in those with double-curve scoliosis; and this may affect physiotherapy outcomes.[22] Therefore, patients with hypermobility were excluded from this study to control for systematic error introduced by JHM differences between curve patterns. Participants were categorized into two groups according to their whole spine radiographic characteristics:[23, 24] participants with 3C (major thoracic with or without a minor lumbar curve) or N3N4 curves (double, well balanced curves) formed the major thoracic group; and those with single lumbar, single thoracolumbar, or 4C curves (a major lumbar curve with a minor thoracic) comprised the major lumbar group (**S2** Fig 1).

S2 Fig 1. CONSORT flow diagram.

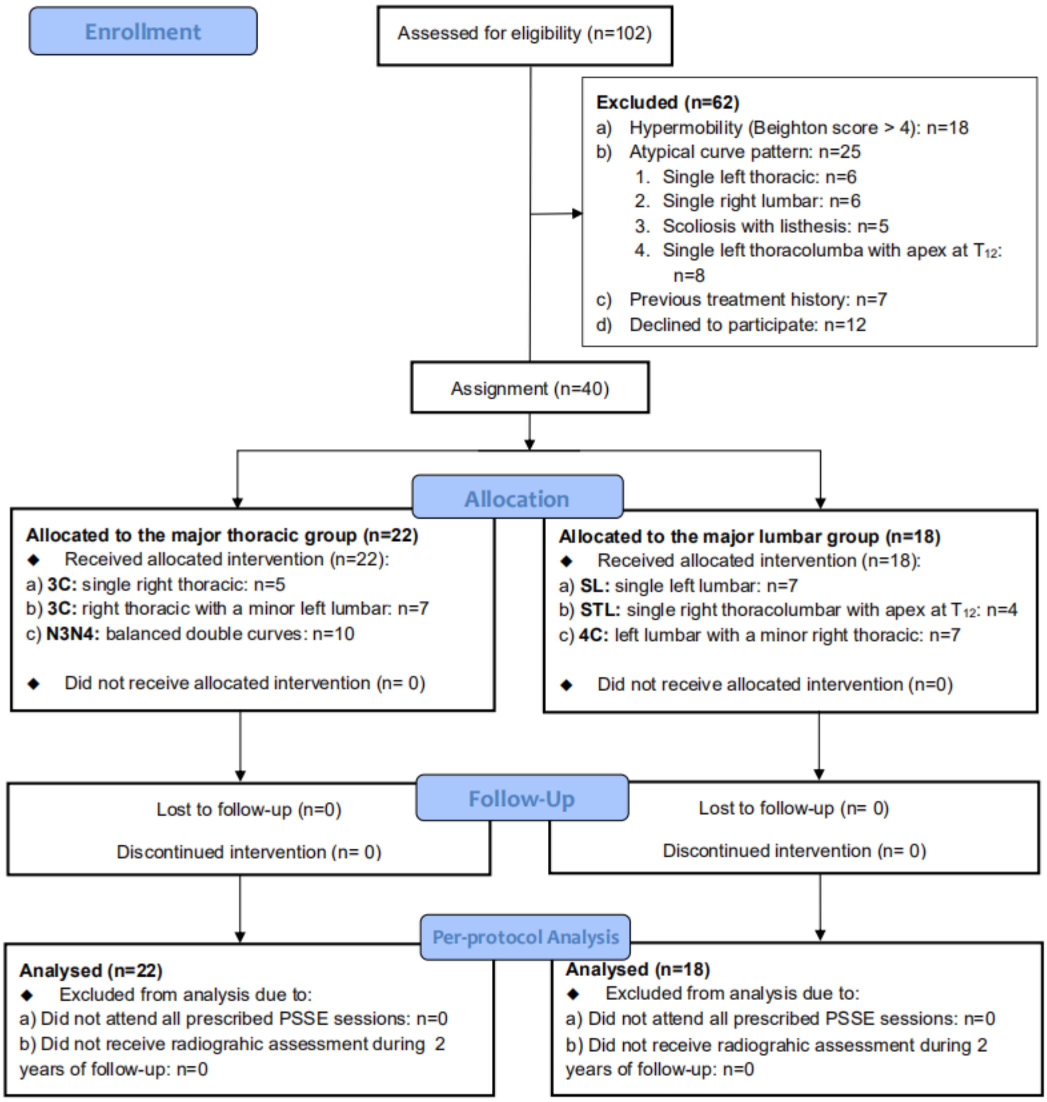

Study intervention

All participants participated in a 6-month intensive PSSE program supervised by two PSSE-Schroth certified physiotherapists (Barcelona Scoliosis Physical Therapy School [BSPTS] approach).[23] Supervised treatment during the 6 months occurred ≥1 time per month for 1 hour each time. Participants were instructed to perform >30min sessions of home exercise at least ≥5

times per week. For participants who met Scoliosis Research Society bracing criteria, Cheneau bracing was prescribed prior to the PSSE program;[25] after an adaption period (2 weeks) of wearing a brace, an initial in-brace angle[26-28] was measured by a spine surgeon who was blinded to this study, using the X-ray with a brace fitted on the participant more than two hours.[28] Bracing compliance was reported by patients on their monthly treatment adherence checklist (**S3** File). After the first 6 months of PSSE, all participants reduced their frequency of supervised PSSE to once every 3 months to review their exercises, which they performed for 2 years after completing the PSSE program. During this time, the frequency of home exercise was maintained. Attendance for supervised PSSE in an outpatient setting was recorded in the hospital's prospective database system. Participants were requested to document home exercise and bracing compliance on daily basis using a treatment adherence checklist (**S3** File), and handed it to a physiotherapist assistant who was blinded to this study every month during the first 6-month study period; then they were required to submit the checklist every 3 months thereafter. Exercise compliance was calculated in hours per week per year for analysis. The PSSE intervention consisted of 50 breaths per exercise. Short semihanging with caudal-cranial spine lengthening (Fig 2. c), corrective exercise in side-lying with shoulder counter traction (Fig 2. d), corrective exercise in sitting with pelvic pulling strategy on the side of the weak point and shoulder counter traction (Fig 2. e), corrective exercise in standing with pelvic derotation and shoulder counter traction (Fig 2. f) were

prescribed for participants in the major thoracic group. Short semihanging with caudal-cranial spine lengthening (Fig 3. c), corrective exercise in side-lying with passive correction on the lumbar prominence (Fig 3. d), corrective exercise in standing with shoulder counter traction (Fig 3. e), muscle cylinder in standing for opening lumbar concavity (Fig 3. f) and the corrective exercise in sitting with shoulder counter traction (Fig 3. g) were prescribed to participants in the major lumbar group. The difference between the two groups' exercises was that a muscle cylinder was prescribed only to participants with major lumbar curves. The muscle cylinder exercise in a kneeling position is typically prescribed to patients with major thoracic curves;[17] however, patients commonly complain that it causes knee pain. Therefore, considering the potential adverse effects during long-term follow-up, the muscle cylinder in a kneeling position was not prescribed in this study. Moreover, dynamic exercises, such as Schroth walking was not used in this study because of limited space in our center. In addition, participants were asked to record and report any discomfort, such as muscle fatigue and muscle sprain, to the on-duty physiotherapist during the study period.

Fig 2. Exercises demonstration for major right thoracic curves

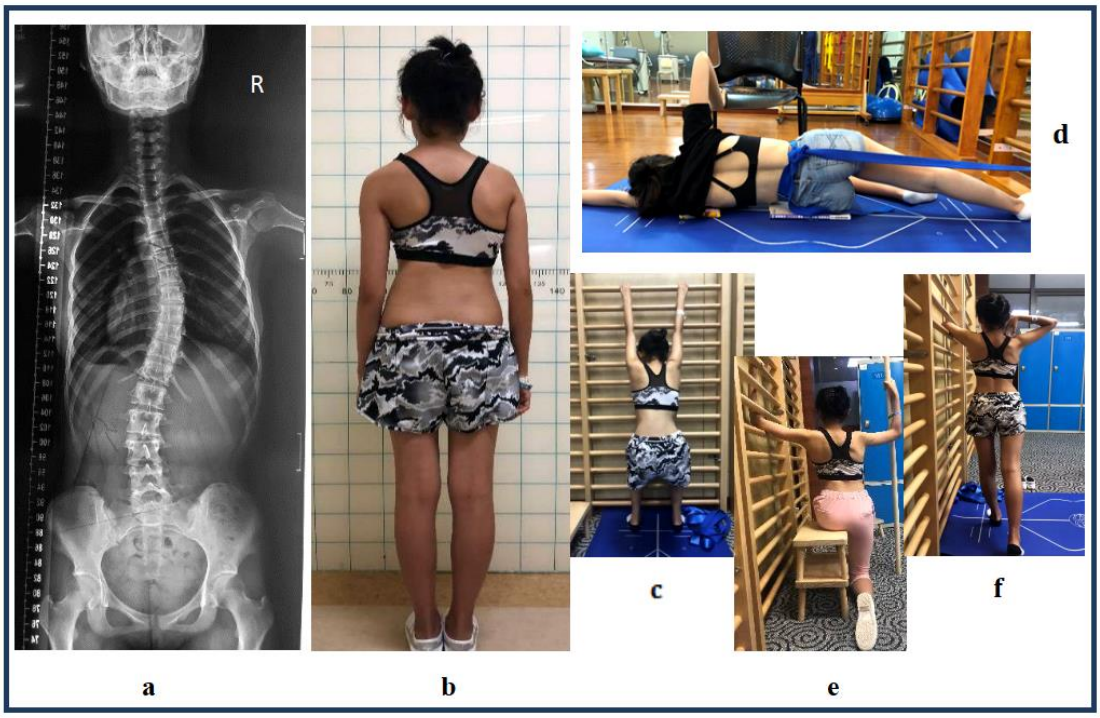

Fig 2: a: a major right thoracic (T4 to 12) with a Cobb angle of 30 degrees. **b:** posterior posture view of the participant. **c:** short semihanging with caudal-cranial spine lengthening. **d:** corrective exercise in side-lying with shoulder counter traction. **e:** corrective exercise in sitting with pelvic pulling strategy on the side of weak point and shoulder counter traction. **f:** corrective exercise in standing with pelvic derotation and shoulder counter traction.

Fig 3. Exercises demonstration for major left lumbar curves

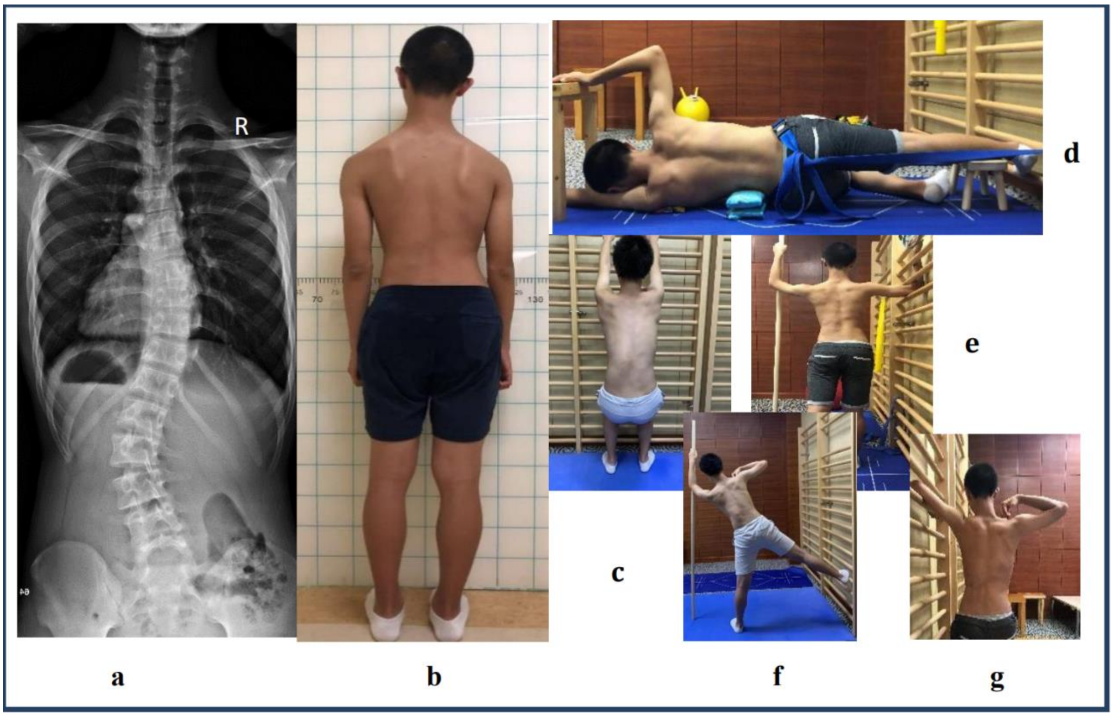

Fig 3: **a:** a major left lumbar curve (T12 to L4) with a Cobb angle of 48 degrees. **b:** posterior posture view of the participant. **c:** short semihanging with caudal-cranial spine lengthening. **d:** corrective exercise in side-lying with passive correction on the lumbar prominence. **e:** corrective exercise in standing with shoulder counter traction. **f:** muscle cylinder in standing for opening lumbar concavity. **g: c**orrective exercise in sitting with shoulder counter traction.

Outcome measurement

Participants underwent whole-spine radiography in a standing position with arms slightly abducted at the sides of body. The Cobb angles of major curves were assessed by a spine surgeon who was blinded to this study. Cobb angles were recorded in the initial assessment and every 6 months after the PSSE program until the 2-year follow-up. An in-brace Cobb angle was

measured using a radiography with the brace fitted on the participant. A percentage value of the initial in-brace correction ($IBC = \frac{Cobb\_in-brace}{Cobb\_baseline}$) was compared between groups to evaluate the bracing effect.[29] Participants remained out of their braces overnight prior to obtaining radiography during two years of follow-up. [28] Curve regression was defined as a decrease in the Cobb angle by 6 degrees or more, stabilization was defined as a <6 degrees change in Cobb angle, and deterioration was defined as a Cobb angle increase of 6 degrees or more.[2, 30]

Statistics

Sample size estimation

To obtain an effect size of 0.50, 80% power for a two-tailed test, and a correlation of 0.6 for repeated measures of Cobb angles over 4 time points, 13 participants were required for each group.[31] Considering a possible 35% dropout rate during the 2-year study period, each group were planned to have 18 participants (**S2** Fig 1).

Statistical analysis

Descriptive statistics with 95% Confidence Interval (CI) were calculated for baseline variables including demography, radiography, exercise compliance and Lonstein and Carlson Risk of progression value ($\mathrm{LCR\text{-}value} = \frac{\mathrm{Cobb} - 3 \times \mathrm{Risser}}{\mathrm{Age}}$)[32] for all participants (Table 1).[33] Changes in Cobb angles, in the form of the difference value

[$D\text{-}value = (Cobb_{\_6\text{-}month/1\text{-}/1.5\text{-}/2\text{-}year}) - (Cobb_{\_baseline})$] were measured to detect curve progression (D-value ≥ 6 degrees), stabilization (D-value < 6 degrees, or D-value > -6 degrees) and regression (D-value ≤ -6 degrees) between groups;[34] raw Cobb angles were repeatedly compared over four testing time points to detect time, group and time × group interaction effects in reducing Cobb angles. A mixed model analysis of variance (ANOVA) was performed if the normality assumptions of Cobb angles were met whereas a nonparametric Friedman-type test would be performed if normality was violated. Post hoc pairwise comparison was conducted if any significant differences were detected in either within-subjects or between-subjects comparisons. A Logistic regression was performed to detect correlations of brace treatment and curve pattern with the regression, stabilization or deterioration after 2 years of PSSE. The data were analyzed using SPSS version 20.0 (IBM, Chicago, IL). The level of significance was set to 0.05 with a two-tailed test.

Results

Participants

In total, 102 patients were screened to confirm eligibility, after which 62 patients were excluded for the following reasons: hypermobility (Beighton score > 4) was detected in 18 patients (single right thoracic: n=7, single left lumbar: n=5; right thoracic with a minor lumbar: n=6); 25 patients had reported, atypical curve patterns[35-38] (single left thoracic: n=6,[36]

single right lumbar: n=6,[37] scoliosis presented with vertebral listhesis: n=5,[38] single left thoracolumbar with apex at T_{12}: n=8[35]); 7 patients were previously treated with either bracing or exercise; 12 patients declined to participate in this study. Therefore, 40 participants were recruited and all completed follow-up (**S2** Fig 1). The major thoracic group had 22 participants (5 males and 17 females). Of them, 12 had 3C curves (single right thoracic: n=5; right thoracic with a minor left lumbar: n=7) and 10 had N3N4 curves (balanced right thoracic with a left lumbar). The major lumbar group had 18 participants (7 males and 11 females). Of them, 7 had single left lumbar curves, 4 had right thoracolumbar curves with apex at T_{12} and 7 had 4C curves (left lumbar with a minor right thoracic). The mean age of participants in the major thoracic and major lumbar groups was 13.5±1.8 years and 12.7±1.7 years, respectively (Table 1). At baseline, the two groups did not differ statistically in age, sex, Risser sign (0-2 / 3-4), proportion of sub-curvature types, LCR-value, or initial Cobb angle of the major curve (Table 1). Ten participants with brace treatment who all started Cheneau orthosis[39] before commencing the PSSE and achieved 37% of initial in-brace correction[27] in each group (Table 1). In particular, 5 of them were wearing a night-time brace only (≤ 8 hours/day) whereas 5 were wearing a full-time brace (≥21 hours/day) (Table 1). Two participants in the major lumbar group refused brace treatment due to cosmetic concerns, which introduced an imbalance in bracing between groups. Thus, bracing was set as a covariate with the repeated analysis to evaluate effects of PSSE in reducing

Cobb angles.

Table 1. Baseline characteristics of participants

	**Major thoracic group** (95% confidence interval)	**Major Lumbar group** (95% confidence interval)
**Age** (years: Mean ± SD)	13.5 ± 1.8 (12.8 - 14.6)	12.7 ± 1.7 (12.6 - 13.7)
**Sex**		
Female	17 (77.3%)	11 (61.1%)
Male	5 (22.7%)	7 (38.9%)
**Risser sign**		
0-2 / 3-4	9 / 13	9 / 9
**Initial Cobb Angle** (min-max)	18 - 45	15 - 48
(degrees: Mean ± SD)	28.5 ± 8.4 (24.5 - 32.5)	26.8 ± 10.1 (22.4 - 31.2)
**Curve pattern**		
3C / SL or STL	12	11
N3N4 / 4C	10	7
**Braced** (yes)	7	3*

In-brace hours (hours/day)	13.6 ± 6.9	16.7 ± 7.5*
full-time brace (≥ 21hr/day): n	3	2
night-time brace (≤ 8hr/day): n	4	1*
**Initial in-brace Cobb angle**		
(degrees: Mean ± SD)	23 ± 4.4	26 ± 6
**Initial in-brace correction rate (%)**	37	37
**Initial progression risk**		
Lonstein and Carlson Risk of progression	44 %	46%
**Exercise compliance**		
1st year: hours/week	5.5 ± 2.2	5.5 ± 2.1
2nd year: hours/week	3.0 ± 0.8[§ ¶]	4.1± 2.1

3C: A single right thoracic curve or a major right thoracic with a minor left lumbar. **SL:** A single left lumbar. **STL:** A single right thoracolumbar. N3N4: double curves but well balanced. **4C:** A major left lumbar with a minor right thoracic. * two participants refused brace treatment due to cosmetic concerns and introduced an imbalance in bracing between groups. [§] An independent *t-test* revealed a significant difference between groups. [¶] A paired *t-test* revealed a significant difference in the major thoracic group.

Exercise compliance

The two groups differed significantly in exercise compliance during the second year of follow-up (independent *t-test*, $p=0.04$) (Table 1). In addition, mean exercise compliance of the major thoracic group was significantly decreased in the second year (paired *t-test*, $p<0.01$) (Table 1); the mean exercise compliance of the major lumbar group was 5.5±2.1 hours/week in the first year and slightly decreased to 4.1±2.1 hours/week in the second year (Table 1). There were no adverse effects detected during this study period.

Intervention effects

In total, 23 participants (57.5%) with Cobb angle < 30 degrees refused to repeat radiography at the 6-month follow-up after the PSSE program because of radiation exposure concerns; therefore, no comparison of participants before and at the 6-month follow-up was performed. Nonetheless, all participants completed radiography at the 1-year, 1.5-year and 2-year follow-up, respectively. Full set of raw data is available in the **S4** File.

A per-protocol analysis was performed to analyze the raw value of Cobb angles over four testing time-points between groups. Bracing (hours/day) was set as a covariate with a mixed model repeated analysis since an imbalance of bracing was noted at study initiation. Levene's test indicated that Cobb angles at the baseline met homogeneity of variance assumption ($p=0.5$);

Mauchly test indicated that the assumption of sphericity was violated ($\chi^2 = 32.1$, df =5 , $p < 0.001$); hence, the degrees of freedom were corrected using Greenhouse-Geisser estimates of sphericity.[40] With current sample size (n=40) and by adjusting for bracing as a covariate, the repeated analysis showed a significant within-subjects time effect ($F_{2.2,79.8}=4.1$, $p=0.02$), but no group ($F_{2.2,79.8}=2.3$, $p=0.1$), bracing ($F_{2.2,79.8}=1.4$, $p=0.3$) or time × group ($F_{1,37}=0.97$, $p=0.3$) effects were detected in reducing Cobb angles after 2 years of treatment (Table 2). A post hoc pairwise comparison (within-subjects) with a Bonferroni adjustment revealed a significant reduction of Cobb angle, regardless of curve pattern, was observed on the 1.5-year (27.7±9.1 degrees vs 25.4±8.6 degrees, $p=0.015$) and the 2-year (27.7±9.1 ±9.1 degrees vs 25.4±8.2 degrees, $p<0.01$) follow-up (Table 2). A separate repeated analysis with a Bonferroni adjustment further suggested that the significant reduction of Cobb angle was observed in the major lumbar group ($F_{3,51}=6.2$, $p<0.01$), in which a reduction of Cobb angle was noted after training for one and a half years (26.8±10.1 degrees vs 23.3±9.4 degrees, $p=0.04$) with a maintained reduction after two years of follow-up (26.8±10.1 degrees vs 22.9±9.2 degrees, $p<0.01$) (Table 2).

Table 2. Per-protocol repeated measures of Cobb angles over four testing time points

Testing timepoint	Group	Mean	Std. Deviation	95% Confidence Interval	Minimum	Maximum	*P*
**Baseline**	T	28.5	8.4	24.8-32.2	18	45	-

	L	26.8	10.1	21.8-31.8	15	48	-
	Total	27.7	9.1	25.6-29.9	16	46	-
**1-year**	T	27	7.2	23.8-30.2	18	38	0.910
	L	24.7	9.4	20.0-29.4	10	42	0.476
	Total	26.0	8.2	23.8-28.0	14	40	0.107
**1.5-year**	T	27.1	7.7	23.7-30.5	10	40	1.0
	L	**23.3**[*]	9.4	18.6-28.0	10	45	**0.043**
	Total	**25.4**[ᵼ]	8.6	23.2-27.3	10	43	**0.015**
**2-year**	T	27.5	7.0	24.4-30.5	15	40	1.0
	L	**22.9**[*]	9.2	18.4-27.5	10	43	**0.009**
	Total	**25.4**[ᵼ]	8.2	23.4-27.1	13	42	**0.005**

A Bonferroni adjustment was performed with repeated measures to control familywise Type I error.

Group T: major thoracic group. **Group L:** major lumbar group. ***** significantly differ from the baseline in the major lumbar group. [ᵼ] significantly differ from the mean value at baseline for all participants.

Overall, we observed 20% with regression (n=8) and 80% with stabilization in this study, in which 65% of participants (n=26) reached bone maturity (Risser: 5) after two years of PSSE and no curve pattern was changed (Table 3). A chi-square analysis revealed that there was no difference (χ^2 =3.6, df =1, *p*=0.057) in distributions of curve regression and stabilization at the 2nd year follow-up between groups. No deterioration of the major curvature occurred in either group

(both average and individual data) at the 2-year follow-up. A logistic regression analysis further revealed that there was no correlation of bracing (OR: 0.69, 95% CI: 0.10-4.82, *p*=0.71) or curve patterns (OR: 0.2, 95% CI: 0.31 - 1.1, *p*=0.068) with curve regression or stabilization at the 2-year follow-up.

In the major thoracic group, descriptive statistics revealed that curve regressed (Cobb angles: -8.8±2.5 degrees) in 18.2% of participants and stabilized (Cobb angle: -2.9±1.6 degrees) in 81.8%, none deteriorated in the first year. Only 2 participants maintained curve regression in the second year, which resulted 9.1% of participants showed regression (Cobb angles: -8.5±2.1 degrees) (Table 3). Thus, up to 90.9% of participants stabilized (Cobb angles: -3.1±1.6 degrees), and no deterioration was detected (Table 3).

In the major lumbar group, descriptive statistics revealed that curves regressed (Cobb angles: -9.5±4.9 degrees) in 11.1%, stabilized (Cobb angles: -3.4±1.2 degrees) in 83.3% and deteriorated by 7 degrees in one subject after the first year of training. In the second year of follow-up, up to 33.3% of participants had improved Cobb angles (Cobb angles: -8.2±2.5 degrees), while 66.7% stabilized (Cobb angles: -3.2±1.6 degrees), and no deterioration was noted (Table 3). Of the participants with curve regression in the first year, one maintained the curve regression,

reducing Cobb angle of 13 degrees, and one only gained an additional degree in the second year. Moreover, one participant with a single left lumbar curve of 31 degrees (Fig. 4: a) who refused brace treatment obtained a Cobb angle of 23 degrees (Fig. 4: b) after one and a half years of PSSE. In addition, another participant with a single right thoracolumbar curve of 44 degrees who refused brace treatment (Fig. 5: a) obtained a Cobb angle of 31 degrees (Fig. 5: b) after two years of PSSE.

Table 3 Descriptive statistics of demographic and radiographic variables at the 2-year follow-up.

**The 2-year follow-up**	**Thoracic curves (n=22)**	**Lumbar curves (n=18)**
**Age** (years: Mean ± SD)	15.6 ± 1.8	14.7 ± 1.7
**Risser sign** 0-2,3-4,5 (n)	0,4,18	0, 10,8
**Cobb angle** (degrees: Mean ± SD)	27.5 ± 7.0	22.9 ± 9.2
≥ 30 degrees: n (%): min – max	8 (36.4%): 31° - 40°	5 (27.8%): 30° - 43°
<30 degrees: n (%): min – max	14 (63.6%):15°- 28°	13 (72.2%): 10° -27°
a) **Regressed**: n (%): min to max of D-value	2 (9.1%): -6° to -10°	6 (33.3%): -6° to -13°
b) **Stabilized**: n (%): min to max of D-value	22 (90.9%): 5° to -5°	12 (66.7%): 3° to -5°
c) **Progressed**: n (%): min to max of D-value	0	0
**Curve pattern:** 3C / SL or STL (n)	12	11
N3N4 / 4C (n)	10	7

Fig 4. Radiographic assessment (posterior anterior view) of one participant with a single left lumbar scoliosis who refused brace treatment for cosmetic reasons.

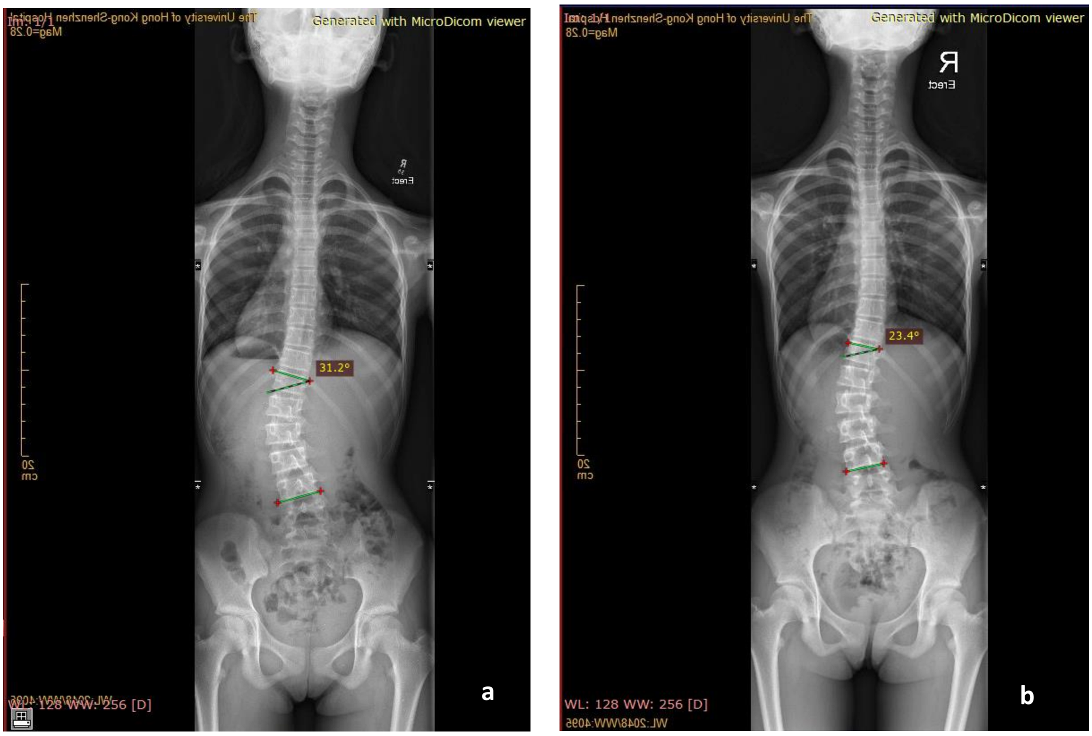

a: an initial Cobb angle of 31 degrees from T12 to L4. **b:** a Cobb angle of 23 degrees from T12 to L4 at the 1.5-year of follow-up.

Fig 5. Radiographic assessment (posterior anterior view) of one participant with a single right thoracolumbar scoliosis who refused brace treatment for cosmetic reasons.

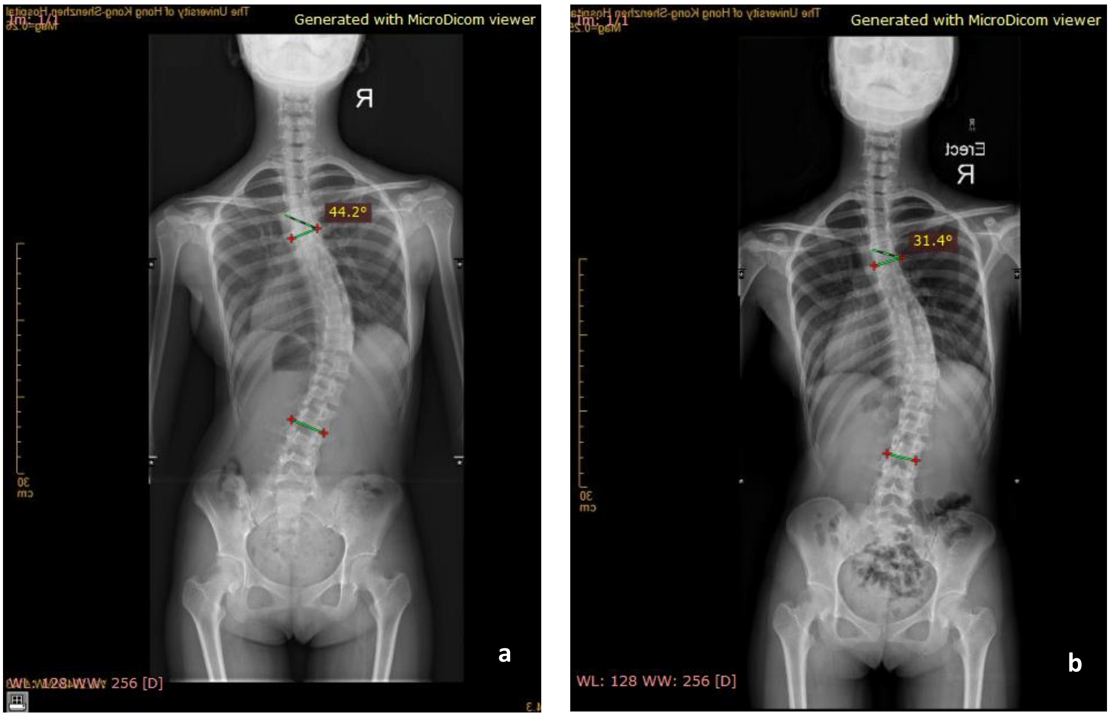

a: an initial Cobb angle of 44 degrees from T5 to L2. **b:** a Cobb angle of 31 degrees from T5 to L2 at the two-year follow-up.

Discussion

This was the first study to investigate the long-term outcomes of patients with different AIS curve patterns undergoing PSSE treatment. The findings of this study indicate no significant difference in Cobb angle changes between groups after regular PSSE for two years, regardless of the curve pattern. This implies that the curve pattern does not significantly impact the effects of PSSE in participants with AIS. A previous 6-month long study suggested that the curve pattern, a thoracic curvature with an imbalanced pelvis (n=15), is significantly correlated with the largest sum of curves after Schroth exercise, and that study further explained this might be due to a higher

risk of progression in thoracic scoliosis.[13] However, with a short-term follow-up and limited sample size, that study could not draw any firm conclusions on the relationship of curve pattern and Schroth exercise in treating AIS. Our results extend this discussion with a longer follow-up period, and curve patterns were controlled before PSSE commenced. In this study, no curve progression was observed in either group, suggesting that a well-planned PSSE program can prevent scoliosis deterioration regardless of curve patterns. This result is consistent with previous findings that PSSE reduces curve progression in patients with mild curve.[41-43] Compared with those studies, we observed regression effects in larger curves and with a longer follow-up period. Moreover, participants in our study presented a higher progression risk (Table 1: LCR-value: 44% vs 46%) at the study initiation; additionally, participants with brace treatment and all received the same initial in-brace correction of 37% improvement. The lack of deterioration may be attributable to the effects of both bracing and PSSE; however, because comparable initial in-brace correction explained the effects of bracing, the effects from the PSSE could be highlighted in our study. Therefore, our results revealed the effectiveness of PSSE programs in curve stabilization and regression of scoliosis for patients with mild to moderate scoliosis. However, a risk of overtreatment exists because patients with only mild curves may not experience progression. Therefore, the burden of treatment should be avoided as much as possible through periodical assessments.[13] Optimal time points for assessment require future study.

After two years of follow-up, no clinical deterioration was detected in either group, indicating a promising effect of PSSE on the prevention of scoliosis progression. Moreover, the regression effect accompanied by a notable decease of progression risk after 2 years. In particular, 65% of participants reached skeletal maturity (Risser 5) and 68% of participants presented <30 degrees of Cobb angle after two years of treatment (Table 3). This is promising because of curve less than 30 degrees are unlikely to progress after skeletal maturity.[2] In particular, a decrease of exercise compliance at the second year may have led to less of a regression effect at the 2-year follow-up in the major thoracic group; a significant time effect in reduction of Cobb angles was only noted for participants with the major lumbar curves; moreover, a difference was observed as early as one and a half years after training, and reductions were maintained at the end of the study period. This might be parallel with our original study hypothesis that major lumbar curves benefited more from long-term exercise in terms of having a lower progression risk; however, this should be interpreted with caution because progression values, in terms of LCR-value was 44% with thoracic curves and 46% with lumbar curves, were comparable between groups at the study initiation. Hence, this observed promising result with lumbar curves might be explained by the assumed more important flexibility of the lumbar spine than the thoracic spine, confounded with a better exercise compliance and its associated effects of good core lumbar muscle stabilization.

The superior flexibility of the lumbar spine compared to the thoracic spine has been proven in biomechanical studies[44-46]. The lumbar spine is mainly stabilized by core muscles that are flexible, stretchable and respond to external forces very well, whereas the stabilization mechanism in the thoracic spine is reliant on the rib cage, which contributes to rigidity and stability[45-47]. Additionally, according to the general principles of the Schroth method, corrective strategies including side shift and axial elongation are done in both thoracic and lumbar regions, whereas spiral breathing is specifically adopted in the thoracic region[17]. Spiral breathing is difficult to learn and hard to control for most participants with AIS due to the requirement of breathing diagonally and asymmetrically not only in the frontal plane but also in the horizontal plane. Therefore, it is relatively easier for participants to correct the lumbar curvature via pelvic positioning, lengthening the curve concavity and stabilizing it in the proper position. In addition, stabilization effects from core muscles are crucial for maintaining correction in lumbar spines[17, 47]. Therefore, the lumbar curvature may respond better to the three-dimensional correction forces from a musculoskeletal functional perspective; however, real-time feedback of the spine during different corrective exercises requires future study.

In our study, significant differences between groups in exercise compliance were noted in the second year. Higher compliance in the major lumbar curve might contribute to the larger

Cobb angle reduction. This extends the results of some previous long-term studies of mild scoliosis that reported the only exercise intensity without reporting compliance.[16, 48] These previous studies have not addressed the relationship of compliance and treatment effects, which is of clinical value to define exercise protocols for subjects with PSSE treatment. In addition, our study revealed that an outpatient home-based PSSE program is a feasible protocol with reasonable compliance. The parents were asked to send monthly videos documenting the exercises performed to the Schroth therapist, which provided a reasonable variation control with respect to exercise quality and compliance. The frequency of such home-based programs was collected via a treatment adherence checklist, and exercise compliance was interpreted by how long participants spent on the PSSE program. This study calculated the supervised PSSE sessions and home exercise sessions in a sum number, in the form of the total hours per week. Hence, we were unable to analyze specifically whether supervised PSSE is superior to home exercises in curve correction despite previous accounts of such a relationship[49-52]. Besides, the bracing compliance was self-reported, and this might introduce recall effects, which can be improved if using a pressure sensor to monitor bracing hours. Nonetheless, only 10 participants wore a brace with 5 of which were wearing nighttime brace only, hence, bracing would not cause a big distraction in our study.

This study had a few inevitable limitations in addition to the small sample size. First, no

untreated control group was included for comparison of intervention effects. However, because of the proven benefits of PSSE on AIS [11-13], it was unethical to leave participants without treatment for as long as 2 years. Thus, the benefit observed may have been influenced by natural recovery of the condition. However, the progression risk at study initiation was evaluated with approximately 45% of progression rate, and with a moderate Cobb angles of 29 degrees with major thoracic and 27 degrees with major lumbar curves, hence, the natural recovery was unlikely in our study. Second, we only enrolled participants representing 5 different types from 8 in the BSPTS classification.[24] The BSPTS concept categorizes scoliosis into 3C (A1, A2 and A3), 4C (B1 and B2), N3N4, single lumbar/thoracolumbar and double thoracic.[23, 24] For participants having a double curve type such as N3N4 or 4C, the minor curve will cause limits to overstretching and twisting of the concavity from the major curve that weaken the effects of correction in the major curve. However, the proportion of curves pattern in this study was similar (Table 1: 40.9% vs 44.4%) between the two groups, which could not cause large variations in the data. Besides, there were only 6 participants with single right thoracic curve and no double upper thoracic curves were presented in this study, therefore, it would decrease the observed power if we extra analysed the single thoracic curves independently. However, a comparison of the effects of PSSE for all eight curve types separately remains valuable for further study. Third, we were unable to blind the participants or physiotherapists due to the nature of PSSE studies, the exercise was prescribed and

performed based on the understanding of the curve pattern by both therapists and patients, which may introduce methodological bias but also limited systematic errors such as learning effects. Performing exercise precisely is crucial to reach and maintain treatment effect, therefore, blinding of subjects and therapists was commonly sacrificed in most PSSE studies.[10, 12] However, the frequency and dosage of both supervised and home programs were strictly fixed, and we blinded the result assessor which controlled bias in the statistical analysis. Finally, we assumed the lumbar spine responded more to PSSE because of its lower progression rate and with proven better segmental spinal flexibility than the thoracic spine. However, because of radiation exposure concerns, no bending radiographic assessment conducted to quantify spinal flexibility before the PSSE program. Thus, this study could not interpret the relationship between spinal flexibility and PSSE. Nonetheless, this study excluded participants with hypermobility that may cause systematic error during exercise. This controlled for potential heterogeneity when the spine started to respond to exercise. However, future study should address the relationship of spinal flexibility with PSSE, to define the correlation of curve location, segmental flexibility and PSSE in treating AIS. In addition to repeated measures of Cobb angles (raw value), we adopted a logistic regression analysis to investigate the correlation of curve pattern with scoliosis progression after 2 years of PSSE using a clinical significance threshold (D-value of a change in Cobb angle: ≥ 6 degrees). This showed a value of clinical practice, but the analysis was underpowered regarding the small

sample size.[53] Thus, a study with a bigger sample size is required to detect the correlation of curve pattern with scoliosis progression after PSSE treatment.

Conclusion

PSSE treatment outcomes did not differ by thoracic or lumbar curve type. In general, PSSE with reasonable exercise compliance has positive effects on preventing scoliosis progression. However, further studies are necessary to address the correlation between spinal flexibility and the correction effects of PSSE when different curve magnitudes, curve types and levels are involved. The cosmetic and quality of life outcomes of PSSE should also be studied. This study formed the base for future larger scale studies and randomized trials.

Supporting information

S1 File. Study protocol. (PDF)

S2 Fig. CONSORT flow diagram. (DOCX)

S3 File. Treatment adherence checklist. (PDF)

S4 File. Study data. (XLSX)

Acknowledgments

This study was financially supported by the Sanming Project of Medicine (SZSM201612055) “Team of Excellence in Spinal Deformities and Spinal Degeneration Diseases” and the Seeding Project (HKUSZH201902042) of the HKU - SZH in Shenzhen, Guangdong

Province, China.

Authors' contributions

YF and JPYC designed the study. JW and HR recruited participants. YF and EHKY performed PSSE teaching. ZX, GL and RZ collected data. MKTT performed statistics. YF drafted the manuscript. JPYC supervised the study and final edited the manuscript. MKTT, KMCC and JPYC obtained funding support. All authors read and approved the submitted version of the manuscript.

References:

1. Weinstein SL, Dolan LA, Cheng JC, Danielsson A, Morcuende JA. Adolescent idiopathic scoliosis. Lancet. 2008;371(9623):1527-37. Epub 2008/05/06. doi: 10.1016/s0140-6736(08)60658-3. PubMed PMID: 18456103.
2. Weinstein SL. The Natural History of Adolescent Idiopathic Scoliosis. J Pediatr Orthop. 2019;39(Issue 6, Supplement 1 Suppl 1):S44-s6. Epub 2019/06/07. doi: 10.1097/bpo.0000000000001350. PubMed PMID: 31169647.
3. Coe JD, Arlet V, Donaldson W, Berven S, Hanson DS, Mudiyam R, et al. Complications in spinal fusion for adolescent idiopathic scoliosis in the new millennium. A report of the Scoliosis Research Society Morbidity and Mortality Committee. Spine (Phila Pa 1976). 2006;31(3):345-9.
4. Korbel K, Kozinoga M, Stoliński Ł, Kotwicki T. Scoliosis Research Society (SRS) criteria and Society of Scoliosis Orthopaedic and Rehabilitation Treatment (SOSORT) 2008 guidelines in non-operative treatment of idiopathic scoliosis. Polish orthopedics and traumatology. 2014;79:118-22.
5. Dolan LA, Weinstein SL, Abel MF, Bosch PP, Dobbs MB, Farber TO, et al. Bracing in Adolescent Idiopathic Scoliosis Trial (BrAIST): Development and Validation of a Prognostic Model in Untreated Adolescent Idiopathic Scoliosis Using the Simplified Skeletal Maturity System. Spine Deform. 2019;7(6):890-8. e4.
6. Almansour H, Pepke W, Bruckner T, Diebo BG, Akbar M. Three-Dimensional Analysis of Initial Brace Correction in the Setting of Adolescent Idiopathic Scoliosis. Journal of clinical medicine. 2019;8(11):1804.
7. Thompson RM, Hubbard EW, Jo CH, Virostek D, Karol LA. Brace Success Is Related to Curve Type

in Patients with Adolescent Idiopathic Scoliosis. J Bone Joint Surg Am. 2017;99(11):923-8. Epub 2017/06/08. doi: 10.2106/jbjs.16.01050. PubMed PMID: 28590377.

8. Negrini S, Carabalona R. Social acceptability of treatments for adolescent idiopathic scoliosis: a cross-sectional study. Scoliosis. 2006;1:14. Epub 2006/08/26. doi: 10.1186/1748-7161-1-14. PubMed PMID: 16930488; PubMed Central PMCID: PMCPMC1560163.

9. Burger M, Coetzee W, du Plessis LZ, Geldenhuys L, Joubert F, Myburgh E, et al. The effectiveness of Schroth exercises in adolescents with idiopathic scoliosis: A systematic review and meta-analysis. S Afr J Physiother. 2019;75(1):904. Epub 2019/06/18. doi: 10.4102/sajp.v75i1.904. PubMed PMID: 31206094; PubMed Central PMCID: PMCPMC6556933.

10. Day JM, Fletcher J, Coghlan M, Ravine T. Review of scoliosis-specific exercise methods used to correct adolescent idiopathic scoliosis. Arch Physiother. 2019;9:8. Epub 2019/08/30. doi: 10.1186/s40945-019-0060-9. PubMed PMID: 31463082; PubMed Central PMCID: PMCPMC6708126.

11. Fan Y, Ren Q, To MKT, Cheung JPY. Effectiveness of scoliosis-specific exercises for alleviating adolescent idiopathic scoliosis: a systematic review. BMC Musculoskelet Disord. 2020;21(1):495. Epub 2020/07/29. doi: 10.1186/s12891-020-03517-6. PubMed PMID: 32718339; PubMed Central PMCID: PMCPMC7385878.

12. Thompson JY, Williamson EM, Williams MA, Heine PJ, Lamb SE. Effectiveness of scoliosis-specific exercises for adolescent idiopathic scoliosis compared with other non-surgical interventions: a systematic review and meta-analysis. Physiotherapy. 2019;105(2):214-34. Epub 2019/03/03. doi: 10.1016/j.physio.2018.10.004. PubMed PMID: 30824243.

13. Schreiber S, Parent EC, Khodayari Moez E, Hedden DM, Hill DL, Moreau M, et al. Schroth Physiotherapeutic Scoliosis-Specific Exercises Added to the Standard of Care Lead to Better Cobb Angle Outcomes in Adolescents with Idiopathic Scoliosis - an Assessor and Statistician Blinded Randomized Controlled Trial. PLoS One. 2016;11(12):e0168746. Epub 2016/12/30. doi: 10.1371/journal.pone.0168746. PubMed PMID: 28033399; PubMed Central PMCID: PMCPMC5198985.

14. Yagci G, Ayhan C, Yakut Y. Effectiveness of basic body awareness therapy in adolescents with idiopathic scoliosis: A randomized controlled study1. J Back Musculoskelet Rehabil. 2018;31(4):693-701. Epub 2018/04/10. doi: 10.3233/bmr-170868. PubMed PMID: 29630516.

15. Yagci G, Yakut Y. Core stabilization exercises versus scoliosis-specific exercises in moderate idiopathic scoliosis treatment. Prosthet Orthot Int. 2019;43(3):301-8. Epub 2019/01/11. doi: 10.1177/0309364618820144. PubMed PMID: 30628526.

16. Liu D, Huang S, Yu X, Yang J, Xuan X, Yang J, et al. Effects of Specific Exercise Therapy on Adolescent Patients with Idiopathic Scoliosis: A Prospective Controlled Cohort Study. Spine (Phila Pa 1976). 2020. Epub 2020/02/26. doi: 10.1097/brs.0000000000003451. PubMed PMID: 32097267.

17. Lehnert-Schroth C. [Schroth's three dimensional treatment of scoliosis]. ZFA (Stuttgart). 1979;55(34):1969-76. Epub 1979/12/10. PubMed PMID: 547573.

18. Lehnert-Schroth C. [The "four-curved" scoliosis. The effect on the additional lumbosacral spinal curve by the three dimensional Schroth's scoliosis treatment]. ZFA (Stuttgart). 1981;57(32):2227-31. Epub 1981/11/20. PubMed PMID: 7314859.
19. Little DG, Sussman MD. The Risser sign: a critical analysis. Journal of Pediatric Orthopaedics. 1994;14(5):569-75.
20. Cobb JR. Scoliosis—Quo Vadis? JBJS. 1958;40(3):507-10.
21. Smits-Engelsman B, Klerks M, Kirby A. Beighton score: a valid measure for generalized hypermobility in children. J Pediatr. 2011;158(1):119-23, 23.e1-4. Epub 2010/09/21. doi: 10.1016/j.jpeds.2010.07.021. PubMed PMID: 20850761.
22. Czaprowski D, Kotwicki T, Pawłowska P, Stoliński L. Joint hypermobility in children with idiopathic scoliosis: SOSORT award 2011 winner. Scoliosis. 2011;6(1):22.
23. Berdishevsky H, Lebel VA, Bettany-Saltikov J, Rigo M, Lebel A, Hennes A, et al. Physiotherapy scoliosis-specific exercises–a comprehensive review of seven major schools. Scoliosis Spinal Disord. 2016;11(1):20.
24. Rigo MD, Villagrasa M, Gallo D. A specific scoliosis classification correlating with brace treatment: description and reliability. Scoliosis. 2010;5(1):1.
25. Negrini S, Atanasio S, Fusco C, Zaina F. Effectiveness of complete conservative treatment for adolescent idiopathic scoliosis (bracing and exercises) based on SOSORT management criteria: results according to the SRS criteria for bracing studies-SOSORT Award 2009 Winner. Scoliosis. 2009;4(1):19.
26. Almansour H, Pepke W, Bruckner T, Diebo BG, Akbar M. Three-Dimensional Analysis of Initial Brace Correction in the Setting of Adolescent Idiopathic Scoliosis. J Clin Med. 2019;8(11). Epub 2019/10/31. doi: 10.3390/jcm8111804. PubMed PMID: 31661811; PubMed Central PMCID: PMCPMC6912396.
27. Dolan LA, Weinstein SL, Abel MF, Bosch PP, Dobbs MB, Farber TO, et al. Bracing in Adolescent Idiopathic Scoliosis Trial (BrAIST): Development and Validation of a Prognostic Model in Untreated Adolescent Idiopathic Scoliosis Using the Simplified Skeletal Maturity System. Spine Deform. 2019;7(6):890-8.e4. Epub 2019/11/17. doi: 10.1016/j.jspd.2019.01.011. PubMed PMID: 31731999; PubMed Central PMCID: PMCPMC6939758.
28. Li M, Wong M, Luk KD, Wong KW, Cheung KM. Time-dependent response of scoliotic curvature to orthotic intervention: when should a radiograph be obtained after putting on or taking off a spinal orthosis? Spine. 2014;39(17):1408-16.
29. Chan SL, Cheung KM, Luk KD, Wong KW, Wong MS. A correlation study between in-brace correction, compliance to spinal orthosis and health-related quality of life of patients with Adolescent Idiopathic Scoliosis. Scoliosis. 2014;9(1):1-. doi: 10.1186/1748-7161-9-1. PubMed PMID: 24559234.
30. Malfair D, Flemming AK, Dvorak MF, Munk PL, Vertinsky AT, Heran MK, et al. Radiographic evaluation of scoliosis. American Journal of Roentgenology. 2010;194(3_supplement):S8-S22.
31. Fitzmaurice GM, Laird NM, Ware JH. Applied longitudinal analysis: John Wiley & Sons; 2012.

32. Lonstein J, Carlson J. The prediction of curve progression in untreated idiopathic scoliosis. J Bone Jt Surg. 1984;3(2):1061-71.

33. Mishra P, Pandey CM, Singh U, Gupta A, Sahu C, Keshri A. Descriptive statistics and normality tests for statistical data. Ann Card Anaesth. 2019;22(1):67-72. doi: 10.4103/aca.ACA_157_18. PubMed PMID: 30648682.

34. Gstoettner M, Sekyra K, Walochnik N, Winter P, Wachter R, Bach CM. Inter- and intraobserver reliability assessment of the Cobb angle: manual versus digital measurement tools. Eur Spine J. 2007;16(10):1587-92. Epub 2007/06/05. doi: 10.1007/s00586-007-0401-3. PubMed PMID: 17549526.

35. Barnes P, Brody J, Jaramillo D, Akbar J, Emans J. Atypical idiopathic scoliosis: MR imaging evaluation. Radiology. 1993;186(1):247-53.

36. Davids JR, Chamberlin E, Blackhurst DW. Indications for magnetic resonance imaging in presumed adolescent idiopathic scoliosis. JBJS. 2004;86(10):2187-95.

37. Malfair D, Flemming AK, Dvorak MF, Munk PL, Vertinsky AT, Heran MK, et al. Radiographic evaluation of scoliosis: review. AJR Am J Roentgenol. 2010;194(3 Suppl):S8-22. Epub 2010/03/05. doi: 10.2214/ajr.07.7145. PubMed PMID: 20173177.

38. Wang W, Zhu Z, Zhu F, Sun C, Wang Z, Sun X, et al. Different curve pattern and other radiographical characteristics in male and female patients with adolescent idiopathic scoliosis. Spine (Phila Pa 1976). 2012;37(18):1586-92. Epub 2012/03/07. doi: 10.1097/BRS.0b013e3182511d0c. PubMed PMID: 22391440.

39. De Giorgi S, Piazzolla A, Tafuri S, Borracci C, Martucci A, De Giorgi G. Chêneau brace for adolescent idiopathic scoliosis: long-term results. Can it prevent surgery? Eur Spine J. 2013;22 Suppl 6(Suppl 6):S815-S22. Epub 2013/09/17. doi: 10.1007/s00586-013-3020-1. PubMed PMID: 24043341.

40. van Ginkel JR, Kroonenberg PM. Analysis of Variance of Multiply Imputed Data. Multivariate Behav Res. 2014;49(1):78-91. doi: 10.1080/00273171.2013.855890. PubMed PMID: 24860197.

41. Weiss HR, Lohschmidt K, el-Obeidi N, Verres C. Preliminary results and worst-case analysis of in patient scoliosis rehabilitation. Pediatr Rehabil. 1997;1(1):35-40. Epub 1997/01/01. doi: 10.3109/17518429709060940. PubMed PMID: 9689236.

42. Weiss HR, Weiss G. Curvature progression in patients treated with scoliosis in-patient rehabilitation--a sex and age matched controlled study. Stud Health Technol Inform. 2002;91:352-6. Epub 2004/10/02. PubMed PMID: 15457754.

43. Weiss HR, Weiss G, Petermann F. Incidence of curvature progression in idiopathic scoliosis patients treated with scoliosis in-patient rehabilitation (SIR): an age- and sex-matched controlled study. Pediatr Rehabil. 2003;6(1):23-30. Epub 2003/05/15. doi: 10.1080/1363849031000095288. PubMed PMID: 12745892.

44. Hajihosseinali M, Arjmand N, Shirazi-Adl A, Farahmand F, Ghiasi MS. A novel stability and kinematics-driven trunk biomechanical model to estimate muscle and spinal forces. Medical engineering & physics. 2014;36(10):1296-304. Epub 2014/07/31. doi:

10.1016/j.medengphy.2014.07.009. PubMed PMID: 25074649.

45. Oxland TR. Fundamental biomechanics of the spine--What we have learned in the past 25 years and future directions. Journal of biomechanics. 2016;49(6):817-32. Epub 2015/12/27. doi: 10.1016/j.jbiomech.2015.10.035. PubMed PMID: 26706717.

46. Panjabi MM, White AA, 3rd. Basic biomechanics of the spine. Neurosurgery. 1980;7(1):76-93. Epub 1980/07/01. doi: 10.1227/00006123-198007000-00014. PubMed PMID: 7413053.

47. Eskandari AH, Arjmand N, Shirazi-Adl A, Farahmand F. Subject-specific 2D/3D image registration and kinematics-driven musculoskeletal model of the spine. Journal of biomechanics. 2017;57:18-26. Epub 2017/04/04. doi: 10.1016/j.jbiomech.2017.03.011. PubMed PMID: 28365064.

48. Monticone M, Ambrosini E, Cazzaniga D, Rocca B, Ferrante S. Active self-correction and task-oriented exercises reduce spinal deformity and improve quality of life in subjects with mild adolescent idiopathic scoliosis. Results of a randomised controlled trial. Eur Spine J. 2014;23(6):1204-14. Epub 2014/04/01. doi: 10.1007/s00586-014-3241-y. PubMed PMID: 24682356.

49. Kuru T, Yeldan I, Dereli EE, Ozdincler AR, Dikici F, Colak I. The efficacy of three-dimensional Schroth exercises in adolescent idiopathic scoliosis: a randomised controlled clinical trial. Clin Rehabil. 2016;30(2):181-90. Epub 2015/03/18. doi: 10.1177/0269215515575745. PubMed PMID: 25780260.

50. Weiss H, Lohschmidt K, El-Obeidi N, Verres C. Preliminary results and worst-case analysis of in patient scoliosis rehabilitation. Pediatric Rehabilitation. 1997;1(1):35-40.

51. Weiss H-R, Weiss G. Curvature progression in patients treated with scoliosis in-patient rehabilitation--a sex and age matched controlled study. Studies in health technology and informatics. 2002;91:352.

52. Weiss H-R, Weiss G, Petermann F. Incidence of curvature progression in idiopathic scoliosis patients treated with scoliosis in-patient rehabilitation (SIR): an age-and sex-matched controlled study. Pediatric rehabilitation. 2003;6(1):23-30.

53. Kim S, Heath E, Heilbrun L. Sample size determination for logistic regression on a logit-normal distribution. Stat Methods Med Res. 2017;26(3):1237-47. Epub 2015/03/04. doi: 10.1177/0962280215572407. PubMed PMID: 25744106.

REPLY TO REVIEWERS

Re: Ms. No. PONE-D-19-35759 - "Does Curve Pattern Impact on the Effects of Physiotherapeutic Scoliosis Specific Exercises on Cobb angle of Adolescent Idiopathic Scoliosis: A prospective clinical controlled trial with two years follow-up"

Dear Editor Alison Rushton,

The authors would like to thank you and the Reviewers for all of your time and effort devoted to the review of our aforementioned manuscript. The Reviewers' comments were indeed extremely insightful and greatly appreciated. As such, the authors would like to take this opportunity to address each and every concern the Reviewers noted in their review of our submission. In addition, where appropriate, we have also revised our manuscript accordingly. Major changes are made in light gray.

We believe that the Reviewers' comments have helped improve the quality of our manuscript. We hope that you and the Reviewers will find our revised work suitable for publication in *PLOS One.*

Editor

Recommendation#1： If applicable, we recommend that you deposit your laboratory protocols in protocols.io to enhance the reproducibility of your results. Protocols.io assigns your protocol its own identifier (DOI) so that it can be cited independently in the future.

Response： Thanks for recommendation. This is a clinical trial without laboratory protocols, this trial was online registered and the study protocol was open access on ***www.chictr.org.cn***, trial registration: ChiCTR1900028073.

Reviewer #2

Comment #1: The Title should be corrected and show DOES not DOSE

Does curve pattern impact on the effects of physiotherapeutic scoliosis specific exercises on Cobb angles of participants with adolescent idiopathic scoliosis: a prospective clinical trial with two years follow-up

Response：We apologize for the typo. The title has been corrected.

Comment #2: Specify in the abstract that the study is a non-randomized prospective trial.

Response：Study design is specified in **line 17**.

Comment #3: In the abstract and the text line 203 The analysis should be announced as a mixed model anova (2 group by N time). Avoid stating 1 way as you have two factors. Group is a between groups factor and time is a within group factor.

Response：Analysis method was renamed accordingly, please see **line 24-25**.

Comment #4: Your sentence on line 231 to 235 is unclear. There is an important difference between groups in the numbers of patients with brace. Specify that in the text.

Response：This was specified on **lines 241 to 244.**

Comments #5: Also did all braced patients have a Cheneau? the sentence suggests you only talk about those wearing a Cheneau brace but does not clearly state whether other types were used. Specify at what point correction was measured. Do you mean out of brace at the end of the study or in-brace and if yes then when was in-brace correction measured.

Response: 1. All braced subjects were wearing Cheneau brace, sorry for this confusion, clarification was made on **lines 237 to 239**.

2. The in-brace correction was the initial in-brace correction (a percentage value of Cobb reduction during an X-ray with the brace fitted on the participant more than two hours), which was measured after a brace adaption period (two weeks). Please see **lines 136 to 138 and lines 188 to 190**.

3. Regarding the Cobb angle at the end of the study, it was measured without brace and participants were required off-bracing at night prior to X-ray measurement in the next day. Please see **lines 190 to 191**.

Comment #6: Abstract. The following sentence in the abstract could be replaced by results of your regression. Your objective was focused on effect of curve type. This sentence does not add to this objective: In addition, 65% of 37 participants (n=26) reached skeletal maturity (Risser sign = 5) and achieved significant reductions 38 in progression risk at the 2-year follow-up (Lonstein

and Carlson Risk of progression: 0.45 ± 0.32 39 vs. 0.13 ± 0.14, p <0.001).
Obviously, patients reaching maturity would have significantly reduced progression risk. If you keep this idea in the abstract, please specify what this means with regards to their curve types or having done exercises.

Response: Fantastic suggestion. We re-structured the result of abstract and emphasized result of correlation of curve pattern with curve regression. Please **see lines 36 to 38.**

Comment #7: In the consort flow chart please specify the number of cases that were excluded because they did not attend all the prescribed exercises sessions. (specify none did if that is the case)

Response: All participants completed prescribed exercise sessions. The clarification was further edited in **S2 Fig. CONSORT flow diagram** and **line 127.**

Comment #8: Figure 2 f. The picture is stretched too wide. Please present in the original aspect ratio.

Response: The picture (Fig.2) is updated with original aspect ratio. Please check **line 168.**

Comment #9: Page 13 line 203. Specify how you calculated D-value. Did you do for example 6 months minus baseline or basline minus 6 months. Specifying will help interpret mean d-values as indicating improved curves or deterioration.

Response: Thanks for this valuable suggestion. Specifying was made on **lines 206 to 209.**

Comment #10: P14 Line 206 Please refer to comment above about using mixed model anova and not one way ANOVA.

Response: Thanks for this reminding and correction was addressed on **line 211.**

Comment #11: L218 It would be interesting to see the distribution of the hypermobility cases excluded in terms of their presenting a thoracic or lumbar dominant pattern given that you suspected a higher relation with single thoracic curves.

Response: The distribution of noted hypermobility in this study was reported on **lines 224 to 225.** We did not reproduce the same distribution as the previous study, yet we did find a few of patients presenting hypermobility regarding the Beighton score. The distribution of hypermobility in AIS can be explained better with a population level, cross-sectional study.

Comment #12: P14 Is single left lumbar atupical? Single right lumbar would be more atypical? Is this an error? Similarly, with single left thoracolumbar. Is this really atypical?

Response: This is a really good question. Proven atypical curve patterns include a single left thoracic curve, a curvature with a loss of kyphosis, a rapid progression and ≤ 6 segmental scoliosis, etc. The single right lumbar and a left thoracolumbar were reported as atypical pattern in previous studies and in which subjects were found having an underlying syrinx presented with a MRI assessment. Therefore, cases with aforementioned curve pattern were excluded to control study heterogeneity yet prescribed with either a neurological assessment or an MRI assessment before commencing any treatments. We cited the related studies, please **check lines 225 to 227 and references 35 to 38.**

Comment #13: L229 to 231. I continue to disagree that you should compare baseline characteristics between groups statistically. Instead you should report what appears to be clinically important differences and maybe announced that this would be justification to explore these characteristics as covariables later. The study did not plan its sample size to detect such differences. The key is to explore if they play a role in confounding the results later.

Response: Sure, we do agree that defining confounding factors is way more important than comparing the baseline statistically in a clinical trial, Thus, baseline characteristics were reported by descriptive statistics in this updated manuscript. Please check **Table 1.** In addition, bracing was set as a covariate with repeated measures to evaluate effects of PSSE in reducing Cobb angles.

Comment #14: L230 Please spell out what LCR stands for.

Response: Lonstein Carlson Risk of progression (LCR-value) was firstly explained on the **line 205,** then the abbreviated term, LCR-value was adopted throughout manuscript.

Comment #15: L231. The imbalance in bracing may not have reached significance but it should be pointed out.

Response: Sure indeed, this was emphasized on the **lines 241 to 244.**

Comments #16: L237 Table 1. If you keep the p values add which test, they came from in the table legend on in the column with the p-values. This would help the informed reader determine if your test likely had enough power.

Response: We used descriptive statistics for baseline characteristics in this updated manuscript, thus no *p* value was reported **in the table 1**, but we adopted t-test and paired t-test to compare the exercise compliance of each year, which would help us on discussing how did exercise compliance affect the outcome. Thus, a table legend was made accordingly on the **lines 250 to 252.**

Comment #17: L237 Here or in the methods state when the in-brace Cobb angle was measured.

Response: The timepoint of measuring in-brace Cobb angle was stated on the **lines 136 to 138.**

Comment #18: L237 Refer to 1st year and 2nd year in the exercise compliance please.

Response: Done. Please check the **Table 1**.

Comment #19: L246 Since in the analysis section you left 2 choices for testing this different please report what test was used to compare the groups here with the p-value.

Response: This is a subgroup analysis to detect any changes of exercise compliance between groups on the 1st and 2nd year follow-up. Hence, an independent *t-test*, and paired *t-test* were used to compare the exercise compliance between and within groups. Please check **lines 256 to 257.**

Comment #20: L248 Similarly. Please report the test used from comparisons within the thoracic group.

Response: This is a subgroup analysis to detect any changes of exercise compliance between groups on the 1st and 2nd year follow-up. Hence, an independent *t-test*, and paired *t-test* were used to compare the exercise compliance between and within groups. Please check the **lines 256 to 257.**

Comment #21: L267 Table 2. Instead of group labels of 0 and 1 can you spell thoracic and lumbar or use T and L for clarity?

Response: Thanks for this suggestion. Revision was made on the **Table 2.**

Comment #22: L272 to 279. This analysis offers more information than that of the D-values. I would only report this ANOVA and possibly compared the frequencies of deterioration, stabilized or improved statistically as well. There is in fact a lot of redundancy between the analyses in table 2 and 3.

Response: Agree. Thanks for this fantastic suggestion to make our points much clearer. Hence, we withdrew the previous table 2 (repeated measures of D-value) and re-centered our primary analysis on the raw Cobb angles. Please see **lines 269 to 286**, and the updated **table 2.**

Comment #23: L289. It is less clear why this second case is highlighted. Only because of the large baseline curve? If yes specify that this case it highlighted because or the large curve. I was wondering if it was because it was the second case having refused the brace. Please clarify.

Response: The second case refused the brace. The clarification was made on the **lines 320 - 321**.

Comment #24: L309 To 311. It is redundant to repeat the baseline mean in each of the parentheses. Clarify which test produced the p-values. This set of results is surprising as it would suggest that the interaction should be significant the pattern of differences between timepoints was not the same between the two groups, yet you report that the interactions was not significant. Since the interaction was not significant you should not put emphasis on the different in changes over time between the groups.

Response: Thanks for this comment, the redundancy was deleted. Please check the re-structured paragraphs on **lines 294 to 321**, using descriptive statistics to illustrate the trend of scoliosis regression and stabilization in each group. The repeated measures with Bonferroni adjustment showed a significant time-effect in reducing Cobb angles (raw value) over four testing times. Additionally, post hoc analysis showed the significant reductions of Cobb were occurred on the 1.5- and 2-year follow-up, respectively. Therefore, we used descriptive statistics, additionally to illustrate how many participants in which group showed clinical curve regression. In case of we withdrew the repeated measures of D-value of Cobb angles, hence the previous redundancy was completely deleted.

Comment #25: L280 to 287. 312. To 317 Ideally the frequency results of the two groups would be reported together in terms of whether the distributions of improved, stable or deteriorated were similar or not between groups. Specify which test was used to test for those differences in the distributions.

Response: Fantastic suggestion, please see the re-structured paragraphs on **lines 294 to 321.** The distribution of scoliosis progression, stabilization and regression was analysed using Chi-square analysis. Please see **lines 296 to 298**.

Comment #26: L321 322. Focusing on a reduction in the progression risk is possibly misusing the formula of Lonstein and Carlson since it uses age and Risser so heavily and you did not report the formula in your paper. The change in angles would not play a clear role and this may mislead readers to think the reduction is due to the PSSE when maturity plays a big role. Their study possibly did not examine patients starting this late to really use the equation at this stage of treatment. If you keep this analysis, please report the full formula and add a discussion related to maturation in the discussion in relation to this reduced risk.

Response: Fantastic point, thanks for pointing it out, we realized that comparing LCR-value after two years follow-up was inappropriate. Therefore, we withdrew the comparison of LCR-value at the 2-year follow-up and replaced it to a description of how much participants reached skeletal maturity (Risser 5) and presented with a curve less than 30 degrees. Please see **lines 294 to 295 and Table 3.**

Comment #27: L328. Please add units for Age, the Cobb angle mean lines

Response: Done, Thanks for this suggestion. Please see **line 323 (Table 3)**.

Comment #28: L323 Your study likely did not have the power to examine so many variables in the logistic regression. Since your focus is on the curve type. Can you report the OR for the association between curve type and the 2 year curve outcomes? Once you enter multiple categorical predictors there really isn't a lot of observations with the regression outcome to determine prediction estimates.

Response: Nice point, thanks for pointing it out, we now only enrolled curve pattern and bracing (since we enrolled bracing into repeated measures) into the logistic regression model to further illustrate the effects of bracing and curve pattern in scoliosis regression with PSSE. Please see **lines 299 to 302.** However, this logistic regression was underpowered since this study was not originally designed for a regression analysis, thus, we acknowledged this limitation on the **lines 449 to 454**.

Comment #29: L351 to 353. You cannot claim you demonstrated Superiority of PSSE. You did not compare PSSE to an alternate intervention. You showed all patients receiving PSSE have avoided curve progression but not superiority to other treatments or to natural history (unless you refer to historical control and predictions given the progression risk.)

Response: Agree, we replaced "superiority of PSSE" by "effectiveness of PSSE" in preventing scoliosis progression. Please see **lines 358 to 359.**

Comment #30: L359 to 361. Again, I disagree with emphasizing the change in progression risk. I do not believe the formula can be used this way. Further once again you omit to emphasize that much of this reduce regression risk is due to the change in age and Risser. It is misleading to imply the change is mostly due to the effect of PSSE and reduced Cobb angles. It is good indeed that curves did not progress, and patients are now close to maturity but here it would be more relevant to refer to natural history data showing how patients with certain curve magnitude at maturity progress into adulthood. EG.

Response: Fantastic point, thanks for pointing it out, we realized that comparing LCR-value after two years follow-up was inappropriate. Therefore, we withdrew the comparison of LCR-value at the 2-year follow-up and replaced it to a description of how much participants reached skeletal maturity (Risser 5) and presented with a curve less than 30 degrees. Please see **lines 366 to 368 and Table 3.**

Comment #31: L366 to 369 again you emphasise a different in the patterns of curve angles changes over time between groups when you ANOVA suggested that there was no interaction. There could

be data distribution issues affecting the results here. Which ANOVA model did you end up using? Was your data meeting all other assumptions than sphericity?

Response: Sorry for this confusion, agree that if there were no interaction effects in reducing Cobb angles, it was inappropriate to further compare variables between groups. In this study, the significance was only detected in the within-subjects comparison, having a time effect in reducing Cobb angles for all participants (observed power 0.7, **lines 275 - 278**). A separate repeated analysis with a Bonferroni adjustment (control type I error) further revealed that the significant reduction of Cobb angles was dominant in the lumbar group (observed power 0.95, **lines 282 - 283**). Therefore, we rewrote this part (please see **lines 371 to 372**). Levene's test indicated that Cobb angles at the baseline met homogeneity of variance assumption (p=0.5). Mauchly test indicated that the assumption of sphericity was violated (χ^2 =32.1 , df =5 , $p < 0.001$), hence, the degrees of freedom were corrected using Greenhouse-Geisser estimates of sphericity. Please see **lines 271 to 276**. The assumption of Greenhouse-Geisser was met with an Epsilon value 0.72.

Comment #32: L371 What do you refer to when stating "Progression value was comparable between groups at the study initiation"?

Response: Sorry for the confusion, the comparable here means the proportion was similar between groups, the clarification was made on **lines 376 to 378.**

Comment #33: L372 373 Since you did not measure spinal flexibility please refer instead to "by the assumed more important flexibility of the lumbar spine than the thoracic spine.

Response: Fantastic points. Please see the reediting on **lines 378 to 379**.

Comment #34: L378 to 381. I would avoid suggesting that muscle actions have a bigger role for lumbar corrections and that only spiral breathing play a role in the thoracic regions. Modern Schroth instruction does not make assumptions about what muscles do or how corrections are done it simply offers a variety of cues and correction instructions and facilitation technique to get the desired movement without emphasis on how.Maybe the key is that breathing is more related to thoracic ok. The axial elongation, side shift are done in both regions. The difficulty with breathing is likely relevant.

Response: Nice points, thanks for this explanation, the revised discussion was made on the **lines 385 to 387.**

Comment #35: L405. Please cite Kuru et al for the effects of supervision vs not.

Response: cited. Thanks for this recommendation. Please check **reference 49**.

Comment #36: L417 There are more types in the whole BSPTS classification. Maybe instead rephrase to suggest that your sample included patients representing 5 different types from 8 in the BSPTS classification.

Response: Nice points, thanks for this explanation, the revised discussion was made on **lines 423-424.**

Comment #37: L435 Refer to hypothesized higher flexibility or previously shown higher flexibility. You did not show that in your study.

Response: Thanks for this reminding, we acknowledge this limitation on **lines 444 to 445.**

Comment #38: In this limitation section please clearly acknowledge that your regression is underpowered.
Response: Indeed. The acknowledgement was made on **lines 448 to 454.**

Comment #39: In the data supplementary file please replace the variable name by study id. Remove initials and replace by participant id numbers to fully comply with confidentiality of study data.

Response: Done, thanks for this important recommendation. Please check the updated **S4 File. Study data.**

Comment #39: I have added edits in the text using the PDF comment function with strikethrough for deletions and insertions in blue.

Response: We have revised the format accordingly. Deeply appreciate your detailed comments.
