# Supplementary material for: Does curve pattern impact on the effects of physiotherapeutic scoliosis specific exercises on Cobb angles of participants with adolescent idiopathic scoliosis: A prospective clinical trial with two years follow-up
Source: PLoS One. 2021 Jan 25;16(1):e0245829. doi: 10.1371/journal.pone.0245829 (PMC7833215; doi:10.1371/journal.pone.0245829)
Supplement: Attachment — Submitted filename: Response to reviewers.docx [file pone.0245829.s007.docx]

REPLY TO REVIEWERS

**Re: Ms. No. PONE-D-19-35759 - "Dose Curve Pattern Impact on the Effects of Physiotherapeutic Scoliosis Specific Exercises on Cobb angle of Adolescent Idiopathic Scoliosis: A prospective clinical controlled trial with two years follow-up”**

Dear Editor,

The authors would like to thank you and the Reviewers for all of your time and effort devoted to the review of our aforementioned manuscript. The Reviewers’ comments were indeed extremely insightful and greatly appreciated. As such, the authors would like to take this opportunity to address each and every concern the Reviewers noted in their review of our submission. In addition, where appropriate, we have also revised our manuscript accordingly. Major changes are made in light gray.

We believe that the Reviewers’ comments have helped improve the quality of our manuscript. We hope that you and the Reviewers will find our revised work suitable for publication in *PLOS One.*

**Editor**

***Comment #1:***  Journal Requirements:

When submitting your revision, we need you to address these additional requirements.

1. Please ensure that your manuscript meets PLOS ONE's style requirements, including those for file naming. The PLOS ONE style templates can be found at

https://journals.plos.org/plosone/s/file?id=wjVg/PLOSOne_formatting_sample_main_body.pdf and

<https://journals.plos.org/plosone/s/file?id=ba62/PLOSOne_formatting_sample_title_authors_affiliations.pdf>

***Response:*** We have revised the format accordingly.

***Comment #2:*** 2. Thank you for submitting your clinical trial to PLOS ONE and for providing the name of the registry and the registration number. The information in the registry entry suggests that your trial was registered after patient recruitment began. PLOS ONE strongly encourages authors to register all trials before recruiting the first participant in a study.

As per the journal’s editorial policy, please include in the Methods section of your paper:
Please give confirmation that all related trials are registered by stating: “The authors confirm that all ongoing and related trials for this drug/intervention are registered”.

***Response:*** We have revised this. Please see **lines 108-110, page 7** (Materials and methods: study design)

***Comment #3:*** 3. You indicated that you had ethical approval for your study.

In your Methods section, please ensure you have also stated whether you obtained consent from parents or guardians of the minors included in the study or whether the research ethics committee or IRB specifically waived the need for their consent.

***Response:*** Thank you for the comment. Please see **lines 105-107,page 7** (Materials and methods: study design)

***Comment #4:*** 4. We note that you have stated that you will provide repository information for your data at acceptance. Should your manuscript be accepted for publication, we will hold it until you provide the relevant accession numbers or DOIs necessary to access your data. If you wish to make changes to your Data Availability statement, please describe these changes in your cover letter and we will update your Data Availability statement to reflect the information you provide.

***Response:*** No change is needed.

***Comment #5:*** 5. Please amend either the title on the online submission form (via Edit Submission) or the title in the manuscript so that they are identical.

***Response:*** Thank you this has been changed. The title is now: **Dose curve pattern impact on the effects of physiotherapeutic scoliosis specific exercises on Cobb angles of participants with adolescent idiopathic scoliosis: a prospective clinical trial with two years follow-up**

***Comment #6:*** 6. Please amend your list of authors on the manuscript to ensure that each author is linked to an affiliation. Authors’ affiliations should reflect the institution where the work was done (if authors moved subsequently, you can also list the new affiliation stating “current affiliation:….” as necessary).

***Response:*** This has been done.

***Comment #7:*** 7. Please include your table as part of your main manuscript and remove the individual file.

Please note that supplementary tables should be uploaded as separate "supporting information" files.

***Response:*** This has been added.

***Comment #8:*** 8. We note that Figures 3 and 4 include images of patients.

As per the PLOS ONE policy ([http://journals.plos.org/plosone/s/submission-guidelines#loc-human-subjects-research](about:blank#loc-human-subjects-research)) on papers that include identifying, or potentially identifying, information, the individual(s) or parent(s)/guardian(s) must be informed of the terms of the PLOS open-access (CC-BY) license and provide specific permission for publication of these details under the terms of this license. Please download the [Consent Form for Publication in a PLOS Journal](about:blank) ([http://journals.plos.org/plosone/s/file?id=8ce6/plos-consent-form-english.pdf](about:blank)). The signed consent form should not be submitted with the manuscript, but should be securely filed in the individual's case notes.

Please amend the methods section and ethics statement of the manuscript to explicitly state that the patient/participant has provided consent for publication: “The individual in this manuscript has given written informed consent (as outlined in PLOS consent form) to publish these case details”.

If you are unable to obtain consent from the subject of the photograph, you will need to remove the figure and any other textual identifying information or case descriptions for this individual.

**Response:** Thank you for the comment. We have included the necessary documents. The statement was made on **lines 107 to 108, page 7** (Materials and methods: study design).

***Comment #9:*** 9. Please include captions for your Supporting Information files at the end of your manuscript, and update any in-text citations to match accordingly. Please see our Supporting Information guidelines for more information: [http://journals.plos.org/plosone/s/supporting-information](about:blank)

***Response:*** These have been included.

**Reviewer #1**

***Comment #1:*** The article entitled “Dose Curve Pattern Impact on the Effects of Physiotherapeutic Scoliosis Specific Exercises on Cobb angle of Adolescent Idiopathic Scoliosis Subjects: A prospective clinical controlled trial with two years follow-up” has a good topic and matter in the field. However, the methodology of the study needs to be revised. The authors investigate the effects of scoliosis-specific exercise method on different curve patterns (major thoracic and major lumbar) and in long term. The idea is valuable. But the cobb angle range is wide, therefore the inevitable effect of brace wearing for some participants on the results looks unnoticed. Randomization is needed for age, gender, cobb angle, curve pattern, brace wearing, in-brace correction, risk of progression... etc. Before consideration for publication, there may be following suggestions to consider again:

Abstract - In method, information regarding Cobb angle of the subjects are needed. Was curve magnitude similar between thoracic and lumbar curve groups?

***Response:*** We thank the reviewer for the comments and we will try to amend our manuscript accordingly.

The curve magnitude was similar between groups: 28.5 ± 8.4 vs 26.8 ± 10.1, p=0.6.

Please refer to the 1) page 3, **abstract:** results: **lines 29 - 31;**

2) **results,** participants, **lines 222 -227;**
3) **Table 1: line 237**

***Comment #2:***  Did patient undergo only exercise intervention or did they use bracing as well?

***Response:*** A total of **10 participants** were wearing brace before commencing the PSSE program and having comparable, 37% of correction effects from bracing.

Please refer to the 1) **results: participants, lines 231 - 235. 2) Table 1: line 237**

***Comment #3:*** The first sentence of the background needs to be revised related with exercise. The statement is associated with brace and surgery.

***Response:*** We thank the reviewer for this suggestion. This has been revised. Please refer to **page 2, abstract: background, lines 10-11**

***Comment #4:*** What were patients’ ages? Was age similar between groups?

***Response:*** Yes, the age was under evenly distributed and was similar between groups.

Please refer to the **page 3, abstract: results,** **lines 30 to 31, Table 1 (line 237) and lines 227 to 228 of page 15.**

***Comment #5:*** Introduction

- The importance of curve progression risk in conservative treatment was extensively reported. But did you randomize your patients in terms of bone maturity among groups?

***Response:*** Thanks for comment. This study was planned as a case control study. Participants were recruited from one scoliosis clinic and grouped into two groups according to their curve pattern. The progression related data was analyzed at the baseline and correlation analysis was performed to detect any influence of these factors, including bone maturity, on the reduction of Cobb angles. Please refer to the methodology, study design, **lines 119-123, line 125** (**S2** Fig. 1: CONSORT flow diagram**), line 237 (Table 1) and lines 210 to 213 (statistical analysis).**

***Comment #6:*** Did you measure curve flexibility? If so, how did you measure? Because you reported that this study aimed to investigate the long-term therapeutic effect of PSSE on thoracic and lumbar major curves and to determine whether a more flexible lumbar curve would respond better to exercise.

***Response:*** This is an important point by the reviewer. Unfortunately we were unable to quantify spinal flexibility prior to treatment in this study due to patients’ radiation concern, therefore, **the hypothesis was re-centered on curve patterns.** Please see the text at **lines 94 to 99 (Introduction), lines 369 to 374 (discussion); lines 433 to 441 (Study limitation).**

***Comment #7:*** Methods

- One of your inclusion criteria was having cobb angles: from 10 degrees to 50 degrees. This means that some of your patients had bracing indication. How many of your patients did wear brace in this period? Then how did you differentiate exercise specific effect?

***Response:*** Ten participants (5 were wearing full time brace whereas 5 were wearing only night time brace), were wearing brace and achieved comparable in-brace correction 37%, which explained the effects from bracing and allow this study emphasized on the effect from the PSSE.

Please check the **Table 1 (line 237)**, **lines 231 to 235 (results) and lines 349 to 353 (discussion).**

***Comment #8:*** Please add detailed explanation about The Rigo Scoliosis Classification system.

***Response:*** Rigo clasification was adopted to rigo cheneau brace fabrication and also BSPT Schroth methods, in which 5 classifications with 8 sub-types were clearly introduced. two important references was cited accordingly, **please see references 22 and 23.**

***Comment #9:*** You included major thoracic and major lumbar curves. I see from the method that some of them, who had major thoracic curve, had double curves or secondary lumbar curves. This would affect results. We know that single lumbar curves and double curves are more common than other curve pattern in idiopathic scoliosis. Single thoracic curves are rare. Therefore, curve distribution among groups would differ. In table 1, curve pattern was reported as double and single, only. But more information is needed regarding curve pattern distribution.

***Response:*** We thank the reviewer for these comments. This information was revised in:

1. **Lines 222 to 227.**
2. **S2: Fig.1 CONSORT flow chart**
3. **Table 1.**
4. **Please also see lines 416-426 of paragraph-study limitation.**

***Comment #10:*** For single curve, how many patient had thoracic, how many patient had lumbar? For double curve, how many had primary thoracic? how many had primary lumbar? What was curve pattern distribution of the participants?

***Response:*** Thanks for your suggestion. The additional information has been added to:.

1. **Lines 222 to 227.**
2. **S2: Fig.1 CONSORT flow chart**
3. **Table 1.**

**Please also see lines 416-426 of paragraph-study limitation.**

***Comment #11:*** How was curve range for thoracic and lumbar curves? How many vertebrae did curves include? This would affect results. What do you think?

***Response:*** Fantastic comment, we do agree that the range of the curvature would definitely impact on the treatment effects, in terms of that may affect the spinal segmental flexibility. However, curve range should also be correlated with curve pattern, such as a short range should be observed in single lumbar curves whereas a long range should be observed in a thoracolumbar, etc. This idea is good but unfortunately out of scope of this study, the sample size of each curve pattern was small in our study, therefore, extra analysis of range of curvature may cause distraction from our primary outcome. Moreover, it will be too informative and overwhelming to readers of those who are not familiar with scoliosis radiographic classification. However, it will be interesting to study the effects of PSSE on the different radiographic parameters. Please see this amendings to **lines 446-447 of paragraph conclusion.**

***Comment #12:*** Did you measure axial trunk rotation of the participants? Curve magnitude would affect from trunk rotation parameter as much as Cobb angle?

***Response:*** This is an important parameter that will be studied in a future study. We wanted to mainly focus on the Cobb angle changes in this manuscript. Further investigation of the cosmesis and quality of life will also be performed. Please see **lines 447-448 of paragraph Conclusion.**

***Comment #13:*** Did you use and patient-reported outcome measures? or Did you assess any clinical parameters such as trunk symmetry and cosmetic deformity?

***Response:*** As discussed in comment #12, these are important parameters to study. We have not included them in this manuscript because we wanted to focus only on curve magnitude changes.

***Comment #14:*** Did any of your patient use spinal brace in this period? When considering wide range of Cobb angle, this looks inevitable. How was the distribution of spinal brace usage among groups? How was in-brace correction for patients who underwent spinal brace intervention? Because if the correction amount differ among groups, this would increase or decrease the exercise effect.

***Response:*** Please check the **Table 1** and **lines 231 to 235; lines 323 to 326** of paragraph results, **discussion: lines 349 to 353; lines 405 to 408.**

***Comment #15:*** Male distribution is higher in group B. Do you think this would affect results? Also bone maturity distribution among groups seems to be different with 11 - 11 in group A, and 12-6 in group B. Group B looks to have participant who had low risk of progression.

***Response:*** These are important confounders. Using the logistic correlation analysis, we did not find any significant relationship with gender and maturity.

Please refer to **lines 323 to 326, Table 1.**

***Comment #16:*** In table one, minimum and maximum values are needed.

***Response:*** Range was reported **in the abstract;** Mean ± SD and 95% CI have been added **in the Table 1.**

***Comment #17:*** Did authors use any randomization method for age, gender, Cobb angle, curve progression risk (bone maturation)? If not, how would it be possible to make exact comparison of two different curve pattern groups (thoracic and lumbar)

***Response:*** It was convenient sampling with a curve pattern controlled study, the assumption of normality was calculated before commencing the study and analysis was conducted for baseline data between groups. Logistic regression analysis was adopted to detect any correlation of variables that differed on baseline between groups and treatment effect, which was reported on **lines 210 to 213; lines 323 to 326,** curve distribution can be found in **S2 Fig 1** and **table 1.**

***Comment #18:*** I am sending my suggestions until here. Discussion is also needed to be revised according to methodology.

***Response:*** Thanks for your valuable suggestions and hope you will find the updated discussion interesting.

**Reviewer #2**

***Comment #1:*** The curve title should be corrected to: Does Curve Pattern Impact the Effects of Physiotherapeutic Scoliosis Specific Exercises on Cobb angles Participants with Adolescent Idiopathic Scoliosis: A prospective clinical controlled trial with two years follow-up.

***Response:*** We thank the reviewer for this suggestion and this has been revised.

***Comment #2:*** It is best to refer to participants or persons with a diagnosis rather than subjects. Throughout the text please replace patients by participants or persons.

***Response:*** These have been corrected.

***Comment #3:*** ABSTRACT Please specify details of the intensive frequency and reduce frequency. (number of visits per week or month and their duration of exercising at minimum). Specify if a home program was performed.

***Response:*** This has been done. Please refer to the **page 2, abstract, methods, lines 19 to 23.**

***Comment #4:*** If the study is controlled. I assumed there are two groups. Specify what the control group did during the trial in the abstract.

***Response:*** This was planned as a clinical, curve pattern controlled study. Please refer to the **page 2, abstract, methods, lines 17 to 19. S2 Fig 1 Consort flow diagram.**

***Comment #5:*** Then the analysis should likely be a mixed model ANOVA with a between subject group factor and a within subject (repeated measures) time factor. There should not be multiple paired t-tests if you choose a relevant pairwise comparison over time within curve patterns. Using separate t-tests would increase your risk of type one error.

***Response:*** We thank the reviewer for this astute comment. The analyses have been corrected according to your valuable suggestions, please see **pages 2-3: abstract, method (abstract): lines 24 to 26; statistics lines (main text): 196 to 213; results (abstracts): line 34-39; results (main text): lines 259 to 291; Tables 2,3.**

***Comment #6:*** There are numerous English issues. I will not point all of them as English is a second language for me too. Please have the paper reviewed by an English editor.
EG always use major thoracic curve and major lumbar curve.

***Response:*** Thank you for the comments. This has been amended accordingly.

***Comment #7:*** Specify the recruitment setting and method for the participants.

***Response:*** This has been done. Please see **page 7, methods, study design, lines 103 to 105; lines 110 to 123** and **S2 (Fig. 1: CONSORT flow diagram).**

***Comment #8:*** Beyond stating no difference in the curve magnitude between groups please report the curve means and SD and how many patients if any were braced. If none wore a brace, specify.

***Response:*** Ten participants (5 were wearing full time brace whereas 5 were wearing only night time brace), were wearing brace and achieved comparable in-brace correction 37%, which explained the effects from bracing and allow this study emphasized on the effect from the PSSE.

Please check **lines 132 to 135 of page 9**, the **table 1** and results, **lines 231 to 235**; discussion, **lines 348 to 351.**

.

***Comment #9:*** Spell out d on first use for D-value.

***Response:*** The D-value stands for the difference value of Cobb angle over three testing times with the initial Cobb angle, to detect the regression, stabilization and deterioration between groups. **This has been reiterated in lines 203-206 and table 2.**

***Comment #10:*** Since we don’t know how you calculated d, we don’t know in the abstract if the change over 1 year is a improvement or deterioration in Cobb angles in each group. The sentence with the change in COBB for the specific intervals should specify which group had which results. There should be multiple F statistics and p-values with your design: one for the interaction between groups and time, one for the main effect of group and one for the main effect of time.)

***Response:*** We are sorry for this confusion and the analysis has been clarified. Please see the **revised abstract, methods (lines 203-206), results (lines 259 to 365) and Table 2.**

***Comment #11:*** The value for the regressions in group B do not seem to map to any of the values in the sentence prior. How were these group be reductions calculated.

***Response:*** Regression was defined as a decrease of Cobb angle ≥ 6 degrees, the percentage value in each group was reported in the results session, in addition, raw Cobb angle was also analyzed using the repeated measures, please refer to the **outcome measurement (lines 184 to 186) and results (lines 259-265; lines 272 - 291)**. All results, including mean, SD, 95% CI of the D-value and of the raw Cobb angles, were reiterated and summarized in the **Table 2 and Table 3, respectively.**

***Comment #12:*** Report data on the compliance and drop outs with the exercise program. Did all 40 participants attend all follow-up. If yes specify this is a per protocol analysis and report how many had started and were not included in the analyses.

***Response:*** There were no dropouts through the whole studies. Per-protocol analysis was specified accordingly. Please see **lines 253 - 257**; **S2: Fig.1 CONSORT flow diagram**

***Comment #13:*** The conclusion is inaccurate: This was the first study to investigate whether PSSE can lead to curve regression based on location of the major curve. The following study examined the effect of curve type in a multivariate analysis of the short-term effects of Schroth on Cobb angles. Schroth Physiotherapeutic Scoliosis-Specific Exercises Added to the Standard of Care Lead to Better Cobb Angle Outcomes in Adolescents with Idiopathic Scoliosis - an Assessor and Statistician Blinded Randomized Controlled Trial. Schreiber S, Parent EC, Khodayari Moez E, Hedden DM, Hill DL, Moreau M, Lou E, Watkins EM, Southon SC.
PLoS One. 2016 Dec 29;11(12):e0168746. doi: 10.1371/journal.pone.0168746. eCollection 2016.

***Response:*** Thanks for you pointing this out, the conclusion and discussion has been revised to “**an extension of previous studies”.**

***Comment #14:*** Introduction

In the intro where you state the following about the reviews it may be important to review each paper in those reviews for discussions of the effects of curve type. The reviews did not address this question but some of the original reviewed trials did. however, in these studies, the relationship of curve location with correction effects was not clearly discussed, and there was only a short-term followup. There may be limitations to those as short follow-ups which would still justify your work that you could highlight.

***Response:*** Thanks for this brilliant suggestion, the introduction was revised and followed your suggestions, please refer to the **lines 77 to 85 (Introduction).**

***Comment #15:*** The following intro statement is also overreaching. Schreiber et al above did include baseline Cobb angle in their study of the effect of Schroth. There maybe limitations to this understanding but it is not completely unknown. You stated “the influence of curve magnitude on exercise outcomes is unknown.”

***Response:*** We apologize for the oversight. This has been corrected.

***Comment #16:*** The objective as stated focuses on curve magnitude and flexibility rather than relation between outcomes and curve types. Your focus seems to be on curve type in the abstract. Please re-center the objective on this topic.

***Response:*** We thank the reviewer for this comment. **It has been re-centered.** Please see **lines 94 - 98.**

***Comment #17:*** Specify your recruitment methods and setting. Were all consecutive eligible participants invited? Only referrals from some DRs…

***Response:*** patients were recruited from a scoliosis clinic, therefore, all consecutive eligible participants were invited and decision was made according to the inclusion/exclusion criteria. Please **see lines 111 to 123 and S2 Fig 1.**

***Comment #18:*** Please add a justification to the exclusion of hypermobility. This has not been recommended by SOSORT and has not been done in other study. Maybe, if possible, report how many patients were excluded on this basis.

***Response:*** There were 18 subjects were detected with hypermobility, in form of Beighton score >4, who were excluded from this study. In addition, a total of sixty-two subjects were excluded after eligibility assessment, the exclusion reasons were specified on the **lines 115-119; lines 218 to 222 (Results: participants) and S2 (Fig.1 CONSORT flow diagram).** One previous study reported hypermobility as a dominant presence in the single curve type and should be taken into account during physiotherapy. Participants with hypermobility were excluded to control/limit the systematic error in this study, the related paper was cited in accordingly. **Please see reference 21.**

***Comment #19:*** Please specify if patients could have been prescribed a brace at baseline with the exercises or not in the selection criteria.

***Response:*** Yes, they could have. Please see line 115 (exclusion criteria: previous treatment) and **lines 132-135 (paragraph: study interventions).**

***Comment #20:*** From a Asklepios (German) Schroth trained perspective grouping N3N4 with 3C is not consistent with only grouping thoracic curves. Please acknowledge as a limitation that the N3N4 group likely had both thoracic and lumbar curves and may have needed different treatment than the 3C curves. For example the Chest twister exercise is not indicated for N3N4. Similarly. The type 4C curves do have a lumbar dominance but also have a thoracic curves. What did you do with the pure lumbar or thoracolumbar curves? Specify if you had any in your sample.

***Response:*** N3N4, BSPT approach, is a bit different from the German approach, it mainly focuses on correction for thoracic curve, like gentle chest twisting was applicable but should perform without changing the balance and without over correction. By contrast, the double curve in the German approach, takes the lumbar curvature in priority, therefore using pelvic strategy more than chest twisting, etc. The principles are mostly same but the practical metric is a bit different. We did not explain too much or try to compare the two methods in this manuscript, we were trying to prove the effects of Schroth with Cobb correction instead of centering on how did we position patients. Explaining this practical metric is valuable but may be overwhelming for readers who are not familiar with Schroth, moreover, the dynamic feedback of the spine with exercise is unclear yet. Therefore, after all consideration, we did not include this explanation in our manuscript.

***Comment #21:*** Please specify the certification of the Schroth therapists BSPTS -rigo, or Asklepios or ISST or Weiss…

***Response:*** BSPTS approach, we firstly registered as BSPTS in 2016, then registered Asklepios in 2018, therefore, mainly BSPTS concept in this study. Please see **lines 129.**

***Comment #22:*** When were participants asked to complete the simple home exercise compliance questionnaire (Weekly, monthly, once at then of the study…)

***Response:*** Monthly. Please see **lines 137-143.**

***Comment #23:*** In listing exercises could the figure order be arranged to match the order of presentation in the text.

***Response:*** Sorry for the confusion. This has been amended.

***Comment #24:*** There is a variation of the muscle cylinder for thoracic curves. Specify why it was not used.

***Response:*** Please see lines **153-161**. This was not considered due to the pain at the knee joint.

***Comment #25:*** Describe how the those was chosen. Did the patient do the same exercise throughout the program? Acknowledge as a limitation why you did not use dynamic exercises such as walking or …

***Response:*** We thank the reviewer for this astute comment. Please see **lines 158-161**.

***Comment #26:*** Throughout the paper: I recommend avoiding the labels A and B for the groups and clearly stating thoracic vs Lumbar groups.

***Response:*** We thank the reviewer for this comment. It has been changed.

***Comment #27:*** Figure 5 and 6 Please add a justification for why these participants were not braced in addition to doing the exercises.

***Response: Two p*atients were mostly considering about the cosmetic issue;** especially one girl was at late Risser stage (Fig 5) and bracing was not compulsory for her, then this girl tried exercise for a half year and resulted with a significant correction, then she had never considered bracing again.

***Comment #28:*** Table 1. Add units for age.

***Response:*** Added. **Table 1**

***Comment #29:*** Replace gender by Sex. I doubt you documented gender.

***Response:*** This has been done.

***Comment #30:*** Why not report count and percentage as you did for sex instead for Sanders stage.

***Response:*** The Sanders stage has been replaced by Risser, because of the progression risk value in terms of the Lonstein and Carlson score, that needs the Risser for calculation. **Please see the S4 File: raw data set and Table 1.**

***Comment #31:*** Can you specify brace types. This is a notable imbalance and surprisingly more braced in thoracic group?

***Response:*** Thanks for the comment. Please see **lines 231 - 233** on page 9. All (n=10) were wearing Cheneau brace **(Table 1).**

***Comment #32:*** For future meta-analysis purpose it may be valuable to still measure thoracic and lumbar curves in both groups.

***Response:*** We agree with the suggestion. Please see **Table 1**, **S2 (Fig. 1: CONSORT flow Chart) and S4 File: study data.**

***Comment #33:*** Rather than or in addition to, please report the number of N3N4 vs 4C per group or add a note to each column showing the thoracic double curves were all N3N4 and the Lumbar were all 4C.

***Response:*** This has been added. Please see **S2 (Fig. 1: CONSORT flow Chart) and the Table 1.**

***Comment #34:*** Specify in the methods if the classification was clinical only or informed by radiographs also.

***Response:*** Radiographic, BSPTS is using this classification system for bracing too, therefore the X-ray is in priority for classification and the clinical presentation was only for clinical reference.

Please see **lines 119 to 120 and S2 (Fig.1 CONSORT flow diagram.)**

***Comment #35:*** Can you report if all thoracic curves were right side and all lumbar were left sides. If not please report the distribution.

***Response:*** This has been clarified in **lines 222-227, (S2** Fig.1 Consort flow diagram) and **Table 1.**

***Comment #36:*** Figure 2 could present statistical significance of the comparison of the values as well with symbols.

Specify what the error bar represent (standard deviations or standard errors).

***Response:*** The new figure 1 has been amended. The previous **Figure 2 was replaced by the table 3** shows raw data of Cobb angles over four testing time points, in which mean +/- SD, 95% CI and range were all presented, which may be of value for future meta-analysis.

***Comment #37:*** Figure 3. It is unfortunate that the patient has a wide racer back bra which hides much of the postural defect in thoracic spine. The semi-hanging picture has allowed too much hypokyphosis. Would you have a semi-hanging example where the sagittal kyphosis is better maintained. The aspect ratio of the photo f seems off (too wide not tall enough).
Can you add the left right marker to the radiograph. It show right thoracic to the right of the image.

***Response:* A new picture has been uploaded**. Unfortunately, we cannot find a better photo as patients cannot come to our center due to COVID. Therefore, I used her another pic for demonstration, please check see whether it is acceptable.

***Comment #38:*** Figure 4. Can you add the left right marker to the radiograph. This one shows the right thoracic curve on the left of the image. Once again in C the patient shows hypokyphosis and this time has lost physiological lordosis. Can you find a demonstration of semi-hanging in this group with better sagittal profile. For G, can you have an example with proper head alignment.

***Response:*** This has been amended.

***Comment #39:*** Figure 5 and 6 were presented with figure 6 first. No need to use initials in the figures. Label as example of a thoracic vs lumbar participants. Could you draw the Cobb measurements on the images? Could the scaling of the two spine images in fig 6 be made more similar?

***Response:*** This has been added.

***Comment #40:*** The dataset uploaded should also include a data dictionary or a legend. What values are coded 1 and 0 … Define cut point 30 deg. Is the data incomplete. What appears in columns V to AO??

***Response:*** Done, the file has been amended. The other columns were including other data such as ATR, SRS-22 etc. We have deleted them as the data on radiographic measurements were used in this study. Please see **S4 File: study data.**

***Comment #41:*** Specify in the selection criteria what was the criteria for brace prescription and brace termination and how you monitored brace compliance. Detail the kinds of brace that were prepared and possibly the targeted or achieved in brace correction if available.

***Response:*** The SRS bracing criteria was used by doctors to prescribe brace treatment, see **lines 132 to 135,** and the reference was cited accordingly. Brace termination was defined with skeletal maturity, and bracing compliance was monitored using a monthly self-reported adherence checklist, **see lines 139 to 143**. In-brace correction and brace type were clarified on **lines 233 to 235** and **Table 1.**

***Comment #42:*** Can you detail the positioning instructions for the participants during the radiographs.
In clinical trials the study is not powered to compare group characteristics at baseline. It does not mean much to do statistical comparisons of those. Key is whether the estimate appear to present clinical differences before deciding whether to control for those.

***Response:*** amended accordingly. **Please see line 144 to 153. Picture legend of Fig. 2 and Fig. 3.**

The comparison of the baseline data was to show the assumption of normality, if the baseline was evenly distributed and which can power our study quality. However, I fully agree with you that a clinical trial should look more on the factors that may affect results, but we have to do so according to suggestions from other reviews. In this study, with no deterioration after 2 years, the logistic regression was conducted to analyze the relationship of age, sex, brace etc. and treatment result, which further explained the influence of aforementioned factors on the Cobb reduction. **See lines 323 to 326 (Results).**

***Comment #43:*** Clarify how the d-values were computed. Why not describe it as a mixed model anova with a between group factor and a time factor and use appropriate pairwise characteristics. It appears to be what you did given the output provided. Can you specify which pairwise was requested? LSD? The use of multiple t-tests to detect regression increases the type one error chances.

***Response:*** LSD was used. We have corrected the analyses appropriately. We thank the reviewer for these valuable suggestions.

***Comment #44:*** Results:

Subjects. I disagree that groups were sufficiently similar for bracing.

***Response:*** Our comment was based on the results of the X^2^ test which showed no statistical difference. Nevertheless, logistic regression was performed to test whether bracing was an impact on the results. **See lines 323 to 326 (Results).**

***Comment #45:*** From the selection criteria I believed no participant could have started any scoliosis treatment before enrollment. Here I learn that bracing could have been started prior to PSSE. This should be clear from the patient selection criteria.

***Response:*** We thank for reviewer for this valuable suggestion. The patients started with either bracing or exercise would be excluded in this study, please see **S2 (Fig. 1) and lines 132 - 135.**

***Comment #46:*** Can you report brace compliance in both groups?

***Response:*** **Please see line 233 to 235 and Table 1,** because of there was no correlation of bracing with regression effects, the bracing hour, bracing compliance from those 10 participants were not further discussed.

***Comment #47:*** Why only report the percent radiograph refused at 6mth into PSSE. Why not also for the other time points.

***Response:*** All participants completed radiographic assessment on the baseline, 1-year, 1.5-year and 2-year follow-up, therefore per-protocol analysis was performed. Please see **lines 256 – 257. lines 272 to 273 and S2 Fig.1.**

***Comment #48:*** Specify if the following statement applies only to group averages or also to patient individual data: There was no deterioration of the major curvature in either group at the 2-year follow-up.

***Response:*** It applies to both average and individual data. No deterioration was noted for either group. Please see **lines 259 to 260 and Table 2.**

***Comment #49:*** Where you report the F and P-values you need three F values. The key one is for the interaction between group and time, then you also need main effects on groups and on time. (your output show all were not significant.

***Response:*** The analysis was re-performed by a statistician who was blinded to this study. We thank the reviewer for this important comment. Three F values of comparisons in D-value of Cobb and raw data of Cobb, respectively, were clarified and reported. **See lines 263 to 265, table 2,3,4 and lines 275 – 277.**

***Comment #50:*** Report the Sanders secondary analysis as a new paragraph. Announce this interest in objective section as a secondary objective.

***Response:*** The Sanders staging has been replaced by Risser as we needed the Risser for evaluation of progression risk.

***Comment #51:*** If the interaction effect is not significant it is not good statistical practice to explore pairwise within groups with t-tests. IF you had set your mixed method anova with Cobb at baseline, 1 year, 1.5 yrs and 2 yrs you may have detected this effect already. Running a different approach with t-test exposes you to a higher risk of type 1 errors.

***Response:*** Fully agree, beside of analysis of D-value of Cobb reduction over three testing times, we re-performed analysis on the Raw Cobb angle over four testing timepoints which suggested a significant time effects in reducing Cobb angles (repeated one way anova).

***Comment #52:*** It would be possible to use a chi-square analysis to compare the distribution of the improved, stabilized and deteriorated in each group statistically in addition to just report the results. A statistician may be able to determine if the three time points could be compared in a single analysis for these proportions to protect against a type one error.

***Response:*** We have proceeded to the reviewer’s suggestions. Unfortunately, the X^2^ analysis also showed no significance, therefore, we further analyzed the relationship of influencing factors, including age, brace, sex etc and the regression effects using logistic regression analysis. See **lines 210 to 213 and lines 323 to 326.**

***Comment #53:*** See above. The following opening statement of the discussion is overreaching: This is the first study to investigate the outcomes of different curve types undergoing PSSE treatment for AIS.

***Response:*** The statement has been corrected. Please see **lines 341 -342** (discussion).

***Comment #54:*** You cannot state the following as all your groups received exercises. You did not show superiority to an alternative. And you did not discuss historical controls. Therefore, our results further revealed the superiority of PSSE programs in the stabilization and regression of scoliosis.

***Response:*** We thank the reviewer for the sentiments.

***Comment #55:*** In the discussion you state: This met our original study hypothesis that lumbar major curves are more flexible. I am not sure this is the only possible physiological explanation. You did not measure flexibility per say. Please justify your statement and address possible alternatives as well. Discuss limitations of not having measured flexibility directly.

***Response:*** We thank the reviewer for these suggestions. Please see **lines 369-389** and **lines 433 – 441.**

***Comment #56:*** P13. Compare your results of lumbar effects being better to those of Schreiber et al. I believe they did not find the same curve pattern as the one with the best response. Could it be that some benefits for your thoracic group may have been missed due to not using the chest twister and prone/supine exercises

***Response:*** It is possible, we thank the reviewer for this valuable discussion point. I remember that there were conference papers, presented in SOSORT 2019 in SF, there were evidences showing that the spine responded better during exercise in prone. However, to our knowledge, this evidence was not published yet and unfortunately our study was initiated before 2019. Positions were designed to cope with patients’ daily activity as much as possible in this study. In Schreiber’s study, they used chest twist for thoracic curve, however, their results also showed the 3CP was significantly correlated with the largest Cobb after treatment, which was explained by the proven higher progression risk was shown in thoracic type. Therefore, it will be not suitable to compare our study with theirs on this point, because they also found the less effects with 3CP. Therefore, we alternatively stated that our study extended their results for the long term effect (**lines 341-344**) Moreover, for the potential variation caused by varied exercises, we further explained in **lines 372-389,** which was more emphasizing on the spinal biomechanics instead of varied positioning during exercise.

***Comment #57:*** Please discuss the limitation of self-reporting compliance and possible recall issues depending on when your participants completed the compliance questionnaires.

***Response:*** Participant need to take a note and sign on his checklist on the day when they performed exercise, then hand the checklist to physiotherapist at their next visit. See **lines 139 – 143,** recall effects were discussed on **lines 405 - 408.**

***Comment #58:*** Limitations. There are more than 4 patterns in RIGO classification. Type 1 and type 2 were not discussed here.

***Response:*** Information added. **See lines 416 to 426.**

***Comment #59:*** Please discuss not monitoring co-interventions such as manipulations, massage, other fitness activities or self-used of off the shelve bracing.

***Response:*** The participants would agree with only PSSE but no alternative exercise before sign the study consent form, but we agree that participants are likely to take another therapy without informing us. Commencing any other treatment were excluded from this study, please see **line 115 and S2 Figure.**

***Comment #60:*** Could you review how many patients in each group experience a change of curve type over time and report which changes occurred.

***Response:*** There were no curve pattern changed in this group of participants. But curve pattern might be changed with long-term exercise and should be identified. Please see **Table 4**

***Comment #61:*** Discuss the imbalanced in the number of braced participant and possible brace compliance effects on results.

***Response:*** Only 10 subjects had brace treatment and half of them only used night time brace. The logistic regression showed bracing did not affect our outcome, therefore, it maybe not very valuable to go in details of this analysis due to our small sample size. Please see **lines 405 – 408.**

***Comment #62:*** Discuss whether after 2 years of follow-up these participants had reached skeletal maturity. How many had reached discharge point or were sufficiently far after peak growth velocity to deem the results final or not.

***Response:*** Fantastic point. **Table 4** was edited to this purpose.

***Comment #63:*** The SRS-SOSORT recommendations propose a number of analysis reporting guidelines. Could you report Risser signs in your dataset or table 1. Could you report number of patients with curve over 30 at each time point. I believe none exceeded surgery threshold of 45 or 50 but this could be specified.

***Response:*** **Table 1 has been amended** and see an updated **table 4.**

***Comment #64:*** The discussion could compare how your follow-up length and results compared to other Schroth studies. Are your results better than others or similar. Was the progression risk of your cohort similar or worse than others.

***Response:*** Fantastic point, related result and discussion were addressed accordingly. See **lines 339 to 344.**

***Comment #65:*** Please add a paragraph about whether there is a risk of overtreatment in this cohort. Could some of the patients have been left alone and avoid the burden of treatment. I can see that some would argue that for the fact that these patients possibly had no risk of progressing to bracing or surgery. However you showed some notable regression. This may appeal to some participants with small curves at low risk of progression. Still the possible overtreatment issue should be discussed.

***Response:*** We thank the reviewer for this important point. The discussion was amended accordingly. See **lines 353 to 356.**

***Comment #66:*** An important limitation in your study is that you may have been underpowered to detect difference between groups. It would be important to discuss how big a different in effects between curve types may be clinically important. This may be difficult to determine but a discussion of this topic should be presented.

***Response:*** We agree. This has been added to **line 417 to 425 and lines 446 to 449.**

***Comment #67:*** I would recommend against including the following in the discussion as you did not measure flexibility and did not study relation of effects of PSSE with baseline curve magnitude per say. However, further studies are necessary to address the correlation between spinal flexibility and the correction effects of PSSE at different curve magnitudes. IF you wish to keep then please move earlier in the discussion as suggestions for future research.

***Response:*** We thank the reviewer for this comment. Please see **lines 446 to 449.** We **re-centered** our discussion point in the introduction and discussion, respectively.

***Comment #68:*** I have annotated a few elements of the paper in an uploaded copy.

***Response:*** We thank the reviewer for these comments. Thanks for nice editing, the format was amended accordingly.

**Reviewer #3**

***Comment #1:*** The objective of this study is to investigate the comparative effectiveness of the PSSE correction effect on the Cobb angle between the thoracic and lumbar curves in AIS subjects. The authors considered a prospective clinical controlled study. While the study objectives sound interesting, a number of shotcomings were observed, in regards to abiding by the CONSORT guidelines for conducting and reporting results of high-quality randomized controlled trials (RCTs).

***Response:*** We thank the reviewer for these comments.

***Comment #2:*** 1. Abstract: The authors state results as: "A significant Cobb angle reduction was observed...", without any statement of p-values, estimated effect size, and its precision as confidence intervals, or CIs (say, 95\%). This can appear confusing, and half-baked to a reader. Check CONSORT checklist for Abstracts reporting of RCTs, and rewrite the Abstract following guidelines.

***Response:*** This was not an RCT study, therefore no needs for randomization etc, nonetheless, the problems in the abstract was recognized and amended.

***Comment #3:*** 2. Methods:

Methods reporting appeared very messy. An orderly manner is suggested, following CONSORT guidelines, without repeating information, such as Trial Design, Participant Eligibility Crtieria and settings, Interventions, Outcomes, sample size/power considerations, Interim analysis and stopping rules, Randomization (details on random number generation, allocation concealment, implementation), Blinding issues, etc. The authors are advised to create separate subsections for each of the possible topics (whichever necessary), and that way produce a very clear writeup.

***Response:*** Thanks for the valuable suggestions. We have revised the formatting.

***Comment #4:*** (a) For instance, the randomization and allocation concealment should be made very clear; the trial staff recruiting patients should not have the randomization list. Randomization should be prepared by the trial statistician, and he/she would not participate in the recruiting. I am confused; was randomization not done during the "Allocation" phase in your CONSORT diagram?

***Response:*** This was not an RCT, it was a CCT hence no randomization was required. Instead we controlled the curve pattern for the study.

***Comment #5:*** (b) I am surprized to see no statement on sample size/power in a manuscript proposing a (clinical) controlled trial. This is really the key here!

***Response:*** We thank the reviewer for the comment. The sample size estimation is in lines 192-196.

***Comment #6:*** (c) t-tests were used for assessing group differences for continuous variables (under the assumption of Normality). What if underlying normality assumptions are violated? Why not non-parametric (robust) tests were proposed?

***Response:*** Related clarification was addressed; normality was measured before commencing the analysis. Please see **lines 195 - 213**.

***Comment #7:*** (d) Similarly, the one-way repeated measures ANOVA may also be replaced/presented by a nonparametric Friedman-type test. I mean, justification is needed for the underlying Normality assumptions.

***Response:*** These have been clarified in the statistical analysis section.

***Comment #8:*** 3. Results & Conclusions:

(a) The authors should check that any statement of significance should be followed by a p-value in the entire Results section.

***Response:*** We thank the reviewer for this comment. This has been added.

***Comment #9:*** (b) The authors admitted a long list of limitations in their work, notably, the "...unable to blind the subjects, or physiotherapists...". Despite the justification provided, I am not sure how good the trial is! With 40 subjects recruited, the results stated, at best, can only be claimed as from a pilot study. This needs to be clearly stated, and the study cannot be claimed as a nicely planned randomized trial. Also, they need to state that future studies (with larger sample sizes) are warranted to really understand the comparative efficiacy.

***Response:*** We thank the reviewer for these comments. Please see the additions in **lines 410-441**.
