# Supplementary material for: Does curve pattern impact on the effects of physiotherapeutic scoliosis specific exercises on Cobb angles of participants with adolescent idiopathic scoliosis: A prospective clinical trial with two years follow-up
Source: PLoS One. 2021 Jan 25;16(1):e0245829. doi: 10.1371/journal.pone.0245829 (PMC7833215; doi:10.1371/journal.pone.0245829)
Supplement: Attachment — Submitted filename: Response to reviewers.docx [file pone.0245829.s009.docx]

REPLY TO REVIEWERS

**Re: Ms. No. PONE-D-19-35759 - "Does Curve Pattern Impact on the Effects of Physiotherapeutic Scoliosis Specific Exercises on Cobb angle of Adolescent Idiopathic Scoliosis: A prospective clinical controlled trial with two years follow-up”**

Dear Editor Alison Rushton,

The authors would like to thank you and the Reviewers for all of your time and effort devoted to the review of our aforementioned manuscript. The Reviewers’ comments were indeed extremely insightful and greatly appreciated. As such, the authors would like to take this opportunity to address each and every concern the Reviewers noted in their review of our submission. In addition, where appropriate, we have also revised our manuscript accordingly. Major changes are made in light gray.

We believe that the Reviewers’ comments have helped improve the quality of our manuscript. We hope that you and the Reviewers will find our revised work suitable for publication in *PLOS One.*

**Editor**

***Recommendation#1：***If applicable, we recommend that you deposit your laboratory protocols in protocols.io to enhance the reproducibility of your results. Protocols.io assigns your protocol its own identifier (DOI) so that it can be cited independently in the future.

***Response：***Thanks for recommendation. This is a clinical trial without laboratory protocols, this trial was online registered and the study protocol was open access on ***[www.chictr.org.cn](http://www.chictr.org.cn)***, trial registration: ChiCTR1900028073.

**Reviewer #2**

**Comment #1:** The Title should be corrected and show DOES not DOSE
Does curve pattern impact on the effects of physiotherapeutic scoliosis specific exercises on Cobb angles of participants with adolescent idiopathic scoliosis: a prospective clinical trial with two years follow-up

***Response：***We apologize for the typo. The title has been corrected.

**Comment #2:** Specify in the abstract that the study is a non-randomized prospective trial.

***Response：***Study design is specified in **line 17**.

**Comment #3:** In the abstract and the text line 203 The analysis should be announced as a mixed model anova (2 group by N time). Avoid stating 1 way as you have two factors. Group is a between groups factor and time is a within group factor.

***Response：***Analysis method was renamed accordingly, please see **line 24-25**.

**Comment #4**：Your sentence on line 231 to 235 is unclear. There is an important difference between groups in the numbers of patients with brace. Specify that in the text.

***Response：***This was specified on **lines 241 to 244.**

**Comments #5:** Also did all braced patients have a Cheneau? the sentence suggests you only talk about those wearing a Cheneau brace but does not clearly state whether other types were used. Specify at what point correction was measured. Do you mean out of brace at the end of the study or in-brace and if yes then when was in-brace correction measured.

***Response:*** 1. All braced subjects were wearing Cheneau brace, sorry for this confusion, clarification was made on **lines 237 to 239**.

2. The in-brace correction was the initial in-brace correction (a percentage value of Cobb reduction during an X-ray with the brace fitted on the participant more than two hours), which was measured after a brace adaption period (two weeks). Please see **lines 136 to 138 and lines 188 to 190**.
3. Regarding the Cobb angle at the end of the study, it was measured without brace and participants were required off-bracing at night prior to X-ray measurement in the next day. Please see **lines 190 to 191**.

**Comment #6:** Abstract. The following sentence in the abstract could be replaced by results of your regression. Your objective was focused on effect of curve type. This sentence does not add to this objective: In addition, 65% of 37 participants (n=26) reached skeletal maturity (Risser sign = 5) and achieved significant reductions 38 in progression risk at the 2-year follow-up (Lonstein and Carlson Risk of progression: 0.45 ± 0.32 39 vs. 0.13 ± 0.14, p <0.001).
Obviously, patients reaching maturity would have significantly reduced progression risk. If you keep this idea in the abstract, please specify what this means with regards to their curve types or having done exercises.

***Response:*** Fantastic suggestion. We re-structured the result of abstract and emphasized result of correlation of curve pattern with curve regression. Please **see lines 36 to 38.**

***Comment #7:*** In the consort flow chart please specify the number of cases that were excluded because they did not attend all the prescribed exercises sessions. (specify none did if that is the case)

***Response:*** All participants completed prescribed exercise sessions. The clarification was further edited in **S2 Fig. CONSORT flow diagram** and **line 127.**

***Comment #8:*** Figure 2 f. The picture is stretched too wide. Please present in the original aspect ratio.

***Response:*** The picture (Fig.2) is updated with original aspect ratio. Please check **line 168.**

***Comment #9:*** Page 13 line 203. Specify how you calculated D-value. Did you do for example 6 months minus baseline or basline minus 6 months. Specifying will help interpret mean d-values as indicating improved curves or deterioration.

***Response:*** Thanks for this valuable suggestion. Specifying was made on **lines 206 to 209.**

***Comment #10:*** P14 Line 206 Please refer to comment above about using mixed model anova and not one way ANOVA.

***Response:*** Thanks for this reminding and correction was addressed on **line 211.**

***Comment #11:*** L218 It would be interesting to see the distribution of the hypermobility cases excluded in terms of their presenting a thoracic or lumbar dominant pattern given that you suspected a higher relation with single thoracic curves.

***Response:*** The distribution of noted hypermobility in this study was reported on **lines 224 to 225.** We did not reproduce the same distribution as the previous study, yet we did find a few of patients presenting hypermobility regarding the Beighton score. The distribution of hypermobility in AIS can be explained better with a population level, cross-sectional study.

***Comment #12:*** P14 Is single left lumbar atupical? Single right lumbar would be more atypical? Is this an error? Similarly, with single left thoracolumbar. Is this really atypical?

***Response:*** This is a really good question. Proven atypical curve patterns include a single left thoracic curve, a curvature with a loss of kyphosis, a rapid progression and ≤ 6 segmental scoliosis, etc. The single right lumbar and a left thoracolumbar were reported as atypical pattern in previous studies and in which subjects were found having an underlying syrinx presented with a MRI assessment. Therefore, cases with aforementioned curve pattern were excluded to control study heterogeneity yet prescribed with either a neurological assessment or an MRI assessment before commencing any treatments. We cited the related studies, please **check lines 225 to 227 and references 35 to 38.**

***Comment #13:*** L229 to 231. I continue to disagree that you should compare baseline characteristics between groups statistically. Instead you should report what appears to be clinically important differences and maybe announced that this would be justification to explore these characteristics as covariables later. The study did not plan its sample size to detect such differences. The key is to explore if they play a role in confounding the results later.

***Response:*** Sure, we do agree that defining confounding factors is way more important than comparing the baseline statistically in a clinical trial, Thus, baseline characteristics were reported by descriptive statistics in this updated manuscript. Please check **Table 1.** In addition, bracing was set as a covariate with repeated measures to evaluate effects of PSSE in reducing Cobb angles.

***Comment #14:*** L230 Please spell out what LCR stands for.

***Response:*** Lonstein Carlson Risk of progression (LCR-value) was firstly explained on the **line 205,** then the abbreviated term, LCR-value was adopted throughout manuscript.

***Comment #15:*** L231. The imbalance in bracing may not have reached significance but it should be pointed out.

***Response:*** Sure indeed, this was emphasized on the **lines 241 to 244.**

***Comments #16:*** L237 Table 1. If you keep the p values add which test, they came from in the table legend on in the column with the p-values. This would help the informed reader determine if your test likely had enough power.

***Response:*** We used descriptive statistics for baseline characteristics in this updated manuscript, thus no *p* value was reported **in the table 1**, but we adopted t-test and paired t-test to compare the exercise compliance of each year, which would help us on discussing how did exercise compliance affect the outcome. Thus, a table legend was made accordingly on the **lines 250 to 252.**

***Comment #17:*** L237 Here or in the methods state when the in-brace Cobb angle was measured.

***Response:*** The timepoint of measuring in-brace Cobb angle was stated on the **lines 136 to 138.**

***Comment #18:*** L237 Refer to 1st year and 2nd year in the exercise compliance please.

***Response:*** Done. Please check the **Table 1**.

***Comment #19:*** L246 Since in the analysis section you left 2 choices for testing this different please report what test was used to compare the groups here with the p-value.

***Response:*** This is a subgroup analysis to detect any changes of exercise compliance between groups on the 1^st^ and 2^nd^ year follow-up. Hence, an independent *t-test*, and paired *t-test* were used to compare the exercise compliance between and within groups. Please check **lines 256 to 257.**

***Comment #20:*** L248 Similarly. Please report the test used from comparisons within the thoracic group.

***Response:*** This is a subgroup analysis to detect any changes of exercise compliance between groups on the 1^st^ and 2^nd^ year follow-up. Hence, an independent *t-test*, and paired *t-test* were used to compare the exercise compliance between and within groups. Please check the **lines 256 to 257.**

***Comment #21:*** L267 Table 2. Instead of group labels of 0 and 1 can you spell thoracic and lumbar or use T and L for clarity?

***Response:*** Thanks for this suggestion. Revision was made on the **Table 2.**

***Comment #22:*** L272 to 279. This analysis offers more information than that of the D-values. I would only report this ANOVA and possibly compared the frequencies of deterioration, stabilized or improved statistically as well. There is in fact a lot of redundancy between the analyses in table 2 and 3.

***Response:*** Agree. Thanks for this fantastic suggestion to make our points much clearer. Hence, we withdrew the previous table 2 (repeated measures of D-value) and re-centered our primary analysis on the raw Cobb angles. Please see **lines 269 to 286**, and the updated **table 2.**

***Comment #23:*** L289. It is less clear why this second case is highlighted. Only because of the large baseline curve? If yes specify that this case it highlighted because or the large curve. I was wondering if it was because it was the second case having refused the brace. Please clarify.

***Response:*** The second case refused the brace. The clarification was made on the **lines 320 - 321**.

***Comment #24:*** L309 To 311. It is redundant to repeat the baseline mean in each of the parentheses. Clarify which test produced the p-values. This set of results is surprising as it would suggest that the interaction should be significant the pattern of differences between timepoints was not the same between the two groups, yet you report that the interactions was not significant. Since the interaction was not significant you should not put emphasis on the different in changes over time between the groups.

***Response:*** Thanks for this comment, the redundancy was deleted. Please check the re-structured paragraphs on **lines 294 to 321**, using descriptive statistics to illustrate the trend of scoliosis regression and stabilization in each group. The repeated measures with Bonferroni adjustment showed a significant time-effect in reducing Cobb angles (raw value) over four testing times. Additionally, post hoc analysis showed the significant reductions of Cobb were occurred on the 1.5- and 2-year follow-up, respectively. Therefore, we used descriptive statistics, additionally to illustrate how many participants in which group showed clinical curve regression. In case of we withdrew the repeated measures of D-value of Cobb angles, hence the previous redundancy was completely deleted.

***Comment #25:*** L280 to 287. 312. To 317 Ideally the frequency results of the two groups would be reported together in terms of whether the distributions of improved, stable or deteriorated were similar or not between groups. Specify which test was used to test for those differences in the distributions.

***Response:*** Fantastic suggestion, please see the re-structured paragraphs on **lines 294 to 321.** The distribution of scoliosis progression, stabilization and regression was analysed using Chi-square analysis. Please see **lines 296 to 298**.

***Comment #26:*** L321 322. Focusing on a reduction in the progression risk is possibly misusing the formula of Lonstein and Carlson since it uses age and Risser so heavily and you did not report the formula in your paper. The change in angles would not play a clear role and this may mislead readers to think the reduction is due to the PSSE when maturity plays a big role. Their study possibly did not examine patients starting this late to really use the equation at this stage of treatment. If you keep this analysis, please report the full formula and add a discussion related to maturation in the discussion in relation to this reduced risk.

***Response:*** Fantastic point, thanks for pointing it out, we realized that comparing LCR-value after two years follow-up was inappropriate. Therefore, we withdrew the comparison of LCR-value at the 2-year follow-up and replaced it to a description of how much participants reached skeletal maturity (Risser 5) and presented with a curve less than 30 degrees. Please see **lines 294 to 295 and Table 3.**

***Comment #27:*** L328. Please add units for Age, the Cobb angle mean lines

***Response:*** Done, Thanks for this suggestion. Please see **line 323 (Table 3)**.

***Comment #28:*** L323 Your study likely did not have the power to examine so many variables in the logistic regression. Since your focus is on the curve type. Can you report the OR for the association between curve type and the 2 year curve outcomes? Once you enter multiple categorical predictors there really isn’t a lot of observations with the regression outcome to determine prediction estimates.

***Response:*** Nice point, thanks for pointing it out, we now only enrolled curve pattern and bracing (since we enrolled bracing into repeated measures) into the logistic regression model to further illustrate the effects of bracing and curve pattern in scoliosis regression with PSSE. Please see **lines 299 to 302.** However, this logistic regression was underpowered since this study was not originally designed for a regression analysis, thus, we acknowledged this limitation on the **lines 449 to 454**.

***Comment #29:*** L351 to 353. You cannot claim you demonstrated Superiority of PSSE. You did not compare PSSE to an alternate intervention. You showed all patients receiving PSSE have avoided curve progression but not superiority to other treatments or to natural history (unless you refer to historical control and predictions given the progression risk.)

***Response:*** Agree, we replaced “superiority of PSSE” by “effectiveness of PSSE” in preventing scoliosis progression. Please see **lines 358 to 359.**

***Comment #30:*** L359 to 361. Again, I disagree with emphasizing the change in progression risk. I do not believe the formula can be used this way. Further once again you omit to emphasize that much of this reduce regression risk is due to the change in age and Risser. It is misleading to imply the change is mostly due to the effect of PSSE and reduced Cobb angles. It is good indeed that curves did not progress, and patients are now close to maturity but here it would be more relevant to refer to natural history data showing how patients with certain curve magnitude at maturity progress into adulthood. EG.

***Response:*** Fantastic point, thanks for pointing it out, we realized that comparing LCR-value after two years follow-up was inappropriate. Therefore, we withdrew the comparison of LCR-value at the 2-year follow-up and replaced it to a description of how much participants reached skeletal maturity (Risser 5) and presented with a curve less than 30 degrees. Please see **lines 366 to 368 and Table 3.**

***Comment #31:*** L366 to 369 again you emphasise a different in the patterns of curve angles changes over time between groups when you ANOVA suggested that there was no interaction. There could be data distribution issues affecting the results here. Which ANOVA model did you end up using? Was your data meeting all other assumptions than sphericity?

***Response:*** Sorry for this confusion, agree that if there were no interaction effects in reducing Cobb angles, it was inappropriate to further compare variables between groups. In this study, the significance was only detected in the within-subjects comparison, having a time effect in reducing Cobb angles for all participants (observed power 0.7, **lines 275 - 278**). A separate repeated analysis with a Bonferroni adjustment (control type I error) further revealed that the significant reduction of Cobb angles was dominant in the lumbar group (observed power 0.95, **lines 282 - 283**). Therefore, we rewrote this part (please see **lines 371 to 372**). Levene’s test indicated that Cobb angles at the baseline met homogeneity of variance assumption (*p*=0.5). Mauchly test indicated that the assumption of sphericity was violated (χ^2^ =32.1 , df =5 , *p* < 0.001), hence, the degrees of freedom were corrected using Greenhouse-Geisser estimates of sphericity. Please see **lines 271 to 276**. The assumption of Greenhouse-Geisser was met with an Epsilon value 0.72.

***Comment #32:*** L371 What do you refer to when stating “Progression value was comparable between groups at the study initiation”?

***Response:*** Sorry for the confusion, the comparable here means the proportion was similar between groups, the clarification was made on **lines 376 to 378.**

***Comment #33:*** L372 373 Since you did not measure spinal flexibility please refer instead to “by the assumed more important flexibility of the lumbar spine than the thoracic spine.

***Response:*** Fantastic points. Please see the reediting on **lines 378 to 379**.

***Comment #34:*** L378 to 381. I would avoid suggesting that muscle actions have a bigger role for lumbar corrections and that only spiral breathing play a role in the thoracic regions. Modern Schroth instruction does not make assumptions about what muscles do or how corrections are done it simply offers a variety of cues and correction instructions and facilitation technique to get the desired movement without emphasis on how.Maybe the key is that breathing is more related to thoracic ok. The axial elongation, side shift are done in both regions. The difficulty with breathing is likely relevant.

***Response:*** Nice points, thanks for this explanation, the revised discussion was made on the **lines 385 to 387*.***

***Comment #35:*** L405. Please cite Kuru et al for the effects of supervision vs not.

***Response:*** cited. Thanks for this recommendation. Please check **reference 49**.

***Comment #36:*** L417 There are more types in the whole BSPTS classification. Maybe instead rephrase to suggest that your sample included patients representing 5 different types from 8 in the BSPTS classification.

***Response:*** Nice points, thanks for this explanation, the revised discussion was made on **lines 423-424.**

***Comment #37:*** L435 Refer to hypothesized higher flexibility or previously shown higher flexibility. You did not show that in your study.

***Response:*** Thanks for this reminding, we acknowledge this limitation on **lines 444 to 445.**

***Comment #38:*** In this limitation section please clearly acknowledge that your regression is underpowered.
***Response:*** Indeed. The acknowledgement was made on **lines 448 to 454.**

***Comment #39:*** In the data supplementary file please replace the variable name by study id. Remove initials and replace by participant id numbers to fully comply with confidentiality of study data.

***Response:*** Done, thanks for this important recommendation. Please check the updated **S4 File. Study data.**

***Comment #39:*** I have added edits in the text using the PDF comment function with strikethrough for deletions and insertions in blue.

***Response:*** We have revised the format accordingly. Deeply appreciate your detailed comments.
